# Identification and development of a novel 5-gene diagnostic model based on immune infiltration analysis of osteoarthritis

**DOI:** 10.1186/s12967-021-03183-9

**Published:** 2021-12-23

**Authors:** YaGuang Han, Jun Wu, ZhenYu Gong, YiQin Zhou, HaoBo Li, Bo Wang, QiRong Qian

**Affiliations:** 1 Department of Joint Surgery and Sports Medicine, Shanghai Changzheng Hospital, Second Military Medical University, 415#, Fengyang Road, Huangpu District, Shanghai, 200003 China; 2 Department of Orthopaedic Surgery, Nantong Sixth People’s Hospital, Nantong Hospital Affiliated To Shanghai University, Nantong, Jiangsu China; 3 Department of Emergency Medicine, Zhongshan Hospital, Fudan University, Shanghai, 200032 China

**Keywords:** Osteoarthritis, Biomarker, WGCNA

## Abstract

**Background:**

Osteoarthritis (OA), which is due to the progressive loss and degeneration of articular cartilage, is the leading cause of disability worldwide. Therefore, it is of great significance to explore OA biomarkers for the prevention, diagnosis, and treatment of OA.

**Methods and materials:**

The GSE129147, GSE57218, GSE51588, GSE117999, and GSE98918 datasets with normal and OA samples were downloaded from the Gene Expression Omnibus (GEO) database. The GSE117999 and GSE98918 datasets were integrated, and immune infiltration was evaluated. The differentially expressed genes (DEGs) were analyzed using the limma package in R, and weighted gene co-expression network analysis (WGCNA) was used to explore the co-expression genes and co-expression modules. The co-expression module genes were analyzed by Gene Ontology (GO) and Kyoto Encyclopedia of Genes and Genomes (KEGG) analyses. A protein–protein interaction (PPI) network was constructed using the Search Tool for the Retrieval of Interacting Genes/Proteins (STRING) database, and hub genes were identified by the degree, MNC, closeness, and MCC algorithms. The hub genes were used to construct a diagnostic model based on support vector machines.

**Results:**

The Immune Score in the OA samples was significantly higher than in the normal samples, and a total of 2313 DEGs were identified. Through WGCNA, we found that the yellow module was significantly positively correlated with the OA samples and Immune Score and negatively correlated with the normal samples. The 142 DEGs of the yellow module were related to biological processes such as regulation of inflammatory response, positive regulation of inflammatory response, blood vessel morphogenesis, endothelial cell migration, and humoral immune response. The intersections of the genes obtained by the 4 algorithms resulted in 5 final hub genes, and the diagnostic model constructed with these 5 genes showed good performance in the training and validation cohorts.

**Conclusions:**

The 5-gene diagnostic model can be used to diagnose OA and guide clinical decision-making.

**Supplementary Information:**

The online version contains supplementary material available at 10.1186/s12967-021-03183-9.

## Introduction

Osteoarthritis (OA) is the most common chronic bone and joint disease in modern society, and it is a serious threat to human health and quality of life. According to the statistics of the World Health Organization (WHO), about 9.6% of men and 18% of women over 60 years of age worldwide have OA. In developed countries, 25% of patients with OA have disabilities. The chronic pain and dysfunction experienced by patients with OA and the heavy economic burden caused by OA are important health problems faced by today’s aging society [1]. The main features of OA are (1) progressive degeneration of articular cartilage, (2) the appearance and formation of osteophytes, (3) the appearance of synovial inflammation, (4) thickening of subchondral bone, and (5) narrowing of the knee joint space [2]. A variety of factors, such as age, sex, obesity, stress injury, trauma, and congenital joint abnormalities, can cause OA [3].

At present, the main OA treatments are non-steroidal anti-inflammatory drugs and joint replacement [4]. In recent years, the incidence of OA has shown a younger trend. At present, the pathogenesis of OA is not completely clear. Therefore, early detection, early diagnosis, and early treatment are key to improve OA prognosis.

In recent years, many researchers have devoted themselves to exploring biomarkers for OA prevention, diagnosis, and monitoring of disease progression. Some studies have shown that the combined detection of serum chondroitin sulfate 846 epitope (CS846) and cartilage oligomeric matrix protein (COMP) can be used to diagnose and monitor the progression of OA [5]. Compared with healthy people, the concentration of type II collagen C-terminal peptide (CTX-II) in the synovial fluid of patients with early OA is higher [6]. The level of COMP can be used as a marker of the occurrence rather than the progression of hip and knee OA [7]. C-reactive protein (CRP) is related to the occurrence and progression of knee OA [7, 8]. The analysis of urine samples from patients with knee OA has shown that metabolite levels may be helpful to predict the progression of OA. Glycolic acid, hippuric acid, and fenugreek are of great significance for distinguishing patients who are prone to OA progression. However, due to the small sample size of the above research, the results have some limitations [9]. At present, the biomarkers that can be used in clinical applications are still very limited.

In this study, we comprehensively analyzed several public microarray datasets to evaluate the Immune Scores of OA and normal samples, and we identified genes related to OA immune infiltration using weighted gene co-expression network analysis (WGCNA). The potential transcriptome biomarkers of OA were identified, and a protein–protein interaction (PPI) network involved in these immune infiltration-related genes was constructed to identify OA diagnostic biomarkers. A diagnostic model for predicting and preventing OA was constructed based on the pattern recognition of support vector machines (SVMs).

## Methods and materials

### Data sources and data download

Normal and OA samples were selected from the Gene Expression Omnibus (GEO) database. The expression datasets of OA (GSE129147 [10], GSE57218 [11], GSE51588 [12], GSE117999, GSE98918 [13]) and a methylation dataset (GSE73626 [14]) were downloaded.

### Data preprocessing

The GEO datasets were processed by keeping the normal and OA samples, converting the probes to Gene Symbol, removing the probes corresponding to multiple genes, and taking the medians of multiple Gene Symbols. The GSE117999 and GSE98918 datasets are from the same platform (GPL20844), and the batch effect was eliminated through the removeBatchEffect function of the limma package in R [15].

### Immune infiltration analysis

For the gene set enrichment (GSE) integrated dataset, ESTIMATE software was used to evaluate 3 scores: Stromal Score, Immune Score, and ESTIMATE Score. MCPcounter was used to evaluate the scores of 10 immune cells. Single-sample gene set enrichment analysis (ssGSEA) was used to evaluate the scores of 28 immune cells. The Spearman correlation coefficients of these Immune Scores were calculated.

### DEG identification and WGCNA

The limma package in R was used to identify the differentially expressed genes (DEGs) between the normal and OA samples of the GSE integrated dataset, and they were filtered according to the threshold false discovery rate (FDR) < 0.05 and |Fold Change|> 1.2. The final DEGs were obtained. According to these DEGs’ expression profiles, we use the WGCNA algorithm to identify co-expression genes and co-expression modules. First, the expression profiles of the DEGs in the GSE integrated dataset were extracted. The Pearson correlation coefficient was used to calculate the distance between each gene, and a weighted gene co-expression network was constructed by the WGCNA package in R. The soft threshold was 12, and the co-expression module was identified. The next step was to transform the expression matrix into an adjacency matrix and then transform the adjacency matrix into a topological matrix. Based on the topological overlap measure (TOM), genes were clustered by the average-linkage hierarchical clustering method. According to the standard of the hybrid dynamic shearing tree method, the minimum number of genes for each gene network module was set to 50. We then calculated the eigengenes of each module in turn, then cluster analysis was performed on the modules. Modules that were close to each other were merged into a new module, and the settings of set height = 0.25, deepSplit = 2, and minModuleSize = 50 were used to obtain the final modules.

### KEGG and GO analyses and PPI network construction

The WebGestaltR (v0.4.2) package in R was used to perform Kyoto Encyclopedia of Genes and Genomes (KEGG) pathway analysis and Gene Ontology (GO) functional enrichment analysis on the modular genes most significantly positively correlated with Immune Score. The Search Tool for the Retrieval of Interacting Genes/Proteins (STRING) database was used to analyze the PPI network of the above genes, and Cytoscape (v3.7.2) was used to identify the network modules in the resulting file.

### Hub gene identification

For the PPI network of the DEGs, we use the degree, MNC, closeness, and MCC algorithms of the cytoHubba plug-in of Cytoscape (v3.7.2) to calculate XX. The first 10 genes were selected as the key genes. The key genes obtained by these algorithms were intersected to identify the final hub genes.

### Diagnostic model construction and validation

The GSE integrated dataset was used as the training dataset, and the GSE57218, GSE129147, and GSE51588 datasets were used as the validation datasets. The hub genes were used as the features in the training dataset, and the corresponding expression profiles were obtained. An SVM classification model was constructed, and the sensitivity, specificity, and area under the receiver operating characteristic (ROC) curve (AUC) of the model were analyzed.

## Results

### Flow chart

**Fig. 1:**
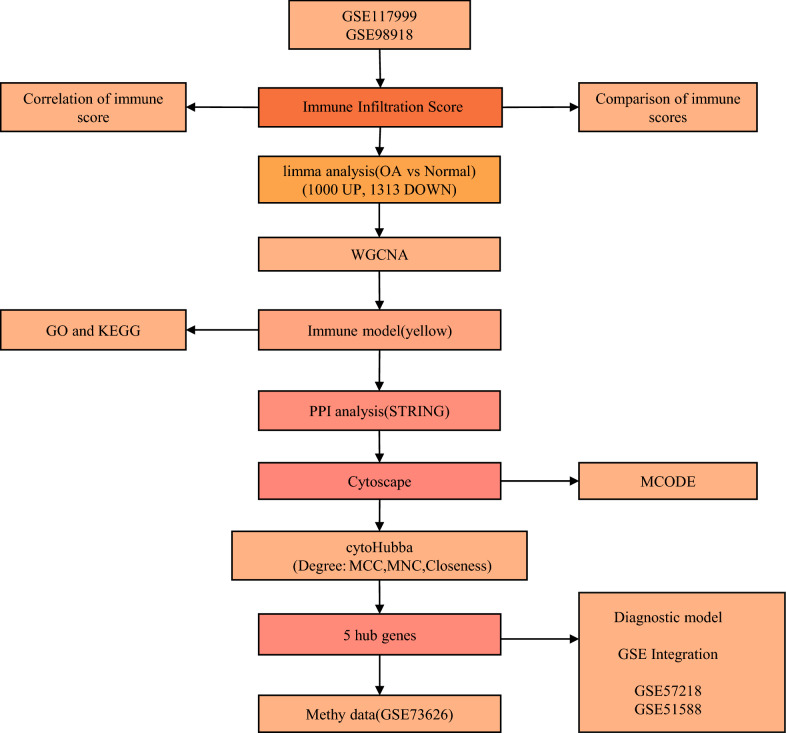
Flow chart of OA immune infiltration analysis and diagnostic model construction and validation

A flow chart of the immune infiltration analysis and diagnostic model construction and validation is shown in Fig. 1.

### Data preprocessing

**Fig. 2:**
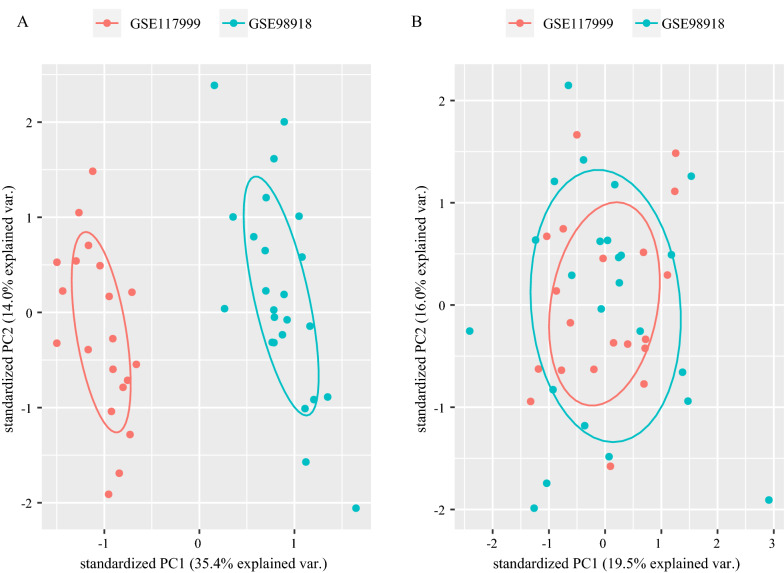
Data preprocessing. **A** PCA diagrams of the GSE117999 and GSE98918 datasets before; **B** after batch effect elimination

The GSE117999 and GSE98918 datasets are from the GPL20844 platform. Principle component analysis (PCA) diagrams before and after batch effect elimination are shown in Fig. 2. The GSE117999 and GSE98918 datasets were integrated and named the GSE integrated dataset, which contained 22 normal samples and 22 OA samples.

The preprocessed GSE129147 dataset had 9 normal samples and 10 OA samples; the GSE57218 dataset had 7 normal samples and 33 OA samples; the GSE51588 dataset had 10 normal samples and 10 OA samples; the GSE117999 dataset had 10 normal samples and 10 OA samples; the GSE98918 dataset had 12 normal samples and 12 OA samples; and the GSE73626 dataset had 7 normal samples and 11 OA samples. The clinical statistics of the samples can be found in Table 1.

**Table 1:** Clinical information of the samples

Data set	Expression		Platforms
GSE129147			
Normal	9		GPL15207
OA	10		
GSE57218			
Normal	**7**		GPL6947
OA	33		
GSE51588			
Normal	10		GPL13497
OA	40		
GSE117999			
Normal	10		GPL20844
OA	10		
GSE98918			
Normal	12		GPL20844
OA	12		
GSE Integration			
Normal	22		GPL20844
OA	22		
GSE73626			
Normal	7		GPL13534
OA	11		

### Immune infiltration analysis of the integrated dataset

**Fig. 3:**
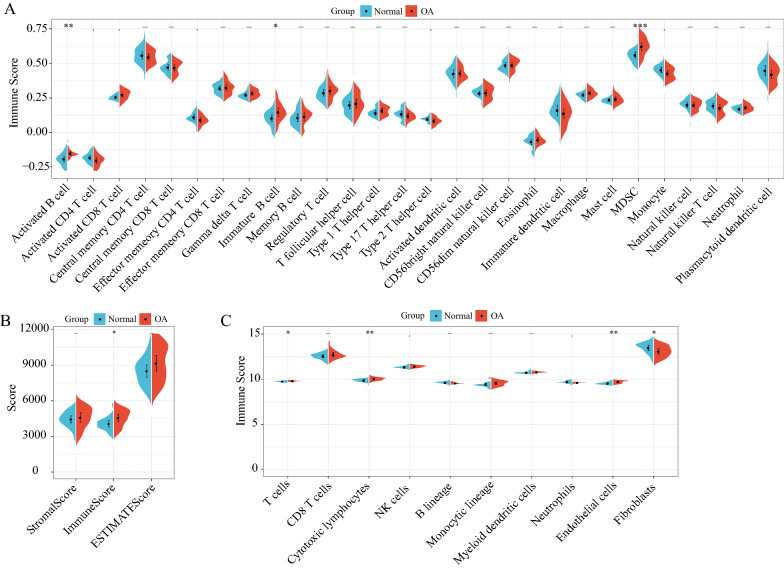
Immune infiltration analysis of the integrated dataset. **A** Comparisons of the ssGSEA Immune Scores; **B** ESTIMATE Immune Scores; **C** MCPcounter Immune Scores of the integrated dataset OA and normal samples

The integrated dataset of GSE117999 and GSE98918 was analyzed for immune infiltration. The scores of the 28 immune cells evaluated using ssGSEA showed that activated B cells, immature B cells, and myeloid-derived suppressor cells (MDSCs) were significantly higher in the OA samples than in the normal samples (Fig. 3A). The Stromal Scores, Immune Scores, and ESTIMATE Scores evaluated by the ESTIMATE software showed that the Immune Score was significantly higher in the OA samples than in the normal samples (Fig. 3B); the scores of the 10 immune cells evaluated by MCPcounter showed that T cells, cytotoxic lymphocytes, and endothelial cells were significantly higher in the OA samples than in the normal samples (Fig. 3C).

### Identification of DEGs in the integrated dataset

**Fig. 4:**
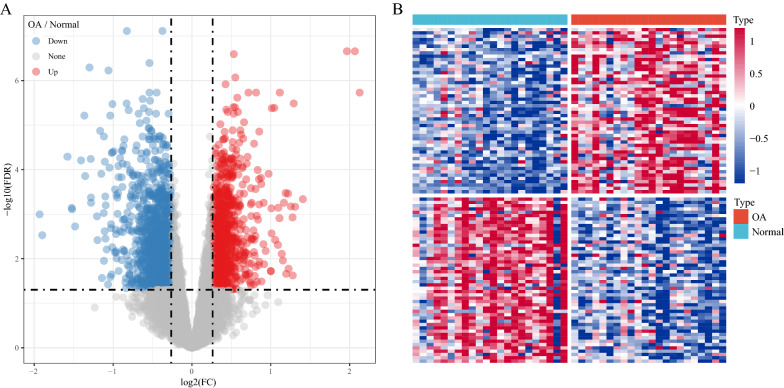
Identification of DEGs in the integrated dataset. **A** Volcano map of the DEGs in the integrated dataset. **B** Heat map of some of the DEGs

The limma package in R was used to analyze the DEGs between the normal and OA samples. After filtering the data, 2313 DEGs were identified, of which 1000 genes were upregulated and 1313 genes were downregulated (Additional file 1: Table S1). A volcano map of the upregulated and downregulated DEGs is shown in Fig. 4A, and a heat map of the first 50 upregulated and downregulated genes is shown in Fig. 4B.

### WGCNA and KEGG pathway analysis and GO functional enrichment analysis

**Fig. 5:**
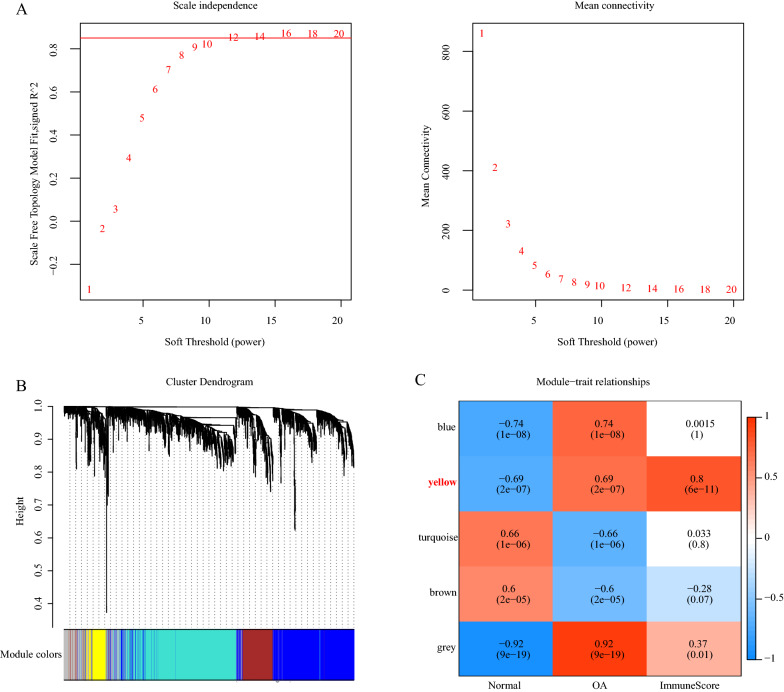
WGCNA analysis. **A** Correlation between the 5 modules in the network topology analysis for various soft-thresholding powers. **B** Gene dendrogram and module colors. **C** Correlation between the 5 modules and Immune Scores

According to the expression profiles of the DEGs, the WGCNA algorithm was used to identify the co-expressed genes and modules. To ensure that the network was scale-free, β = 12 was chosen (Fig. 5A). The gene expression matrix was transformed into an adjacency matrix, then the adjacency matrix was transformed into a topological matrix. Finally, 5 modules (Fig. 5B) were obtained. The correlation between each module and sample type (OA or normal) and Immune Score was further analyzed (Fig. 5C). The module with the most significant positive correlation with Immune Score was the yellow module, which also had a significant positive correlation with the OA samples and a significant negative correlation with the normal samples. The yellow module contained 142 genes, of which 136 were upregulated and 6 were downregulated. The genes contained in the module are shown in Additional file 2: Table S2.

**Fig. 6:**
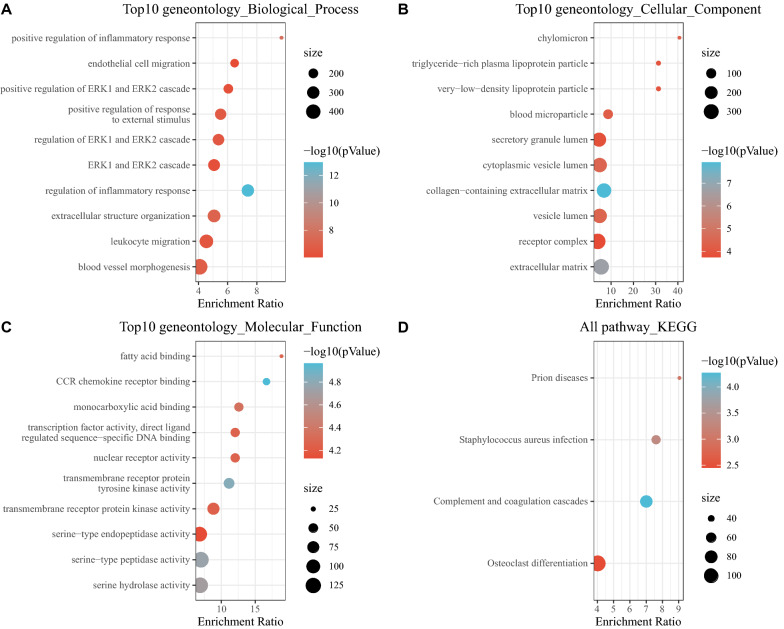
KEGG pathway analysis and GO functional enrichment analysis. **A** BP annotation diagram; **B** CC annotation diagram; **C** MF annotation diagram; D. KEGG annotation diagram of the yellow module genes

KEGG pathway analysis and GO functional enrichment analysis were performed on the co-expressed genes of the yellow module. A total of 661 biological processes (BPs), 48 molecular functions (MFs), 27 cellular components (CCs), and 4 pathways were enriched among the DEGs (P < 0.05). For BPs, the top 10 enriched items mainly participated in regulation of inflammatory response, positive regulation of inflammatory response, blood vessel morphogenesis, endothelial cell migration, humoral immune response, and other biological processes (Fig. 6A). For CCs and MFs, the top 10 enriched items are shown in Fig. 6B and C, respectively. The KEGG pathway enrichment analysis results suggested that the DEGs predominantly participated in the complement and coagulation cascades and osteoclast differentiation pathways (Fig. 6D).

### Identification of hub genes

**Fig. 7:**
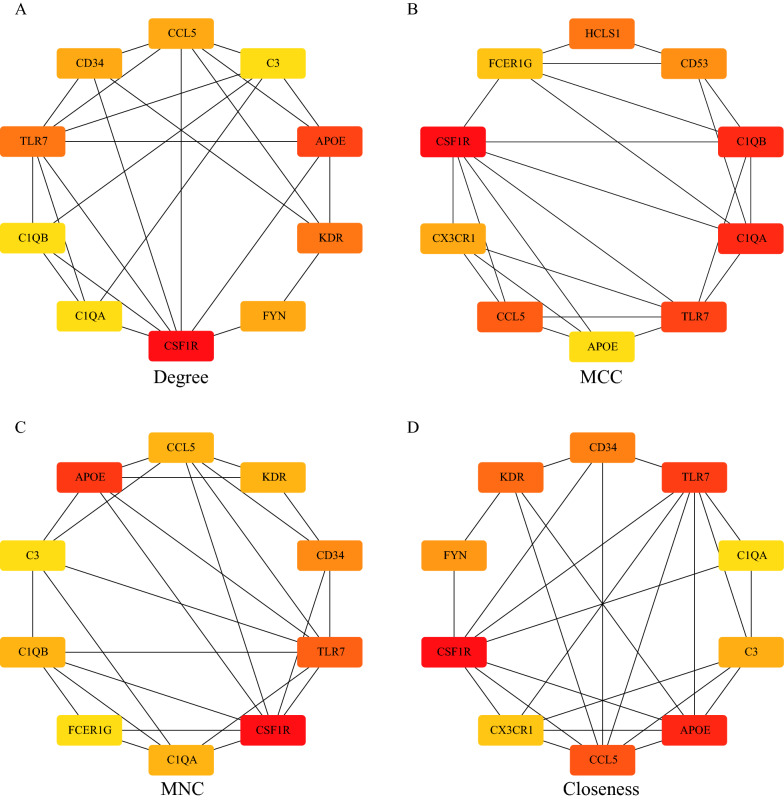
PPI network diagram used by 4 algorithms. **A** Degree algorithm; **B** MCC algorithm; **C** MNC algorithm; **D** Closeness algorithm

**Fig. 8:**
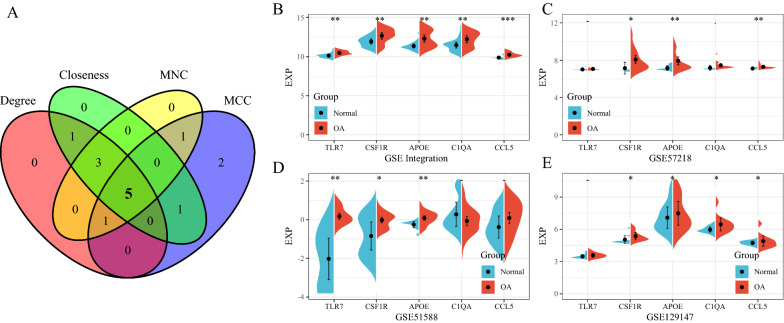
Comparison the expression of the 5 hub genes in different datasets. **A** Venn diagram of the identified hub genes. **B** Comparison the expression of the 5 hub genes in the GSE integration datasets; **C** Comparison the expression of the 5 hub genes in the GSE57218 datasets; **D** Comparison the expression of the 5 hub genes in the GSE51588 datasets; **E** Comparison the expression of the 5 hub genes in the GSE 129,147 datasets

For the 142 genes in the yellow module, the STRING database was used to construct a PPI network. The degree, MNC, closeness, and MCC algorithms of the cytoHubba plug-in of Cytoscape (v3.7.2) were used to calculate the PPI network of the 142 DEGs, and the top 10 genes were selected as the key genes. The PPI network diagram of the genes identified by these 4 algorithms is shown in Fig. 7. Taking the intersections of the hub genes obtained by these algorithms, a Venn diagram was constructed (Fig. 8A). Finally, 5 genes, including *TLR7*, *CSF1R*, *APOE*, *C1QA*, and *CCL5,* were obtained. In addition, we compared the expression of the 5 hub genes in the OA and normal samples in the different datasets (Fig. 8B–E). The gene expression in the OA samples was significantly higher than in the normal samples.

### Construction and validation of the diagnostic model

We used the GSE integrated dataset as the training cohort and the GSE57218, GSE129147, and GSE51588 datasets as the validation cohorts.

**Fig. 9:**
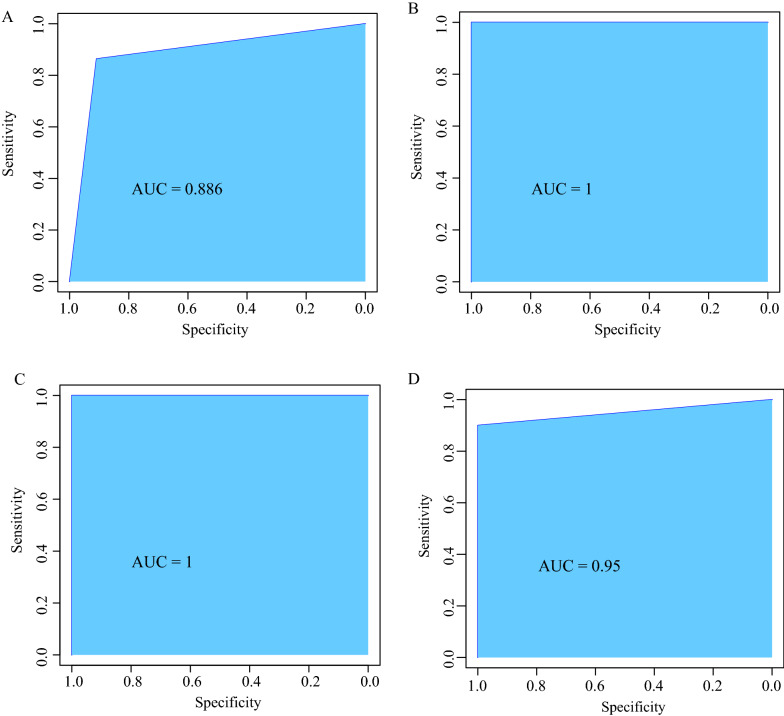
Construction and validation of the diagnostic model. **A** Classification results and ROC curves of the diagnostic model in the GSE integrated dataset; **B** In GSE57218 dataset; **C** In GSE129147 dataset; **D** In GSE51588 dataset

The 5 hub genes were used as features in the training cohort, and an SVM classification model was constructed. The classification accuracy rate was 88.6%. Of the 44 samples, 39 were correctly classified. The sensitivity of the model was 91%, the specificity was 86.4%, and the AUC was 0.886 (Additional file 3: Fig. S1A, Fig. 9A). The GSE57218 dataset was used for verification, and the results showed that 40 of the 40 samples were correctly classified; the classification accuracy rate was 100%, the sensitivity was 100%, the specificity was 100%, and the AUC was 1 (Additional file 3: Fig. S1B, Fig. 9B). The GSE129147 dataset was also used for verification, and the results showed that 19 of the 19 samples were correctly classified; the classification accuracy rate was 100%, the sensitivity was 100%, the specificity was 100%, and the AUC was 1 (Additional file 3: Fig. S1C, Fig. 9C). Finally, the GSE51588 dataset was used for verification, and the results showed that 49 of the 50 samples were correctly classified; the classification accuracy rate was 98%, the sensitivity was 100%, the specificity was 90%, and the AUC was 0.95 (Additional file 3: Fig. S1D, Fig. 9D). These results indicated that the diagnostic prediction model constructed in this study could effectively distinguish OA samples from normal samples, and the 5 genes could be used as reliable biomarkers for OA diagnosis.

### Methylation of the hub genes

**Fig. 10:**
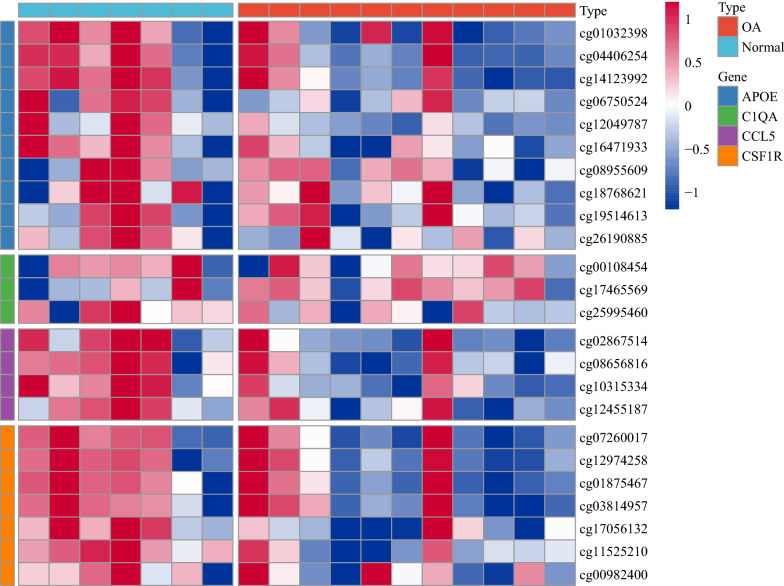
Methylation at the methylation sites of some of the hub genes

The methylation of the 5 hub genes in the normal and OA samples was analyzed using the GSE73626 dataset. The methylation at the methylation sites of the *CSF1R*, *APOE*, *C1QA*, and *CCL5* genes in the normal samples was greater than in the OA samples (this information for the *TLR7* gene in the GSE73626 dataset was not available), which was consistent with the fact that the expression of these 4 genes in the normal samples was lower than in the OA samples. There was a negative correlation between the degree of methylation and gene expression (Fig. 10).

## Discussion

OA is a chronic degenerative joint disease that is part of the aging process. In addition to synovitis, it is characterized by the loss and degeneration of articular cartilage, resulting in joint stiffness, swelling, pain, and loss of mobility [16, 17]. OA is a major public health problem [18]. It has been listed as the fastest growing major health condition by the WHO and the second leading cause of disability [19].

In this study, an immune infiltration analysis was performed on an integrated dataset (GSE117999 and GSE98918). The results showed that the Immune Score in the OA samples was significantly higher than in the normal samples. Through DEG analysis of the OA and normal samples, a total of 2313 differential genes were identified. Through WGCNA, we found that the yellow module was significantly positively correlated with the OA samples and negatively correlated with the normal samples, and it was significantly positively correlated with Immune Score. Therefore, the 142 genes of the yellow module were selected for further study. The GO functional enrichment analysis results showed that the yellow module genes were related to BPs such as regulation of inflammatory response, positive regulation of inflammatory response, blood vessel morphogenesis, endothelial cell migration, humoral immune response, and so on. The upregulation of different cytokines related to inflammation, such as matrix metalloproteinase-3 (MMP-3) and interleukin (IL)-1β, has confirmed that inflammation is a preliminary response in patients with OA [20]. Studies have shown that miR-940 regulates the inflammatory response of chondrocytes by targeting myeloid differentiation primary response 88 (MYD88) in OA [21]. MiR-149 inhibits the inflammatory response of OA chondrocytes by downregulating the activation of TAK1/NF-κB [22]. Artesunate attenuates IL-1κ-induced inflammation and apoptosis by inhibiting the NF-κB signaling pathway in chondrocyte-like ATDC5 cells, thus delaying the progression of OA in mice [23]. Some researchers believe that the growth of blood vessels (angiogenesis) and nerves (neurogenesis) from subchondral bone to articular cartilage may mediate the relationship between joint pathology and pain symptoms in OA [24]. In OA, angiogenesis of the synovium, osteophytes, and meniscus increases and may lead to the ossification of osteophytes and deep articular cartilage [25, 26]. Studies on angiogenesis in OA rodent models have shown that vascularization changes occur in the early stages of OA development [27]. Although both angiogenic and anti-angiogenic factors are upregulated in OA joints, angiogenesis seems to dominate, and articular cartilage loses its resistance to vascularization [28]. Nerve growth factor (NGF) increases FGF2 expression in human chondrocytes through the PI3K/AKT and ERK/MAPK pathways, and it promotes endothelial cell migration and tubular formation. NGF may be related to the angiogenesis of OA subchondral bone [29]. The KEGG pathway enrichment analysis results showed that the yellow module genes were closely related to the complement and coagulation cascades, osteoclast differentiation, and other pathways. The complement and coagulation cascades pathway has been confirmed in previous studies [30–32]. In OA, NF-κB promotes osteoclast differentiation by downregulating miR-1276 and upregulating MITF [33]. The ability of monocytes from patients with OA to produce osteoclasts is stronger than that of monocytes from control patients. With increased osteoclast formation, absorptive activity is enhanced, osteoclast apoptosis decreases, and IL-1 receptor type I expression decreases. This may suggest that systemic bone metabolic changes affecting osteoclasts are involved in the pathophysiological mechanism of OA [34].

The degree, MNC, closeness, and MCC algorithms of the cytoHubba plug-in of Cytoscape were used to calculate the PPI network of the 142 DEGs.

The intersections of the 4 algorithms resulted in 5 hub genes, including *TLR7*, *CSF1R*, *APOE*, *C1QA*, and *CCL5*. These 5 hub genes are widely involved in immune response, fat metabolism, inflammation, bone development, and so on. Toll-like receptor 7 (TLR7) is a member of the toll-like receptor (TLR) family, which plays a basic role in pathogen recognition and innate immune activation. It recognizes the pathogen-associated molecular patterns (PAMPs) that are expressed on infectious agents. It also mediates the production of cytokines necessary for the development of effective immunity. The related pathways include NF-KB family pathways and diseases related to the TLR signaling cascade, which induce pro-inflammatory cytokines and interferons, respectively [35–37]. Targeting extracellular miR-21-TLR7 signaling provides lasting analgesic effects for OA [38]. Colony stimulating factor 1 receptor (CSF1R), which is a receptor for a cytokine that controls the production, differentiation, and function of macrophages, plays an important role in regulating the survival, proliferation, and differentiation of hematopoietic progenitors (especially mononuclear phagocytes, such as macrophages and monocytes). It promotes the release of proinflammatory chemokines to IL-34 and colony stimulating factor 1 (CSF1) and thus plays an important role in innate immunity and inflammation. CSF1 plays an important role in the regulation of osteoclast proliferation and differentiation and bone resorption, and it is necessary for normal bone and tooth development [39–41]. Studies have shown that *CSF1R* is a DEG between those with a torn meniscus in the knee joint and those with end-stage knee OA [42]. Hypercholesterolemia is a risk factor for atherosclerosis, which is closely related to the occurrence of OA. Apolipoprotein E (APOE) has been studied to explore the effects of hypercholesterolemia on the progression of OA by constructing ApoE-deficient mice and dietary hypercholesterolemia rats [43]. Chondroitin sulfate, as a common drug for the treatment of OA, can reduce atherosclerosis in ApoE knockout mice [44]. APOE is also involved in innate and adaptive immune responses, such as controlling the survival of suppressor cells of myeloid origin [45–47]. The complement component 1q (C1q) protein can be expressed and secreted by human articular chondrocytes, bind to chondrocytes, and affect the relative expression of collagen. Primary human articular chondrocytes express genes encoding C1q and complement C1q subcomponent subunits A, B, and C (C1QA, C1QB, and C1QC, respectively), and they secrete C1q to the extracellular medium [48]. Diseases related to C1qA include immune deficiency caused by C1q deficiency and complement deficiency in classical component pathways. Related pathways include the TLR signal transduction pathway and the production of C4 and C2 activators [49, 50]. C–C motif chemokine ligand 5 (CCL5) belongs to the C–C motif chemokine family [51, 52], and its expression level is a potential predictor of OA [53, 54]. In the future, we can conduct early diagnosis of osteoarthritis based on the expression of the above five genes by sequencing the patient's genome and intervene in the early stage to improve the prognosis of OA patients. However, due to the high price of genome sequencing, which increases the medical burden of patients, there are still certain barriers in clinical application.

However, our diagnostic model still has certain limitations. First of all, our study is retrospective. It is still necessary to design a multicenter and prospective study with a large sample size to validate the model. Secondly, the samples are mainly from Americans and need to be promoted among populations in other regions. Finally, other regression modeling methods will be used to determine whether the prediction accuracy can be further improved.

Before realizing clinical translation, we need to carry out corresponding in vivo and in vitro experiments. In future studies, targeted studies on these 5 genes include the use of enzyme-linked immunosorbent assay (ELISA) and immunohistochemistry to detect the expression of 5 genes in the serum, synovial fluid and synovium of patients with OA and non-OA. And the relation between the gene expression and the severity of articular cartilage damage and synovitis need to be explored. Cell biology and molecular biology methods (such as MTT, Western blot and TUNEL) need to be performed to study the effects of these five genes on chondrocyte proliferation, apoptosis and the expression of various inflammatory indicators. Knockout mice, gene overexpression or silencing lentivirus and gene-specific antagonistic peptides were used to study the effect of blocking the above five genes on OA.

We used these 5 genes to construct a diagnostic model, and the diagnostic model performed well in training cohort and validation cohorts. In addition, we verified the methylation expression of some hub genes. The 5-gene signature can thus play an important role in the prevention and diagnosis of OA.

## Supplementary Information

**Additional file 1: Table S1.** 2313 identified DEGs

**Additional file 2: Table S2.** 142 genes contained in the yellow module

**Additional file 3: Fig. S1.** The classification accuracy and the sensitivity in the four models

## Data Availability

The data used to support the findings of this study are available from the corresponding author on reasonable request.
