# Supplementary material for: Identification and development of a novel 5-gene diagnostic model based on immune infiltration analysis of osteoarthritis
Source: J Transl Med. 2021 Dec 23;19:522. doi: 10.1186/s12967-021-03183-9 (PMC8705150; doi:10.1186/s12967-021-03183-9)
Supplement: Supplementary file 1 — Additional file 1: Table S1. 2313 identified DEGs [file 12967_2021_3183_MOESM1_ESM.doc]

"","logFC","AveExpr","t","P.Value","adj.P.Val","B"

"POSTN",-1.93104506759847,12.2841576050764,-4.63724723301834,2.97636343106968e-05,0.00100666627508625,2.30520904181592

"IL11",-1.89791672676459,8.99141744587283,-4.13383423438943,0.000150943470427214,0.00296544380383387,0.778891629242038

"CEMIP",-1.57900881623933,9.70694288327917,-6.00040300387728,2.97363202089938e-07,5.11493300210279e-05,6.66917134254626

"SPON2",-1.52634788138863,12.3343657680439,-4.78253681480127,1.84367997388022e-05,0.000720122075387405,2.75719684478659

"SOCS5",-1.52163721489874,7.53284382395255,-4.74819399666057,2.06542001822062e-05,0.000776224762730013,2.64995946998478

"VEGFA",-1.48323000153278,10.4535411225948,-4.34375657098961,7.72697941708622e-05,0.00188065217404429,1.40708530902091

"MRI1",-1.40708282338582,7.33014326856279,-5.89026362269396,4.34181634432627e-07,6.21366020669221e-05,6.30970798239233

"ADAMTS14",-1.36573972538195,8.80806537896894,-6.90955873925344,1.29646560911499e-08,6.10328244248895e-06,9.64404333884828

"COL6A3",-1.35531041085471,10.2611403806608,-5.50998859572564,1.59520331757345e-06,0.000139885255627801,5.07450320513306

"SLC17A9",-1.30001973727538,11.5232708932006,-8.13583501882641,1.98000299356281e-10,5.06003907883502e-07,13.6031686550732

"PLXNA1",-1.28428341878132,8.36672078708846,-5.94423095281569,3.60707014828695e-07,5.76132838238439e-05,6.48577005760269

"ZC3H7B",-1.28130287984057,8.03341728679379,-5.03512805985104,7.94737723201385e-06,0.000419382393225651,3.55275852850055

"HMCN1",-1.28038536256719,8.66709635408191,-4.91232610384104,1.19802596800391e-05,0.000552357900557264,3.16454607452986

"COL5A2",-1.20534365091652,12.3453991969888,-4.58225949817177,3.56398514602999e-05,0.00111656970713363,2.13534724986321

"AXDND1",-1.19869406715575,7.76354906224678,-4.01380263981758,0.000220157785112976,0.00381628160647871,0.425760312050889

"NRBP2",-1.16513485562106,10.1863351978015,-6.61521585327277,3.57420348691546e-08,1.34169113459924e-05,8.68137998275426

"TYMS",-1.15060973120088,8.26647556170079,-5.56903567247531,1.30427710197003e-06,0.000124771192925892,5.26554317020554

"COL1A1",-1.14219743361424,14.7127120139486,-3.04563168569143,0.00384967874887986,0.027183379015214,-2.21654695865203

"FCF1",-1.11916995365318,8.50411491909161,-4.66571013770003,2.71067426548394e-05,0.000941577707480431,2.39340016653475

"KIAA0895L",-1.11715808225595,8.32052071865332,-6.47064621127098,5.88407569298663e-08,1.80615467223474e-05,8.2079768886013

"TDRD12",-1.11674106611244,8.24611959821147,-3.492318333376,0.00107531044951652,0.0111773553930279,-1.04809091640996

"LOXL2",-1.10796646570665,11.7297227292651,-4.2261317414488,0.000112609470507017,0.00241253990167667,1.05350120834779

"SS18L1",-1.10569139191372,7.64648080891956,-5.86558022296777,4.72581802413635e-07,6.65670540423426e-05,6.22923192043818

"FOSL1",-1.10005051996775,8.27890125712756,-5.05958857961039,7.32153099798233e-06,0.000398099902805185,3.63038471092369

"PLAU",-1.09749374875032,11.0277536780337,-4.04946342119091,0.000196890146817953,0.00354699681412524,0.530185624710199

"TNFRSF12A",-1.09430456620059,9.33523835816687,-4.8483332439309,1.482287248353e-05,0.000629800878744852,2.96330259479639

"TK2",-1.08209826998647,6.43099996323854,-3.78775590922487,0.000442847467769555,0.00622317231023532,-0.225915373058537

"AVPR1A",-1.06449084539004,7.50166363272075,-2.86353966080904,0.00630902414164795,0.0377970974112325,-2.6633164983154

"TMEM259",-1.05976091238458,10.0379892875928,-8.05093975327947,2.63587103347124e-10,5.89413711472088e-07,13.3330287960113

"TMEM200A",-1.04660101854309,8.32431663539877,-5.71679907817799,7.87054448839064e-07,9.08362389373034e-05,5.7449360842974

"FNBP4",-1.0372326341616,9.07368379766656,-6.91301882311465,1.28111753464861e-08,6.10328244248895e-06,9.65534451313428

"COL3A1",-1.03551384508496,15.0181765109009,-3.07252985832522,0.00357396521050207,0.0258530787103403,-2.14901479769122

"C12orf75",-1.02629865926187,9.20032855865009,-4.40363497812115,6.37045778403008e-05,0.00168332524813167,1.58855319281407

"CAMK2N1",-1.02609638563331,10.8394017528603,-4.30080793875062,8.8697154891817e-05,0.00206333342504514,1.27752646680722

"SGK2",-1.02001844021908,9.98999460278815,-3.14302069447657,0.00293686129252911,0.0225915699725877,-1.97022986171206

"NARF",-1.0174006853831,9.37397644469391,-6.08304458003446,2.23769650879419e-07,4.21370029955992e-05,6.93922881534583

"CMIP",-1.01175753925231,9.37447991067774,-6.09249453739248,2.16607801771295e-07,4.12223081477308e-05,6.97012505065586

"ABHD12",-1.01152962290618,7.26816315994734,-3.84135101694771,0.000375781081096008,0.00553887811111764,-0.0730696552164005

"KDM6B",-1.00902441226153,8.58275687630866,-7.24226761537918,4.13335650057958e-09,3.36098247449401e-06,10.7283084297786

"ADAM12",-0.991511859853532,11.7060667609085,-6.11909024808985,1.97655376955148e-07,3.88555718500072e-05,7.0570937874622

"TMEM129",-0.979216328940026,7.65588833165572,-3.71670048462988,0.000549740508989119,0.007183570464066,-0.426872958539668

"RHOBTB3",-0.976361206151882,9.6515675084803,-5.23488784045854,4.05757981108893e-06,0.000279314798685643,4.18934023594799

"METTL3",-0.967230745908727,9.10457156131321,-4.26768909011805,9.86193884679656e-05,0.00222044332163299,1.17797349295456

"PIM1",-0.963210270869878,8.5512449048904,-3.74363701829861,0.000506580359149385,0.00680346549911663,-0.350921210313205

"SOX11",-0.962937520453567,8.92677167071874,-4.15750960449538,0.000140046492120574,0.00280862297931047,0.849084772453997

"C1orf53",-0.961437735130144,8.01448377010145,-3.70713757804809,0.000565897467491328,0.00732407428588126,-0.453768668705028

"ANO8",-0.957893453611462,6.06582104610121,-5.09046686888898,6.60055510935708e-06,0.000376679726866861,3.72851247894169

"SPTLC2",-0.956246024097841,7.86942281395174,-3.55945742913577,0.000881456273521405,0.00983678807050806,-0.864424095503328

"EZH2",-0.946241301246939,8.05772697953389,-5.02998253705649,8.0856032775196e-06,0.000424174067541197,3.53644128279758

"DTYMK",-0.944195694855852,8.65494207414193,-4.65044700086192,2.85011449904414e-05,0.000980494197565398,2.34608572040316

"PPP2R3A",-0.938220222876546,8.1485943360678,-5.14603000354466,5.47548861720457e-06,0.000340107694004072,3.90545151892023

"OGT",-0.933607566527396,8.9985035403817,-5.76278854717823,6.72344377644057e-07,8.25893784426122e-05,5.89448184625067

"UBE2C",-0.931223467881136,9.39491783564161,-5.48911804376059,1.7127434455463e-06,0.000148015785011487,5.00705877931818

"ETNPPL",-0.930295947431844,8.10335535437757,-3.23355034180623,0.00227485197540854,0.0189014523864763,-1.73688439137552

"MRPS27",-0.915032915316973,8.87799135766312,-4.22461324059793,0.000113155677959219,0.00241844913143664,1.04896256154149

"NT5DC2",-0.912752491494587,10.7956793530039,-5.96898328373212,3.31287867402636e-07,5.48741542589422e-05,6.56656886427404

"RASL12",-0.892164741854431,7.16923715606384,-3.51648732727447,0.0010012482831911,0.0107141686368026,-0.982198659913694

"ERN2",-0.891855206933782,8.54854572432355,-5.4830819697312,1.74831205266065e-06,0.000148931211000221,4.98756090968281

"CRIPAK",-0.887576807765929,8.17864067401246,-6.14722428619206,1.7940444335226e-07,3.6889265369294e-05,7.14911566529367

"GLIS1",-0.885303015650024,8.62129229442236,-6.12501451135478,1.9366427242884e-07,3.84940018831059e-05,7.07646921693692

"TATDN2",-0.883643090251791,7.59880640812379,-5.29738124297125,3.28455164594033e-06,0.000245846629264546,4.3896337753825

"TAF4B",-0.881848520486127,7.54601950592123,-4.20194153093614,0.000121624148465576,0.00253582096958122,0.981280318491138

"LENG8",-0.879012726842655,13.2296085536664,-5.81682093745822,5.58650642077118e-07,7.33103218908613e-05,6.0703621160665

"NXPH4",-0.877061149838489,8.70773647219701,-4.5022778832134,4.62656565884132e-05,0.00133923354483839,1.88953866166189

"DCHS1",-0.874360515807828,10.037234407936,-3.78912916568644,0.000440993275311322,0.00621664988340759,-0.222012487914596

"RHOT2",-0.868276496539499,9.77947818165351,-6.23382904886737,1.33124421754761e-07,2.9400775071246e-05,7.43250995637869

"ARL4C",-0.865789228145125,10.8012358203469,-3.73968044998657,0.000512709044623576,0.00686515875693949,-0.36209508625812

"FIBIN",-0.859513454100201,8.19523691682486,-4.31608234041246,8.44558161899256e-05,0.00199295834272416,1.32354455041427

"ARID2",-0.859173818544232,7.84928041252605,-4.52220370719296,4.33593623495918e-05,0.00129060837449559,1.9506332813605

"SNRNP70",-0.857986927802195,12.2460896278766,-6.37546052431943,8.1700505541458e-08,2.08791477661592e-05,7.89623991965365

"SYVN1",-0.856252352478953,7.33770973252637,-3.75925618315624,0.000483068105817938,0.00663208391786423,-0.306751470186642

"ARHGAP1",-0.85564944315605,11.4067060263863,-4.51741908067364,4.40404736811287e-05,0.00129792427295175,1.93595429449931

"DNMT1",-0.844352400822975,9.04693675679216,-2.91717313545346,0.00546387458333099,0.034331657104119,-2.53364705038075

"TM9SF2",-0.839371491426913,11.1478792964195,-3.03915055188697,0.00391903389998528,0.0275471895626077,-2.2327611377035

"GPX7",-0.835948385413992,8.38844710768423,-2.75628134827578,0.00837502674065008,0.0459783109685539,-2.91763669121267

"PLEKHG4",-0.829742116382945,9.71087962634834,-7.26814921141541,3.78242372500745e-09,3.25349638616993e-06,10.8124139887983

"ZNF469",-0.82706548689227,10.2680305860294,-5.78934009959293,6.13857367326139e-07,7.73330594654739e-05,5.98088608219237

"ABCB4",-0.826724570587813,7.59104770045391,-3.63027128161389,0.000713416992883507,0.0085195704844413,-0.668628125850057

"PRIMPOL",-0.825939673313512,9.55292849057016,-9.07986083402183,8.61233194774335e-12,7.70330031065904e-08,16.5533967452961

"MXRA5",-0.818092025708792,11.3055439204807,-3.39176236438579,0.00144346629259471,0.0138086462610838,-1.31944920786988

"PCDHB10",-0.817987350277519,6.69989454348841,-4.67666935158673,2.61470668509126e-05,0.000913564216593701,2.42740424377886

"TAF13",-0.80615751996611,8.26783106021525,-4.38681912866298,6.72594428105249e-05,0.00172378821266115,1.53749417361471

"MAPK8IP3",-0.803018687070159,12.0623357687819,-7.0702825105555,7.45928207159306e-09,4.60134817168028e-06,10.1684831191954

"ZRANB2",-0.800174397637431,10.6469791917964,-4.25513662824005,0.000102656128871898,0.0022615954302825,1.14032359058157

"PXDN",-0.797026771878272,12.191387325316,-3.33887167473176,0.00168249569925777,0.015301956123387,-1.46032353907493

"ACTG1",-0.79601188156219,10.6636845108509,-3.49536553000675,0.00106569231278564,0.0111301375419445,-1.03979738086289

"CA12",-0.795828011877464,7.79264991350887,-3.15738158017996,0.00282092810847543,0.0219406882315291,-1.93349085011903

"TBC1D2",-0.794657520217547,7.69523114289183,-3.10780874120133,0.00324040867765903,0.0241431365408756,-2.05986152130347

"SYN1",-0.792231480619193,9.23330058812896,-4.44583760551519,5.55722862358203e-05,0.0015154460799887,1.71702118999046

"AIPL1",-0.791116390028234,8.02003272848981,-2.83805464945186,0.00675185956479793,0.0396046671493897,-2.7243536079647

"LAX1",-0.790357917032335,10.3176042152568,-3.13801508925154,0.00297831568657501,0.0227980698832436,-1.98301066261013

"TAF1C",-0.787983760942575,11.6471849110716,-5.61089967892935,1.13056050063856e-06,0.000114912481795018,5.4011809618477

"UAP1L1",-0.786869491360187,9.42966122389217,-4.83300295263603,1.55969246115249e-05,0.000648868335757136,2.91520589474982

"RAD54L",-0.78189727739417,8.08084698558178,-5.66015273582971,9.55398179807294e-07,0.000102262646234301,5.560949027399

"PPFIBP1",-0.779479134053494,8.99934268646249,-5.04992834647534,7.56266117950568e-06,0.000407380595871594,3.59971627312436

"NPC1",-0.772965335954577,9.60271968796934,-5.09807241348946,6.43401984605626e-06,0.000372486022738189,3.75270466644723

"FAP",-0.769307014996199,11.3366091764877,-2.8930498285898,0.00583003774667203,0.0358150910886731,-2.59217247618726

"C1QTNF8",-0.766797694744581,7.78942418497634,-2.93863044430577,0.00515630937940095,0.0330850855409267,-2.4813139349702

"C4orf47",-0.765006499808338,7.55352374248365,-4.5761528499406,3.63587643256601e-05,0.0011259783226977,2.11652612040125

"TNFRSF10D",-0.762464493937058,11.6926150656986,-4.24705993771691,0.000105338174797875,0.00230930711882253,1.11612241631419

"ARHGEF1",-0.76133727225557,11.4465926254398,-5.19658915947427,4.61762979795791e-06,0.000305943627613589,4.06684655892711

"UCKL1",-0.759927755120525,11.6730563822178,-4.99698800992905,9.03006177054142e-06,0.000449968732627341,3.43191344179151

"BMP15",-0.757076651575378,13.008980873736,-2.87512493754709,0.00611677596025768,0.0370423172488319,-2.63544577658578

"PSD3",-0.756156419251667,8.55662587097502,-4.20751726126386,0.000119485974073298,0.00250408505893403,0.997911469996799

"H2AFY2",-0.755213491357093,7.2257991482076,-5.33388015615146,2.90252151214908e-06,0.000221894048422371,4.50684149693195

"COL13A1",-0.753099885188087,9.62274840910134,-2.94694825722947,0.00504148964576988,0.0325425418330813,-2.46095796461666

"MDK",-0.752845026423671,11.8337559260867,-3.74503716630376,0.000504428486447146,0.00679497077865437,-0.346965551602455

"CCNB2",-0.752453369110621,7.83917400086793,-2.99474294461583,0.00442670031697474,0.0298231243236772,-2.34324829115661

"NPIPB3",-0.750278714433245,6.71956010202744,-5.53256976445185,1.47703190985568e-06,0.000136199091935094,5.14752328494711

"GOLIM4",-0.749341159733243,6.45248441667051,-3.26611013411615,0.00207335944601539,0.0176869761746326,-1.65196038826902

"HOXB2",-0.747913186779785,9.07759180166812,-5.58001391493647,1.25631144992082e-06,0.000124166605125047,5.30109735778123

"GJA1",-0.740733620942754,11.063527319623,-3.44664990255758,0.00122977245587897,0.0123245935368173,-1.17189433259887

"WDR41",-0.737565714367227,8.66225928919852,-5.5115555567297,1.5867085455332e-06,0.000139885255627801,5.07956865682089

"PHOX2A",-0.725943277247835,7.0594803921093,-5.01429469282647,8.52184067589147e-06,0.000434322529490093,3.48671929489756

"ULK3",-0.724027872214535,8.38222269835048,-5.27138736021582,3.58657985986283e-06,0.000257671996438097,4.30626142350812

"DSEL",-0.716553183552222,10.0906827472344,-3.91304362998298,0.000301228244380472,0.00472690531905461,0.133035291617912

"CLIP3",-0.714413237134869,8.97257876904785,-3.04554857241258,0.00385056081528046,0.027183379015214,-2.21675502978747

"CACNA1A",-0.714399315922767,11.4310605263949,-5.09633867625236,6.47161370693614e-06,0.000372808426525671,3.74718911006788

"COL12A1",-0.714311318194207,11.2922131168693,-2.76244612847039,0.00824109107457279,0.045571832529531,-2.90320340990744

"LOX",-0.714190373151926,10.5068105245667,-3.66337790904763,0.000645839765702235,0.00797337996455989,-0.576379346679042

"RBM4",-0.712711842305104,9.008775963652,-6.14194541571052,1.8269584396578e-07,3.71391585534528e-05,7.13184764947682

"SLC25A37",-0.708298354976632,10.0985072261582,-4.0267600896525,0.000211410567442807,0.00371375834657058,0.463654823473783

"MSH2",-0.702435879448855,8.38233114244553,-4.26627741386225,9.90655300313649e-05,0.00222078103600387,1.17373704173029

"COL27A1",-0.701822331060812,11.7947193408567,-6.24605230379483,1.27633279791957e-07,2.85403967774789e-05,7.47252040903686

"PLOD1",-0.700221954706569,11.4324199412208,-2.99116730626956,0.00447014839058496,0.0300174491588492,-2.35209793959438

"PLEKHA4",-0.694668717833499,9.23812268344664,-2.83492593278987,0.0068081711284925,0.0398619674853864,-2.73182101790596

"DYNLT1",-0.69432228886658,10.835749660053,-4.22139120707698,0.000114323208821896,0.00242889297222672,1.03933447943537

"PHF11",-0.69392911152163,8.42273314684556,-4.86456552136437,1.40444897085947e-05,0.000606864435741669,3.01427768273369

"HJURP",-0.693165354649047,8.87604782263844,-5.54930324417563,1.39510633791402e-06,0.000129984673327833,5.20166502145348

"GPATCH8",-0.69300853228635,8.07194552945087,-3.52733383922679,0.00096961974977701,0.0104997141063928,-0.952545017200448

"LRRC17",-0.691532299970302,9.89166913525001,-3.43453170404502,0.00127418449874122,0.0126562390327494,-1.20458941210632

"PDPN",-0.690675914651552,11.7950304935249,-3.38745885782517,0.00146163843252064,0.0139377664815361,-1.33096017293972

"HHIPL2",-0.687625708688142,7.53090132705443,-3.0399786354413,0.00391010764703136,0.0275060620124829,-2.23069073437678

"INTS8",-0.685456107670226,8.18226694627843,-3.00798985918643,0.00426915652945807,0.0291492141814792,-2.31040157706604

"SPATS1",-0.685271697853037,7.96836350516421,-3.19266925073337,0.00255412307743823,0.0204249922808639,-1.84276922059964

"FUT4",-0.680845914354313,7.43478917166235,-4.00014154852305,0.000229759750671831,0.00392819290024368,0.385868788883648

"FAM193B",-0.68052171775218,11.2366894286366,-5.36642934691789,2.59915484081265e-06,0.000203931056786393,4.61150050501205

"GALNT2",-0.68042291513872,10.9984394941424,-5.29233880933108,3.34110287342886e-06,0.000247989987465596,4.37345409527725

"GCGR",-0.6778290737189,8.39435936761399,-3.71940850535065,0.000545246408475704,0.00714572381041895,-0.419250101794489

"WSB1",-0.676357939095745,10.860503982201,-6.95489070450812,1.1092075293312e-08,5.53051018176855e-06,9.79206887419206

"MAST2",-0.675891681373567,10.7498657464415,-5.51767450432863,1.55396436825837e-06,0.000139008887640401,5.09935141080219

"SH3BGRL2",-0.67580430206076,8.60094477801318,-4.49546207429863,4.73029024719702e-05,0.0013571771751134,1.86866302503834

"PPP1R37",-0.674353569452435,8.57801985653968,-4.22701122327785,0.000112294294584441,0.00241156378850069,1.05613020209328

"HERC4",-0.673456519105882,8.47461677837406,-5.22567021518831,4.1858996328077e-06,0.000286902523108417,4.15984064116073

"SH3PXD2A",-0.673275037372672,10.1441643646873,-4.80774646824862,1.69597914553599e-05,0.000683319165191292,2.83606572033756

"PTK2",-0.672856620342463,9.69326159512716,-5.08996283257229,6.61174097133402e-06,0.000376679726866861,3.72690952181931

"COTL1",-0.672654298520733,12.8427937329575,-2.90186442222951,0.00569367081393439,0.0353655887339278,-2.5708256514091

"IGDCC4",-0.672585460509984,8.34272739837937,-4.56997106631075,3.71009974087813e-05,0.00114628625672831,2.0974822801901

"ZBTB49",-0.6681731689257,5.61396253267539,-3.75422662340705,0.000490522188341232,0.00670355342034858,-0.320984935507191

"UPF1",-0.667319429066685,9.46252585824337,-4.30506569025195,8.74944284813052e-05,0.0020433261502638,1.29034747871951

"DPP4",-0.666139175190626,9.58478889988784,-3.65423531643542,0.000663860841833422,0.00813799438044817,-0.601898961587509

"CCDC62",-0.665587640103405,10.9849722320707,-2.77630624212857,0.00794716478639191,0.0443993850292832,-2.87067059707985

"ARF4",-0.665077995202456,11.7509722013848,-3.45566326101725,0.00119770097493553,0.0120844177894088,-1.14753338013799

"TNS3",-0.664315404112434,9.17321057489868,-4.2140614596483,0.000117023073715182,0.00247449854100578,1.01744316171984

"MEGF6",-0.662807042287437,11.0550799036202,-4.78003113220182,1.85903404245687e-05,0.000721393925932991,2.74936466476855

"TM2D3",-0.661718088749218,8.62839528144096,-4.37455666942791,6.99731939665745e-05,0.00177050985412737,1.50030824080575

"CHTF18",-0.66135430029224,9.90624431140607,-6.77435209321428,2.06525075760528e-08,9.0110416592197e-06,9.20212412510486

"NPHP3",-0.659417620676033,8.89614116863439,-3.7597446232919,0.000482350050561049,0.00662731186980539,-0.305368684414521

"PLOD2",-0.659048000166941,12.8004010585528,-3.39999408959076,0.00140930418065478,0.0135844037242898,-1.29740734432235

"IBTK",-0.657652126584598,11.5182976551829,-3.91383240138011,0.00030049350233056,0.00471951559542703,0.135313135055767

"HNRNPH3",-0.655175008165431,8.39871827355982,-4.4702327599514,5.13441962283826e-05,0.00144190946048593,1.79149028238169

"ANTXR1",-0.652240792604955,10.4680208949592,-2.95272609334068,0.00496314196210819,0.032145418740099,-2.44679532724919

"WEE1",-0.650582432038215,8.24123794554702,-2.88846388023045,0.00590218194289248,0.0360971394107363,-2.60326107171897

"TMEM128",-0.64946005635678,8.79088545291407,-5.38408650175582,2.44795526226744e-06,0.00019462876305201,4.6683270276542

"MMP2",-0.649325659477077,11.8518823655155,-2.99925339439003,0.00437245696801529,0.0296212949835779,-2.33207503522667

"SCHIP1",-0.648444800127326,8.81070786433555,-5.9464028538471,3.58024674162185e-07,5.76132838238439e-05,6.49285863389741

"GSDMA",-0.647313039466489,10.7614185677375,-3.0970571336793,0.00333882653309805,0.0246607216559005,-2.08710114695797

"TUBA1A",-0.64637805341531,13.0395284355667,-3.52126346444857,0.00098720133419279,0.0106385811249246,-0.969147298438247

"C1QTNF6",-0.642995311374957,11.3526487948033,-3.33115399554958,0.00172037213910844,0.0155235337006793,-1.48076997025859

"INSIG1",-0.642367143224789,9.76046855066979,-3.3466119722128,0.00164530486049482,0.0150860372370025,-1.43978895707331

"CCDC18",-0.642294102596079,9.27008530238509,-4.62998789590654,3.04811754508951e-05,0.00101490314436997,2.2827450750584

"MTHFD2",-0.641723084000814,7.50187799970497,-6.07373714120585,2.3105425610779e-07,4.3055516536586e-05,6.90880143541909

"ZFP1",-0.641590724713003,8.86653039027012,-3.68644953652126,0.000602427928648543,0.00759686314768161,-0.511830022817582

"MRPL57",-0.640850144758948,13.2365156048955,-2.96007865751572,0.0048650850629162,0.0317633236096744,-2.42874589728947

"SPAG5",-0.635862872103386,9.82229951421796,-5.51960927067663,1.54375110048632e-06,0.000139008887640401,5.10560732438493

"COPA",-0.635731312598921,8.45936028926571,-4.08960894207407,0.000173548076089164,0.00327835431167799,0.648241656775961

"RP2",-0.634884848011895,9.20504893398049,-3.56061692975039,0.000878422078502391,0.00983678807050806,-0.861235122936619

"PRRC2C",-0.632206059391695,10.0901101789133,-4.62900839989621,3.05792830686687e-05,0.00101490314436997,2.27971493560987

"NPRL3",-0.631786435537657,8.96910142206335,-3.50329831466172,0.00104103735095229,0.0109676779571175,-1.01818767970583

"CTHRC1",-0.630129224321445,9.24532224476828,-4.28142375879349,9.43797121459767e-05,0.00215777467757536,1.21922112086971

"NUP107",-0.62852262895084,9.31036190149015,-4.31769991996634,8.40184144477776e-05,0.00198810240219087,1.32842174105394

"RAB32",-0.628465508488821,7.3767420915593,-4.00010371852713,0.000229786895534552,0.00392819290024368,0.385758409254777

"UBD",-0.62836983484608,12.7345163967807,-3.10664436852171,0.00325093377729974,0.0242114714163676,-2.06281442280314

"CENPT",-0.628081518180331,9.51997942655831,-5.36026908507333,2.6540537371321e-06,0.000206371567353834,4.59168320224154

"RUNX1",-0.62732035885066,11.0011167671678,-4.50551089342129,4.57814684145702e-05,0.00132736578357901,1.89944480537698

"FLYWCH2",-0.626918737834211,8.87262123669297,-6.47611537484718,5.7741420928404e-08,1.80615467223474e-05,8.22588861375678

"MAFB",-0.626071483570886,13.4541780102544,-2.91977472477191,0.00542569288196849,0.034205234399933,-2.52731570464781

"LPIN3",-0.62601454569163,11.9209644251586,-4.2264935112813,0.000112479720514883,0.00241253990167667,1.05458260234094

"P3H4",-0.625422568288414,11.3684371065407,-2.74803455633395,0.00855734153143289,0.0465889358251577,-2.93690888050651

"GOLGA6C",-0.624783112685529,10.1132290449134,-7.37752002068918,2.60078447982621e-09,2.58474630886728e-06,11.1673754504853

"FGFR2",-0.624378885287072,11.4919399498953,-2.86858712724836,0.00622458508932654,0.0374576878101488,-2.65118327088205

"DVL2",-0.62280370049829,8.55921816351088,-4.08325292361754,0.000177055784304276,0.00332526114385158,0.62951582016384

"CRISPLD2",-0.622705175941729,11.0585110707672,-2.93737201200181,0.0051738919816726,0.0331503415688185,-2.48439030028811

"DAPK3",-0.622034592544145,12.1949481447744,-2.93571254531663,0.00519716332193281,0.0332161681550754,-2.48844568121954

"UNK",-0.621831375255356,8.11523494291855,-3.2614837656005,0.00210092144977364,0.017845861260684,-1.66405877244223

"CEP192",-0.621747277382995,7.73592845641095,-3.46249210235514,0.00117393545201922,0.0118786005353857,-1.12905246394846

"RHBDL1",-0.617239500536183,13.6357243717366,-3.50207231764368,0.00104481179189727,0.0109815735283491,-1.0215292180678

"WIZ",-0.616654495736267,9.04555760061655,-3.75872990447896,0.000483842939696711,0.00663762756766447,-0.308241275122121

"VMP1",-0.615562746414398,11.5179503347882,-2.72903430519034,0.00899142108754188,0.048312653743778,-2.98115546941447

"CHST1",-0.614817227541481,9.27175364910148,-5.2368472085786,4.03080887444761e-06,0.000279314798685643,4.19561235473272

"C9orf92",-0.614092266538588,7.96820131340277,-3.14748717370059,0.00290033140999268,0.0224053564693002,-1.95881473470982

"DMPK",-0.613698962797827,12.6259331669587,-4.97164635666119,9.82860867923811e-06,0.000476109494578781,3.35175335134588

"PACS1",-0.613064598479811,9.04771201830404,-4.94888833336829,1.0604849120819e-05,0.000503209936133502,3.27985913319492

"REC8",-0.611711108042493,7.11324399165615,-3.936940966633,0.000279720914306333,0.0044938478297282,0.202144194949804

"ZFP3",-0.611569666357453,7.65824778309932,-3.57066631737826,0.000852540373692266,0.00963429863864873,-0.833572401342667

"MFAP2",-0.611066491266861,10.9065109393468,-7.1193916738425,6.30127528015425e-09,4.17494494395109e-06,10.3284944182215

"ST6GAL2",-0.609617626485266,9.37180790556244,-5.03596732470173,7.9250540455656e-06,0.000419382393225651,3.55542037256303

"GRIN3B",-0.604846197596533,11.2644396491848,-4.56339447297178,3.7906939747957e-05,0.00116514990575808,2.07723200668597

"ITGB1",-0.604842515854754,13.7792174025965,-3.87086788714159,0.000343147848605184,0.00519337721125055,0.0115617380609629

"S100A2",-0.604244998263038,9.50198157012803,-3.24773736359414,0.00218487877844731,0.0183814721372422,-1.69994512929789

"MAP2K7",-0.604216703728852,9.14346418980518,-3.88851886374386,0.000324959821553092,0.00498987660752211,0.0623221023999232

"SLC2A5",-0.602785232050859,10.7539465931314,-3.88733641469388,0.000326148555776124,0.00499954714162732,0.0589181314640408

"NUDT17",-0.601962894391161,9.2398067288133,-4.65448869704823,2.81251974036415e-05,0.00096942515675095,2.35860965218983

"ZNF518B",-0.60036777681595,7.41195844440908,-3.71716236622814,0.000548971480239558,0.00718296967943351,-0.425573003415023

"PPP2CB",-0.599564821483826,10.4201430119535,-2.96848830872557,0.00475515081760269,0.0312738577117995,-2.40806476213168

"PITPNM3",-0.598965313694826,8.54444621440473,-3.38357060098467,0.00147824418213306,0.0140288117634898,-1.34135308399189

"ENO2",-0.59807999003951,10.8383483667814,-4.22198922840435,0.000114105627739124,0.00242715288302639,1.04112125538104

"KIAA1522",-0.597948457649057,8.30011014457828,-5.39796566427667,2.33525368226247e-06,0.000187333422071719,4.71301955870013

"EIF4G2",-0.597240659313584,9.03459968740548,-3.77572340834207,0.000459418500695217,0.00639077570679373,-0.260081927548711

"NPIPA1",-0.59650312780453,10.8826027037083,-4.45941324321678,5.31790693929843e-05,0.00147950291193017,1.75844382500417

"RGS3",-0.595447576053424,9.83713282490659,-4.1968610240311,0.000123604815989094,0.00256813769248421,0.96613440216879

"NEK9",-0.590833413098014,10.343393057726,-3.66909440742367,0.000634811646821433,0.00786981673595884,-0.560405784772247

"SH2B1",-0.589361703343716,9.82526142361541,-4.25770559517776,0.00010181708232935,0.002257540822882,1.14802526112939

"TDP1",-0.587425666349936,7.53978518731116,-5.14776723080884,5.44355353789826e-06,0.000339302192471993,3.91099103246891

"RAC3",-0.586288462980362,8.11012912834011,-2.84373930351349,0.00665065054714561,0.0392004901607538,-2.71077131194541

"TNFSF11",-0.585878113017621,10.5929763787744,-3.70658548141938,0.000566844071607799,0.00732407428588126,-0.455320341277008

"ZFYVE27",-0.585018930993215,9.52439183710465,-4.13249054699387,0.000151586043342398,0.00296774593345644,0.774913057770833

"BAG3",-0.585016684776638,9.16627119597332,-3.94942787830152,0.000269081700108429,0.00440402793526047,0.23833470743899

"FUS",-0.583370602915657,7.12725055511803,-2.80921076420578,0.00728778400662882,0.0418124336416238,-2.79297994496344

"AMPH",-0.583316827293123,7.73430668754855,-4.0241578930074,0.000213139750482267,0.00373145682456261,0.456040123494965

"STX1A",-0.582578361576722,9.8495570073682,-4.76164059824317,1.97563948458123e-05,0.000747946496068906,2.69191876200748

"NOL4",-0.582517901562671,9.14619385089934,-3.61555977781447,0.000745577708229632,0.00878432404154727,-0.709476449641818

"NPEPL1",-0.581944498610493,10.507732033027,-5.48482477399989,1.73796770282097e-06,0.000148931211000221,4.99319018209159

"TRPC5OS",-0.580790907612897,7.13580936306834,-4.34965886344258,7.58162661012137e-05,0.00186301811028106,1.42492989947006

"NOL12",-0.579608129819929,9.635384451591,-5.89699233576885,4.24263700327488e-07,6.21366020669221e-05,6.33165150758343

"TOR4A",-0.579491212330534,8.09255048364419,-3.23045114612407,0.00229496809098444,0.0190084124958056,-1.74494065555823

"ADAMTS12",-0.579225770515553,10.5471564053495,-3.28762114814835,0.00194964514519255,0.0169553728742584,-1.59557039260375

"PPL",-0.578258168591315,12.400564443833,-2.97515820654707,0.00466961522522975,0.0309273405272621,-2.39163427627946

"ZNF862",-0.578083185798196,7.39529524919746,-3.32330709239289,0.0017597122438473,0.0157621858881267,-1.50152983852899

"WDR90",-0.577127217578617,10.3841898310294,-6.82734660278671,1.72064732360387e-08,7.89247691588453e-06,9.37540354528774

"PLXNA3",-0.575085878871031,9.40771874748504,-6.06774285126931,2.35870478147111e-07,4.31863476440766e-05,6.88920678411716

"H2AFZ",-0.573677942124828,10.5461983037476,-4.310181957172,8.60702230314692e-05,0.00201532751284025,1.30576038761138

"BNIP3",-0.572906973289122,11.2232258229827,-2.84607401166651,0.00660949320453891,0.0390479603487439,-2.70518758482333

"PRC1",-0.571957894123527,9.39086688751542,-4.88802913376398,1.29899965781104e-05,0.000584016334028942,3.08804862603301

"DLG1",-0.57194735238045,9.74113616129066,-4.35399979342395,7.47643185399794e-05,0.00184957355323818,1.43806003015083

"GSDMB",-0.571909350054069,9.53414392990517,-6.10670820644389,2.06263902958116e-07,4.01071191306275e-05,7.01660143685804

"ACAA2",-0.571878760977452,10.176139008286,-3.21955913483983,0.00236700846896948,0.0193384178385661,-1.77321633481295

"AFAP1",-0.571446159436496,7.35175790930034,-3.54767824292749,0.000912854282289975,0.0100989797500837,-0.896787902151615

"TRIO",-0.570557804641362,10.288913501783,-4.72671787706538,2.21719541062949e-05,0.000809130015056374,2.58302167773218

"LOXL1",-0.570242946589039,11.6195840463573,-3.13026252492203,0.00304360910972026,0.0231471079390463,-2.00277978116377

"SMARCD1",-0.569782661678667,8.46848392863126,-4.78084875755639,1.85401010913826e-05,0.000721008409616833,2.75192023311951

"UBR1",-0.569672483846627,8.72524256966568,-3.96918864721323,0.000253040792253857,0.00420467315823001,0.295717224896501

"PAGR1",-0.569454959005895,8.77653383614914,-3.78147475771149,0.000451424594732341,0.00631394415572076,-0.243757705986982

"USP22",-0.569295355084749,9.48231833203979,-4.73340582365126,2.16879171979547e-05,0.000801601551145066,2.60385674459653

"PTPN1",-0.56901861162539,9.84611638882395,-4.52767297272534,4.25934218153953e-05,0.00127630439339298,1.9674195014782

"FBXW8",-0.568487209561975,7.57368596551943,-3.50235546722925,0.00104393890800356,0.0109806461246481,-1.02075753295088

"PFKFB3",-0.567979906131546,11.7042012827028,-3.7142703308114,0.000553803793995866,0.00720633272729569,-0.433711188801756

"MICAL1",-0.567912055095967,11.1919033115163,-4.46359227378865,5.24628697433229e-05,0.00146641918255985,1.77120449476958

"GAL3ST2",-0.566922180398205,15.1125978010094,-2.9973559122656,0.00439520010575337,0.0296700885629517,-2.33677681861957

"PDLIM7",-0.564470647018009,11.5209019003027,-4.35319211692564,7.49589594777619e-05,0.00184957355323818,1.43561664079061

"DLGAP4",-0.563392175758425,9.49252776764864,-4.026906909837,0.000211313410614818,0.00371375834657058,0.464084524301148

"PNMA3",-0.562585056240719,13.4220072681171,-4.16357314484018,0.000137381104875398,0.00277696111312541,0.867089864786154

"SFXN3",-0.562376747966452,12.889698667032,-3.27310686331898,0.00203232576048227,0.0174775187774289,-1.63364347659636

"SUSD5",-0.561457007987626,9.65066329953411,-2.81422330064805,0.00719190568904339,0.0414351049504983,-2.78108887708041

"MARK3",-0.561084952487123,10.1776119025121,-4.77217617265517,1.90798641527152e-05,0.000732445686326013,2.72482004770736

"NFATC4",-0.558411992718447,8.96006979763803,-2.92434392153148,0.00535923556931523,0.0339247576431282,-2.51618668064547

"KAT6A",-0.556657192019101,11.6157319090569,-4.01593715972185,0.000218693074148561,0.0037945687715263,0.431998937835748

"NCAPD2",-0.556569719771641,9.92848554762848,-3.97952145709492,0.0002450258224959,0.00410418252680633,0.325775688736534

"FAM184B",-0.555910700888801,7.91233313567336,-4.45408372050697,5.41063299891353e-05,0.00149599402963778,1.74217654366989

"C16orf58",-0.554904869359499,10.5815823610747,-3.93263486429893,0.000283483366293236,0.00452041518718828,0.189676532432864

"CACNB4",-0.554196141462802,7.87590243501053,-3.47629950915776,0.00112725106859436,0.0115581204033759,-1.09162201499558

"CCDC106",-0.553602977003408,14.3308083635022,-2.88515918526487,0.00595468428684609,0.0363189046053153,-2.61124423220698

"DBN1",-0.553302927200722,9.49049754803575,-3.92951154911715,0.000286242948872859,0.00455569405016599,0.180637510882906

"NLK",-0.552823083636662,9.11735596548229,-5.60007122747111,1.17315710434676e-06,0.000117902288986849,5.36608255659241

"GOLT1B",-0.552813813584067,7.59127704953966,-3.44750496360913,0.00122669514329086,0.0123075431398374,-1.16958487060364

"PKN3",-0.551963884328527,9.2982768179126,-3.04890041952134,0.0038151382435983,0.026983516606991,-2.20836086857809

"HOXD10",-0.551055198014168,11.3920572469781,-3.91176724237671,0.000302420883652794,0.00474146116359757,0.129349758439223

"ZNF426",-0.550708225550508,8.49869529337932,-3.79386942688978,0.000434650226919002,0.00615634038745371,-0.208534873240599

"ING5",-0.550533996453872,10.0349672484643,-6.68564181529484,2.80375461627501e-08,1.16642712396613e-05,8.91190004090284

"TGM7",-0.549781772498317,14.9143760220015,-2.7957761112153,0.00755063104384929,0.0428954762086662,-2.82477766458608

"ARFGAP3",-0.549664276337813,10.2320307741056,-4.64734616002234,2.87929162704749e-05,0.000988630478239014,2.33647964717631

"ENOSF1",-0.549359061223649,11.0260980465079,-4.48112764713235,4.95591817073426e-05,0.00140279145816875,1.82479667079458

"AGA",-0.549169407519297,7.95798354424618,-3.36167628524438,0.00157515176029497,0.0146088149849033,-1.39974349061364

"ABCC5",-0.547206471859344,10.5976351294795,-3.79641090120461,0.000431285737246483,0.0061242283388385,-0.20130540307917

"GSKIP",-0.546800140774828,7.89625141198752,-3.08139573292984,0.00348722899515023,0.0254624650996908,-2.12667145746982

"ATG16L2",-0.546405460086865,10.6735674605749,-6.38831446432263,7.81585336403878e-08,2.08791477661592e-05,7.93833576365993

"MAGED1",-0.546164689557044,10.3335889907731,-5.28213982484708,3.4584479560197e-06,0.000252347692409945,4.34073833077012

"GOLM1",-0.546003340377091,11.2377980215594,-3.6924599689349,0.000591587536158615,0.00751561611683742,-0.494979167153269

"SRGAP2C",-0.545853150750659,8.7127484251245,-3.30248417165781,0.0018682892433171,0.0164477491504427,-1.55647711189458

"NCAPG2",-0.545801922893469,8.66947598065022,-4.59986196312613,3.36446701774563e-05,0.00108055566392193,2.18964765323482

"CARS",-0.545483519426883,10.0768902276221,-7.02033937329155,8.85629449861092e-09,5.05954410191713e-06,10.0056374060509

"WWC3",-0.545375922745469,9.92944807882149,-3.42432175184695,0.00131278591808676,0.0129035314772825,-1.2320845628716

"CCDC159",-0.545310846968265,9.05133600053344,-5.06643651982315,7.15521182153331e-06,0.000391807105034952,3.65213386458448

"TMEM255B",-0.5447797768713,8.94013875596171,-5.20272189088449,4.52306562219816e-06,0.000304184665095875,4.08644790157206

"TLE3",-0.544409226456557,10.5799384228107,-3.46449155071098,0.0011670626436427,0.0118286592816568,-1.12363741410583

"MZF1",-0.541919065777202,10.8205794127348,-6.49818989229135,5.35091448075554e-08,1.77263905826363e-05,8.298182452444

"PSMA1",-0.541676231312969,9.38867708995144,-4.64018663884222,2.94778298986393e-05,0.00100443599820335,2.31430836091766

"ADAMTS4",-0.54166832267885,10.00082774998,-6.67842489670362,2.87437633519398e-08,1.16862996046102e-05,8.88828154943367

"ARMC7",-0.541509184272025,10.954572280171,-5.19702609326806,4.61082825725627e-06,0.000305943627613589,4.06824290816257

"SMC4",-0.541447173242011,9.07849658315479,-5.15923649857308,5.23728741208617e-06,0.000333415781191493,3.9475741653559

"GPR162",-0.540988421914403,11.9380074139036,-2.73978850486035,0.00874329833453462,0.0473249210004508,-2.95613849043299

"PTBP2",-0.540644383510745,9.00399974902281,-3.13647576034729,0.0029911742351814,0.0228476156674466,-1.98693843390643

"UBASH3B",-0.540631601219559,7.14400719605173,-2.98247389626624,0.00457745351276977,0.0304862494005727,-2.37358478762082

"PIK3R2",-0.54060546702925,12.6871075793907,-5.11004386795089,6.18025647618202e-06,0.000365932793378984,3.79080203460331

"MGAT4B",-0.540249546631419,9.31594508182212,-4.23805373922922,0.000108409423278731,0.00235444586545926,1.08915841946641

"RFX8",-0.54009832478463,10.4140417114074,-7.53611966009816,1.51267023207793e-09,1.85198949788474e-06,11.6806891627987

"FAM160B2",-0.539735397888375,9.98600166885583,-6.39043963164728,7.75878977797357e-08,2.08791477661592e-05,7.94529562996107

"STRA6",-0.539470314650828,9.54356062992508,-3.69688504685012,0.000583726589809013,0.0074641064796951,-0.482563821665863

"OSGEPL1",-0.539340954358916,6.7901101758031,-3.05678638227272,0.00373300131962728,0.0265851123530249,-2.18858807123109

"NCSTN",-0.539054755478988,13.906048975074,-3.82719879469742,0.000392470979867407,0.00572201577738227,-0.113533785106871

"SOGA1",-0.538903837378228,11.2262833449445,-8.25060411420194,1.34622722297216e-10,4.01377646529151e-07,13.967205630015

"PERP",-0.538772720290572,9.59423959541779,-3.62059462977324,0.000734417621849323,0.00869490194391962,-0.695506601466482

"AEBP2",-0.536111115373862,7.90615020991942,-3.22506890776159,0.00233030333113816,0.019166343122175,-1.75892031420057

"TPM2",-0.535136782347077,12.5915302055983,-3.63068071118853,0.000712541261004238,0.00851663001638282,-0.667490019224406

"SFT2D3",-0.534511279483233,8.77157527408647,-3.72017699663725,0.000543977509262123,0.00713955514540727,-0.417086337194448

"RNASEH2C",-0.53438889327418,10.7038168129502,-4.75271149986779,2.03481892789084e-05,0.000766334227390298,2.66405194083888

"SLC2A1",-0.533759571781502,11.2064313518699,-3.31287759418769,0.00181332358805895,0.0160727392156424,-1.52907694832443

"PRR14",-0.53232705020605,11.2175762982956,-4.7228731187758,2.2455005744735e-05,0.000811539588198213,2.57104826237631

"RBM6",-0.532016924466564,9.74763643678208,-7.33563539668894,3.00169176968819e-09,2.82617179305011e-06,11.0315285858383

"GPR68",-0.531433058011283,9.28431008670203,-3.82825436829885,0.000391201834503327,0.00571282417749388,-0.110518238497468

"EGLN3",-0.531369922582991,9.59530185954402,-5.74499080303561,7.14618859498066e-07,8.57974280373215e-05,5.83659073593445

"SYT7",-0.530390532187731,9.41990284771872,-4.08289409512751,0.000177255851284145,0.00332526114385158,0.628459041133583

"DZIP1L",-0.52948572256657,7.00566810915049,-4.5224178062697,4.33291268359357e-05,0.00129060837449559,1.9512902561149

"TUBE1",-0.529348025234385,8.10367582164583,-4.7204715464929,2.2633608265898e-05,0.000814673276194467,2.56357081182229

"PDGFRB",-0.528871633948592,12.4381955656582,-3.12664404101809,0.00307454401973965,0.023315763158934,-2.01199632519653

"SPDYE1",-0.528287968556644,9.98913751038256,-4.39552255042755,6.53960011972166e-05,0.00169792317186794,1.56391158845382

"ARMC6",-0.527737386195451,12.199800041975,-3.92251175858491,0.000292522276318118,0.00461865048636788,0.160392221593892

"FAM114A1",-0.527377441164329,9.99432892433916,-3.3440178556252,0.00165768125018383,0.0151684193782806,-1.44667414863143

"AMPD3",-0.525780536147655,6.56968831144858,-4.31841833690621,8.38248604463902e-05,0.00198614957420593,1.33058808512471

"AHI1",-0.525709852861178,9.33751103505893,-4.06314933401729,0.000188609527014724,0.00345700392291639,0.5703731217838

"CDC16",-0.52570544831641,9.7899962451877,-3.0430951934582,0.00387668366783364,0.0273238515471622,-2.22289531045459

"DCANP1",-0.525345050843514,12.2101756769457,-2.84938737185039,0.00655148967242567,0.0387940418642433,-2.69725787882389

"SHC1",-0.525024497050632,11.0345848254271,-3.25352638952353,0.0021491417543859,0.0181606031385023,-1.68484358443755

"PRPF38A",-0.524371573511813,7.98402670725277,-3.84330880834076,0.000373526876705811,0.00551694947732364,-0.0674661077165224

"WTIP",-0.524105593814594,8.39554155270904,-2.79042695152004,0.00765770938325204,0.0432823896230634,-2.83740866645075

"MDGA1",-0.522561103402669,9.57627531208894,-4.20760668009258,0.000119451983782235,0.00250408505893403,0.998178261859417

"FAM76B",-0.522275290021885,7.56205047784944,-3.62177144941285,0.000731832320814035,0.00868153076063811,-0.69223985371891

"GALNT7",-0.521731967605742,8.98497028659579,-3.23799183394076,0.00224631401301793,0.0187252149948172,-1.72533058999915

"IER3IP1",-0.520939131547079,9.6622778293834,-4.19223850461852,0.00012543418897849,0.00259409503657365,0.952360557358714

"SLIT2",-0.520869599376624,10.4399957917366,-2.87141199985203,0.0061777876509408,0.0373107506035381,-2.64438638451209

"PURA",-0.520085300528708,10.1172495975785,-4.74264757051217,2.10361009116705e-05,0.00078658284067182,2.6326629757072

"NDRG1",-0.519771177418034,10.7759212118465,-3.10150053014682,0.0032978165338467,0.0244689506320961,-2.07585095468001

"CLEC18B",-0.51894696747874,10.7190438646026,-3.4162765024134,0.00134398561988978,0.0131667901173101,-1.25371691756251

"LIMA1",-0.518733563105414,9.20167668116813,-3.92775096910525,0.000287809931923805,0.00457656166416439,0.175543816684251

"BMP1",-0.518717974963536,11.0324510334689,-6.60807470117255,3.66329693691367e-08,1.34169113459924e-05,8.65800090096396

"MITD1",-0.51844121583547,8.61209592811634,-3.95244146795436,0.000266573311161784,0.00436696883092779,0.247077036302123

"HOXA3",-0.517483149742645,9.1719167069567,-4.08155941564323,0.000178001938314593,0.0033343211251411,0.624528664862428

"SLC5A1",-0.517145786777638,9.82392193058785,-3.1213842049646,0.00312003954550993,0.0235503744428807,-2.02538142430452

"ARMC9",-0.516794517181454,9.40772980905623,-5.07968197781312,6.84402862052914e-06,0.000385008893058635,3.69422245707419

"MGRN1",-0.516657080622657,9.0925703053008,-3.99335281956724,0.000234681084335861,0.00398690400539812,0.36606850046933

"SART1",-0.516460140622728,13.760778124368,-3.47392916544157,0.001135137409812,0.0116169754714685,-1.09805378763506

"PHLDA2",-0.515412572653938,8.63218804185372,-2.89286938390482,0.00583286083847173,0.0358201330379062,-2.59260900870073

"BIVM",-0.514279365280908,8.08087754848769,-3.00469659914531,0.00430782485465094,0.0293310791252759,-2.31857639000139

"ARFGAP1",-0.514252286555406,10.8762003131095,-6.60714611266236,3.67504419449733e-08,1.34169113459924e-05,8.65496078591079

"SQLE",-0.514144085158417,10.2617482135496,-4.78878268415075,1.8059479949943e-05,0.000713170059215299,2.77672543159132

"IL17RD",-0.513961972219937,8.68288048092849,-2.89239184001242,0.00584033824755011,0.0358537443069402,-2.59376419544865

"XAF1",-0.513154990323413,11.072354836083,-5.1048252463788,6.28963851938062e-06,0.00036896228703311,3.7741918817889

"LIF",-0.511528161804399,10.9480703245805,-4.07072613847309,0.000184171610185711,0.00339304421690235,0.592647962548377

"CDKN2D",-0.511463783013275,9.45295914377998,-2.89621913116805,0.00578066035329081,0.0356300025647161,-2.58450226843635

"KCNRG",-0.511339590203136,9.11413904330117,-4.12178108090684,0.000156803408220184,0.00304566359354058,0.743223192676842

"EVC",-0.511094848720113,9.63535319047077,-4.04804975468804,0.000197765313716574,0.00355560170560382,0.526038003740633

"FAM162A",-0.511020812163288,11.0453316125358,-2.91592823171213,0.0054822336461262,0.0344111149808953,-2.53667535567164

"SLC35G1",-0.509748733028456,11.915893321102,-3.06538327894718,0.00364535246157171,0.0262451479088186,-2.1669949690482

"LYSMD1",-0.509111741100243,8.40198396390544,-5.11804684069058,6.01615477945312e-06,0.000358743309498789,3.81628235406933

"ATP6V1A",-0.508675958711896,9.4086413542073,-3.16375715501436,0.00277085462470681,0.0216988007629514,-1.91714659908409

"MTERF1",-0.508669157244056,7.34419117458924,-3.24426976473574,0.00220655373579949,0.0185232471983656,-1.70898299849345

"FOSL2",-0.508374209681994,9.8678241257615,-5.62873024940721,1.0637415156623e-06,0.00011193689396284,5.45899706983733

"ZBTB21",-0.507982657864371,8.97922070285574,-4.12709466150634,0.000154193325386613,0.00301403089776134,0.758941866595669

"XAB2",-0.507749540080066,13.0620987549223,-3.07632366083034,0.00353660462708355,0.0256725401099127,-2.139458928571

"RCN2",-0.507081980781443,9.1980645230606,-3.51240584604142,0.00101340343452662,0.0107909369287183,-0.993343941981865

"SPSB3",-0.506752953394559,10.2375541434021,-4.46508844493625,5.22087580477053e-05,0.00146160011379562,1.77577412571471

"SLC39A4",-0.506452848471799,13.4359246836907,-3.14140831885763,0.00295015459314878,0.0226503500072269,-1.97434814447559

"CCDC17",-0.505456078583659,8.88237387581382,-3.54513137447785,0.000919782444909704,0.0101442590363685,-0.903777736958105

"SYNDIG1",-0.504377774551651,9.17173301161832,-3.76896359319428,0.000468988517140863,0.0064856436680034,-0.27925238607737

"ATP1A3",-0.50341967789454,8.40426911304526,-3.68584547343412,0.000603527912713788,0.00759785222463542,-0.513522778019086

"ARSJ",-0.503409161276644,7.08006679807761,-2.84729102159647,0.00658813308671378,0.0389346259624126,-2.7022757116725

"ARMCX2",-0.50306075360238,9.7878603568141,-2.87345600011317,0.00614413057740515,0.0371451003376819,-2.6394654898483

"EGFR",-0.503021144285309,9.19614648608525,-5.07577371654101,6.93443104443232e-06,0.00038765636548078,3.6818007753501

"OSBPL10",-0.502772960729272,9.12664151005802,-3.80280477981887,0.000422931981821723,0.00604056363093719,-0.183106632967722

"ABCG5",-0.50124240195278,8.12212563714341,-2.77781762121013,0.00791570672269547,0.0442512742382185,-2.86711613329892

"HOXD11",-0.500185560971479,10.078684122001,-3.64062569846544,0.000691583950127818,0.00837136286476309,-0.639824459531391

"SUN1",-0.500019143718962,9.0865739374784,-4.15261800604239,0.000142233311928903,0.00284019075986168,0.83456787891918

"MBD1",-0.49994814495893,8.37057093732754,-4.18917617018106,0.000126660598249344,0.00261341573481259,0.943239176490733

"SERPINB9",-0.499700049001989,9.08512354185685,-3.92745871011034,0.000288070855388179,0.00457664256841841,0.174698360881804

"GTF2IRD2",-0.499338233476909,12.5740553006174,-3.95724138795831,0.000262624725304886,0.00432207333116752,0.261007944276566

"VAV2",-0.499138378708217,10.4464775732262,-5.44851236890401,1.96659348089309e-06,0.000162121616496297,4.87596484392328

"SMC5",-0.499116612871444,6.83887134469897,-3.07625608104433,0.00353726691649541,0.0256725401099127,-2.13962921582924

"BAG2",-0.497743405969263,8.41770464404715,-3.39074312752727,0.00144775062107358,0.0138348348613169,-1.32217622128799

"DLG5",-0.497689263896341,9.14148120000131,-3.75828308160813,0.00048450173115326,0.00664157200659055,-0.309506070651729

"RBBP8NL",-0.49660442228722,13.0751529893133,-2.86864423646047,0.00622363574998523,0.0374576878101488,-2.65104590656928

"SEC31B",-0.495831244578167,10.0701612846929,-5.12009223853864,5.97490910568611e-06,0.00035867499661617,3.82279614685982

"ARHGAP11B",-0.495554946061178,9.40146541628175,-4.26746961261149,9.86886226178393e-05,0.00222044332163299,1.17731480171082

"SIN3B",-0.495251493299332,10.4933243078859,-5.57512020772743,1.27747254501881e-06,0.000124290076671545,5.2852472445907

"ERF",-0.49505404418321,11.4584441502794,-3.2707185548012,0.00204624517750439,0.0175629281891682,-1.63989856690132

"ZNF793",-0.495044359425625,9.40724642384886,-6.25028528603179,1.25784866926906e-07,2.84831073981699e-05,7.48637687092976

"TMEM246",-0.494978705447348,9.75534433559453,-4.07774187130554,0.000180152821376386,0.00335354195796272,0.613289889124323

"ZNF766",-0.494711613564239,8.1535079746328,-3.69854601813634,0.000580801952327579,0.00743202154877544,-0.477901662438907

"TRMU",-0.494495002962516,9.31757484542752,-4.10038991183004,0.000167752097792759,0.0032026865287243,0.680033635394522

"TEP1",-0.494378516398006,8.94120464968493,-3.15512264887511,0.00283887351295349,0.0220228136484063,-1.93927680510451

"YTHDC2",-0.493639579151825,8.61031560581014,-4.49954812073158,4.66783772818563e-05,0.00134877698925702,1.88117650037139

"SUPT20H",-0.493510228035284,10.1253774288711,-3.51033778047708,0.00101961610654843,0.0108254137305584,-0.998988432930473

"TPGS2",-0.493336792698171,10.8387781323836,-2.86207542547633,0.00633371786841233,0.037898151436105,-2.66683352289138

"CHD3",-0.49328886581441,9.52136364326382,-4.48754277415893,4.85366914626541e-05,0.00138260011715831,1.84442199976089

"HSPB6",-0.492905367684598,9.26675889501036,-4.29206679666571,9.12170993013993e-05,0.00210552605084223,1.25122097990113

"KLHL17",-0.492817605477729,13.156187937032,-3.50409606671814,0.00103858833689028,0.0109547799284376,-1.01601299931802

"PBK",-0.492815769347667,7.66112126934545,-3.76269419656029,0.000478035685675598,0.00658149986017986,-0.297016423508619

"NRGN",-0.492564724773114,10.9034467655448,-3.45485529190231,0.00120054302383808,0.012099444593487,-1.14971861169008

"FNDC1",-0.492459915990304,13.8093647959826,-2.75256616758243,0.00845671083415218,0.0462494956013905,-2.92632386350975

"CA9",-0.49227915882174,10.6770055071957,-3.26170655743289,0.00209958623137201,0.0178429919681776,-1.6634763905008

"APLN",-0.491465303108459,9.46038667882333,-4.51600676722019,4.42435176056668e-05,0.0013012659346451,1.93162245331649

"TCF3",-0.490269906224428,9.81843620180996,-4.37057296754453,7.08776108689558e-05,0.00178158570199983,1.4882363122596

"OXSM",-0.490003686710038,9.443398996795,-4.76494252598029,1.95418661719287e-05,0.000742623173279656,2.70222785427684

"PCNT",-0.489944584648127,9.30888541964299,-4.47095590801855,5.1223791401533e-05,0.00144078994399689,1.79370007194292

"BUB1",-0.489503935594476,8.76940685625129,-6.42514689570645,6.88364517744006e-08,1.9861536867617e-05,8.0589631626295

"FKBP10",-0.488665517013539,9.63992822972151,-5.48986352668686,1.70840066479613e-06,0.000148015785011487,5.00946710902138

"ASH1L",-0.488515865088001,8.14537693299939,-2.80602211670763,0.00734939057264916,0.0420714393453187,-2.80053658709545

"AKR7A2",-0.48841069552868,9.08049858365607,-4.00994714029339,0.000222827573208152,0.00385136469287018,0.414495590054559

"SBSN",-0.487153758628265,8.05114085005775,-4.79452858165023,1.77190773572602e-05,0.000704392388542283,2.79469763563969

"ZNF146",-0.486713454824827,8.81325961029899,-4.40123656136367,6.42001401122042e-05,0.00168893574480474,1.58126616544008

"RCOR3",-0.486330659488745,8.86670261807267,-3.93389458405793,0.000282377649890522,0.00451425717505946,0.193323187757477

"OSTC",-0.484578454412988,10.2899253098795,-4.39904099006593,6.46570921013443e-05,0.00169750741323706,1.5745967641579

"CDX2",-0.484427718285527,11.8583930431459,-3.07353552478214,0.00356402565681632,0.0258124918926264,-2.14648245634827

"KIF5C",-0.484234539010316,9.64919028970243,-4.4789481870968,4.99113347217293e-05,0.00141052743576148,1.81813154182316

"PCNA",-0.484089981481493,9.67135694029593,-3.28717932082513,0.00195211425362363,0.0169603554555964,-1.59673087439195

"ZNF185",-0.483520324167333,14.1891178730705,-2.88962780761988,0.00588379324060511,0.0360216212461276,-2.60044788697744

"KIAA0355",-0.482743834426644,9.36327034583582,-2.95785356344816,0.00489456717744821,0.0318743764970408,-2.43421133045838

"NKAIN4",-0.482514176015295,9.85880550573586,-3.37696300710474,0.00150687605082757,0.0141951056731197,-1.35899846519012

"DTX3",-0.482310050939359,8.35098584375063,-3.99103393364794,0.000236385394068893,0.00400627894080626,0.35930871147765

"CGB7",-0.481959232820056,13.4235388513383,-3.16433947917421,0.00276632310682264,0.0216893001452725,-1.91565273545076

"KHNYN",-0.480249569084721,10.714739358572,-3.3093672638747,0.00183171404461057,0.0161814975526116,-1.53833700621565

"MRPL12",-0.47889951613549,9.25956611747307,-2.75575461982684,0.00838656317795245,0.0459783109685539,-2.91886883859546

"HECTD3",-0.478580246046409,10.8543687579718,-3.42642775398877,0.00130473349115962,0.0128848903398385,-1.22641701487486

"EML3",-0.478006859637379,11.1734856045582,-4.09641098011009,0.000169868987827226,0.00322588781660429,0.668295856732303

"PCDHB16",-0.477655201387665,6.59113562445718,-2.72389684465381,0.00911223170608758,0.0488342459527264,-2.99308182154027

"ICMT",-0.47764659915333,9.96370888236487,-3.40939122440175,0.00137124674449413,0.0133379110183611,-1.27220689665032

"POU3F1",-0.477204823896558,11.0554760628265,-2.78006934759684,0.00786905257263514,0.0441560481404862,-2.86181800245241

"TIGD7",-0.476820530083467,9.84948846987508,-5.07069584394673,7.05365101882953e-06,0.000389589702976946,3.66566525089672

"FADS2",-0.476150315997998,9.8518032624622,-3.38158109399174,0.00148681013832483,0.0140876835617017,-1.34666812938093

"KCTD10",-0.474974319640735,9.29901603521659,-4.59575894012516,3.40997104689737e-05,0.0010845467200988,2.17698418809222

"CXCR5",-0.474121477079231,11.2781365268521,-3.23778118235137,0.00224765982694675,0.0187277068673733,-1.72587878505748

"CCDC66",-0.474086471717545,9.23375031448498,-3.99088751722073,0.000236493407205065,0.00400627894080626,0.35888195377786

"UBTD1",-0.472790246975439,10.5902928690449,-2.73865412053425,0.00876916971829179,0.0474059242049675,-2.95878064663223

"RPH3AL",-0.472537977479279,9.91989126089705,-4.72622259131265,2.2208219430526e-05,0.000809130015056374,2.58147907668122

"MAP4K5",-0.472369121079456,9.43664470846884,-3.80751140246329,0.000416882593482455,0.00598524294928382,-0.169700447899582

"FAM156A",-0.47207781598596,12.3645217332982,-4.07357951897798,0.000182526730499793,0.00336968078628566,0.601041357449184

"DMTF1",-0.471984675116607,10.7613698587004,-3.7821095177228,0.000450550588314549,0.0063115892516515,-0.241955277362397

"MZT1",-0.471182519583792,9.17028224435766,-5.90823491608116,4.08193307858224e-07,6.08514173689648e-05,6.36832083864516

"ANKRD10",-0.471078302314417,9.69506720730355,-4.34805521995614,7.62085247439378e-05,0.00186752643718398,1.42008061225525

"ABCB6",-0.470936037360627,10.6921514659309,-3.84469093159893,0.000371943363922545,0.005503469675112,-0.0635093761911643

"ATG2B",-0.470671590773955,9.74159581385764,-4.66691040882012,2.69999738162417e-05,0.000939693641242699,2.39712307306358

"ANKS6",-0.470329034518215,9.08600831181815,-3.63060894846581,0.000712694680223885,0.00851663001638282,-0.667689505710946

"SLC35D1",-0.470315453420918,10.243952943744,-3.68887939604587,0.000598022647461364,0.00756579005688567,-0.505019399823002

"RRAD",-0.470074375317292,10.2906416293578,-4.5810628442031,3.57796223699919e-05,0.00111898892408529,2.13165839256269

"DAZAP1",-0.470013536674095,9.29140825161988,-5.91971152933652,3.92413568854085e-07,5.99990284891514e-05,6.40576018610018

"EXOC1",-0.469844854552361,13.851040263568,-5.00949926400151,8.65976481498148e-06,0.000437611674506225,3.47152842813439

"KRTAP2-3",-0.469720091921729,10.917604177821,-2.82864208397755,0.00692258891724174,0.0402987937326839,-2.74680167807784

"MUC1",-0.469636546058746,11.2996640320706,-5.15416840898993,5.32746284195315e-06,0.000336281587536502,3.93140630989057

"LTBP1",-0.469417621839874,11.0044560450259,-3.22241832323934,0.00234789335033988,0.0192490669771907,-1.76579957864002

"CCDC86",-0.468618263005192,10.5275289817335,-3.50513363300344,0.00103541135488002,0.0109341639477265,-1.0131841668864

"SOX8",-0.468187313247252,12.1020064562808,-2.96426569963935,0.00481005687789527,0.0315075457666307,-2.41845395295714

"FHL3",-0.467720233463282,12.4928980993798,-6.12547665440707,1.93356333657731e-07,3.84940018831059e-05,7.07798070876872

"FRMD8",-0.4677151125256,10.1045867366834,-6.48335709274034,5.63173285584553e-08,1.79903694746823e-05,8.24960538083845

"ABCA5",-0.466771127476505,10.4104215834604,-5.06602908287479,7.16500221657279e-06,0.000391807105034952,3.65083963288286

"RFX1",-0.466661925965047,9.83771111166107,-3.87713241725654,0.000336582891405313,0.0051374840822096,0.0295642994702847

"CDK6",-0.465451020482538,9.68378289697903,-3.6928582997653,0.000590875772544882,0.00751561611683742,-0.493861897869676

"JADE3",-0.465320779547058,10.4309904685108,-2.88713021371499,0.00592331809211205,0.0362016526647737,-2.60648356286305

"AARS",-0.465226581331123,9.7804797937198,-3.13723396139407,0.0029848341953053,0.0228284304915846,-1.9850039510338

"C5orf15",-0.4626380770371,10.3123217747111,-2.83485859692688,0.00680938783721274,0.0398619674853864,-2.73198166804581

"PCBP1",-0.462307692629654,15.4321798046356,-2.96011598369981,0.00486459191545927,0.0317633236096744,-2.42865419067894

"GAS6",-0.461564958994922,12.1598537433027,-2.87580302925276,0.00610569413961057,0.0370002582870913,-2.63381210542872

"PRSS21",-0.460052293847237,10.8056009839199,-2.77349762816848,0.00800593101222596,0.0446023356828745,-2.8772722928354

"HSF4",-0.459997656985017,10.3019636024286,-6.38248557644991,7.97452885405012e-08,2.08791477661592e-05,7.91924641893436

"TXN2",-0.459577861949072,14.0375846797548,-4.60238947190312,3.33673298466465e-05,0.00107551020473272,2.19745041424178

"CDKN1A",-0.459566444260284,11.5838172571733,-5.38041303999793,2.47867595863012e-06,0.000196199266477585,4.65650168799423

"HIST1H3F",-0.459424616857342,10.9149553252004,-2.95578789002932,0.00492208632872828,0.0320069801289059,-2.43928272583068

"SPECC1",-0.459135168791642,9.95225120596434,-4.50743734818927,4.5495317759443e-05,0.00132424665110751,1.90534880140307

"DUT",-0.45877405162506,11.6760049658754,-3.35557115551922,0.00160323302639134,0.0147760101025836,-1.41598557071122

"SKOR1",-0.458749530836984,8.95978761888632,-4.63624220654307,2.98619775071247e-05,0.00100666627508625,2.30209829173024

"IKBIP",-0.458672603583082,8.38467562634547,-4.23320916083395,0.000110097403176513,0.002381538628083,1.07466385737383

"R3HDM2",-0.458556897969945,10.8476353031374,-4.95774900534577,1.02956917915821e-05,0.000489839442711734,3.30784000113611

"NSUN2",-0.458423497476055,11.1610822507404,-3.8722349350889,0.000341704773273631,0.00518526591653901,0.0154890524129128

"LRRC40",-0.458241756522987,7.8558210627248,-3.11473820176675,0.00317843162507421,0.0238027173003345,-2.04227347811543

"PTPRH",-0.456816816966205,12.5203010124604,-2.97070671905187,0.00472654039693724,0.0311773898085584,-2.4026027002785

"UGGT2",-0.456373917547628,9.96275882214445,-3.03043803886425,0.00401411527681156,0.0279955197609676,-2.25452213476923

"TBC1D17",-0.45537607052974,10.7746492413695,-6.96126564504642,1.08514502326666e-08,5.53051018176855e-06,9.81287900874389

"TESK1",-0.455179031336579,11.3491189548739,-4.18284627104582,0.000129232710196602,0.00265424104788405,0.924394087904727

"ELP5",-0.454913667436445,10.4557438127309,-4.67467572570138,2.63191266877904e-05,0.00091778334759821,2.4212165093955

"PTTG1",-0.453715317807747,10.3039440361853,-4.38203132507877,6.83065492667821e-05,0.00174064937298214,1.52297037530382

"SEC61G",-0.453240548907601,10.9933144683349,-3.10828094299487,0.00323614945567791,0.0241315037985086,-2.05866379469228

"ANAPC4",-0.452655942196666,9.3370036180917,-2.97948250129187,0.00461493101343895,0.0306565006428295,-2.38096880353507

"GPER1",-0.45260266814488,11.905671216437,-4.13239758208646,0.000151630598869651,0.00296774593345644,0.774637815519284

"PYROXD1",-0.451599916089807,8.90195315149944,-3.56119979438088,0.000876900610698872,0.00983503136350604,-0.859631855453613

"RNF144A",-0.451467852752248,8.39052227924432,-2.88805570667844,0.00590864327198983,0.0361243060466938,-2.60424743465861

"TAF9",-0.451328716996002,8.86152074126265,-3.64696080645182,0.000678543676600505,0.00825184760755026,-0.622179995027274

"TJAP1",-0.45089045619819,11.7453691876912,-4.10707360495369,0.000164253749713416,0.00314934118823504,0.699761736793681

"CCDC60",-0.450796948188316,8.37255262474301,-2.85708837279374,0.00641849815862599,0.0382553026098226,-2.67880292579003

"RBM3",-0.450505175061895,11.6687319130122,-3.98815009458847,0.000238521663142159,0.00403180021295423,0.350904568336246

"OSBPL7",-0.449772641247145,9.21634542907264,-3.41426377357145,0.00135190081410739,0.0132146875681676,-1.25912420980444

"ZNF814",-0.44971959672948,9.71857311586185,-5.2811415076712,3.47015106114632e-06,0.000252347692409945,4.33753668780302

"UBE2G2",-0.449485840290674,11.8562919779246,-3.3930931873894,0.00143789038707816,0.0137774082134125,-1.31588781169217

"AUTS2",-0.449464616346784,10.9143105513895,-3.32194398177391,0.00176663263681121,0.0157858597601976,-1.5051331217234

"KIF11",-0.449339986401275,10.4803514870852,-4.76133562152089,1.97763258226056e-05,0.000747946496068906,2.69096669251855

"TOP3B",-0.449309279291905,9.13312015327678,-3.37332537897007,0.00152286293304784,0.0143005223145894,-1.3687040046668

"UGGT1",-0.448949697847518,7.3877195089058,-3.29854677616893,0.00188952212366477,0.0165775680579887,-1.56684374243838

"SNAPC4",-0.448074299967072,10.1594796670266,-7.55080928863111,1.43872862880904e-09,1.85198949788474e-06,11.7281411597155

"TMEM50B",-0.448010542199787,10.6162812871128,-3.02035631382011,0.00412683757625684,0.0285148696028037,-2.27965193408479

"LUC7L3",-0.447791539098645,10.3715611834358,-3.70015890736122,0.000577975463659394,0.00741707537259892,-0.473373416636156

"PORCN",-0.447617655728548,9.51718603698144,-4.07580046008919,0.000181256310217255,0.00336358312601293,0.607576213295038

"TTC3",-0.446996436928883,9.65519793872142,-4.17743041164859,0.000131473697189212,0.00269099882038651,0.908279859632287

"SLC19A2",-0.446966218270243,7.86235404679335,-2.72165925557918,0.00916531648582416,0.0490306060451281,-2.99827127155593

"FADD",-0.446838320656175,9.91661841975467,-4.08417790093066,0.000176541068210033,0.00332086558276476,0.632240144124453

"PXYLP1",-0.446804920488756,12.9405425634161,-4.10727266816787,0.000164150651450475,0.00314934118823504,0.700349524291717

"ANGPTL6",-0.446773124530134,10.8007426370369,-3.25091007535143,0.00216522378934011,0.0182533875435934,-1.69167067034166

"RAD23B",-0.446566361228058,11.283623824368,-5.01792772372694,8.41879405930033e-06,0.00043248777369563,3.49823045880871

"HGSNAT",-0.446536389819871,11.270207487303,-4.63540156252145,2.99444804908644e-05,0.00100691129981405,2.29949651018759

"KATNAL1",-0.446324077678534,9.71795453901256,-4.17839249222025,0.000131072862237291,0.00268586762034696,0.911141763297329

"PCDHGC3",-0.444811185719544,10.0775718888866,-3.08769565634182,0.00342680252962445,0.0251547273091719,-2.11076949238182

"UACA",-0.444303111364975,7.32508402015879,-3.06409596279522,0.00365835298501674,0.0262499547456312,-2.17023086414501

"AGPAT5",-0.444093165096993,7.36811770537417,-3.01306015933518,0.00421025786835496,0.0289014977003077,-2.29780412032771

"CTDSPL",-0.44405050524079,10.6487164520874,-3.57220432156428,0.000848644333105431,0.00961456521527742,-0.829334997399726

"EP400",-0.443616959174896,8.3318425409224,-2.79567555311869,0.0075526311420771,0.0428954762086662,-2.82501526886143

"NUP62",-0.443387590645319,10.8906509266121,-3.39323183551068,0.00143731065755686,0.0137774082134125,-1.31551673056206

"FOXL1",-0.443183411457268,9.8599105971305,-3.16275101794031,0.00277870066725477,0.0217389719189694,-1.91972728274846

"RAB22A",-0.443082547911533,9.0786086605814,-3.78833097776535,0.000442070088467691,0.0062171319281435,-0.22428107735491

"HEMK1",-0.442428773270477,7.20597983421021,-2.80148669019984,0.0074378493682918,0.0424420055340899,-2.81127460414096

"CRLS1",-0.442222498608576,8.93664573517186,-2.87214376195646,0.00616571859675267,0.0372630202626042,-2.64262495540073

"ITPR3",-0.442219413187518,9.58188268836662,-4.58885730794929,3.4878776811449e-05,0.00110001546614552,2.15569190903663

"BTBD19",-0.442040781947734,10.3453438108807,-5.60691098669463,1.14606935582124e-06,0.000115830704555289,5.38825119288405

"NOL9",-0.441895094725696,8.16202916090166,-3.09377549052637,0.0033694216523139,0.02483542807509,-2.09540326539193

"C10orf88",-0.441822200874471,7.38895987313339,-2.92818768406958,0.00530391822075782,0.0336350997769113,-2.50681548372505

"IL21",-0.441724839040434,14.1916785549031,-2.9044498908372,0.00565423926366895,0.0352265182282209,-2.564555907934

"SAR1B",-0.44162636192568,13.5422993644219,-3.11736217418176,0.00315525543886615,0.0237047399109816,-2.03560690559251

"CERCAM",-0.441572770385854,14.3969471693317,-4.79360080492653,1.7773610942226e-05,0.00070499362781703,2.79179526253598

"PTPRM",-0.441563542748618,10.1335123895724,-3.15629826496247,0.00282952079045283,0.0219788525490277,-1.93626594487435

"OR2A42",-0.440975965895266,16.5905286139924,-3.37946528972459,0.00149597169279634,0.0141476441673707,-1.35231857308955

"SCAI",-0.440813081128627,7.6265500254561,-4.29872873379551,8.92903328150178e-05,0.00207174418122938,1.27126738135237

"MINK1",-0.440207925363195,10.5080003942661,-4.63604898716505,2.98809207932695e-05,0.00100666627508625,2.30150026647231

"SENP5",-0.440152790180239,9.85175226222541,-3.22155941694846,0.00235362017848125,0.0192783476981919,-1.76802801506226

"AKAP17A",-0.438606019980174,10.6738668747978,-5.65689827777223,9.66090179081938e-07,0.000102262646234301,5.5503860726756

"MAZ",-0.43775466485568,10.6429074458843,-3.28067371171728,0.00198881517925207,0.0171960494811531,-1.61380726222445

"GPR17",-0.437640018298971,11.5363600953749,-3.51521308100176,0.00100502810037191,0.0107401121192074,-0.985679015803769

"DDX39B",-0.437245793797219,13.7337637650847,-3.25247287464036,0.00215560388024172,0.0181980169012006,-1.68759306038188

"LCAT",-0.436691767969332,10.9674505273858,-4.98561261932525,9.3802722318319e-06,0.000462269118333997,3.39591767716558

"GKAP1",-0.436007691217448,11.498433784469,-3.84308877390359,0.000373779572168162,0.00551694947732364,-0.0680959557675154

"SLC23A3",-0.43558151088949,10.9833748023359,-3.04525678135362,0.00385365904356635,0.027183795989889,-2.21748548903627

"MIIP",-0.435522798208337,12.3835644469079,-3.34573623215618,0.00164947311748307,0.0151010361303248,-1.44211366226396

"ANKRD36B",-0.435431863876346,10.840978448386,-5.15234637302579,5.36025554956615e-06,0.000336281587536502,3.92559468679768

"BRAT1",-0.435317871370655,8.59930128301304,-4.11845966847875,0.000158456672853311,0.0030679430681546,0.733402265921779

"CASP8",-0.435053225113388,7.84094663675566,-3.48484989615892,0.00109923565446149,0.01133585470075,-1.06840044922105

"MARVELD1",-0.434971215233233,14.6161130227454,-3.59472668710827,0.000793508488702316,0.00919370035906459,-0.767168778570357

"RAD18",-0.434470446059033,9.86943123048698,-5.72540910808491,7.64189901409694e-07,8.93502820020785e-05,5.77292231988775

"GMFB",-0.434454369046994,9.63016622861784,-3.01344596803277,0.00420580746740477,0.0288820306274103,-2.29684498480571

"ZNF655",-0.43347254333612,7.4108695618019,-3.20486098112752,0.00246761763916565,0.0198753768334238,-1.81127915474649

"TRAPPC4",-0.433466279466314,9.92294302495142,-3.57110055407089,0.000851438642146836,0.00962793038518632,-0.832376123449921

"SCFD1",-0.433435064636408,12.27330917557,-4.579925047944,3.59130169417754e-05,0.0011192473171976,2.12815128134626

"KIAA1109",-0.433191501512409,8.43734083752931,-4.78451425393171,1.83165093284418e-05,0.000716989136491235,2.76337874440226

"NDN",-0.432541473618235,11.5700869953876,-3.72120952900939,0.000542277109072557,0.0071224634392063,-0.414178773528681

"LRRC59",-0.432167602958339,11.1456899797312,-3.13879810075325,0.00297179492339593,0.0227734559812577,-1.98101225201555

"CD276",-0.432141908780864,13.1696784624935,-2.9888881162573,0.00449805107302175,0.0301298085371706,-2.35773524822711

"LGR4",-0.43180865355736,8.76457495858792,-4.14328353961543,0.000146498857345241,0.00290545239362419,0.806886279595505

"TAF6",-0.431429723662095,9.26989011063339,-5.89908539668293,4.21224833797257e-07,6.21366020669221e-05,6.33847783600597

"CPSF1",-0.430665476666768,11.829216412096,-3.20732462091121,0.00245047668893099,0.0198052123904235,-1.80490675928322

"STAT2",-0.430412438662866,11.7446741504561,-3.31522894984556,0.00180110310019783,0.0160218465238384,-1.52287091107309

"KIAA0319L",-0.430129282807663,9.62431709994133,-3.99696271519411,0.000232051592974501,0.00395726496350891,0.376595334274637

"ITGA11",-0.429621719434722,11.3137674115459,-2.83020108701196,0.00689403697738633,0.0402110947142041,-2.74308716859586

"ABCG4",-0.429612177789899,9.00483233326273,-2.81290540305775,0.00721700010023732,0.041512834338632,-2.78421670837129

"SAP30",-0.428719432859918,9.54314093738635,-3.62281380398389,0.000729549712604111,0.00867746995264291,-0.689345892112192

"VOPP1",-0.428602395720739,10.0140472686535,-3.19074578151042,0.00256802978305247,0.0204812682964893,-1.84773053407154

"LIMD1",-0.428202994673665,9.81775417499116,-3.59745254896163,0.000787073156612253,0.00913689273110746,-0.759630424079089

"CHIC1",-0.428129144397156,9.90501683189199,-2.89750737471668,0.00576070120901388,0.0355846629585806,-2.5813828861148

"ASL",-0.427990163466589,10.1663694461256,-3.09829734619069,0.00332733208079388,0.0246143038165536,-2.08396210514716

"BCKDK",-0.427647752247815,12.2699740900203,-4.26484150075192,9.95213494700215e-05,0.00222415212248918,1.16942844294647

"GK5",-0.427501398598788,7.00637960853956,-4.08522280540104,0.000175961364695611,0.00331693662069524,0.635318018404334

"TGFB2",-0.427381623793149,9.39780378031005,-3.93578264316941,0.000280728220371787,0.00449995262923916,0.198789808639176

"TUBB3",-0.426900380331638,10.0040734867908,-4.12330613285615,0.000156049926260064,0.00303432296833292,0.747733707042948

"FAM177B",-0.426834189044779,12.6590083183471,-2.73054810340811,0.00895610598379371,0.048170709544223,-2.97763821760724

"VPS26B",-0.426694730676621,15.0041003897706,-2.71057939134216,0.00943240215587898,0.0499368902676902,-3.02392306263053

"PTS",-0.426623394629132,9.51986311233526,-3.02721611058412,0.00404982146992312,0.0281895938814999,-2.26255910561733

"SUCO",-0.426585488067797,9.58073560908562,-4.00700466221834,0.000224886217259595,0.00387847492114508,0.405901820136839

"KRTAP4-12",-0.426293969791638,11.0083952139254,-2.8586724523247,0.00639145505687966,0.0381503969010745,-2.67500253666837

"DDB2",-0.426260053458197,11.8982666310403,-4.82842831935764,1.58355174081536e-05,0.000657265825787609,2.90086231632544

"FANCC",-0.426228813577516,7.95168025910864,-3.31084253222357,0.00182396372250254,0.0161369372066508,-1.53444604687196

"MLXIP",-0.425809690486629,12.9562030257125,-5.01043860823817,8.63257545093671e-06,0.000437491076406884,3.47450376603384

"BAG4",-0.424720349934122,8.05923637260656,-3.96260201655095,0.000258281807793288,0.0042726692158705,0.276575611950538

"KRBOX1",-0.424522086904609,9.24161974117682,-4.30011483723567,8.88944619680938e-05,0.00206523770148991,1.27543986995065

"LAMC1",-0.424345763081018,15.8458205124613,-2.86676474413797,0.00625494977694147,0.0375611938770413,-2.65556565027185

"TYK2",-0.424153552185825,10.4800476267896,-4.90156172740617,1.24176922651462e-05,0.000566683920742859,3.13064191036906

"PPIE",-0.42411646745378,9.60050146867533,-3.91911768999737,0.000295614772988765,0.00465925345726521,0.150581862575523

"KNTC1",-0.424101112441439,7.90865907843925,-3.89740745282735,0.000316157070042588,0.00488409494597282,0.0879261306052745

"EGFL8",-0.424079556453535,11.3879509845144,-4.48132065512884,4.95281133096533e-05,0.00140279145816875,1.82538697650465

"CDKN3",-0.423976940248783,8.48888860954987,-3.31806939768946,0.00178644494598731,0.015931063628498,-1.51537047490115

"PTGES3",-0.423581526068101,6.81151284862859,-2.80980773039242,0.00727630358865965,0.0417599598644634,-2.79156455603473

"ZNF700",-0.423240826437295,7.70014125136264,-2.99173009369761,0.00446328352061211,0.029982605670383,-2.35070551329354

"GIPC1",-0.423031977026508,12.2069158800489,-3.07829749234864,0.00351731218436655,0.0255985344451315,-2.13448421156997

"HSBP1L1",-0.422998363682629,8.64988468204455,-4.41736083648194,6.09396410002252e-05,0.00162952053490737,1.63028501177103

"SPC25",-0.422887360016256,9.549117696262,-3.59358616447388,0.000796216021949221,0.00921313610391308,-0.770321965554683

"KDELR1",-0.422876831396305,11.1758444903114,-2.98949386794539,0.00449061937822699,0.0301109191188882,-2.35623727013161

"ATG4B",-0.422431688560112,10.6275050930363,-5.164379966055,5.14731349354456e-06,0.000328858182450066,3.96398625834014

"HPS4",-0.42236936693253,9.80311729217982,-4.15657078630586,0.00014046364524318,0.0028138344342164,0.846298052301434

"SPIN1",-0.422025203181626,9.72254806323442,-2.77361011355666,0.00800356971133292,0.0446023356828745,-2.87700798419095

"ACSF2",-0.421582806862498,8.49924863117706,-2.97586039059762,0.00466069452292593,0.0308911316489892,-2.38990310423587

"NREP",-0.421390676307409,12.3996583128485,-2.8133861335843,0.00720783700327483,0.0415032118135708,-2.78307588490358

"GYS1",-0.420663089088741,10.2011967006518,-3.03694165040611,0.00394293814623913,0.027656382907438,-2.2382821094224

"CCNL2",-0.419509632953025,9.82712828876612,-3.81944721709352,0.000401913384938535,0.00583116670167515,-0.135665699346419

"LPCAT1",-0.419481710719179,9.86000186057197,-3.42681111954321,0.0013032727434682,0.0128807989546423,-1.2253851079133

"NPEPPS",-0.419401601695803,10.0606870360164,-4.0462336514504,0.000198895159695419,0.00357232481103549,0.520710616853945

"SCP2",-0.418868771813193,10.0927097890888,-4.02409992471141,0.000213178426670188,0.00373145682456261,0.455870518896897

"EIF1AD",-0.418044441460387,12.8308205545188,-4.97355903275291,9.76598253328815e-06,0.000474738210701065,3.35779968500795

"WDHD1",-0.41721108433793,8.88406730869894,-6.37954587948461,8.05577119759225e-08,2.08791477661592e-05,7.90961910797805

"SRSF6",-0.416887105768458,12.5804946018507,-2.86493488225056,0.00628557760606638,0.037681869234223,-2.65996409428132

"MRPL3",-0.416732197469821,9.5219337297036,-2.9280531647167,0.00530584512259373,0.0336350997769113,-2.50714358559167

"UBAP2L",-0.416514683237695,9.73853876587951,-3.59788281651233,0.00078606194492104,0.00913107930694317,-0.758440238197228

"DPP9",-0.416419664170313,8.7866952126131,-2.80789597326337,0.00731312833450443,0.0419287348698315,-2.79609653675717

"SRSF5",-0.416263391513127,12.5334680211781,-2.90029390173762,0.00571774807819795,0.0354483617288682,-2.57463230301729

"PXMP2",-0.415925297732182,8.26474554963406,-4.44920420535004,5.49691536163372e-05,0.0015041343317385,1.72728914641731

"TMEM219",-0.415918737404827,9.56040792351848,-3.95332842558065,0.000265839365324878,0.00435893712767805,0.249650671697462

"PLK3",-0.415756480156148,9.71053198676284,-3.55728115086522,0.000887178295207494,0.00987600032543053,-0.87040796574572

"SCAMP4",-0.415610017205418,10.5513727665724,-5.21720472213914,4.30728107500773e-06,0.000293373736545063,4.13275818989133

"ALPK3",-0.415348581062503,10.9764071619504,-3.16741408488414,0.00274251288741183,0.0215479040400066,-1.90776244140286

"MKRN3",-0.415264939805469,7.68957847368747,-3.33886267132733,0.00168253942057702,0.015301956123387,-1.46034740816863

"SRP54",-0.415246233203083,9.77188702642832,-4.80298263822575,1.72296811167098e-05,0.000692633180891733,2.82115237623543

"MATN2",-0.414553929001533,13.3085000276003,-2.99533800454581,0.004419508140178,0.0298004452015244,-2.34177484885076

"CTPS1",-0.414010861197362,9.38114325868669,-4.56641187560793,3.75350576633195e-05,0.00115570507149591,2.08652176044155

"TMEM263",-0.413547096772319,11.0647590906177,-3.85069073846501,0.000365144135053073,0.00542981166414332,-0.0463250472436467

"USP44",-0.413406308622645,7.48829659054968,-3.38521613203482,0.00147119474426718,0.013999044032019,-1.3369556009359

"ADAMTS2",-0.412684143622187,11.0364926978213,-3.56476584558268,0.000867646845388285,0.009749581920321,-0.84981968774643

"CDK10",-0.412621057848977,10.2318599946013,-4.26136743362972,0.000100632637782998,0.00224186457945212,1.15900661602667

"APCDD1L",-0.411980276208752,9.39702989993006,-3.33428489314099,0.00170490985650508,0.0154347836148884,-1.47247868422528

"ZNF765",-0.411669950321217,9.92085848935653,-5.62363030043326,1.08244219453831e-06,0.000112580281500558,5.44245762807538

"NF2",-0.411484069849555,8.9247349788479,-3.06827534634918,0.00361630373144471,0.0261065607150179,-2.15972204161768

"MC1R",-0.411152395986244,13.0757432624151,-5.1437648034443,5.51740755993684e-06,0.000340347944274862,3.89822911294246

"MYLK4",-0.411149202166484,9.69424546148143,-2.71374301524689,0.0093554190029703,0.0496647293400528,-3.01660634155663

"ZNF212",-0.411026017795205,7.91355557864011,-2.96524718760072,0.00479724210800881,0.0314687117715994,-2.41604000627863

"HOMER3",-0.411006855347402,12.2389162682954,-6.50619334188584,5.20525312208894e-08,1.7569202471896e-05,8.32439313062215

"CHPF",-0.410184184736814,13.6677048579645,-4.2110263571362,0.000118159121520066,0.00248676297043818,1.00838306781147

"CSNK1G2",-0.409903713895149,8.7730997559451,-3.4254422622751,0.00130849570071187,0.0128967931625536,-1.2290693620631

"AGO3",-0.409344675276824,10.6284460932096,-3.97955782305471,0.000244998053107964,0.00410418252680633,0.325881542860322

"SRRT",-0.408908430047337,10.2557323105343,-3.17337531443086,0.00269689807466642,0.0212719619302062,-1.89245057009583

"SMPD4",-0.408798250690724,11.2157379663873,-4.53903051198722,4.1044956103686e-05,0.00123820104509079,2.00230100075038

"SRRM2",-0.408521436657537,14.0524397587122,-3.33300683890522,0.00171120565608511,0.0154727852893972,-1.47586380522953

"L3HYPDH",-0.408502791374962,9.73715900044714,-4.23793809034044,0.00010844942310237,0.00235444586545926,1.08881232787879

"ANKRD9",-0.408244074069886,8.74892356559204,-3.13673407766596,0.00298901277469479,0.0228408584051752,-1.98627939340628

"DGCR8",-0.407779905467891,12.6671138687097,-2.91119608063614,0.00555254651755096,0.0347549001583167,-2.54817862916874

"POLDIP2",-0.407326996074012,9.24444212839039,-2.74257627454173,0.00868001796235067,0.0470393339377434,-2.94964205113094

"CPT2",-0.407215421934799,8.40785862253732,-3.61484996588242,0.000747164075775891,0.00879343299444402,-0.711445071750693

"NEK8",-0.406870029241469,8.5269778487714,-4.35827272752806,7.37427728148442e-05,0.00182712529485422,1.45098949024151

"HSPG2",-0.40677488928069,13.0735809877806,-2.7414131294997,0.00870636894533901,0.0471678479900574,-2.95235313857217

"DDX18",-0.406494610981555,9.69271079733723,-3.63724809288699,0.000698634428061933,0.00839910704543005,-0.649225026228256

"AP5Z1",-0.406385323460567,10.1368191023376,-5.8132164907912,5.65600129893488e-07,7.33103218908613e-05,6.05862355974465

"TXLNA",-0.406383766442341,9.67800539088622,-3.54467099730506,0.000921040151879391,0.0101456202444399,-0.905040937453778

"ARGLU1",-0.406179220942203,9.28738249888117,-2.861918051951,0.0063363772486109,0.037898151436105,-2.66721145328109

"ST6GALNAC4",-0.405395577403887,11.8706839364006,-3.69910309377037,0.000579824212614176,0.00742984038197332,-0.476337768748041

"ZNF630",-0.405284321698527,11.0778493278188,-3.20631679301333,0.00245747507813659,0.0198293963341387,-1.80751395142566

"NOP2",-0.405079997975308,10.4107065320382,-6.15625748377065,1.73908860919647e-07,3.61750652673438e-05,7.17866640444925

"CARS2",-0.404880022288806,10.7933321255863,-5.18153643932848,4.858104989885e-06,0.000314879131029177,4.01875742494678

"BRWD3",-0.404547426248842,9.63804268559221,-3.8713107228726,0.000342679741423983,0.00519337721125055,0.0128338615641681

"DLG3",-0.403916540414094,7.05865308366881,-3.01700301619361,0.00416498315285308,0.0286897896116244,-2.28799822219526

"MXD3",-0.403810679936051,8.12917063956506,-5.26031294436761,3.72345967601933e-06,0.000264321310096467,4.2707677035757

"ASAP3",-0.403599612455546,11.7331074730004,-4.13640244984531,0.00014972266727157,0.00295776694264879,0.78649751447155

"CCNO",-0.403240427806077,9.54102573553647,-3.8343696751726,0.00038392679970077,0.00562495210470686,-0.0930400030136962

"ACADL",-0.402979358426935,6.84238151504803,-2.85251823990136,0.00649711566372614,0.0385623431016579,-2.68975908742491

"ABHD2",-0.402606290028956,9.87992839801919,-3.98280507432038,0.000242530673106376,0.00407383212319245,0.335335466640871

"SFPQ",-0.402562649074039,12.0534445492291,-3.63508945016367,0.000703176496066001,0.00844224181250513,-0.655230525049643

"DNAJC4",-0.402504415360271,12.223227260088,-4.44693928252262,5.53742096921443e-05,0.00151234998043171,1.72038092978236

"S100PBP",-0.401813269389589,8.98854594665366,-3.1859016014837,0.00260337077340572,0.0206810726378448,-1.86021714249968

"PCMT1",-0.401699009335481,11.0693642410997,-2.95410243079311,0.00494464723325948,0.0320579337239447,-2.44341891022726

"RILPL1",-0.401636184716368,10.2498933713837,-4.36418324528293,7.235216075208e-05,0.00180769246325972,1.4688822584705

"CDK11A",-0.401569420640104,11.2513665690709,-4.49356045338968,4.75963348567555e-05,0.001362321334804,1.86284073577385

"CYP27C1",-0.401309608413341,10.1467813268047,-4.22845136046985,0.000111780053572638,0.00240629768755828,1.06043562839403

"FHOD1",-0.401269546486914,10.8589229930726,-5.19305734545896,4.67297332035681e-06,0.000308467969475509,4.05556058955677

"KIF1B",-0.400589390221588,9.25941620382672,-3.85301314149654,0.000362544640455395,0.00540013411582562,-0.039669802204644

"GNPTG",-0.400203158208104,15.2430881426512,-3.57490764319547,0.000841837627611017,0.00956167194941809,-0.82188456771309

"FOXI3",-0.399798066321164,12.0864854825624,-2.89626010807641,0.00578002449529879,0.0356300025647161,-2.5844030605305

"CKS2",-0.39966931529738,9.36175018779156,-5.06850391395352,7.10573711565602e-06,0.000391121634652217,3.65870138490848

"IFT122",-0.399260023370033,10.7855218096806,-4.39301704306202,6.59272077285058e-05,0.00170906428924127,1.55630458827913

"PIK3CB",-0.399122260617403,10.8678080116702,-5.86106592320084,4.79961927196117e-07,6.70784290282135e-05,6.21451744177989

"SETD1A",-0.399011838205878,10.9900530791314,-3.94165813765783,0.00027565475289619,0.00445053057270753,0.215809476867312

"SLC35E2B",-0.398376047356313,11.4955333925261,-2.95399783561218,0.00494605048231244,0.0320579337239447,-2.44367553900474

"PIP5K1C",-0.398135125439294,11.0821624142869,-3.29480259055011,0.00190992526869153,0.0166992439548498,-1.5766947680503

"GALNT10",-0.398055035910467,11.0154488204925,-2.87835748104356,0.00606411509765989,0.0368106396274305,-2.62765550915993

"IFT20",-0.398042569977704,9.98049688783972,-4.884359046531,1.31496353345746e-05,0.000587243181086563,3.07650294999255

"DMWD",-0.397826583565282,9.53815798914474,-6.49124742666273,5.48056384583546e-08,1.78257830251183e-05,8.27544615789891

"MON1B",-0.397371447723538,14.3371141604149,-5.66221886457051,9.48671351895174e-07,0.00010223362538586,5.56765547137956

"ATP8B3",-0.397064986993749,8.55627420594997,-4.18525487562936,0.000128248054587147,0.00263704534311433,0.931563449790665

"EVI5",-0.39664630096212,8.36317510228656,-2.87101187614276,0.00618439623501931,0.0373254602726925,-2.64534939490599

"B3GNTL1",-0.396539904388266,9.98634892541206,-3.96312324874177,0.000257863259722825,0.00427121838257557,0.278089837584501

"TMEM126A",-0.396424754841336,9.62831785524037,-3.06500347265331,0.00364918358164021,0.0262485907084687,-2.16794977036393

"VPS13C",-0.396309857268401,9.15035251548713,-3.33300252964661,0.00171122692115185,0.0154727852893972,-1.47587521764934

"SIPA1L3",-0.39618843270495,8.83545029302955,-2.717700587231,0.00925993126364195,0.0493407371957581,-3.00744483413676

"BARHL2",-0.395549547629622,14.6494982071817,-3.3154085759197,0.00180017276694531,0.0160218465238384,-1.52239670756727

"YTHDF1",-0.395535161552122,11.7723872435756,-4.69379352857352,2.47139424916226e-05,0.000875460826203241,2.48058890515439

"CDK11B",-0.395417721814097,10.8091204432797,-3.14407633030194,0.00292818865974051,0.0225455704637493,-1.96753286349175

"GBE1",-0.395337520937852,10.4389802902169,-3.06345903686575,0.00366480134891206,0.0262658779369743,-2.17183156372213

"TMEM38B",-0.395181064757204,7.97723145497442,-3.7142133201533,0.000553899463359136,0.00720633272729569,-0.433871583681144

"TCF25",-0.394232765444059,11.1795179425388,-3.66117656553522,0.000650135421748937,0.00800431697155315,-0.582527023715619

"GLT8D1",-0.394128104435941,12.3838455357662,-3.90825640252293,0.000305724966026413,0.00477499062345292,0.119215285147559

"SLTM",-0.393554573397179,8.26465325677506,-3.16048170529182,0.00279647419613455,0.0218264078946994,-1.92554604404364

"NEUROG1",-0.393413291271004,12.0568041161862,-3.20066887502614,0.00249704522635306,0.0200492109758662,-1.82211537602313

"KCNMA1",-0.393138407594531,10.4008947698949,-3.74005721328012,0.000512122381093217,0.00686243990664911,-0.361031318268302

"ALG13",-0.393050560122632,10.7158123404456,-3.6922538735487,0.000591956124357087,0.00751561611683742,-0.495557214728746

"NEDD1",-0.392955085435739,10.4182017055196,-3.52398117678069,0.000979292679180679,0.0105597147304781,-0.961716413224104

"PPIL3",-0.392616481239381,9.07063837347708,-3.05697075305207,0.00373110099832815,0.0265851123530249,-2.18812539396501

"BOD1",-0.392201244419683,8.8281620961395,-2.76022665019725,0.00828908161018093,0.045738242111205,-2.90840237858964

"USP35",-0.391487301856637,12.069908265686,-3.01660403114872,0.00416954375452863,0.0286991028183004,-2.28899088243981

"IAH1",-0.39139600800226,13.2769122188276,-2.86119382937446,0.00634862892512835,0.0379454135788911,-2.66895048021051

"TRIM59",-0.391360330512629,7.51591342274995,-2.72336616215966,0.00912479596122617,0.0488576698444702,-2.99431286288011

"RBPMS2",-0.391326114677721,7.57203791206531,-3.31537494691355,0.00180034690618223,0.0160218465238384,-1.52248548756749

"SBF1",-0.390783072230311,10.5863337330894,-5.19701466208836,4.61100607473118e-06,0.000305943627613589,4.06820637616086

"KRIT1",-0.390739039029546,9.1032881007566,-3.89175510050356,0.00032172775675351,0.00495300158396174,0.071640971359745

"UCN2",-0.390691124128132,10.3326385189832,-4.32245534389101,8.27452857805856e-05,0.00197627559055927,1.34276410847936

"CAPN15",-0.390448831553735,8.53869131012607,-3.83971959094698,0.000377669533080989,0.00555146284082647,-0.0777380093254614

"TSC2",-0.389509142759977,10.6351239670128,-5.33123066375608,2.92870619883722e-06,0.000222943085919145,4.49832781934354

"PKD1",-0.389493949690145,11.5150633320276,-4.06018863245363,0.000190371735873695,0.00347281869683164,0.561674101309112

"FBXL17",-0.388999633328485,9.27367531439723,-4.33885921869308,7.84964612754503e-05,0.00190791195075616,1.39228621541361

"OAZ2",-0.388772508374156,10.0846057494909,-5.71888958081785,7.81441211384327e-07,9.07740378600924e-05,5.7517306097685

"TNPO2",-0.388584610715034,12.5737001374187,-2.88218070013719,0.00600237618882433,0.0365474838808299,-2.61843402837953

"COL5A3",-0.38781527298778,9.87972085469518,-5.13269396176076,5.72688698746535e-06,0.000347866494216697,3.86294143160157

"KMT2D",-0.387575763083307,11.2320490616101,-3.40823146104595,0.00137589016093069,0.0133622687778985,-1.27531923848867

"EIF4ENIF1",-0.38755907891553,10.0854992387007,-4.86683951239935,1.39387100956872e-05,0.000605217439081914,3.02142276780349

"RBFOX2",-0.387419338977281,7.50461662030768,-2.91015861368917,0.0055680736890627,0.0348277168614135,-2.5506988933039

"NEUROG3",-0.387144081258878,15.6090800997711,-4.4220063913273,6.0030685061889e-05,0.00161244583344164,1.64442039854945

"PLAGL1",-0.386945262062406,11.3866571724263,-4.9443096502883,1.07681719098688e-05,0.000509608008718632,3.26540559029463

"ARHGAP28",-0.386683663334024,8.83784993060982,-3.24492683797213,0.00220243099353058,0.018497318330173,-1.70727087222093

"CD44",-0.38653463588939,11.8301144653743,-3.37740059369181,0.00150496371150206,0.0141942455965608,-1.35783052868125

"SH3PXD2B",-0.385947165915441,11.3302598234956,-3.21799484291254,0.00237752873579472,0.0193765884075771,-1.77727239892391

"ZNF587",-0.385772357813082,9.61793470028634,-4.11533833278862,0.000160025778722783,0.00308148671213333,0.72417609950319

"B3GNT8",-0.385707423867297,15.2349669864423,-3.10366559514017,0.00327800636945622,0.0243796632986653,-2.07036550419427

"TMEM25",-0.385466827376961,11.5955470142485,-4.92147059454761,1.16206087170421e-05,0.000542770416029157,3.19336450769985

"PDK1",-0.384632776368084,8.68748897833909,-3.09579693996564,0.00335054442278517,0.0247268519716188,-2.09028993652364

"FBXW5",-0.38441002882986,11.3647220888148,-4.00224852644703,0.000228252788222819,0.00391863160126489,0.392017249490677

"TIPRL",-0.384272808655073,9.29304927084977,-3.06215457326726,0.00367804132765492,0.026339664255572,-2.17510922224674

"TOLLIP",-0.384165063648778,10.250197458943,-4.35239000867517,7.51527481729917e-05,0.00185180098080806,1.43319027197894

"CABP4",-0.383638379179866,11.3677246045578,-3.46850880213541,0.00115336984638892,0.0117431036892723,-1.11275222609062

"CSTF3",-0.383503269472667,9.2349868217519,-3.51706795822766,0.000999530459130015,0.0107141686368026,-0.980612545482805

"PTPN23",-0.383088207750768,9.97512520697761,-4.21471984097178,0.000116778047124561,0.00247223962723228,1.01940885894699

"EDARADD",-0.382921029503066,12.7422406916187,-5.01042660114907,8.6329224647342e-06,0.000437491076406884,3.47446573310711

"C3orf18",-0.382001910105622,9.80955177687407,-3.04879795949423,0.00381621650263778,0.026983516606991,-2.20861755224796

"CDA",-0.38143979661368,14.1153605165011,-2.75840922975602,0.00832857085645889,0.0458995083337009,-2.91265736193878

"CEP95",-0.380884615713651,12.0610783975469,-3.53835427208876,0.000938463912041511,0.0102931826624835,-0.922363830797323

"DDIT4L",-0.380066415452054,9.98774944473279,-2.8651556762416,0.00628187462131811,0.0376722947035735,-2.65943347327358

"METTL17",-0.379969208726264,11.7559852495268,-3.03722665038265,0.00393984627300084,0.027656382907438,-2.23756992211838

"POLH",-0.379874728684193,6.60409306577124,-2.74927819686956,0.00852961523480494,0.0464776993406718,-2.93400519045535

"AP1G2",-0.379830334782536,11.0573159141488,-3.63789067798254,0.000697287791894457,0.00839910704543005,-0.647436939972771

"LTBP4",-0.379704819748007,13.259420347638,-2.78917047199659,0.0076830643940112,0.0433982756376591,-2.84037314589985

"TPCN2",-0.379690066362274,10.4252976645067,-5.24552589749848,3.91431744275743e-06,0.000274600881307795,4.22339976274053

"UHRF1",-0.379586809831791,9.49104752041212,-4.62474367857099,3.10100665954736e-05,0.00102315676499534,2.26652423466903

"GYPE",-0.379559978514154,13.0819265432735,-2.86770310092377,0.00623929764468647,0.0375049716282918,-2.65330937316638

"TRIM35",-0.37950298751807,9.13071032215515,-2.93919457730987,0.00514844552690012,0.033058342437443,-2.47993456737892

"SAFB2",-0.379451614968776,9.58334547077729,-3.98350936164647,0.000241998715303372,0.00406871712223874,0.337386375836243

"GATB",-0.37901279127238,9.56187622694714,-3.8616912881834,0.000352988313444358,0.00530193781629397,-0.0147836432646455

"SPDYE5",-0.378537936706964,9.85893168880891,-5.28589408704189,3.41478603129135e-06,0.000251387272896177,4.35277952903847

"GRAMD1A",-0.378231695187942,11.8594793737138,-3.49112305109919,0.00107910582183536,0.0111972877301698,-1.05134299485623

"ERAL1",-0.37790377154872,10.5058823001139,-4.27308154698623,9.69331341370894e-05,0.002194983337441,1.19416155924196

"CEP120",-0.377588614888925,7.15188900034314,-3.40028628212518,0.00140810582742889,0.0135844037242898,-1.29662437633494

"RBBP7",-0.377468346649355,12.109730431929,-3.55297506802562,0.000898605105959425,0.00997217539733757,-0.882241977796741

"RAB2B",-0.377201059206538,9.43864646850023,-4.0430828384458,0.000200870237953722,0.00359880673131757,0.511470539521253

"WWP2",-0.376929966712144,11.3003825104091,-3.17042120532555,0.00271941242844893,0.0214212104502523,-1.90004068088428

"NKTR",-0.376578243945184,10.8865435547256,-4.37707675766294,6.94068918861704e-05,0.00176116296305206,1.50794713472422

"LSM8",-0.375767190742724,8.91671179861005,-2.82822495808101,0.0069302468403304,0.040317133569649,-2.74779528972397

"RARS2",-0.375704945947305,11.2801378299944,-3.44916290935381,0.00122074921296348,0.0122616410279077,-1.16510593661923

"GPD1",-0.374888620639791,16.6399477866216,-5.6738623414485,9.1163355074222e-07,9.94403206660218e-05,5.60545528067411

"IFT172",-0.374503061524244,9.38454691706975,-4.11850333690915,0.000158434826985873,0.0030679430681546,0.733531364532721

"SYNC",-0.374456016645317,9.1325626171576,-3.51893445418323,0.000994027410244433,0.0106735632304098,-0.975512831501308

"KMT2B",-0.373956611505244,14.0120791179281,-9.22700842049414,5.32816537753704e-12,7.70330031065904e-08,17.0034803425936

"CDH3",-0.373943077667859,9.41053239554635,-3.75330413802948,0.000491901330291519,0.00670702964754953,-0.32359447698883

"GLIS3",-0.373659147171034,8.88215270070669,-3.39136584037636,0.0014451316318846,0.013817188542375,-1.32051018231935

"HP1BP3",-0.373639480844197,11.7778946975735,-3.99245768941715,0.00023533756174912,0.00399426341758065,0.363458889039535

"TBC1D23",-0.373419445210276,8.07383312654141,-2.82732819843459,0.00694673687399324,0.0403736763933934,-2.74993106587083

"TAC4",-0.373386079322637,11.7797792445902,-3.85668807993925,0.00035846770768708,0.00536172978496169,-0.0291346049162877

"TANC1",-0.373354884565218,9.39773792286788,-2.9996591839921,0.00436760751399505,0.0296212949835779,-2.33106927155041

"LCE1D",-0.373256071923331,14.8407340222442,-3.86200488625421,0.000352647575697805,0.00530193781629397,-0.0138838299137261

"LCE1A",-0.373030226522623,14.2627825009781,-3.4927426001863,0.0010739663467853,0.011169874405606,-1.04693643648213

"APPL1",-0.372146884612667,10.1501509132039,-3.09736921547637,0.00333593060647385,0.0246596952971945,-2.08631132772393

"SLC35C2",-0.372051069203314,10.7763897389971,-3.91556505654993,0.000298885610799742,0.00470665905950403,0.140317533893067

"C18orf21",-0.371246792948755,12.4805329494235,-2.96428668555119,0.00480978254152529,0.0315075457666307,-2.41840234417208

"ALX1",-0.371054479782039,12.8086911761249,-2.84578904704715,0.00661450399157644,0.039064662233513,-2.70586928017263

"EHMT1",-0.371016787691193,10.4531451897422,-3.99869650524769,0.00023079885055129,0.00393965709686263,0.381652812404123

"LRRC75B",-0.370945834253707,8.57730990810269,-3.56744437677596,0.000860757732052179,0.00970264339551445,-0.842446027054308

"ZNF17",-0.370442744021496,8.48645603061904,-5.52894056102115,1.49542071285158e-06,0.000136487658837765,5.1357843468272

"EVL",-0.370263068437806,11.6673557931966,-4.86802861023635,1.38837094016871e-05,0.000604296052279274,3.02515940492103

"ZNF664",-0.370106418884179,9.40375878652554,-2.78387541537009,0.00779077164370956,0.043826765388151,-2.85285578895079

"SLC36A4",-0.3698747641669,8.25693557629267,-3.27763377419813,0.00200618811951945,0.0172957586843775,-1.62177968417723

"SLC25A29",-0.369750620978687,10.8842519597555,-5.23474187451424,4.0595811760449e-06,0.000279314798685643,4.18887300589425

"UBE2O",-0.369553478088671,10.0529532287922,-4.12578784292801,0.00015483130447039,0.00302047677826697,0.755075205668223

"ANAPC5",-0.369215865091991,12.2618790403339,-3.21879003281121,0.00237217536585509,0.0193505905698959,-1.77521069733175

"ILF3",-0.369060916743546,10.5486189705173,-6.44052224729236,6.52818528792531e-08,1.94637844359493e-05,8.1093187117213

"EGLN2",-0.369048174412825,10.0376986053649,-3.68635382356381,0.000602602091036962,0.00759686314768161,-0.512098247242087

"APBA1",-0.368862027526628,11.3933717802756,-3.72914919240367,0.000529371651989667,0.00702517023920115,-0.391807095585293

"IFNGR2",-0.368827705564458,10.4514145309644,-2.75622951210937,0.00837616140861237,0.0459783109685539,-2.91775795612618

"CRYZ",-0.368809790056702,8.73005090366416,-3.72122019679413,0.000542259567815328,0.0071224634392063,-0.414148731355118

"NIN",-0.368712075172826,7.98888731129098,-3.39211464679905,0.00144198829374826,0.0138018879544476,-1.31850654936928

"SMG6",-0.368644542731682,10.0618279285971,-6.29554965255517,1.07614923786785e-07,2.61003543786946e-05,7.63456524892958

"CD99",-0.36855537233596,11.6628007468665,-3.79405061061424,0.000434409534509019,0.00615634038745371,-0.20801955924889

"SLITRK5",-0.368011822926677,8.26268414322828,-3.6132398482617,0.000750774556302631,0.00883011573813134,-0.71590986620116

"E4F1",-0.367849937126168,8.28865984629188,-3.44922157983775,0.00122053930605635,0.0122616410279077,-1.16494741594709

"RNF4",-0.367593185106911,9.42372411118108,-3.07924717215191,0.00350806519213103,0.0255608648637283,-2.13208996683771

"TPX2",-0.367563591773063,9.23797927998318,-3.93943719343219,0.00027756201122863,0.00446998531392399,0.209374605255816

"INPP5E",-0.367260122160486,9.22241982653312,-3.75017634173851,0.000496605322865631,0.0067352332227015,-0.332439993642091

"DDHD2",-0.367036690615988,8.56863113059482,-4.20321458755904,0.000121132700815764,0.00253147533281916,0.985076742584824

"SMG9",-0.366852747721765,9.64552546978889,-4.10230971409851,0.000166739901959646,0.00318676293392746,0.685698819493887

"KIF14",-0.366825961267821,9.16771196447344,-5.01726538013012,8.43748856949941e-06,0.00043248777369563,3.49613167887084

"TRUB1",-0.366794378479812,7.09049434014251,-3.41295257809892,0.00135708104805881,0.0132489386959203,-1.26264580593391

"MAPK1IP1L",-0.366785894372615,8.76744336744784,-3.2220757332266,0.00235017601571627,0.0192589549909062,-1.76668847399581

"RAB15",-0.366159920791157,9.64849741717656,-5.75079559124624,7.00548220373301e-07,8.52524293486937e-05,5.85546971576898

"INF2",-0.365320400614314,9.90832494837773,-4.72726681777734,2.21318281002802e-05,0.000809130015056374,2.58473145059008

"CSRNP2",-0.365318337360007,9.32404777036229,-2.84828038150509,0.00657081579106885,0.0388602486545032,-2.69990789246933

"PAIP1",-0.364492396001738,8.67697409960725,-3.37285631474314,0.00152493611262628,0.0143049722699378,-1.36995506567627

"SSC5D",-0.363568270213522,14.6614619052374,-3.63204868351457,0.000709622769416462,0.00849694894383607,-0.663686916512814

"SLC24A5",-0.363239544775919,14.6244111481463,-3.20097691380526,0.00249487162935859,0.0200407537393785,-1.82131942304505

"TEX30",-0.363154548606753,9.87923255602169,-3.90229779792933,0.000311412713028615,0.00483581772861883,0.102025052658044

"BRMS1L",-0.363047775410051,8.59483724134765,-3.93870759702277,0.000278191341504748,0.00447532815483673,0.207261078216319

"LYPLAL1",-0.363008937605512,9.24028083355044,-4.30172044508376,8.84380405662482e-05,0.00205998451522085,1.28027379244797

"ZNF100",-0.361846851074391,8.14327619664893,-3.48478978772575,0.00109943025914256,0.01133585470075,-1.06856380735523

"SLC25A32",-0.361582874687549,10.0865398016543,-3.26843529611559,0.00205963677241615,0.0176209964581417,-1.6458759325005

"SPACA3",-0.361527604205573,15.618090315179,-3.08570853587804,0.00344575513229799,0.0252524021145755,-2.11578754378464

"PALLD",-0.361500000962032,11.0304847239769,-4.62809171785243,3.06713797753081e-05,0.00101607465333423,2.27687931070236

"MICALL2",-0.361453173281163,10.9623831558923,-5.43799925051772,2.03818517159002e-06,0.000165698390386007,4.84205150191888

"TAMM41",-0.361310403046145,10.6220581396479,-5.0144159753306,8.51838064777307e-06,0.000434322529490093,3.48710354037349

"SEMA4C",-0.361154838924158,10.6820067963495,-2.79694161713914,0.00752748492042946,0.0428441545471087,-2.82202330683174

"CST6",-0.361079164821939,10.935156546735,-3.07608321110173,0.0035389615915392,0.0256725401099127,-2.14006480179087

"BIRC5",-0.360935299056239,10.7354784347017,-3.76344705224649,0.000476940446196552,0.00657318000154863,-0.294884038659972

"GPSM1",-0.360515940518503,13.4997831201637,-6.25879675336173,1.22148683440198e-07,2.81038155910689e-05,7.51423966334022

"MCTS2P",-0.359977604526607,9.49955350583934,-5.18456311492238,4.80877792903307e-06,0.0003128153758999,4.0284242450674

"TFPI2",-0.359958291191681,9.92448367340957,-4.22503607168876,0.000113003326185276,0.00241808194034498,1.05022629299457

"POGZ",-0.35980274178892,10.8342671432832,-2.89986302368348,0.00572437032444432,0.0354506317148409,-2.57567642731061

"PIAS3",-0.359576667617118,11.6412722424811,-3.36157378674558,0.00157561933026459,0.0146088149849033,-1.40001632129644

"KLHL42",-0.35953315037419,11.4392669047879,-2.98500987003831,0.0045459049134246,0.0303541389634988,-2.367321092986

"MMS19",-0.359048244989626,9.32020823979643,-3.57921468111189,0.000831100828514977,0.00947582072740881,-0.810007879915226

"ZBTB20",-0.358839822953778,10.2485046678522,-3.49783059423166,0.00105797180484297,0.0110873213924053,-1.03308525036835

"IDNK",-0.358510418555619,8.67713811601376,-3.3099887005849,0.00182844552488983,0.0161606037523489,-1.53669811937427

"CLIP1",-0.357984840880615,9.22125323436389,-3.20798347591184,0.00244591176515649,0.0197971682042924,-1.8032020651121

"IL36A",-0.357463185069472,11.5507671163475,-3.45220624129596,0.00120990631420446,0.0121732362512956,-1.15688117987545

"GOLGA3",-0.357224037924977,10.9964128750324,-4.33203472041065,8.02375829666945e-05,0.00193185749891144,1.37167453971364

"DRD4",-0.357205174934453,11.970485195566,-3.77231147892762,0.000464225082461546,0.00643761434120512,-0.269760149782893

"SCRIB",-0.357005552817984,12.2670578534756,-3.02856544677552,0.00403483170908864,0.0281009624701371,-2.25919392159258

"WDSUB1",-0.356951563736311,9.34920798747183,-5.1514592159646,5.37629459642459e-06,0.000336281587536502,3.92276515653283

"TCEAL7",-0.356887847038246,12.4596965081422,-3.2543556073776,0.00214406834365035,0.0181262942341971,-1.68267909897505

"BEND3",-0.35639685850953,11.0901260844136,-4.74387253412421,2.09511629933319e-05,0.000785734496410305,2.63648247230222

"RBP3",-0.355618012675313,5.2953746885969,-2.8691739393361,0.00621483679307832,0.0374460139411849,-2.64977172741926

"SLC38A6",-0.355224907041723,10.1494444043403,-3.87486392406605,0.000338946036200406,0.00516034522688431,0.0230436262499678

"RRM2B",-0.354631563994838,10.2247838755936,-3.2189606627048,0.00237102813908923,0.0193500558303683,-1.77476826146265

"GLS",-0.354482017565163,8.71365375750781,-3.24994908745207,0.00217115937785074,0.0182948045738916,-1.69417745668573

"H2AFX",-0.354339829207451,9.09822818135951,-5.00185639668101,8.88415100680473e-06,0.000446428588091938,3.44732535804617

"ERCC1",-0.354331147883922,11.9665589127975,-3.50454086690662,0.00103722523928525,0.0109468568174477,-1.01480034864636

"MED29",-0.354152192253331,8.97270382376525,-4.20033290552534,0.000122247914764829,0.00254289877584654,0.976483876888809

"UBAP1L",-0.354033611763867,10.1500445217501,-3.78081789672504,0.000452330752354805,0.00632167564755867,-0.245622730089692

"COLGALT1",-0.353891819790578,10.8185119217356,-2.7338607842074,0.00887927102200589,0.0478293523976704,-2.96993649944939

"TTYH3",-0.353677284569365,12.9035612971561,-2.9113257431901,0.00555060877217473,0.0347549001583167,-2.54786360397337

"HOXC4",-0.353610508078114,12.1269926223326,-3.25290690040595,0.00215293939621581,0.0181841042771032,-1.68646040057644

"CLASRP",-0.353550232708283,11.1502098287909,-3.31234741176609,0.00181608990942161,0.0160831843513085,-1.53047591771864

"TTC9",-0.353487057823157,8.39228246693742,-2.88526531588792,0.00595299143427189,0.0363189046053153,-2.6109879489717

"MAP1A",-0.353145340731357,11.3568245099491,-3.60490773673603,0.000769727140729731,0.00900565652093797,-0.738997316131178

"ZCCHC9",-0.352306687110362,10.2484772526135,-3.43813009015122,0.00126083958967264,0.0125655484232055,-1.1948878173662

"STYX",-0.351956741842985,9.38894514138816,-4.02645419765719,0.000211613127655148,0.00371375834657058,0.462759587785608

"PTGS2",-0.351892472066025,8.76411286702921,-2.81782442623312,0.00712374769608779,0.0411351589849305,-2.77253695828602

"LASP1",-0.351559766573125,10.2748579002488,-3.19052034204173,0.00256966438720617,0.0204851721135165,-1.84831190092011

"CLSTN1",-0.351547747587361,9.984039745171,-3.04230213036635,0.00388516346875201,0.0273628698001987,-2.22487948738543

"CD1B",-0.351381712853144,10.2858182744576,-3.16749027126017,0.00274192535066373,0.0215479040400066,-1.90756686486428

"ARHGEF16",-0.351295760146053,8.6726275494415,-4.33752561249791,7.88337769005587e-05,0.00191138659410788,1.38825738823314

"TUBG1",-0.35125565493216,11.647475517527,-3.80283283687598,0.000422895670735056,0.00604056363093719,-0.183026740833349

"LUC7L",-0.350841102889174,10.9288646667734,-3.39874960555935,0.00141441899929472,0.0136108345768602,-1.30074166272184

"C9orf50",-0.350785428627432,11.0413432088275,-4.53048851973057,4.22043033811953e-05,0.00126889543392639,1.97606377093593

"DNAJC1",-0.350635093969368,9.34553703767668,-3.00836461206903,0.004264776971292,0.0291415566231637,-2.30947095954038

"PLEKHG2",-0.349845627747424,9.94895689065438,-5.66781721772991,9.30680043587712e-07,0.000100902638180246,5.58582884643624

"SIGMAR1",-0.349740500319752,9.35195292603592,-3.20784926336044,0.00244684101401367,0.0197971682042924,-1.80354933872017

"SPICE1",-0.348943534031778,7.92153762732364,-3.29775953601059,0.00189379478085273,0.0165987725794583,-1.56891554631258

"ETV6",-0.348720687166702,11.3514994504325,-4.15348928270723,0.000141841386514594,0.00283825566371318,0.837153044768939

"PILRA",-0.348555316495798,10.7702335325195,-4.03132705264139,0.000208408614181838,0.00368401353665899,0.477024369606045

"DCAF17",-0.348534854728607,9.86625171712787,-3.6530459858325,0.000666240339855753,0.00813847124924813,-0.605216236712087

"FXR2",-0.348378891988075,9.52654733370325,-3.98662204020877,0.000239661173134129,0.00404462143980796,0.346452624404716

"SAP25",-0.348362945169871,14.3119437133253,-3.08062101433044,0.00349472857396326,0.0255068133250219,-2.12862552059025

"ZBTB1",-0.348238473493695,7.47329014496007,-3.27774643835627,0.00200554169639966,0.0172957586843775,-1.62148429547062

"BIRC2",-0.348236579864638,9.70784657797335,-3.29389774285935,0.00191488730261534,0.0167262787873466,-1.57907442814997

"LLGL1",-0.348141837273199,4.57661145333012,-3.01999938583168,0.00413088220039412,0.0285230068737324,-2.28054060979103

"GMEB2",-0.347973322620756,10.8533956064094,-3.55921398286125,0.000882094599906914,0.00983777449983465,-0.865093572751441

"VPS4A",-0.347773523573149,12.3577832027522,-3.22387998974942,0.00233817785271552,0.0191966715053221,-1.76200643539108

"NKPD1",-0.347196607837205,9.60607191819575,-4.7135987100539,2.31524827391525e-05,0.000828349527441398,2.54217848058995

"RTBDN",-0.346813863058983,11.8770002724364,-2.9628337412524,0.00482881060056805,0.0315956813582889,-2.42197486522778

"DOCK7",-0.34679764385257,9.02200858483765,-3.03080320579092,0.00401008708394451,0.0279892500369424,-2.25361088760308

"RBSN",-0.346414402232847,7.11675067084879,-2.89602430120443,0.00578368451293255,0.0356300025647161,-2.58497395209195

"LRP6",-0.346356312498107,9.69550057349204,-3.06382329196874,0.0036611122528006,0.0262499547456312,-2.17091615714862

"C1orf174",-0.346011667814619,10.0369499546692,-3.7530504310629,0.000492281283653647,0.00670709815939078,-0.324312109802684

"INPP1",-0.345796949681624,9.22106723150637,-4.00677665202098,0.000225046507247392,0.00387847492114508,0.405236017284157

"F2R",-0.345508328640886,9.0180102237256,-3.29552700654289,0.00190596148467703,0.0166727359410207,-1.57478934147434

"ITPKA",-0.345440957731158,9.70335653481195,-2.85544284353766,0.00644670289164406,0.0383394508073872,-2.68274920743135

"DNAJB7",-0.345344985503772,8.64478460085511,-3.01087784299116,0.00423551461005062,0.0290151354583426,-2.30322792374435

"DCDC1",-0.345328869635233,15.2382180157154,-3.25230187614263,0.00215665449711971,0.0181982982542332,-1.68803928300479

"WNK3",-0.345280136308314,8.08552937260747,-4.04124119616052,0.000202033462934209,0.00360506836400268,0.506071248801929

"SHKBP1",-0.344897955574423,12.5998344074504,-2.90959861894523,0.00557647161583554,0.0348436956813419,-2.55205900650036

"CDAN1",-0.344664868667939,9.1262888794325,-4.07433421215612,0.000182094065160642,0.00336864605135338,0.603261774005858

"ANKRD36",-0.344556920972959,10.3737132778915,-4.6642455670408,2.72375835397297e-05,0.000944289015391909,2.38885790293434

"ZNF106",-0.344259991439886,10.4101384484869,-3.66012950751457,0.000652188246282139,0.00801855363418639,-0.585450447963676

"MEOX2",-0.343880992495524,9.10035781156576,-2.95238323907048,0.00496775915709759,0.0321636784514364,-2.44763625010791

"SHARPIN",-0.34300705002984,11.7027353445796,-4.23046082975215,0.000111066348153269,0.00239381433989618,1.06644414640465

"ODF2",-0.342239282237541,10.4943685715047,-5.39111012644361,2.39026418226301e-06,0.000190890339091531,4.69094121725183

"SRRM1",-0.342073655511552,10.8555976259505,-2.98865098249117,0.00450096349377601,0.0301338832111374,-2.35832160735212

"MAPK15",-0.341993857999149,12.4151244128635,-3.1803759181529,0.00264424430368615,0.0209583014393626,-1.87444596575481

"LZTS1",-0.341549226770697,9.8311123760496,-3.85455491642873,0.000360828791268234,0.00538803526460555,-0.0352504914815333

"B4GALT1",-0.341545007009882,11.1209577190219,-2.93013934341716,0.00527603531751704,0.0335405102327869,-2.50205411167784

"MIER2",-0.341379338085829,9.42704125509473,-3.22891723752025,0.00230498645851422,0.0190193278396499,-1.74892624495181

"CAMK2G",-0.341337787405628,8.42441225158696,-2.84165156168779,0.00668765568588793,0.0393661282384411,-2.71576171443095

"MRPS25",-0.340955608442435,10.3656621769753,-3.17973891602993,0.00264899496859303,0.0209789447739061,-1.87608527553861

"PKLR",-0.340080755843697,18.3071429999668,-5.11166875766212,6.14658408168686e-06,0.00036530313168537,3.79597467278284

"PLEKHO1",-0.33899289667489,11.8144770985983,-4.27486422243493,9.63818878892899e-05,0.00219360762398411,1.19951495254093

"TSPAN10",-0.338780278796746,10.9372357043053,-2.8213685579957,0.00705725365056963,0.0409229207633841,-2.76411297619176

"C6orf223",-0.338661260061579,8.422570195581,-3.06459821379681,0.00365327561259884,0.0262499547456312,-2.16896847281391

"SNX1",-0.338587147299101,8.8380485821899,-2.77919698878464,0.00788709694846501,0.044201841262873,-2.86387094979934

"TMUB1",-0.338355366730717,11.0321497763453,-4.72704249338519,2.21482169463183e-05,0.000809130015056374,2.58403274503234

"WDR34",-0.337896033214145,10.5854382305118,-3.73764474541191,0.000515890076941214,0.00688199670872586,-0.367841818898667

"CDH23",-0.337865626527416,11.1299324217674,-4.45286789922697,5.43200709254489e-05,0.00149727542185725,1.73846650715323

"TRAF5",-0.337763790179478,10.0696597837873,-3.71500735271755,0.000552568430460129,0.00720633272729569,-0.431637522084553

"ACE",-0.337711098970559,14.8744928803521,-2.82741982978393,0.00694505024583347,0.0403736763933934,-2.74971285270783

"C4orf46",-0.337601097027227,10.6416133782633,-2.91691643094353,0.00546765560342305,0.034331657104119,-2.53427157145229

"EMX1",-0.337121383942943,10.3142800797747,-3.83582486239154,0.000382214974469147,0.00560446203137588,-0.0888788774170948

"RUVBL2",-0.337030631547194,10.6793064412641,-4.63316286468814,3.0165284582625e-05,0.00101243297541947,2.29256854213993

"CRLF3",-0.336890981199286,9.67495877518388,-3.21321407381943,0.00240995533625094,0.0195873198592427,-1.78966093635558

"PHPT1",-0.336836673592614,15.6673415856494,-2.86696630490631,0.00625158459435689,0.0375535919437375,-2.65508104044684

"TMTC3",-0.336631703247145,8.66148444601802,-2.76951009169907,0.0080900550669329,0.0449257367447558,-2.88663700147746

"TXNDC15",-0.336613536799849,9.9935869315693,-4.15986666185657,0.000139004463656549,0.00279398971949664,0.856082484647342

"ZNF707",-0.336176574583789,8.0551598713125,-3.21026590334408,0.0024301598764859,0.019697838708861,-1.79729492890734

"STK17A",-0.33607040269891,8.4532485081347,-3.11071556255314,0.00321427286573537,0.0239883718377722,-2.05248660512015

"ACAD8",-0.336048809146696,10.8374782613803,-3.78866765779145,0.000441615574093604,0.0062171319281435,-0.223324203344189

"GSDMD",-0.335935179309708,9.81608542594732,-5.85263219001622,4.94058406582948e-07,6.7986237195095e-05,6.18703055246993

"ETFDH",-0.335705193134103,13.223350792308,-4.40837193611956,6.27367841915069e-05,0.00166513105697606,1.60294976975801

"POLM",-0.335217370430186,11.2766314915932,-5.61372088209209,1.11971711597128e-06,0.000114912481795018,5.41032700787168

"SIRT3",-0.33521366347798,9.24631412877461,-3.44489639402566,0.00123610627409199,0.0123811338954264,-1.17662941342616

"GPBP1",-0.335208301145425,10.0128589438988,-3.52853595468977,0.000966173775878268,0.0104877928863388,-0.949255379785646

"PPAN",-0.334968849241303,10.5078711372735,-4.98721598371499,9.33011075266001e-06,0.000461067268658384,3.40098997340929

"CELSR1",-0.334864127487897,11.6553040980307,-4.48494638528801,4.89480113895163e-05,0.00139210012042457,1.8364778235836

"AIP",-0.334822437311674,11.6200957364517,-3.33190352547379,0.00171665842259558,0.0155019194961193,-1.47878547714106

"STT3A",-0.334718341586735,10.9541953808908,-2.75623627715159,0.00837601331713685,0.0459783109685539,-2.91774213015787

"MAP1S",-0.33450339763327,15.4684350380718,-5.02023812251724,8.35390289415314e-06,0.000432239122369318,3.50555202322166

"MAPK7",-0.3343903443483,11.4090164501825,-3.65004671536936,0.000672277411773861,0.00818677373670701,-0.613579236028489

"CLK1",-0.334008720268136,12.6067008377516,-3.85771012429109,0.000357341755188378,0.00535384142258366,-0.0262037639228296

"SYPL1",-0.333914879209535,9.95269057030359,-3.06855633641792,0.00361349300794572,0.0260968011381272,-2.15901517551409

"KIFC3",-0.333896006108958,11.441708007882,-6.00529108887237,2.92406067205062e-07,5.07849721964209e-05,6.68513737053576

"PTCD1",-0.333829483718798,9.56126643899019,-3.09083385196419,0.00339707078594399,0.024998025211745,-2.10284041421876

"NOXO1",-0.333801789382026,8.39326514644198,-3.14628810543848,0.0029100959154308,0.0224584580807341,-1.96188023861826

"DOCK6",-0.333674142891992,10.6718891706648,-3.75238896216886,0.000493273233608759,0.00671099416787068,-0.326183016889008

"PRRC2A",-0.332893867395406,10.8528813673592,-4.42127514800944,6.01728770353729e-05,0.00161384197494121,1.64219501626474

"ZNF587B",-0.3328596495895,9.57419566015607,-4.81333563987506,1.6648442291411e-05,0.000676872691252389,2.85356849041909

"ESYT1",-0.332571094961477,8.99135572787043,-3.46573409179464,0.0011628109571796,0.0117989365927316,-1.12027137814994

"NHLRC3",-0.332567851803899,9.95379575879989,-3.84640181772999,0.000369992158834715,0.00548096542884784,-0.0586104928728552

"FBXO11",-0.332474925702849,7.5201409364051,-3.26727409305846,0.00206647918497405,0.0176623249593888,-1.64891488384958

"RCCD1",-0.33206454738554,10.1147484949386,-4.86016223144464,1.42515702742837e-05,0.000612851780376589,3.00044489886733

"ZNF598",-0.332010262614078,11.0679012578851,-6.27097617700587,1.17127313613462e-07,2.75696120162003e-05,7.55411176147201

"SH3BGRL3",-0.331817760676511,11.0199303161754,-4.18709505294091,0.000127500699781128,0.00262469507293971,0.937042033728397

"CRTC2",-0.331775594696479,11.0076996998734,-5.10172914558546,6.35543412774183e-06,0.000370333423814898,3.76433936670776

"CCNB1IP1",-0.331657177057865,10.4318608217275,-5.31074219281752,3.1392489540177e-06,0.000236953690035538,4.43252040475082

"C2CD2",-0.331535752877414,11.884950089375,-4.63082757315354,3.03973181673792e-05,0.00101451049383628,2.28534284675251

"MDM4",-0.331245008442815,9.68805320496661,-4.27388534859873,9.6684199831881e-05,0.00219490311014279,1.19657527152703

"CREBBP",-0.331179595579512,8.94854204956954,-3.48208310195921,0.00110822749657094,0.0114068364132091,-1.075918180577

"RABGGTA",-0.331102937458141,9.50965277905444,-3.54126819259566,0.000930387460617926,0.0102108596828185,-0.914374865873263

"ZNF23",-0.330187741817786,8.72457659837411,-3.10083699277265,0.00330391035870948,0.0245040018270953,-2.07753161497522

"P3H1",-0.330148482191913,11.7912590486944,-4.11206800326119,0.000161685978295588,0.00311010802766642,0.714512825234735

"RABGGTB",-0.330138165858807,8.15148734181242,-2.93218999716067,0.00524688548147968,0.0334077249351214,-2.49704892697135

"KAT5",-0.330117761050468,10.0487893569745,-3.1401754348451,0.0029603573949293,0.0227091910111022,-1.97749623862241

"CD70",-0.330053028526741,16.2578161085699,-4.26995972260229,9.7905890792309e-05,0.00221141222270658,1.18478887828621

"GRB10",-0.329417013858187,10.4175525642016,-2.71847933386159,0.00924124787224932,0.0492894106102171,-3.00564096857519

"IER5L",-0.329404740500852,10.2059253230413,-3.44005226882855,0.00125376574652286,0.0125159684372474,-1.18970304664979

"FBXO31",-0.328943600370309,9.74740356718712,-3.52486682618994,0.000976728518117917,0.0105511452056832,-0.959294142052022

"DNHD1",-0.328890280636617,9.42776578982988,-4.52903868615276,4.2404238561799e-05,0.00127276748931547,1.97161226487007

"RASGRF2",-0.328787238993307,12.0554545162311,-2.79657956768707,0.00753466792115731,0.0428511292437714,-2.82287899852841

"IFT81",-0.328629363942037,8.52182995746115,-3.45298158197618,0.00120715862108228,0.0121592683403947,-1.15478511977083

"ARNTL",-0.328520951574124,10.0955775354938,-4.17520249634332,0.000132406505523039,0.00270082095473392,0.901653553081864

"LRRC32",-0.327657079186629,11.782145747426,-3.59097854462349,0.000802439660608738,0.0092518061325473,-0.777529171157378

"CDKN2B",-0.32740975779954,11.5055161630362,-3.28130642363743,0.00198521726787764,0.0171812054693097,-1.61214737362723

"ITGB3BP",-0.327190730434623,11.187890286766,-5.0870900860111,6.67585294277177e-06,0.000377925105358368,3.71777423944541

"DCDC2B",-0.327165714953589,9.76517647830732,-3.62111298673215,0.000733277789513179,0.00868805539260949,-0.694067758580859

"SRSF2",-0.327146186338664,11.1041118730724,-3.95399150287751,0.000265291955740441,0.00435395210664288,0.251574863907645

"VENTX",-0.326992900185154,9.01975689049043,-3.36107863190818,0.0015778799498168,0.0146100902806795,-1.40133425620087

"MLLT1",-0.325951337336337,10.431278681455,-3.21824669617196,0.00237583197685196,0.0193715853390632,-1.77661944899901

"SH3YL1",-0.325383918408943,7.95272362909201,-3.46661787567341,0.00115979585550429,0.0117817081539558,-1.11787679509932

"HMGXB4",-0.325223021059099,9.37483155747203,-4.39215139850782,6.61117160352708e-05,0.00170906428924127,1.55367678084782

"BCL3",-0.324573475063991,12.5219231182027,-4.72922224243632,2.19894711153043e-05,0.000807740551913098,2.59082247674191

"SLC38A10",-0.324536052217928,11.082159270474,-4.62273191585827,3.1215337922417e-05,0.00102315676499534,2.26030331257829

"RCC1",-0.324525240307894,9.1178415134467,-3.36862012393194,0.00154378131396697,0.0144514410913423,-1.38124895609726

"IFRD1",-0.324116067723965,9.30256721654464,-4.51561957190874,4.42993434064993e-05,0.0013012659346451,1.93043493447439

"PPP1R14B",-0.323865452006398,10.3358471804897,-3.3628374838279,0.0015698638973833,0.014596307307843,-1.39665226762674

"MAPRE3",-0.323809313667558,10.4413854578216,-3.10356014454361,0.00327896863416562,0.0243796632986653,-2.07063273263643

"SPATA21",-0.323713145388473,11.8410196618952,-2.82791971952338,0.00693585562758581,0.0403366454232388,-2.74852231668514

"TNNT2",-0.323703454697563,14.7139201671773,-3.70111090006356,0.000576313367235568,0.0074063720017795,-0.470700178084187

"CAMK2D",-0.323688275901265,9.63920190426166,-2.96338904261468,0.00482152996861963,0.0315711382169241,-2.42060961988051

"PGRMC2",-0.323583200818678,11.0595982955171,-3.70696656809409,0.000566190512091105,0.00732407428588126,-0.454249306601275

"BTBD6",-0.323498942558189,11.2615800160436,-2.86517466660721,0.00628155622444831,0.0376722947035735,-2.65938783355339

"ANKRD65",-0.323469530894025,8.55291569199298,-3.07912242378317,0.00350927855695214,0.0255608648637283,-2.13240449803754

"PCDHA6",-0.32325907025669,8.65947758336553,-3.70599252323616,0.000567862431355431,0.00732407428588126,-0.456986721480803

"PABPC1L",-0.32315493380578,10.0476678962047,-5.08761969766889,6.66398745354786e-06,0.000377925105358368,3.71945829960575

"WDR73",-0.323147292435078,11.0937009937684,-3.97311404215434,0.000249966471021897,0.00417131548517791,0.307132018203177

"CANT1",-0.322669021250613,9.88459757786301,-4.71969201410118,2.26918808147945e-05,0.000815130634329034,2.56114394716124

"ADTRP",-0.322635781959718,11.5701635838144,-2.87955168123945,0.006044767495209,0.0367305861826745,-2.62477603430092

"AGMO",-0.321763560930259,8.71302827074624,-3.49538172642917,0.00106564141084802,0.0111301375419445,-1.03975328827462

"PUSL1",-0.321718439199842,9.03826227524079,-2.90228926611039,0.00568717388623279,0.0353379137376931,-2.56979566800458

"SYNPO",-0.321298589210935,11.9400628304067,-4.35029655681606,7.5660831747552e-05,0.00186175600981012,1.42685842695202

"PCDHB5",-0.320882516154399,7.06354503666507,-2.74674780396516,0.00858611656922033,0.0466860301844324,-2.9399122514198

"BNC2",-0.320819618217786,9.29038264988849,-3.34012631510766,0.00167641360001746,0.0152773117120287,-1.45699696456371

"PCTP",-0.320626479361868,8.20690905885531,-3.98535946958857,0.00024060668255821,0.00404911848004122,0.342774758338189

"MRPL4",-0.320382967710131,10.0234904032066,-3.35711179708045,0.0015961021003546,0.0147330600997128,-1.41188849431569

"ZBTB33",-0.320371789235897,7.00869363458685,-2.76317204255541,0.00822545085560945,0.0455275650853952,-2.90150236930138

"FAM50A",-0.320276242445034,10.9275246008225,-3.05850825974667,0.0037152891491576,0.0265214715041023,-2.18426632577557

"SRSF11",-0.32006307415978,11.4909392463392,-3.03864020646919,0.00392454463966125,0.0275750899681462,-2.23403693777812

"PIWIL4",-0.319942559740603,9.67698877070537,-6.21055837142332,1.4423874891695e-07,3.14669143826258e-05,7.35634572887313

"SEC23A",-0.319819620771632,9.29756296625997,-2.77073469850102,0.00806413327852998,0.0448452145562034,-2.88376202639458

"GALT",-0.318997101649991,12.2484311431636,-4.71172597552151,2.32958819912335e-05,0.000829189658191591,2.53635114697604

"ZNF532",-0.318922570678469,10.2016872562608,-3.7698483693573,0.00046772508294689,0.00648112626555919,-0.276744203096111

"RPL28",-0.318899377291366,10.4129013039727,-3.2204632849748,0.00236094791561834,0.0193118414551882,-1.77087140202797

"UBA7",-0.318628825021955,11.4651701607735,-2.97346295919039,0.00469121787212536,0.0310359454565275,-2.39581263783561

"PCDHGA3",-0.318534704456402,11.1872834638142,-3.63901593450485,0.000694935641877562,0.008388464033433,-0.64430534352651

"TIAL1",-0.318431472896149,10.8342556120413,-3.72191327315277,0.000541121097271096,0.00712121351073806,-0.41219682279504

"PLXDC1",-0.318426078093566,10.9658807323818,-3.22936030700235,0.00230208841209658,0.0190193278396499,-1.74777512720217

"IL1RAPL1",-0.317701893533254,9.78188703514137,-3.05663065172987,0.00373460715205722,0.0265851123530249,-2.18897886189913

"WBP1",-0.317649003990221,10.6627521629233,-3.09302776941229,0.00337642952457257,0.0248768318637062,-2.09729410954921

"MSL3",-0.317393204773937,8.07922431497342,-3.39939763603373,0.00141175339801128,0.0135924954451151,-1.2990054977784

"WWP1",-0.317300904851836,8.94818830487636,-3.05627043546678,0.00373832404651478,0.026590409092685,-2.18988274009349

"VIPAS39",-0.317202295492468,9.3829596862769,-4.34881452067663,7.60225499025514e-05,0.0018655245476087,1.42237658839872

"RIN1",-0.317129748883403,12.5178162279977,-3.32714682086532,0.00174035639110737,0.0156527076322372,-1.49137505256571

"SLC35F5",-0.317019159966081,13.2696239236094,-3.6056645523389,0.000767986889722776,0.00899259861606419,-0.736901446096629

"NLRP1",-0.316878912683332,11.841118684794,-4.97026647109853,9.87403557989423e-06,0.000476109494578781,3.34739165796453

"NAA25",-0.316703867980906,7.87622977636157,-2.76029724619043,0.00828755118419433,0.045738242111205,-2.90823705788724

"EPS15L1",-0.316383897444629,10.2091626844208,-4.37310060607137,7.03024481431674e-05,0.00177382298283938,1.49589539565282

"RBM5",-0.316296090294685,10.6589762720396,-3.32644870802881,0.00174386043379027,0.0156641693964555,-1.49322184595107

"ACBD5",-0.316187126765736,8.0420780916877,-2.96982818019658,0.00473785144671013,0.0312258263458997,-2.40476612072036

"GFY",-0.316138627505335,11.2206828401048,-2.82002401201531,0.0070824116041752,0.0410258152001971,-2.76730966793804

"YY1",-0.315939938028702,10.9441587621363,-2.83988717963097,0.00671907824929627,0.0394867249676941,-2.71997720742689

"LTBR",-0.315898062797867,10.1228803677923,-2.89824030129871,0.00574937442186708,0.0355392394722806,-2.57960774036461

"SRPK1",-0.315880886922772,9.7753504856193,-4.31911592567811,8.36373342980647e-05,0.00198433457991788,1.33269176035191

"MAP1LC3C",-0.315843700511692,10.0754689736984,-3.87399311712623,0.000339857428058089,0.00516982102936323,0.0205410280087559

"MAGOH",-0.315304167065808,8.93946205480843,-2.91693749969758,0.0054673451882898,0.034331657104119,-2.53422031593765

"ZHX3",-0.315039248028253,11.7226130886052,-2.95791128730228,0.00489380024184161,0.0318743764970408,-2.43406957973121

"SLC25A25",-0.314919098170503,8.66861727275055,-3.51623305075515,0.00100200146063898,0.0107141686368026,-0.982893223109856

"PITRM1",-0.314610271308716,11.7199560553275,-2.89484066646309,0.00580208863369217,0.0357000402508048,-2.58783907015835

"MCEE",-0.314165311336973,10.5582291631788,-4.15256846723846,0.000142255627527311,0.00284019075986168,0.834420899313502

"CEP131",-0.314141358870424,10.2889980239809,-3.0333240174689,0.00398238298554263,0.0278502147100751,-2.24731842930649

"CACNB3",-0.313993623898499,12.6278063066543,-3.39701402776433,0.00142158173041173,0.0136577205023283,-1.30539057036597

"NPHP4",-0.313968087749744,8.51460540585973,-3.56011274054587,0.000879740216070021,0.00983678807050806,-0.862621863894544

"C6orf62",-0.313893206807327,9.95393849317871,-3.06384833334707,0.00366085876735365,0.0262499547456312,-2.17085322327187

"MTCH2",-0.313320942652995,8.67323245172592,-2.91810465485175,0.00545017462725249,0.0343061836407177,-2.53138050064444

"CALML3",-0.313182188537414,10.3067480755389,-4.72495452121235,2.23013346718141e-05,0.000810801349503307,2.57752981980327

"RFC5",-0.312976870238002,10.6107317925579,-4.79657119678362,1.75995943374663e-05,0.000701200764149077,2.80108816889961

"CDK3",-0.31265085200452,10.9339227164742,-6.2578714145431,1.22538857180579e-07,2.81038155910689e-05,7.51121045384358

"FADS3",-0.312319984555712,11.1247828321605,-4.88795098479103,1.29933758702845e-05,0.000584016334028942,3.08780275250754

"ATRX",-0.312003193482314,9.42935054894621,-3.3868146893508,0.00146437718615536,0.0139415878036899,-1.33268245163189

"TNR",-0.310613656184879,15.8942882180202,-4.27414152429939,9.66049942413277e-05,0.00219490311014279,1.19734457285225

"TMEM158",-0.310407396820884,6.5252945535234,-3.67565843511515,0.000622371591196211,0.00776402049854186,-0.54204774841607

"HOXA9",-0.310120620161687,10.2983122774621,-5.79335159233874,6.05472441437678e-07,7.6817705708359e-05,5.99394430136645

"C14orf132",-0.309881995374795,11.0706155825817,-3.65379969276158,0.000664731447075014,0.00813847124924813,-0.603114067652607

"UMPS",-0.309058259342319,10.1801359990541,-3.94616127376853,0.000271826491077028,0.00442666873441392,0.2288619482625

"TBL3",-0.308725049569466,10.8967105154019,-4.43149573621874,5.82149880897924e-05,0.0015778907908156,1.67331175190543

"RCN3",-0.308569690845724,13.0527564362793,-3.94856686488243,0.000269802558790154,0.00440507741234607,0.235837512890118

"EML1",-0.308434583573476,10.7767657068339,-3.01059968139437,0.00423874405480786,0.0290190939136846,-2.30391906482083

"TTF1",-0.308425017517818,9.02942713417237,-2.79256641698539,0.00761471441965008,0.0431211225872492,-2.8323587628538

"ZNF653",-0.308374966978127,10.307142153945,-3.89113352187522,0.000322346113670751,0.0049539945253059,0.0698508204411734

"ZYX",-0.307666345145176,12.004853898099,-4.16226006323989,0.000137954085548541,0.00278286799620412,0.863189840597592

"PIGBOS1",-0.307291987615057,9.2023601343352,-4.59263570161831,3.44501292972537e-05,0.00109265650384477,2.16734730007398

"KIAA0232",-0.307273794402525,10.129993260607,-4.54451470671559,4.0317130462748e-05,0.0012224290624544,2.01915524651134

"FGF3",-0.307028598363765,17.3182368881296,-5.57490659002619,1.2784042767938e-06,0.000124290076671545,5.28455541184648

"WDR26",-0.307009148780905,9.35219657413374,-2.94758899608175,0.00503274460625394,0.0325138202460371,-2.45938830076768

"AIFM2",-0.306825344402572,8.69411473767487,-2.75468061327974,0.00841013163312357,0.0460651698667935,-2.92138068909516

"PBRM1",-0.30658377959071,9.71025771966101,-3.05795134642596,0.00372100921186543,0.0265517087319748,-2.18566429698883

"FBXL5",-0.306075195332101,11.5305448595707,-3.24101802056806,0.00222706423826364,0.0186013612916722,-1.71745289251108

"NID2",-0.305643929754181,11.4001556903716,-4.3610411979606,7.30881945067128e-05,0.00181593709934804,1.45936923842769

"TIA1",-0.305447816242522,9.23584658104418,-2.91759029569067,0.00545773539788538,0.034331657104119,-2.53263208695321

"PVALB",-0.305302624247224,14.1475801525889,-3.53028449818413,0.000961182318761623,0.0104526386020223,-0.944469306266898

"PIGZ",-0.30529005813721,9.98348370582035,-3.70668227507903,0.000566678002828971,0.00732407428588126,-0.455048310526005

"NCAN",-0.305273779532094,8.87356488921568,-4.98051138378551,9.54164098391978e-06,0.00046764497414066,3.37978263769851

"FLNC",-0.305084064273725,12.2016081061013,-3.05262991280834,0.00377608421731645,0.0267950696404498,-2.19901389434101

"BET1",-0.305033125715727,10.0830116914987,-2.77303506735268,0.00801564789423063,0.0446425669924943,-2.8783590999383

"KPNA2",-0.305030122075147,12.6288446649257,-3.14485004215569,0.00292184752463948,0.0225200044671588,-1.96555577694621

"S100A3",-0.304312944868999,10.1665561010774,-5.00534717142716,8.78096290460768e-06,0.000442486325071906,3.45837857695618

"MYO18A",-0.304256489840991,11.4946758157559,-3.09971199133703,0.00331426641015725,0.0245401125046784,-2.08038058172584

"ELN",-0.304222385299648,14.897295254829,-4.42820044054892,5.88393415847725e-05,0.00158759725733031,1.66327622156336

"HMGXB3",-0.30415262125141,11.0169823801793,-3.11746265480086,0.00315437111635257,0.0237047399109816,-2.03535154923321

"BCL6",-0.304075071747279,9.45672946848604,-3.59960827844964,0.000782019273335291,0.00910278170243893,-0.75366657036404

"TCF7L1",-0.303972386284133,9.32127814391729,-3.07301757354723,0.00356914160513401,0.0258286303293861,-2.14778676211812

"SLC39A10",-0.303452736419544,8.21981274234056,-3.06545109358951,0.00364466881036551,0.0262451479088186,-2.16682448063906

"MRAS",-0.303342846268489,9.08332419278922,-3.29111942857687,0.00193019963141697,0.0168353687013253,-1.58637865626493

"STK19",-0.303300857203091,9.91421336062292,-4.61961365052928,3.15361490651879e-05,0.00103135314557065,2.25066258560493

"SMO",-0.303054866724217,12.2895705096705,-2.74801212344578,0.00855784242391795,0.0465889358251577,-2.93696124897217

"TMEM8A",-0.302730831982407,11.5028303180694,-3.8265187806195,0.00039329068274463,0.00572929725050381,-0.115476220140141

"CEP76",-0.302546744186257,8.93236639414842,-4.16544938682753,0.000136566396830452,0.00276361569332574,0.872663464942205

"RANBP3",-0.302320475867203,10.0705195686691,-3.28742778626335,0.00195072536440851,0.0169565238308571,-1.59607827896401

"SEC16A",-0.301861995533805,10.9648230089413,-3.008771414809,0.00426002762537398,0.0291202270501777,-2.30846066674782

"SFSWAP",-0.301722229153434,9.74653440318859,-3.37891200949338,0.00149837627314768,0.0141597745115366,-1.35379581528378

"OXSR1",-0.301528578443962,9.05212832378954,-2.74865547991168,0.00854348798741473,0.0465391158973392,-2.9354592452103

"CCDC144A",-0.301389128311333,9.39876296797816,-3.12595012905079,0.00308051010676769,0.0233307558424925,-2.01376299571558

"FOXK2",-0.30132581028651,10.3210565085564,-3.5648175411016,0.000867513387583054,0.009749581920321,-0.84967740525406

"SREBF1",-0.301171709342764,9.95185772450338,-3.9576024003613,0.000262330043999768,0.00432119903969783,0.262056040073236

"ESRRA",-0.30100647741018,10.1758697093531,-4.33744934152747,7.88531112108904e-05,0.00191138659410788,1.38802698822398

"CPXM1",-0.300569535420763,12.9350892726076,-2.8453330766985,0.00662252906437977,0.0390862495653875,-2.70695995937901

"CPT1C",-0.300477209893181,10.0532789655163,-3.54472002941531,0.000920906122520054,0.0101456202444399,-0.904906405594351

"C6orf120",-0.300427698169988,8.60246471094804,-3.05718841116824,0.00372885875131989,0.0265851123530249,-2.1875791590319

"TMEM200B",-0.300350745873494,10.0410148310686,-3.20822254321057,0.00244425735853756,0.0197941692561695,-1.80258345860558

"RBM4B",-0.300096305802425,8.95237249777368,-4.16145254887466,0.000138307608790628,0.00278624415952201,0.860791679576496

"ATF6B",-0.300094270122791,11.1791565434668,-3.09248923309437,0.00338148534126283,0.0249038251419724,-2.09865578341933

"CNTLN",-0.300027500674096,8.79111839105008,-3.8491220248114,0.000366910189258959,0.00544701773913155,-0.0508193483011183

"C11orf68",-0.300011070056151,10.1818647150733,-4.10914883130329,0.000163182037823522,0.00313551393622447,0.705890015124668

"FCRL1",-0.299275345597771,12.0281790311334,-3.38697455243818,0.00146369705524044,0.0139415878036899,-1.33225505213969

"SEC22A",-0.299093153465375,8.36305483277264,-3.33523168898936,0.00170026005684242,0.0154004821047363,-1.46997045340786

"IL3RA",-0.299090930250816,11.6710808249389,-3.13849225728789,0.00297434032381773,0.0227773861527292,-1.98179286666727

"FAM149B1",-0.298334165375337,8.56761935432241,-3.19773257912684,0.0025178544558312,0.0201619956850333,-1.82970019136909

"ISOC2",-0.298332635539841,10.7604538795877,-3.0210900497744,0.00411853470699503,0.0284684958939081,-2.2778248693067

"KLHL5",-0.298261436423267,7.25694870454317,-3.53687023346114,0.000942602927462838,0.0103153298353043,-0.926431150746575

"U2AF2",-0.298024494861803,9.26136923534582,-3.0785063003396,0.00351527706733812,0.0255941357173836,-2.13395782505728

"LIG1",-0.297875930791239,10.0575189409461,-4.64643118404859,2.88795689731703e-05,0.000989706148201233,2.33364555623834

"NOC3L",-0.297701413995535,8.12169104445917,-3.9221178615353,0.000292879563087609,0.00462021384838998,0.159253476759494

"PTGER2",-0.297509443078274,9.20269967120444,-3.23042124258531,0.00229516300469227,0.0190084124958056,-1.74501836573575

"GBP4",-0.297470384707974,14.0199522900903,-2.71904087401376,0.00922779718397162,0.0492577243884843,-3.00434000513719

"SF3A3",-0.297411907872622,12.1929597754772,-3.94234685009754,0.000275065894782681,0.00444841457123646,0.217805272838731

"CTBP1",-0.297339089585375,8.47425959317082,-3.459518710352,0.00118422751750857,0.0119619684137272,-1.13710192293045

"TMEM236",-0.297015420622441,7.99448982443049,-3.40461018316227,0.00139048547671473,0.0134747533547941,-1.28503334418582

"SEC24C",-0.296992680916699,13.045726647852,-5.58203550839132,1.24767171391302e-06,0.000123997773834389,5.30764567006833

"IL5",-0.296930666904396,9.98215381379596,-3.56675918446525,0.000862515026318595,0.00971632953766583,-0.844332565115923

"NPR2",-0.296814423164216,11.3139587141964,-3.94267202096933,0.000274788292703758,0.00444841457123646,0.218747631672914

"DYNC1H1",-0.296797172828875,11.5172438022641,-2.86842355056644,0.0062273050067614,0.0374580562427554,-2.65157671015042

"DYRK2",-0.296646547124258,9.08476805767303,-2.71899769366906,0.00922883085091621,0.0492577243884843,-3.00444005117137

"ECHDC3",-0.296619451493514,10.6133483828318,-4.57720833405474,3.62334978896857e-05,0.0011259783226977,2.11977857754018

"PUM1",-0.296509647658493,9.67865356609397,-3.08690794123468,0.00343430378228599,0.0251891186393251,-2.11275894850126

"ABTB2",-0.296425465113108,10.6640560385143,-4.0349724425132,0.000206042137206484,0.00365301069622081,0.487701014938414

"CDK20",-0.296330266716524,10.5597563825656,-6.95390767936207,1.11296532250918e-08,5.53051018176855e-06,9.78885977713184

"CIRBP",-0.296319536650994,10.6238186676731,-3.27475979200407,0.00202274483725501,0.0174049458362939,-1.62931275682969

"USP16",-0.296301141579562,9.55446159983976,-3.22096679861606,0.0023575792084562,0.0192931081702072,-1.76956535234468

"PHKG1",-0.296073860656357,16.6976204519862,-4.88884072322345,1.29549532305399e-05,0.000584016334028942,3.09060212772916

"AANAT",-0.296014208320333,13.6369411832387,-3.17620498591913,0.00267549715549334,0.0211684956278728,-1.88517602896855

"GPR107",-0.296003582254354,12.5401405581698,-5.77785404255431,6.38510139247647e-07,7.93215825069525e-05,5.94350239383579

"MAGI3",-0.295960001217917,9.46054673523107,-3.43533198681159,0.00127120499119214,0.0126406815383192,-1.20243227806139

"CAPZB",-0.295843805624058,10.8222095004507,-3.01982181843817,0.00413289573593609,0.0285236773997533,-2.28098268962801

"CBX7",-0.295747346665644,11.3090119513509,-2.71889648507736,0.00923125403761659,0.0492577243884843,-3.00467454042215

"ARHGEF17",-0.295286270872278,9.74521764807567,-3.31679702539196,0.00179299697637839,0.0159815261138182,-1.51873075405394

"ZBED4",-0.295236929299217,7.97831488292382,-3.63326372563848,0.000707040167327286,0.00847169561508226,-0.660308331428199

"WDR55",-0.295022069188283,10.8961022983733,-5.03876208605822,7.85116279980395e-06,0.000418004319421705,3.56428516166531

"SLC12A2",-0.294377694166409,9.43708627492774,-2.96865379036905,0.00475301105673574,0.0312738577117995,-2.40765741506011

"CITED4",-0.294281288431318,13.0142224434549,-3.11237297939978,0.00319945964258978,0.023917732363681,-2.04827959019144

"TSC1",-0.294234711057733,8.66045510322416,-3.03002759855571,0.0040186474136224,0.0280162056049459,-2.25554627258879

"UBE2E3",-0.294209466030463,11.2755137240798,-3.16428032618212,0.00276678310310872,0.0216893001452725,-1.91580449129508

"DHDDS",-0.293995923921178,10.3284056561796,-3.4126675906624,0.00135820946733728,0.0132489386959203,-1.26341111871718

"ZC3H4",-0.293294877291473,11.6551349656637,-3.13014414227821,0.00304461652303516,0.0231471079390463,-2.00308141758011

"TMEM41B",-0.293246853136389,7.73255703785592,-3.32249335717953,0.00176384040326417,0.0157687860939494,-1.50368099427954

"MXD4",-0.293128615707005,10.5269873777219,-2.94362466370223,0.00508707940709617,0.0327465863668742,-2.46909633925781

"PTK7",-0.293110378515218,11.339703878401,-5.02431041779262,8.24072552795447e-06,0.000428628120094254,3.51845909434076

"INO80D",-0.29249827706084,11.9437632028395,-2.95503528476581,0.00493214850229101,0.0320327786666306,-2.44112984414691

"NPIPB15",-0.292246859611012,15.3554161363657,-2.90990159686441,0.00557192656161058,0.0348357227654784,-2.55132315683469

"CDK1",-0.292126037027838,8.4591296742114,-4.82146091621812,1.62058240746186e-05,0.000666450544530695,2.87902405273974

"SNAPC1",-0.291995645925795,8.672633381866,-3.35435904806595,0.00160886459510381,0.0148202774159691,-1.41920818484541

"C2CD5",-0.2919445586218,10.3356316903043,-3.55108099830948,0.000903675782394153,0.0100098180007734,-0.887444771196977

"TKTL1",-0.291866651257983,12.1050690637238,-2.73443925121164,0.00886591648263309,0.0477861946242312,-2.9685909300741

"MCM3AP",-0.291851553425317,9.11770800963387,-3.86865279457858,0.000345498638521761,0.00521678292690251,0.00519956183157877

"VPS37B",-0.291521095241496,10.3225874534939,-4.25153814047175,0.000103842765199263,0.00228211698605604,1.12953866509579

"POLR1C",-0.291357716516293,11.1491584981206,-3.51423756806068,0.00100793099526945,0.0107518649817383,-0.988342962848112

"VLDLR",-0.291304504185021,10.303596657818,-4.25631715467964,0.000102269725395014,0.00226084457285572,1.14386252947075

"PLEKHN1",-0.290931329590208,14.4586164843533,-3.68777464780592,0.000600021679208952,0.00758571577340561,-0.508116176923693

"C16orf46",-0.290419022373186,7.83804154618958,-2.90746316772389,0.0056086042294254,0.0349833755439997,-2.55724395580751

"SH3GLB2",-0.290315549091275,11.1158594902208,-3.55174583309094,0.000901892819874359,0.0100011372804799,-0.885618720353207

"FOXA3",-0.290079584132672,16.117910207084,-3.10015134888172,0.0033102183337735,0.0245304456391359,-2.07926802639191

"CCDC93",-0.290074789711783,9.60683487061486,-2.79901648440401,0.00748644207379186,0.042651261865625,-2.81711793671758

"TRPC4AP",-0.289518589537739,9.93989414765028,-3.37841811977932,0.00150052584583957,0.0141726012968448,-1.35511436670781

"MOSPD3",-0.289485233795741,11.9834349660244,-2.93607327546386,0.00519209637041746,0.0332027400066047,-2.48756426505606

"PCGF3",-0.289438545739447,9.28503243041919,-5.10477725031388,6.29065333697235e-06,0.00036896228703311,3.77403913603045

"DENND4B",-0.289436979794685,10.1446708281248,-3.68351747964407,0.000607785214723599,0.00764066739718234,-0.520045110147789

"ECHDC2",-0.289419608999486,11.0356214551991,-2.84468900906876,0.00663388005477391,0.03912742509062,-2.70850036129156

"CC2D1A",-0.288798703086382,12.9049883658224,-3.17971221627857,0.0026491942671057,0.0209789447739061,-1.87615398222859

"CRELD2",-0.288784417286308,15.0567210115487,-3.87608030148541,0.000337676919995567,0.0051454024035781,0.0265398127845282

"MAP1LC3A",-0.288148844670495,10.8824643010764,-4.76505930309887,1.95343211601236e-05,0.000742623173279656,2.70259248966586

"BCS1L",-0.287897668014997,10.2324831795224,-2.95133340538276,0.0049819221585782,0.0322437067636778,-2.45021078370469

"BBS2",-0.287544796188516,11.3097191881876,-5.82371040091056,5.45603091452397e-07,7.2838012708895e-05,6.09280115451689

"PIDD1",-0.286942465360443,12.4235983715328,-3.59957401292847,0.000782099361431529,0.00910278170243893,-0.753761381537883

"POMZP3",-0.286924784553754,11.3302339890861,-4.34546529661996,7.68462198150978e-05,0.00187289104396769,1.41225037612969

"RERE",-0.286876820051457,10.3102801829096,-4.33641474287194,7.9115834483099e-05,0.00191515989589737,1.38490182142907

"SOBP",-0.286755189528359,10.1664183741399,-3.88650236392082,0.000326989566292558,0.00500814756113662,0.0565174126689136

"FAM180B",-0.286675199843586,16.740958007903,-3.65935892695262,0.000653702993933194,0.00802614472098209,-0.587601653719185

"PMS2",-0.286599561015397,11.0381127309918,-3.24145001693302,0.00222432904822653,0.0185976073328214,-1.71632796150261

"CDK19",-0.28637371027966,10.7358061901622,-3.15950955723383,0.00280412093761762,0.0218765457710605,-1.92803793219569

"NOL3",-0.286208809177385,11.1008437623869,-5.01737430148638,8.43441147529916e-06,0.00043248777369563,3.49647681496756

"NAV2",-0.285973007007875,10.5316458349801,-2.91727651038827,0.00546235265472941,0.034331657104119,-2.53339554518784

"ADHFE1",-0.285774204116565,9.44968811090591,-2.97999848077907,0.0046084461814912,0.0306356349835362,-2.37969550097334

"TTC14",-0.28562081232514,9.10105293854912,-3.33623046091315,0.00169536805573051,0.0153717380379944,-1.46732406951348

"CNOT10",-0.28562077635293,7.94366989102605,-2.77356010792541,0.00800461935339692,0.0446023356828745,-2.87712548412046

"RPRD1A",-0.285602968203623,8.68884537908071,-2.77802552371265,0.00791138843401115,0.0442463936968928,-2.8666270816494

"KDM5C",-0.285347651575544,9.8182675847199,-3.0143909202742,0.00419492581441757,0.0288287602214812,-2.29449545556445

"ZNF107",-0.285327682227319,9.53483175028771,-5.70314955604645,8.24703248810102e-07,9.22069776122745e-05,5.70058041830259

"OBSL1",-0.284952093705723,10.4113925901441,-4.24379040707359,0.000106443203204649,0.00232214934405849,1.10633091109014

"HOXD9",-0.284923384040193,10.4390233934203,-3.77327847581756,0.000462857920094038,0.00642363485846566,-0.267017633523448

"ANKRD52",-0.284851244344642,10.5230237535253,-3.72563984500199,0.000535039022395614,0.0070689165964809,-0.401698464818853

"GPS1",-0.28481563040803,11.4581403369112,-2.85583856044892,0.00643990971838717,0.0383233071085594,-2.68180034820067

"TGFB3",-0.284650873827031,10.8552624341961,-2.95528501407715,0.00492880754580875,0.0320274021747086,-2.44051696861303

"NVL",-0.284540246085399,9.22156789448828,-2.91324751443023,0.0055219626126813,0.0345997860519285,-2.54319339920401

"GNAI2",-0.284323655627707,15.7145336255402,-4.99938615743499,8.95789368652936e-06,0.000447619441783027,3.43950479540662

"ATXN2L",-0.284068581577845,12.872631519532,-3.38391498449194,0.00147676619223198,0.0140222242106358,-1.34043286419946

"ATP11A",-0.283785779237396,8.68470614326613,-3.59040896946766,0.000803805263045989,0.0092518061325473,-0.77910304221762

"PGAP1",-0.283712902606263,9.63021240884102,-3.7475736339466,0.000500552607495113,0.00676651661869165,-0.339797661260509

"SLC35B2",-0.283566222427934,10.7515762283381,-3.59552661115281,0.000791614783752798,0.00917770373723513,-0.764956915285893

"TMEM138",-0.28349037211358,9.3180661755547,-2.76516261948992,0.00818270343648229,0.0453190036455826,-2.89683621866857

"RHOBTB1",-0.283407389735952,9.54952687836219,-2.87790744993229,0.00607142112493703,0.0368424872808679,-2.62874041984907

"PJA1",-0.283331542680308,10.1262098230267,-3.04296299530458,0.00387809598514422,0.0273238515471622,-2.22322608249019

"ALG3",-0.283312927120564,9.02309645931399,-3.2666945127387,0.00206990242562877,0.0176746942682926,-1.6504314411948

"KLK12",-0.283030130290703,10.2338177074196,-3.08039415631547,0.00349692751592633,0.0255124536429062,-2.12919766180637

"NECAB3",-0.282978943642382,10.337494232553,-5.09234656418624,6.5590044081416e-06,0.000376070608516811,3.73449070712594

"SLC35B3",-0.282804085117258,10.5860549692563,-2.86191170735836,0.00633648448462128,0.037898151436105,-2.66722668943542

"CYB5R2",-0.282758300389656,11.3020540257124,-4.90617262958068,1.22284249987403e-05,0.000560908448211449,3.14516213962883

"YBX3",-0.282342627137432,14.4726667204,-2.73030071373619,0.00896186852923885,0.0481727362138082,-2.97821311269815

"SMYD3",-0.282251645044713,9.18864765397815,-2.89008953724221,0.00587651323530053,0.0360140271552899,-2.59933168371752

"MEN1",-0.282206936014568,9.98356868959624,-4.38547815763547,6.75511254510365e-05,0.0017287869573585,1.53342572396429

"MYBL1",-0.281933933486805,8.44323664488617,-4.20207217528141,0.000121573625604246,0.00253582096958122,0.981669894985254

"FGFR1OP",-0.281926085294927,9.10148765174903,-3.43431342893775,0.00127499830680868,0.0126572945119315,-1.20517771479099

"CHKB",-0.281919018044468,9.94222566983801,-4.54655914007629,4.00490552614063e-05,0.00121843120675391,2.02544011853959

"NUMBL",-0.281835987246561,10.0888778891642,-4.29259806536879,9.10619693771921e-05,0.00210466094339611,1.25281915825164

"JKAMP",-0.281416475678147,7.68797257670814,-3.06397958928405,0.00365953037726419,0.0262499547456312,-2.17052334600519

"MTAP",-0.281405672545112,9.08502842891126,-4.24231980120956,0.000106943910115209,0.00233023094768693,1.10192779426198

"ALKBH2",-0.281179661132938,17.5029814451871,-2.95688932181195,0.00490739498351092,0.0319346630993186,-2.43657891628845

"CCDC14",-0.280978312753694,9.784887309542,-3.39285330388755,0.00143889393796248,0.0137796432849094,-1.31652982070425

"SACS",-0.280160623470641,9.95785310880678,-3.02539861539712,0.00407009453681427,0.0282647209507261,-2.26709030495306

"CCDC81",-0.280022926465341,11.2390323267818,-3.67008178788106,0.000632925231266041,0.00785732093137974,-0.557645422073842

"LRIG1",-0.279978421677765,11.4932107826637,-5.45916873669056,1.89657144741562e-06,0.000157072993624158,4.91035212543374

"NAPB",-0.279112536586256,11.3495443823031,-3.8546399093843,0.000360734430579124,0.00538803526460555,-0.035006844219069

"OVCA2",-0.279079196129175,9.98663508946292,-3.42893104057066,0.00129522325406229,0.0128212449633451,-1.21967770390485

"IFFO2",-0.278970497416907,9.41499077792497,-4.07468623545004,0.000181892591003572,0.00336840223650404,0.604297540386613

"AGO2",-0.278722686196888,10.4139167271021,-4.03951606959309,0.000203129019520073,0.00361929783883923,0.501014569279032

"ZNF254",-0.278398782990804,11.9646463079254,-3.94897629480863,0.000269459542594585,0.00440507741234607,0.237024949163068

"SAP130",-0.27837368611214,9.55581532175938,-4.0501338602333,0.0001964764088816,0.00354668665840862,0.532152885466746

"EGR4",-0.278264256512095,10.9167774252045,-3.63326611650777,0.000707035094377865,0.00847169561508226,-0.660301682703436

"TBC1D21",-0.278160748728199,8.89591286881582,-3.21948086847336,0.00236753378271666,0.0193384178385661,-1.77341930119086

"CCDC78",-0.277940809606161,17.7993666740555,-5.56633628392104,1.31634698605985e-06,0.000125256017200131,5.25680257354464

"KDM4B",-0.277785684535365,9.65285647925517,-3.78715809053218,0.000443656997609541,0.00622965465481717,-0.227614189969455

"POM121",-0.277708444309994,12.1434164092163,-2.82965326111371,0.00690405751126096,0.0402564161730598,-2.74439259013493

"PHF21B",-0.277644347566198,12.8538598392187,-3.83332134839658,0.000385164593009563,0.00563846923432738,-0.0960372214406799

"ARPC4-TTLL3",-0.277517311972703,10.397116456079,-4.2312710502281,0.000110779838627534,0.00239051934041973,1.06886712396231

"USP53",-0.277445017560298,10.9944455908018,-2.89592537194957,0.00578522066132097,0.0356300025647161,-2.58521345172189

"SPTAN1",-0.277105246415996,11.627267374374,-2.71402585161744,0.00934856477533295,0.0496647293400528,-3.01595191071508

"NDC1",-0.27708019801007,9.75141547666054,-3.28504360465533,0.00196409135460643,0.0170478555276829,-1.60233911126905

"PEX1",-0.276874465213544,11.3422175507386,-2.92222570344364,0.00538994932693836,0.0341071112520694,-2.52134740622658

"THEM5",-0.276849106189449,11.451064557813,-4.05593343443639,0.000192932441690653,0.00350392735980112,0.54917664026952

"ATXN3",-0.276705370152133,9.20006256130054,-3.60237051594679,0.000775588949990684,0.00905868697679643,-0.746021972029395

"RAB34",-0.276527946647372,11.697585587674,-4.45680976267475,5.3630078770265e-05,0.00148727139817299,1.75049633155343

"SH2B3",-0.276349124996663,6.81390215393353,-2.89645375349578,0.00577702048925103,0.0356300025647161,-2.58393421878276

"HAPLN3",-0.276093925054735,10.837982999214,-3.4141916939492,0.00135218509422883,0.0132146875681676,-1.25931782091721

"ANAPC7",-0.275927010381897,10.0248327318844,-4.27256817446125,9.70924505219224e-05,0.00219581143790982,1.19262006466775

"TNRC18",-0.275902632239809,11.7060891398129,-3.95785623328013,0.000262123041104279,0.00432119903969783,0.262792997971595

"PPARD",-0.275595043924117,8.7102424841844,-2.83127768346949,0.00687438376899066,0.0401788319679405,-2.74052122330755

"METRN",-0.275560045497391,11.7397890111291,-3.74828666294846,0.000499468228623937,0.00676380555779986,-0.337782240036892

"HMMR",-0.275160593707675,8.22056856533203,-2.81130169930179,0.00724764609302748,0.0416221961342436,-2.78802148363553

"ABHD1",-0.275045769991454,9.92695663094505,-3.30662784799109,0.00184618853302976,0.0162932741328907,-1.54555934157606

"ASIC3",-0.275023089430951,9.67084327418928,-3.82723595667608,0.000392426231596386,0.00572201577738227,-0.113427628348121

"MED6",-0.274899906950891,9.34294848666793,-3.24282844964278,0.00221562272756334,0.0185819385716739,-1.71273786814988

"IL9R",-0.274761204954167,10.8296070835169,-3.2084943673584,0.00244237755114851,0.0197879039911665,-1.8018800563155

"TBCD",-0.274695594048783,11.2050735638736,-2.83939918661861,0.00672779327358884,0.0395184078502495,-2.7211428100413

"PHYKPL",-0.274529408750103,10.9809459486826,-2.75970146218087,0.00830047517980797,0.0457869875089685,-2.90963216292162

"PRKCI",-0.27435799014274,8.73659831643845,-2.7817407460971,0.00783458704880802,0.0440095183572605,-2.85788338988937

"AQP11",-0.274310597118467,7.05717678787748,-2.78650043840696,0.00773720233076651,0.0435802306344717,-2.84666959717988

"HIST1H2BE",-0.274242463816456,8.8387057474692,-3.02187856674093,0.00410962939694793,0.0284494782626249,-2.27586107066335

"KRT7",-0.274215816851303,16.3770991489167,-3.46948941280092,0.00115005079464203,0.0117405617385156,-1.11009406337311

"MAP3K10",-0.27420949512242,16.3784643996307,-3.96881807976533,0.000253332900413309,0.00420467315823001,0.294639911693667

"D2HGDH",-0.274099311705386,11.7292311492717,-3.84725972243783,0.000369017472475578,0.0054737591750544,-0.0561535981232346

"PSMC6",-0.27315051399915,9.52458215745335,-3.727650373228,0.000531785045369951,0.00704152677766325,-0.396032223219902

"ZSCAN5A",-0.272982982999228,16.1401367866582,-3.4037700919674,0.00139389241713245,0.0135004555766553,-1.28728603760863

"VWA2",-0.272773439487963,10.5089025064997,-2.80761555019941,0.00731854441624287,0.0419351188539938,-2.79676112261157

"NCOA5",-0.272745423775868,9.36905238941691,-2.91863677065257,0.00544236312290522,0.0342690721244814,-2.53008555040532

"PDZD2",-0.272632006992978,12.5270298790571,-4.81086213292418,1.67855310470824e-05,0.000679358291631802,2.84582182711691

"RFC4",-0.27251363203035,10.0413121038688,-5.29439490797193,3.31792813509861e-06,0.000247310068369913,4.3800511244778

"NXT1",-0.272386719324651,9.75327835261938,-3.78024779419488,0.000453118645038707,0.00632280767636304,-0.247241288129512

"DBR1",-0.272283201665255,8.75453923134927,-3.12980800274806,0.0030474787175725,0.0231590258193095,-2.00393785434741

"NRM",-0.272189429460516,11.0340315179126,-4.61712943806929,3.17940408441373e-05,0.00103520307685282,2.24298373011663

"GMPPA",-0.271737159314322,12.2622207995363,-3.42576112579147,0.00130727727849318,0.0128967931625536,-1.22821122266433

"PDK2",-0.271691067600322,7.67908597246538,-2.89427021434673,0.00581097795170484,0.0357102660865846,-2.58921962788869

"WDR75",-0.271469483399082,9.82966404999057,-3.20524420626476,0.00246494390138047,0.0198634527878212,-1.81028811269383

"CUEDC1",-0.271266781462744,8.69801581604674,-2.78900630071742,0.00768638300750417,0.0434033161683213,-2.84076041500634

"ANKRA2",-0.270841423593152,8.50298580017724,-2.82328990634595,0.00702144689969327,0.040741700807205,-2.75954308842935

"ARHGEF11",-0.270538750634651,10.458908064481,-3.77421154487903,0.000461542403805455,0.00641533182725391,-0.264371002240903

"CDCA5",-0.270427310734386,10.1917171592568,-4.25724415835592,0.000101967297495034,0.002257540822882,1.14664174956568

"DIDO1",-0.269948870391964,9.42283359963008,-2.88536002138068,0.00595148119817415,0.0363189046053153,-2.61075924960851

"ZNF638",-0.269901521938559,9.5366698348732,-2.78124380957247,0.00784481964374743,0.0440477020109848,-2.85905339341944

"PIGG",-0.269829038907893,9.16777537292701,-3.69887966592818,0.000580216167120394,0.00742984038197332,-0.476965018637057

"POFUT1",-0.269737673577328,8.18877394228625,-3.04904752205002,0.00381359067997797,0.026983516606991,-2.20799233634749

"ATXN7L3",-0.269413028527024,11.0409298495178,-3.93287659013549,0.000283270867651968,0.00452041518718828,0.1903762408823

"GTPBP2",-0.269386382791936,10.0874157167037,-4.71221598662043,2.32582767359543e-05,0.000829189658191591,2.53787582881298

"KLHL41",-0.26920582864361,8.60546555964133,-4.58047996358173,3.58478981587911e-05,0.00111916762681085,2.12986170009917

"GSS",-0.268927386454566,10.5762174533659,-2.81896240527391,0.00710233418105045,0.0410512620887921,-2.76983291759505

"GPN1",-0.268857985873648,10.6713473267124,-3.80267540233146,0.000423099459419995,0.00604056363093719,-0.183475029988304

"PRKRIP1",-0.268721213333318,11.1810490068762,-3.40085343041042,0.00140578258215418,0.0135715297421242,-1.29510451677572

"RHOXF1",-0.268719730097239,10.1023232119212,-3.16748146567871,0.00274199325178314,0.0215479040400066,-1.90758946964917

"TTBK1",-0.268613210328624,11.2017398058907,-3.06509051634447,0.00364830523485404,0.0262485907084687,-2.16773095655734

"CMPK1",-0.268605503441417,9.27190867771199,-3.11688292114881,0.00315947652182118,0.0237179502722866,-2.03682478279707

"ABCB9",-0.268575436574606,9.98250320923971,-4.39564044393366,6.53711092439139e-05,0.00169792317186794,1.56426956719413

"FAM71D",-0.268500396066827,9.83928743336224,-3.10187628238619,0.00329437037420274,0.0244535234954825,-2.07489911734231

"FBRS",-0.268378181267614,10.5959717455756,-4.62925538939949,3.05545150999593e-05,0.00101490314436997,2.28047899461588

"C12orf49",-0.268368539146669,9.11775546205287,-3.39819711416749,0.00141669539510356,0.0136254107112944,-1.30222171274842

"GDF1",-0.268282721572378,11.0046279875844,-5.10918682336925,6.19809024505742e-06,0.000365932793378984,3.78807389786409

"PI4KA",-0.267904607822693,11.329567704342,-2.87725818822758,0.00608197599112451,0.0368815147475344,-2.63030542071238

"DUSP8",-0.267901624167923,11.5746276895616,-3.74456503136991,0.000505153120362556,0.00679961186618944,-0.34829949880239

"NXF1",-0.267834453224673,11.0279501879645,-2.83497195276363,0.00680733969804263,0.0398619674853864,-2.73171122177298

"AXIN2",-0.267532523247189,9.79900025197422,-5.19886597225559,4.58229626079541e-06,0.000305943627613589,4.07412306721649

"POC1B",-0.267388672838385,9.09682925530329,-2.83094880555936,0.00688038189485004,0.0401788319679405,-2.74130513804515

"FGF19",-0.267169527868122,9.62518199662585,-3.9040918806682,0.000309689505172403,0.00481331371228353,0.107199550266723

"RBM25",-0.267149172515772,11.8948956796159,-3.31543580154612,0.00180003179768989,0.0160218465238384,-1.52232483198484

"FAM83G",-0.266383920274093,10.3519954677379,-3.36364807914571,0.00156618261993223,0.0145703927144278,-1.39449401143773

"RABEP2",-0.266119222511644,10.2253163015392,-3.50230003572434,0.00104410973548138,0.0109806461246481,-1.02090860662706

"CATSPER1",-0.265908470644753,9.1897284101765,-4.0055262938449,0.00022592747316115,0.00388616977632674,0.401585210093737

"ZC3H7A",-0.2656827335203,9.9806874852993,-4.32702569178884,8.15394567336844e-05,0.00195530742829609,1.35655419427247

"ANAPC15",-0.265144354073085,11.7103335454658,-4.7858041578807,1.82384589780503e-05,0.000715863679325133,2.76741168169729

"N4BP2L2",-0.264998105698167,11.2907669063275,-3.11723653309509,0.00315636152518332,0.0237047399109816,-2.03592619610872

"KIAA1324L",-0.264957688299837,8.99790919361821,-2.84874318387717,0.00656272963703673,0.0388358155729243,-2.69880008051744

"BRD2",-0.26475693889506,11.0754284945555,-2.92077912544617,0.00541101867779458,0.0341565124276207,-2.52487033000482

"SNAPC3",-0.264686535818268,7.72036104112673,-3.14899823948471,0.00288807016467669,0.0223559875274346,-1.95495053619605

"C11orf49",-0.264675303188472,10.6129625938304,-2.93513974179279,0.00520521861850951,0.0332542831051467,-2.4898451326733

"BOD1L1",-0.264634013258053,8.89077811538596,-2.74329776991712,0.0086637095459189,0.0469936628462533,-2.94795996248188

"TUSC1",-0.264615893446331,10.8865671421621,-3.6210784589276,0.000733353661067714,0.00868805539260949,-0.69416360348401

"APEX1",-0.264516024719429,10.3264855204865,-3.70607456782043,0.000567721421331656,0.00732407428588126,-0.456756161276735

"OR2T5",-0.264150024013288,9.55187850506702,-2.88978043444334,0.00588138587332385,0.0360216212461276,-2.6000789343548

"ACSF3",-0.264095724392455,10.6671190152093,-3.65980391343401,0.000652827862242642,0.00802090496405125,-0.586359428132669

"NANOGNB",-0.264005274184429,9.80203171439678,-3.65178062907953,0.000668780934187051,0.00814974259650691,-0.608744933008398

"NUF2",-0.263780361797011,9.01602803687801,-3.86352602725016,0.000350999293281781,0.00528537572181632,-0.00951868118501586

"PCDHGA2",-0.263439553946382,13.3896915647982,-3.4857159757186,0.0010964353188873,0.0113180215923687,-1.06604650715252

"FAM181B",-0.263367995979531,10.7863949368559,-3.54518887696324,0.000919625469055057,0.0101442590363685,-0.903619953061201

"LIMK1",-0.263194482294297,10.2955212957369,-2.85329150807921,0.0064837509750976,0.0384985391173415,-2.68790614832513

"XPO6",-0.26311707315627,11.3459693987042,-3.4140945740055,0.0013525682227802,0.0132146875681676,-1.2595786884111

"OR5V1",0.263210101988946,11.0677232769923,2.76777790892906,0.00812685251841746,0.045065488128323,-2.89070207210897

"ZNF543",0.26365164171378,10.9865740154127,3.24364683729982,0.0022104689780186,0.018547410669688,-1.71060595533701

"SOX15",0.26385734297571,9.33224311349095,2.927914193989,0.00530783647326405,0.0336350997769113,-2.50748253400188

"SERPINB10",0.263876928303072,9.3493838744575,2.95332752141234,0.00495505225296845,0.0321046467777445,-2.44532003948268

"RNASE11",0.263913088331579,9.3009139822642,3.78331573278996,0.000448894232459034,0.006293314204122,-0.238529755548234

"MYCL",0.264066507459214,10.6799199986473,3.54862756658825,0.000910284667990797,0.010076783679262,-0.894181790263852

"AQP7",0.26429973204948,11.2790118596196,3.27691208121029,0.00201033359963059,0.0173231492118457,-1.62367171008731

"PRSS23",0.264506655811681,13.839376410698,2.92087067058101,0.00540968305345312,0.0341565124276207,-2.5246474204508

"HIST1H4B",0.26473777572766,11.5344674201459,2.72895393458593,0.00899329962361119,0.048312653743778,-2.98134216867147

"PGPEP1L",0.26492405911787,11.0430061192678,3.03227418072903,0.00399389896811261,0.0279089291564713,-2.24993945080617

"TRIM36",0.264967320792994,8.64876361197601,4.16722884253355,0.000135798059091713,0.00275117947801999,0.877950544627502

"PLEKHD1",0.265170675599423,10.5127893232006,2.71046395576104,0.00943522215354647,0.0499368902676902,-3.02418992288187

"INSL4",0.265930263778321,10.23504810799,3.16596010165841,0.00275374855676143,0.0216155366089975,-1.9114943668868

"RIPPLY2",0.265947684714219,6.35885248568719,2.88143734726169,0.00601433415752973,0.0365953822258671,-2.62022762569714

"CCDC65",0.26595947913299,10.0757421127126,4.28675496788685,9.27823871416666e-05,0.00213614430318825,1.23524616933276

"DCD",0.265978238625181,11.3203469843053,3.59325985477612,0.000796992289451145,0.00921615712087364,-0.771224009326298

"FBXO39",0.266149701150898,9.73690759650519,2.80538556823508,0.00736174670790452,0.042128690613469,-2.80204440107762

"WNT8A",0.266151366356199,10.2524065444007,3.46098214429762,0.00117915129007046,0.0119174222757461,-1.13314066034106

"HIST2H2BE",0.266217344460372,11.6898001004125,2.90022412183687,0.00571882005419607,0.0354483617288682,-2.57480140415417

"GABRP",0.266455150819338,11.633065785335,4.26040222625282,0.000100943530284327,0.00224599354882627,1.15611171508218

"OR4C13",0.26679288359527,9.02826338536598,3.93938225550233,0.000277609351208539,0.00446998531392399,0.209215452174227

"CD209",0.266911838156881,12.3999274097123,3.63977924012028,0.000693344423224473,0.00838056647774499,-0.642180764127406

"KIR2DL4",0.267047357505506,11.7631885528789,3.52899550990359,0.000964859516166291,0.0104798857830594,-0.947997623538736

"CYP4A22",0.267092625454742,11.5080318018157,4.04109272135057,0.00020212752727175,0.00360506836400268,0.505636001883743

"SLC36A2",0.267280403402539,8.71814468949883,3.50756753765846,0.00102799525302954,0.0108879852465633,-1.00654649737734

"DCAF8L2",0.267397504803597,9.72599769325128,3.31490228755432,0.0018027961445463,0.0160289364959189,-1.52373324311735

"ANP32D",0.267500929484614,11.5739375836409,4.0429195563596,0.000200973107752599,0.00359880673131757,0.510991787230772

"HHEX",0.267676488124374,6.25315356480357,2.98570006837389,0.00453735383412817,0.0303321086467559,-2.3656157358743

"ARHGAP25",0.267763647637411,12.0713593118581,3.75371339523454,0.000491289018897855,0.00670379043406844,-0.32243680411461

"ANKRD18A",0.267817539673546,10.7857458485708,2.99758715055288,0.0043924225799306,0.0296625320998031,-2.33620393688693

"OR8D1",0.267893927302358,10.3711395913737,4.56759037614591,3.73907855592954e-05,0.00115324786701765,2.09015062097626

"STAB2",0.268114579622216,10.2168377053565,4.43256585450936,5.80136317691784e-05,0.00157481920898154,1.67657130504201

"PGA3",0.268498491744149,10.6299022701814,2.92798561841705,0.00530681292546655,0.0336350997769113,-2.50730833178939

"ARHGAP6",0.268548811218382,8.91480218912867,4.73511594041196,2.15658249163856e-05,0.000800396352550251,2.60918579114051

"SIGLECL1",0.268557627661098,9.88869974692384,3.69319684069036,0.000590271492656193,0.00751556351041042,-0.492912282329529

"PLS1",0.268811488631446,6.40986836888917,3.08510145962168,0.00345156487983321,0.0252846208580411,-2.11732017159684

"OR1C1",0.269000737191403,9.48225942807009,4.39795716786065,6.48838322130086e-05,0.00169750741323706,1.57130494816827

"RPE65",0.26902451016513,5.75581459924241,3.59445905013653,0.000794143049499087,0.00919509709546224,-0.767908759606672

"SGPP2",0.269139836541394,9.05647233943948,4.3704361094958,7.09088836616846e-05,0.00178158570199983,1.48782166299584

"SLC5A7",0.269254608797825,9.92964193333477,3.29069312619422,0.00193255939389876,0.0168477363535355,-1.5874990821704

"KRT33B",0.269498961396227,11.6314754389249,2.93288815400193,0.00523699558984289,0.0333634665622149,-2.4953443454737

"TCEAL5",0.269547995783451,10.9455759787372,3.27296793024933,0.00203313301894067,0.0174775187774289,-1.63400742468236

"SKIDA1",0.269591162687396,7.96700800117325,3.44264572291917,0.00124428148772929,0.0124490780391439,-1.18270498063063

"TRIM61",0.270052681816898,10.609312000418,4.36793107578175,7.14836839428247e-05,0.00179099666954229,1.48023286094708

"KIR3DL2",0.270510863333879,12.4751402694249,3.5026121201594,0.00104314830923193,0.0109806461246481,-1.02005803052187

"EBLN2",0.270626871851693,12.1183934385212,3.4988287109096,0.00105486095748127,0.0110741769868958,-1.03036671804005

"SLC1A3",0.27084397003096,9.84395089812747,3.37306162940215,0.00152402832855524,0.0143039573817024,-1.36940747485652

"FAM161B",0.270924901464008,11.0064506086425,4.37348380074806,7.02156513244815e-05,0.00177382298283938,1.49705667652413

"FAM86C1",0.271201038242992,11.7997986233706,4.0728344908237,0.000182954833708683,0.0033741020826955,0.598849557257673

"KIF2B",0.271415300859325,5.96756860430173,3.13766086548763,0.00298127001840252,0.0228109235924734,-1.98391461265147

"SOX17",0.271685504458926,9.70490442602945,2.77943349199466,0.00788220120019339,0.0441882473426072,-2.86331442456792

"GABRG3",0.271890962714114,10.3496332215724,4.45115320683402,5.46229176822054e-05,0.00150151868608766,1.73323482167623

"KRTAP20-3",0.272195646270578,11.1338955271452,4.00190339770347,0.000228498975057642,0.00391909699406152,0.391010012439525

"NCR1",0.272321666221753,11.7086118929267,4.21685790425597,0.000115985774986468,0.00246129244215057,1.0257932577169

"CABLES1",0.27247138369415,12.3438136055296,3.16640435749363,0.00275031096630246,0.0215980302353752,-1.91035421364011

"KDR",0.272596097474088,10.3190590147111,3.39617571497789,0.0014250538045948,0.0136756810355908,-1.30763557402685

"RCL1",0.273019224640294,12.3432501110368,2.8077945675922,0.00731508645138041,0.0419287348698315,-2.79633686745323

"TRIM50",0.273144318658685,9.46906889494232,3.86653605560232,0.000347759580551702,0.00524100348482679,-0.000878461135931374

"UNCX",0.273174219229661,9.71999326183422,3.0060322095301,0.00429210320589191,0.0292500701905526,-2.31526173429602

"ZNF772",0.273756038075916,9.54038198972663,3.33106142385845,0.00172083133566647,0.0155235337006793,-1.48101504926906

"HSPA1L",0.273890764168463,11.7237212285984,3.32682791808531,0.00174195623172977,0.0156592236328713,-1.49221870926472

"PSG1",0.27407910879594,10.4884322907557,3.3408386636514,0.00167296966298917,0.0152614759312663,-1.4551079012877

"SPZ1",0.274082475608218,10.0242944990068,4.58815966222329,3.49584925992796e-05,0.00110001546614552,2.15354020871835

"PROKR2",0.274150133639846,11.5003715657614,4.05474234966098,0.000193655183287844,0.00351348638320105,0.545679497802381

"POMP",0.274266996778591,10.0825522828752,2.94490540954465,0.00506946601061022,0.0326684717088639,-2.46596094795561

"LCN6",0.274376456723711,8.53956729170499,3.64282855418134,0.000687022515978591,0.00832665703817142,-0.633690949968401

"CLEC6A",0.27447647591363,8.61144016008603,2.98926179661515,0.00449346519810694,0.0301174967886605,-2.35681118872075

"C6",0.274548061457342,10.044489773812,3.01734417491507,0.00416108724305307,0.0286739944880494,-2.28714936335797

"GJA8",0.274603966800502,8.94149858422833,3.27059022485042,0.00204699565511855,0.0175629281891682,-1.64023459044161

"KRT23",0.274683332052346,9.90710420014472,4.33462480835559,7.95723849949623e-05,0.00192360864212822,1.37949573930291

"APIP",0.274805591936012,9.38513059842395,4.32134667776796,8.30404059151338e-05,0.00197804237205836,1.33941980827906

"PRAMEF20",0.275266667050991,9.53856190129895,2.98401722188443,0.00455822957452719,0.030392161333849,-2.36977329148512

"TMEM64",0.275678027190947,8.79152228952771,5.27736346749327,3.51479634834367e-06,0.000253533031756129,4.3254215084883

"ODF4",0.275694507127417,12.0947787430667,3.20127953905662,0.00249273797250026,0.0200326098787319,-1.82053741194494

"PNPLA1",0.275945521737869,9.72413358376302,3.536006409683,0.000945020185679559,0.0103300680858881,-0.928798205415345

"PSRC1",0.276337387603471,10.5462150651761,5.04822134513729,7.60607742305956e-06,0.000407380595871594,3.59429858040742

"BCAS1",0.276348360172264,11.7537700913022,3.1089689742123,0.00322995289958232,0.0240953408759917,-2.05691841459824

"WFDC1",0.276573361301244,9.85218366261685,3.42289879554577,0.00131825346612304,0.0129430495364846,-1.23591279945178

"FUNDC2",0.276585995484631,9.87742143636355,2.92135492278378,0.00540262302809504,0.0341565124276207,-2.52346820296727

"NCKAP1L",0.276625476308974,9.33667069946472,3.13681723327585,0.00298831728643667,0.0228408584051752,-1.986067232621

"GABRA6",0.276630242548283,8.36067486524367,4.2554830372103,0.000102542596922941,0.0022615954302825,1.1413620009057

"OR1L8",0.276678667288273,9.67817721106638,4.69180500066371,2.48763196065288e-05,0.000879383508568151,2.4744096543361

"SIGLEC8",0.276699175425323,10.4783223808761,4.87684362869751,1.34825889743053e-05,0.000598486437124934,3.05286804216757

"OR6N2",0.276829814085167,9.01171099444828,3.14160426316749,0.00294853610007927,0.0226476437502439,-1.97384774060571

"OR5M11",0.277007456932628,8.80179291216172,3.81466801191586,0.000407843879226457,0.00589429261227009,-0.149299899895659

"PET117",0.277050244492097,8.77177513177674,2.81473814114195,0.00718212447638981,0.0414054220941468,-2.77986670420633

"MUSK",0.277157703906347,10.8621277277291,4.47395185464123,5.07279002399563e-05,0.00142908883053949,1.80285645807085

"RCSD1",0.277422263665624,9.68468248546605,4.34558725452069,7.68160746098173e-05,0.00187289104396769,1.41261905606606

"PRTG",0.277629214422834,7.68207646091665,2.71724484770964,0.00927088133532545,0.0493738601392191,-3.00850032477187

"CYP4X1",0.278339752207161,10.9291126815616,4.00560931146909,0.000225868877912635,0.00388616977632674,0.401827589431859

"INMT",0.27839111853489,10.9693273190346,4.13368298230985,0.000151015669142638,0.00296544380383387,0.778443753500001

"CXorf65",0.278439996003399,9.77040901653164,3.07413131665682,0.00355814937600509,0.0257908161212946,-2.14498195655161

"ASPA",0.278761353186013,8.37061761479369,2.77811365058219,0.00790955863155023,0.0442463936968928,-2.86641977196533

"TGM4",0.278861761282662,6.98538808553824,3.73468498185805,0.000520548972414324,0.00692349484573966,-0.376194267251837

"GZMK",0.278937243859369,10.5888648250284,3.51442741024,0.00100736544259148,0.0107518649817383,-0.987824571069702

"OR52L1",0.278939216542355,11.4492668658275,4.52084453668266,4.35517897685366e-05,0.00129203642979992,1.94646285138006

"CCR2",0.279085009300262,9.38490033644082,4.29423088511444,9.0586796582798e-05,0.00209910259594517,1.25773155441518

"ELAVL4",0.27975711761319,11.1125247490532,4.21170454914478,0.000117904344292659,0.00248676297043818,1.01040730336845

"KRT4",0.280035441493162,10.3316777063132,3.7171843585084,0.000548934889111591,0.00718296967943351,-0.425511104557491

"KRTAP19-7",0.280138685077761,12.9034735209873,3.27214596859136,0.00203791517546735,0.0175067178366522,-1.63616043768887

"NBPF6",0.2802136045214,11.5212113585598,3.69548558575788,0.000586201745980043,0.00748505569867022,-0.486491100930194

"IQCJ",0.280258047077353,11.2536825136158,3.05668753617826,0.00373402050671559,0.0265851123530249,-2.18883611740041

"ANGPT2",0.280373520090997,11.8109718520982,3.46696138602471,0.00115862595653773,0.0117817081539558,-1.11694597145641

"TCHH",0.280438604246571,9.75260182355663,4.74227899199532,2.10617240025603e-05,0.00078658284067182,2.63151379033456

"HPR",0.280461100098671,13.8580286995245,2.87171517174668,0.00617278473133544,0.0372931259908341,-2.64365665481746

"IRF5",0.28046135194997,9.67534792525451,2.77852066153238,0.00790111282971269,0.0442463936968928,-2.86546225937858

"SLC18A1",0.280591994356456,9.70258643756878,3.93259215716954,0.000283520925709947,0.00452041518718828,0.189552912940185

"DOK2",0.280609315026908,11.7226926186801,3.49469250187489,0.0010678095531454,0.0111447170923092,-1.04162950794003

"TAAR8",0.280655642049826,10.9844632927572,3.52056888350628,0.000989232371076975,0.010640348366991,-0.971045938012856

"MS4A12",0.281124383631639,8.76255850669941,3.35727857707001,0.00159533196330838,0.0147330600997128,-1.41144490441903

"RGS6",0.281389514476178,10.1299983530801,4.56017513937873,3.83076907122407e-05,0.00117544816320973,2.06732292682747

"ERVW-1",0.281403768906882,11.2957565532085,3.74595575679675,0.00050302149800537,0.00678623799232132,-0.34436996307481

"CXorf56",0.281481293896459,11.1556815875368,4.62383805556225,3.11023079255543e-05,0.00102315676499534,2.26372368703657

"CBLN2",0.281527938374312,10.8111186292083,3.96000066702097,0.000260380537311868,0.00429699947598894,0.269019860251355

"CTSG",0.281638892895526,12.7662671005878,3.86826499902424,0.000345911787543786,0.00521755140587756,0.00408592062342095

"OR51I1",0.281644814862515,9.85458388383304,5.00034295877715,8.92925945774708e-06,0.000447438438206268,3.44253382470993

"CEACAM18",0.281825433076428,9.72573142829943,3.27010553524753,0.00204983248864475,0.0175788367158993,-1.64150364602828

"ALDH1A3",0.281828342290691,9.45927432711613,3.07664093283451,0.00353349688355603,0.0256725401099127,-2.13865943564899

"ZNF85",0.28206173133748,15.8995977394324,3.13185501334974,0.00303008782865838,0.0230660600710084,-1.99872145114037

"OTC",0.282116851121732,9.34790610743791,2.77808260193426,0.00791020325850267,0.0442463936968928,-2.8664928113439

"SLC46A2",0.282171541795886,8.67878177366335,2.84551858589325,0.00661926301428567,0.0390798666873123,-2.70651623667631

"CTAGE15",0.282219186751171,9.90285704925301,4.2320178759672,0.000110516384418743,0.00238771449380059,1.07110068928756

"FAM216B",0.282506005034621,9.69827005806963,3.63804636182111,0.000696961907484809,0.00839910704543005,-0.647003701679215

"TYW1",0.28266938975038,12.0342534936949,4.38803589109788,6.69958426285655e-05,0.00171949588060604,1.54118619324833

"SPDEF",0.282762098912858,11.8324688550923,3.28011533203032,0.00199199553469219,0.017214883149811,-1.61527198281049

"OTOP1",0.283017138534197,10.8307727653603,4.03745475173904,0.000204445635220979,0.00363551487919294,0.494973734070132

"TSGA13",0.283221540660428,9.45841981955383,4.31570617875223,8.4557850194401e-05,0.00199295834272416,1.32241048357554

"TMEM97",0.283271245108438,9.86994052364795,3.67279985095791,0.000627760078006943,0.00780945760463574,-0.55004466303104

"TRIM43",0.283399154350144,10.5746589578394,3.51636068689588,0.00100162332948961,0.0107141686368026,-0.982544585046377

"TXNRD1",0.283667240372784,10.814923429957,3.53188759088554,0.000956627750984777,0.0104157722686346,-0.940080198314715

"TNFRSF1A",0.283780072245698,17.6975897914221,4.55351481143584,3.91500112026817e-05,0.00119514428396719,2.04683026802736

"OR6C4",0.283829808698138,6.08429350078341,3.12621207575563,0.00307825667248081,0.0233307558424925,-2.01309611977497

"OR5D16",0.283858407697911,11.210262657266,3.7064670900744,0.000567047259145887,0.00732407428588126,-0.455653065542625

"PSENEN",0.283887722436685,12.6956098399083,3.94356075045191,0.000274030956706251,0.00444491494650308,0.221323394795453

"LAT2",0.284291544930392,14.4853925900091,3.37400771986778,0.0015198518953747,0.0142797429392637,-1.36688392197812

"RAB19",0.284476503044306,10.6058095244813,3.80150636622967,0.000424615664963369,0.00605254950639817,-0.186803526487798

"XKRX",0.284602049102791,7.12878467255767,2.76290724715443,0.00823115283713146,0.0455450334375022,-2.90212290307614

"BMP10",0.284696060029944,11.555707041675,3.57173144914504,0.000849840384658893,0.00961593588941362,-0.830637929634042

"HOXB1",0.284708688771977,10.2850048866547,3.6391303152945,0.000694696975869724,0.008388464033433,-0.643986992026943

"RASSF9",0.28524761610568,8.06485608024218,3.24191557759757,0.00222138490502505,0.0185974072543704,-1.71511552513458

"NFE2",0.285384103309147,8.95126532009142,3.54693253044547,0.000914877631828617,0.0101151087489383,-0.898834784141719

"SLC5A11",0.285387201071547,11.3706464855322,3.55586157731703,0.000890929880718898,0.00990543482671247,-0.874310122567485

"NPHS1",0.285420402130315,10.4233180581056,3.6908032903259,0.000594556650129452,0.00753674627958642,-0.499625270504148

"BHMT2",0.285431457456784,11.2039735105934,2.91972033041037,0.00542648863698417,0.034205234399933,-2.52744812024308

"RNF152",0.285473081439703,9.58649690386171,4.18814877535937,0.000127074659578715,0.00261893846221616,0.940179640954491

"TRIM64",0.285955755003126,9.71526587679282,4.11628983699608,0.000159545861525727,0.00307610137040478,0.72698826786938

"FMO4",0.286507897624487,9.77891216329655,3.72175200960627,0.000541385788730715,0.00712121351073806,-0.412651005577401

"APOC3",0.286540468817698,10.2452488972977,2.83917265456004,0.00673184245523977,0.0395184078502495,-2.72168384940773

"SLC25A33",0.286634169626897,10.9359995594283,4.04978920506909,0.000196688995254198,0.00354694499607092,0.531141549812302

"BSPRY",0.286642623584664,10.7835726539366,3.50558619321093,0.00103402854643323,0.0109268456796504,-1.01195015437299

"S1PR5",0.286687966247275,10.5507634594498,4.3925315556085,6.6030625176512e-05,0.00170906428924127,1.55483078626141

"TNP2",0.286940744414764,12.5479674961835,3.10590333125494,0.00325764896886559,0.0242513867682216,-2.06469335814827

"C1QTNF9",0.286991610179481,10.3866467669025,3.69429215754775,0.000588320470487632,0.00749605761862767,-0.489839579499385

"CFAP52",0.287208565397814,10.9184202874776,4.45636376141483,5.37077155357901e-05,0.00148727139817299,1.74913502185423

"IL20RB",0.287218012931165,9.73225438606882,4.07902599468008,0.000179426518553751,0.00334698747696355,0.617069799539971

"C20orf202",0.287347732028985,8.22736413556417,2.84942269847034,0.00655087380158735,0.0387940418642433,-2.69717329909276

"RAB44",0.287462368200524,10.750507703002,4.20759037873159,0.000119458179627823,0.00250408505893403,0.998129624604749

"SERPIND1",0.28775964607618,10.8979654674688,2.89981353601509,0.0057251313669951,0.0354506317148409,-2.57579634145115

"MAS1L",0.288591356743455,9.66835092316523,3.6697578845774,0.000633543462480229,0.00785954160909072,-0.558550983156175

"NR5A2",0.288795189119837,8.97223461066572,3.30881764720308,0.00183460943821797,0.0161990761304449,-1.53978633144946

"RXFP1",0.289493618470273,9.19349089862529,2.8330482510065,0.0068421751842435,0.0400260535876167,-2.73629980901838

"KRBA2",0.289586470112697,13.4849418255056,2.9767942918007,0.00464885472594891,0.0308355069308491,-2.38760023362372

"NELL1",0.289632307346844,11.0374746287494,3.2587067946493,0.0021176318057436,0.0179537039682215,-1.67131579030308

"CELA1",0.289743677657071,8.78687843953118,3.79782797633896,0.000429420682986228,0.00610644403651879,-0.19727333778446

"PGM5",0.289947353202074,10.251877960052,3.37499035520417,0.00151552566593835,0.0142465783699271,-1.36426245116493

"HTR5A",0.290126565466919,10.2137673974553,3.53005799128759,0.000961827520401819,0.0104533004328482,-0.945089370187358

"AGTRAP",0.290189797818501,12.4191434321801,3.12544669787188,0.00308484530891644,0.0233343425230874,-2.01504455415106

"PCDHB11",0.290401666325552,12.5937518651075,2.99935272151636,0.00437126947745319,0.0296212949835779,-2.33182885778262

"TYR",0.290495592318607,7.76277492246265,4.38836043729471,6.69257034301374e-05,0.00171949588060604,1.54217102951474

"C3",0.290501395454905,11.9655492224159,3.90173635579142,0.000311953877336139,0.00484001987134969,0.100405975315836

"GRIA4",0.290544587706295,14.366815449233,2.89726196065027,0.00576449852705156,0.035595828149957,-2.58197721000804

"ADCY10",0.290550327995206,10.6672058575366,3.32409798802778,0.00175570874423522,0.0157478317482237,-1.49943875899125

"SDE2",0.290890502551724,8.93885480388204,3.24762870866439,0.0021855548793065,0.0183814721372422,-1.70022841470571

"ST6GALNAC3",0.291176809389347,8.39294262920259,3.14779556779684,0.00289782502820389,0.0224024165641916,-1.95802618382576

"KRTAP19-8",0.291266145059007,11.0068533676095,3.09982875384964,0.00331319014985148,0.0245401125046784,-2.08008492178719

"HESX1",0.291367728818427,9.71008202383434,3.85671520503735,0.000358437780655262,0.00536172978496169,-0.0290568252033276

"TRIM39-RPP21",0.291656182668026,12.3593078846859,3.49729413432665,0.00105964741829556,0.0110983797809656,-1.0345462053899

"IFITM10",0.291791389009305,10.6562312252322,3.25808795697281,0.00212137276001379,0.017976900665034,-1.67293247612721

"SAMD7",0.291987321173773,7.14331493493874,2.95434055101876,0.0049414539989971,0.0320579337239447,-2.44283464934543

"KRT20",0.292458249196882,8.52281967173963,3.40116501605773,0.00140450776818484,0.0135665439876126,-1.29426945820341

"N6AMT1",0.292497438214616,10.9179809676767,4.01926002096968,0.000216431704443148,0.00377363232045173,0.441713792553737

"GREB1L",0.292781810552519,10.5863033539921,5.07159442167369,7.03240752623229e-06,0.000389589702976946,3.66852029217309

"CDC20B",0.292815314386845,10.6955831144068,3.62250079831624,0.000730234431999596,0.00867984302594072,-0.690214958617949

"CYP8B1",0.292924642102419,9.8507125490495,3.30499848853218,0.00185484912605441,0.0163616351163645,-1.54985336406139

"SBK1",0.29299445479635,6.41142428371845,3.25960368805736,0.00211222105664011,0.0179248209118762,-1.66897236388213

"NMUR1",0.293214952230473,12.1577322708961,3.88205716372331,0.000331507328939179,0.00506866205760083,0.0437266342904792

"FAM3B",0.293246765763676,8.958792011137,2.84502449220687,0.00662796527728493,0.0391054323368569,-2.7076980238839

"DIO1",0.29325779246542,9.85329530707875,3.96876383560103,0.000253375686990439,0.00420467315823001,0.294482217082418

"PRSS12",0.293539064359667,8.79428293804517,3.52819152056827,0.00096715992870412,0.010492130967003,-0.950198001435257

"FNBP1L",0.293542000411586,8.66861987447789,3.12081676777984,0.00312498536171717,0.0235678175108594,-2.02682456709883

"OR2W1",0.293671805234657,9.53969983706452,3.42877511967459,0.00129581367844642,0.0128212449633451,-1.2200975547074

"HTR1F",0.293699883406306,9.92389905282599,3.64486728579693,0.000682826673991325,0.00829829237162419,-0.628012653766071

"OR5D13",0.293868636877736,8.68641322977793,3.09833095599136,0.00332702109938886,0.0246143038165536,-2.08387702579386

"CLPSL1",0.293898019114046,11.9504643876566,3.08274136243436,0.00347423871893955,0.0253987153425049,-2.12327664037353

"NUDT10",0.293921951641147,9.97078401046628,3.47740296297364,0.00112359765092133,0.0115451110725628,-1.08862701654522

"KIFC1",0.294229977212298,9.10804872644876,2.76974325047887,0.00808511376257284,0.044917577670393,-2.88608969001521

"OR1J2",0.294476667491201,9.01501594213239,4.72114709048889,2.25832281226822e-05,0.000814498725577947,2.56567403959434

"HHLA1",0.294640466012659,8.68500142391433,3.49326458227846,0.00107231487810838,0.0111591860700878,-1.04551595328849

"ANKRD34A",0.295025397998092,10.1319396147723,2.94954168309015,0.00500618090276296,0.0323889831074873,-2.45460325393869

"DEFB136",0.295036162749462,10.8150848712622,4.14045121451882,0.00014781755339985,0.00292511970439151,0.798492280226402

"OR52K1",0.295089068490698,10.0971339536613,3.72644641482252,0.000533731319630454,0.00705824515260758,-0.399425511045014

"C1orf50",0.295117922626568,10.9645722184903,3.84126145917419,0.000375884511537639,0.00553887811111764,-0.0733259517757432

"HLA-DQB2",0.295129265423464,12.651291623197,2.91497855817359,0.00549627749007202,0.0344871652121706,-2.53898490779073

"A1CF",0.295424427538254,11.1558884085665,5.37254819301738,2.54573906445021e-06,0.000200619938872026,4.63118891736511

"DNAJC17",0.29544990819404,12.0230080037857,3.75607216481638,0.000487774219439043,0.00668131164743111,-0.315763253813984

"MYH11",0.295480393622135,10.3372967964264,3.05519576935713,0.00374943364398169,0.0266482393552596,-2.19257895156482

"NOSTRIN",0.295728278280684,10.9727072926672,3.67612545572762,0.000621495482269371,0.0077639194708916,-0.540740942247994

"IL22",0.295895519410632,9.42343147743423,3.59023926952463,0.000804212562810166,0.0092518061325473,-0.779571937077106

"NCF1",0.295970771133703,11.9575882890722,3.14241453374364,0.00294185217474923,0.0226157256356205,-1.97177825659523

"UTP6",0.295989350080653,13.4552951878971,2.75223885079956,0.00846394257025137,0.04627489872837,-2.92708882868182

"CASR",0.29612865632267,6.93162108824314,3.20722659017242,0.00245115658965832,0.0198052123904235,-1.80516038140815

"NPS",0.296139103898744,10.0838233553957,3.11723311024611,0.00315639166347801,0.0237047399109816,-2.03593489444796

"CHST7",0.296407867928982,9.46951125808582,3.95417081372878,0.000265144111550856,0.00435395210664288,0.252095234415759

"SEMG2",0.296455070025315,9.05073527910215,3.34903729973931,0.00163381322454859,0.0150191596988437,-1.43334889598982

"CDRT1",0.296507447961146,10.1764618676498,4.36132886825766,7.30205039458056e-05,0.00181593709934804,1.46024009253213

"RNASE2",0.297043655532294,10.3277719788777,5.09912270269045,6.41135067436712e-06,0.000372378740953745,3.75604618518281

"ALDH8A1",0.297079195585775,12.6326508904042,2.74580478647362,0.00860726152945441,0.0467725703221172,-2.94211268901349

"PIP4K2C",0.297239526923565,10.1129928891501,3.43492503890277,0.00127271925508784,0.0126487081968146,-1.2035292279645

"PPM1H",0.297245725693074,8.82539982388156,2.92126640669257,0.00540391288267267,0.0341565124276207,-2.52368376108658

"KRTAP23-1",0.297346394149603,11.8939773378477,4.40885741828886,6.26384124902991e-05,0.00166499043245017,1.60442558110544

"OR11G2",0.297756596087487,9.78949428816741,4.31263284250482,8.53960119054478e-05,0.00200216154256429,1.31314634634181

"DEFB119",0.297812234909808,10.0461315082988,3.81761281814222,0.000404179778241313,0.00585455226960231,-0.140899911992955

"XDH",0.298078937158094,10.4775153707777,4.17355708610729,0.000133099554900047,0.00271186553258193,0.896760712419908

"LAD1",0.29814257344198,8.4869649200262,3.82101740705801,0.00039998316378781,0.00580787241639621,-0.131184376306322

"TIFAB",0.298213605835871,9.43610844230773,3.3032141263722,0.00186437782937425,0.0164213958590231,-1.55455442227565

"WT1",0.298375252245711,9.68337798044283,6.18866886823784,1.55537314800235e-07,3.31238931483501e-05,7.28471229379793

"BTN1A1",0.298381108269606,8.44390279755649,2.72880063672667,0.00899688372298555,0.0483173980547849,-2.98169826560349

"COL28A1",0.29858006601973,10.5223053803145,4.52383452425719,4.31295747677601e-05,0.00128805503008424,1.95563781283584

"HMGN5",0.29871812804091,11.3763951548671,3.64361141033317,0.000685408428456805,0.0083240131545579,-0.631510731523235

"CNGA3",0.299059255984387,9.80994446019885,3.78951268431462,0.000440476780675554,0.00621426587500393,-0.220922375239931

"PRR11",0.29939188635117,11.5447627162103,4.77134567885937,1.91323558747096e-05,0.000732888039063553,2.72222570501445

"LTB4R",0.299907062391641,12.7930619776188,3.77352455287514,0.000462510630410715,0.00642363485846566,-0.266319673242213

"PRKACG",0.300088037830294,11.0297458577743,3.68092494927044,0.000612560251908778,0.00768449533407863,-0.527306043278847

"LHX9",0.300263070317127,12.2644571642659,3.97997136985389,0.000244682479262919,0.00410418252680633,0.32708532760593

"MYZAP",0.300414510950059,9.62296223049324,3.69915461627279,0.000579733863959729,0.00742984038197332,-0.476193121996235

"MAP9",0.300463727769797,8.37115042756941,3.63752677190597,0.00069805010697908,0.00839910704543005,-0.64844958217666

"GAB3",0.300745299493954,10.1369060390689,3.29960891765669,0.00188377196011489,0.0165433463890502,-1.56404800048505

"CD1E",0.302165035825645,9.87034262131962,3.86192076246727,0.000352738948607916,0.00530193781629397,-0.0141252115274852

"MFSD9",0.302524329986964,9.74699287965405,6.06686444577698,2.36584608928364e-07,4.31863476440766e-05,6.88633548088037

"CXCL3",0.302555874292921,10.2235317449701,2.92834191445311,0.00530170977459381,0.0336350997769113,-2.50643929281841

"NAT10",0.302605917251809,10.4925004011033,3.92280808331661,0.000292253771047478,0.00461865048636788,0.161248923721514

"ZNF597",0.302737461031128,7.18928945576234,2.89876754438487,0.00574123913845819,0.0355012191316552,-2.57833057077507

"ZNF705E",0.303007154744321,11.0538586294455,4.24533651613173,0.000105919252348869,0.00231920380081876,1.11096077037543

"OR1L6",0.30327583123856,12.0655482694769,4.26462924687464,9.95889010069783e-05,0.00222415212248918,1.16879160456221

"OAS1",0.303291849422033,11.0851254566872,3.84022346524982,0.000377085299426077,0.00555129786954178,-0.0762962687800623

"SLC45A2",0.3035501163286,10.0324580535233,6.02400544553902,2.74176877544814e-07,4.80857859058743e-05,6.7462733326934

"CPLX3",0.303637849282745,6.19392388353123,2.71371754177086,0.00935603655184627,0.0496647293400528,-3.0166652800615

"SMU1",0.303957883522759,10.9610394287294,3.7901434064272,0.000439628638369678,0.00620719551049342,-0.219129490791389

"RAB9B",0.30398530491866,9.92669695050206,2.86148891494353,0.0063436343054895,0.0379282333191516,-2.66824194754708

"UGT3A2",0.304124750598772,10.5668531251248,3.96120565034652,0.000259406330277909,0.00428487520068468,0.272519505593635

"CWF19L1",0.304205541792236,9.96085994384258,3.89900275620399,0.000314601762592803,0.00487153128928795,0.0925244716845475

"CXCL9",0.304404301772909,10.9159603702217,3.06439088096263,0.00365537078590109,0.0262499547456312,-2.16948961329706

"SRR",0.305433936547649,11.6914924706945,4.02928741543752,0.000209744163468094,0.00370030901408356,0.471052583614815

"KRT76",0.305518219718854,8.88627855845474,3.80584447996358,0.000419015471408574,0.00600622417309934,-0.174449404286702

"ZCWPW2",0.305602411752623,7.46707195568444,3.23450911858969,0.00226866265572222,0.0188675528815503,-1.7343911183054

"KRTAP19-2",0.305990433511388,13.4599242377211,3.07720261248637,0.00352800140966963,0.0256474009004046,-2.13724393004989

"ASRGL1",0.306903207127034,11.019568918115,3.93607696645726,0.000280471937613482,0.00449987667441039,0.199642094360023

"FETUB",0.307234486555712,10.1687749032294,4.44909108587856,5.49893148279379e-05,0.0015041343317385,1.72694409089812

"DCC",0.30737111441498,10.1668174383339,4.54356523443803,4.04422265862235e-05,0.00122414719357183,2.01623677597363

"AKR1D1",0.30760912648311,6.75966861097397,2.99920217038769,0.00437306948692655,0.0296212949835779,-2.33220198931788

"CELA2B",0.307651772632031,10.811491733668,4.1956186600908,0.000124093916593356,0.00256934730779925,0.962431866407263

"HERC5",0.30796039647577,8.05416379402144,3.26580336815602,0.00207517637623178,0.0176869761746326,-1.65276293299739

"TNFRSF19",0.30857985767361,14.7045612526065,5.51031050858959,1.59345449605181e-06,0.000139885255627801,5.07554382039453

"PGBD4",0.308879863965114,11.4160687436762,3.86858651314658,0.000345569219541588,0.00521678292690251,0.0050092160892552

"PCSK1",0.309276815455304,9.11756301967647,3.11333490993702,0.00319089184641627,0.0238636556189551,-2.04583726942862

"MRO",0.309562789950286,10.3958209724071,4.2053533526509,0.000120311406026201,0.00251725233029557,0.991455914551475

"RUFY4",0.309602184971627,13.1438155562775,3.20691168788717,0.00245334182829202,0.0198108601095749,-1.80597505443637

"SLC22A5",0.310182532423163,9.64869857968896,3.57365541830548,0.000844984083530261,0.00958523796466254,-0.825336118161811

"ZC3H12B",0.310241628413179,8.41217485932927,3.17352870082947,0.00269573387783384,0.0212719619302062,-1.89205634728678

"CCR8",0.310489836501732,9.68271645609647,4.49910218032573,4.67461419497653e-05,0.00134877698925702,1.87981061236145

"EIF4B",0.310853467961143,12.5840059791478,3.92454320188418,0.000290686326799387,0.00460592356077435,0.166265931769391

"SGMS2",0.311191728545076,11.3906856200761,3.13160759406448,0.00303218486277925,0.0230722054488549,-1.99935206546639

"PACRG",0.311535354338426,10.571277136923,3.18436255035913,0.00261469480656127,0.0207516749754102,-1.86418180180856

"PHB",0.311672140522861,12.2440341137095,4.2560125745042,0.000102369283023821,0.00226084457285572,1.14294943160325

"PNLDC1",0.311976567324038,9.41390739823449,2.93146061308292,0.00525723634058236,0.0334447727228584,-2.49882945953548

"PRAMEF11",0.312045775606251,11.4384869942818,4.82281737079637,1.61330687905512e-05,0.000664987252521131,2.8832749176042

"OR9Q2",0.312181824101112,11.9514354423058,3.42262603321141,0.00131930400003366,0.01294625850609,-1.23664651796074

"DCT",0.312518587458133,10.3122710249894,3.13305239965794,0.00301995851751078,0.0230245159744503,-1.99566914466765

"OR52B2",0.312629715669567,11.1346738136335,3.53750056246647,0.000940842797286683,0.0103066361302275,-0.924703717219487

"SERPINB12",0.312890505279066,9.66265247480507,3.70237338007968,0.000574116274428633,0.00738344071405738,-0.46715452327216

"KCTD14",0.312946670414421,10.0649046213275,3.99993548227848,0.000229907650877921,0.00392819290024368,0.385267538631757

"RBP7",0.313001226293762,9.71971265106414,2.89470781430993,0.00580415772823689,0.0357000402508048,-2.5881606037066

"IRX6",0.313224535083316,10.2468540321732,3.32619544377073,0.00174513330019206,0.0156641693964555,-1.49389177612055

"NCAPH",0.313242348625009,14.8520876866464,2.8886252625761,0.00589962909826251,0.0360938662581457,-2.60287106052565

"REG1B",0.313246524167855,9.61274307958337,3.91014133204991,0.000303946726680895,0.00476117205254618,0.124655824008829

"TM4SF19",0.313567371709109,8.92791203367624,2.77071413579635,0.00806456790358915,0.0448452145562034,-2.88381030825595

"FRMD3",0.313962892056812,8.80350925660028,4.73217346694475,2.17763211032532e-05,0.000803209501476487,2.60001686484661

"CNDP1",0.314294783131293,10.5206870466849,5.76204993739141,6.7404825605799e-07,8.25893784426122e-05,5.89207892578239

"MTMR12",0.3143153870018,12.3567248197073,3.15690051818609,0.00282474077269717,0.021951254423449,-1.93472324563682

"RSPH10B",0.314527956693352,9.12300687048369,3.92630266651034,0.000289105194523063,0.00458899984456351,0.171354416415097

"LDHAL6A",0.314935177652561,18.3603039913976,5.253764716741,3.80681475329022e-06,0.000269170391785015,4.24978797811984

"TCTE3",0.315412498750334,10.9035054481129,5.20820264699228,4.44017672904002e-06,0.000299770314295777,4.10396979070347

"RPS26",0.315870406691735,12.67139867793,3.0046046519821,0.00430890915876936,0.0293310791252759,-2.31880454447765

"KLHL15",0.316232211931853,10.4434978777178,2.99768309412951,0.0043912706330983,0.0296625320998031,-2.33596623272403

"TSPAN16",0.31661851028772,10.587393087526,3.44764076259042,0.00122620708741211,0.0123075431398374,-1.16921805655415

"OR10A4",0.31663348447298,9.061781383263,4.93162051496472,1.12338649387244e-05,0.000530244353268708,3.22536895945589

"OCLM",0.316678429830016,9.93879768320916,5.35907044858018,2.66486846993882e-06,0.000206371567353834,4.5878277515231

"LELP1",0.316722387041787,11.7693235985879,3.81890621088554,0.000402580518352183,0.00583611255494505,-0.137209518726055

"HMGB3",0.317301353544677,11.7709795573796,3.89142760965005,0.000322053407147885,0.00495375184907009,0.070697778099059

"TRIM77",0.31730689797368,10.8441995663595,4.36814111601389,7.14353142345601e-05,0.00179099666954229,1.48086909643464

"LRRTM3",0.317854151402219,7.98798003694227,3.21176151705594,0.00241989013920528,0.0196412952360451,-1.79342272902308

"TAS2R46",0.318694741496799,10.8929028259908,3.28374710674249,0.00197139606713165,0.0170863877155611,-1.60574254607869

"OR2T8",0.319341096584642,11.6420929716253,4.92363592438099,1.15370170165699e-05,0.000540276694789056,3.2001906305853

"SALL2",0.319406535192774,9.67991801600682,5.02879346000212,8.11788183723815e-06,0.000424622187679396,3.5326711462203

"IGSF5",0.319409091543255,10.2376922567271,3.55963764292049,0.000880984030268953,0.00983678807050806,-0.863928491362718

"CCDC126",0.319596944801043,8.92151339402263,3.46896682664879,0.00115181844437878,0.0117405617385156,-1.11151070222177

"MYOM3",0.3196127366504,10.2037076128201,3.06169822747561,0.00368268371919729,0.0263623565637136,-2.17625564522528

"SPRY1",0.319688041009599,9.48749492938001,3.65407679320659,0.000664177527835783,0.00813799438044817,-0.6023411469675

"OR4F29",0.319917576750518,11.0387754604754,3.40273318993054,0.00139810847440788,0.013519331080369,-1.29006603095666

"APOL4",0.320244214218789,11.1574464849186,4.40518618641961,6.33860592731774e-05,0.0016798714286487,1.59326698114884

"HAVCR1",0.320483158868099,11.0242558003875,3.94352124893577,0.000274064575213422,0.00444491494650308,0.221208903559685

"PCSK5",0.320740604060941,11.4027405797866,3.52499424326111,0.000976360144782752,0.0105511452056832,-0.95894562555877

"NPR3",0.320787719332689,9.15764450951058,3.49404307883731,0.00106985634480373,0.0111530653567564,-1.04339718842768

"NBPF14",0.321057158021993,12.3774788184401,3.22264055907266,0.00234641372207216,0.0192457565676978,-1.76522292702568

"ANKRD22",0.32112917039794,9.43397431613806,3.28342983984944,0.00197318752685731,0.0170936327689833,-1.60657527914684

"IFIT1B",0.321259009016126,9.53010826526101,2.75589297440586,0.00838353150698263,0.0459783109685539,-2.91854520930121

"ACPP",0.32154041539127,8.71526440918337,4.79975964907154,1.74146678198384e-05,0.000695381679975646,2.81106521956648

"ADAM2",0.322165450610743,7.7523081795063,2.80158652244291,0.00743589167266928,0.0424420055340899,-2.81103837293782

"LRFN5",0.322406612161119,10.2870433231459,3.19143837336641,0.00256301411672171,0.0204535317019984,-1.84594430716929

"KCNU1",0.322621123696319,8.43764007282543,3.05291589557212,0.00377310504260091,0.0267845540107491,-2.19829684864503

"BSPH1",0.322961530703743,10.5263920047814,3.80779779263973,0.000416517204093683,0.00598480021207381,-0.168884435609147

"HSD3B1",0.323236671770434,11.6931590809612,4.32519946847403,8.20192116001756e-05,0.00196155304320259,1.35104324709304

"LIN7A",0.323946863028477,9.06582088288603,3.74740908562229,0.000500803175311518,0.00676651661869165,-0.340262739499733

"PRAMEF15",0.323948693120125,10.0558815417958,5.07827378369092,6.87646679848956e-06,0.000385621048771723,3.68974650069783

"CACNG5",0.324144938083773,10.0407605643854,4.44474716076456,5.57690256578264e-05,0.00151849634702109,1.71369601013296

"CES1",0.324454339823578,9.38112127418758,3.80876637356735,0.000415283732534429,0.00597187354606785,-0.166124427672611

"NCR3LG1",0.324605991474572,10.1880345980974,3.68706524126286,0.000601308728477741,0.00759125747617382,-0.510104494508265

"KRTAP20-2",0.324621233306063,13.6531859691057,2.98232643808337,0.00457929424213242,0.0304871584285474,-2.37394889138197

"CIDEC",0.325795230561784,10.1192558512309,3.14174372772328,0.00294738463759802,0.0226476437502439,-1.973491563073

"PLCL1",0.326261805517337,6.48975409091124,3.20555975458273,0.00246274439603427,0.0198629551400618,-1.80947203156501

"FKBP1B",0.3263933212071,12.4334922311703,3.58297773699057,0.000821827582561929,0.00940576061112906,-0.799624829607636

"FCER1G",0.32654381556058,12.5030735455842,4.89836989984796,1.25503970262309e-05,0.000571282576087135,3.12059275745733

"GOLGA6L6",0.326575277255479,13.2726658574592,2.78328712158052,0.00780282404685069,0.0438669765474896,-2.85424161590828

"DRD5",0.327141633264583,11.5716205089388,3.87094379420948,0.000343067565880704,0.00519337721125055,0.0117797894166367

"IDO1",0.327166978800099,11.8438784119867,5.20816977876589,4.44066931010011e-06,0.000299770314295777,4.10386469923821

"HIST1H4C",0.327185063935133,12.5898029622277,2.75489896596338,0.00840533505949308,0.0460529981253512,-2.92087006913193

"MMP26",0.32727417274987,9.34755358970782,3.7507339850272,0.000495763504563284,0.00672891755169392,-0.330863235681991

"CXXC4",0.327308009760412,8.71407138226837,3.34333743559962,0.00166094211300089,0.0151827253242069,-1.44847956562617

"SOHLH2",0.327593520761202,7.36816630029802,3.03816019410403,0.00392973447399767,0.0276007145682545,-2.23523678139015

"CCR4",0.327626744521764,10.4248051381512,3.22474363890385,0.00233245517767329,0.0191721451565316,-1.75976469707762

"TLX2",0.327866740058745,12.0506391881343,3.2489689477603,0.00217722912506698,0.0183286832086227,-1.69673373444706

"HOXA11",0.328366591949591,12.4858089486723,3.25872068722885,0.00211754789504271,0.0179537039682215,-1.67127949440913

"RGS4",0.328653236945057,11.9165130636367,3.56952504201994,0.000855442494893684,0.00966099166108151,-0.836716118439202

"NLGN4X",0.328755094551038,9.59867450714017,3.1859794327591,0.00260279933826146,0.0206810726378448,-1.86001661414946

"OR6K2",0.328819202022869,9.58683072464822,4.22769059435484,0.000112051419793563,0.00240924020274886,1.05816117009771

"ERP27",0.328832453161068,7.23799528556593,3.47698224584437,0.00112498926489944,0.0115474590493643,-1.08976899210808

"GIMAP8",0.328938068998763,9.58854688167518,3.88817617729746,0.000325303897911591,0.00499087601178426,0.0613355433952245

"PGA4",0.329232163057014,12.2751707500173,3.16738680146174,0.00274272332154368,0.0215479040400066,-1.90783247941858

"ZSCAN4",0.32927449066155,8.79427986495663,4.28870278526486,9.22053870719431e-05,0.00212559557903349,1.24110311364287

"LSM3",0.329331394324017,10.4942731524794,3.28763739644495,0.001949554399665,0.0169553728742584,-1.59552771383482

"DNAH8",0.329431432450342,8.38153523331842,4.51257711364519,4.47404120370982e-05,0.00130992018155753,1.9211050584268

"SP7",0.329662541337331,11.6159300246767,4.11843750988792,0.000158467759180868,0.0030679430681546,0.733336757881952

"RHBDL2",0.329677889858155,9.85084010247342,3.38698426738728,0.00146365573308186,0.0139415878036899,-1.33222907850709

"CXCR1",0.329730696797448,11.4104070517559,3.22463833671172,0.00233315221676836,0.0191721451565316,-1.76003804543484

"GPR174",0.329771553741535,9.24115364831479,3.26327039426999,0.00209023656588362,0.0177953088537809,-1.65938781013156

"MRPS10",0.330224665237996,11.6397453953003,3.34690068302495,0.00164393288003443,0.0150811873286851,-1.43902247763547

"GML",0.330344545667698,10.8904030294959,4.73536331356241,2.1548219942223e-05,0.000800396352550251,2.60995670276918

"TLR3",0.330376960314057,9.22135725102514,2.80303591319551,0.0074075230540299,0.0423500095600962,-2.80760804513654

"ACCSL",0.330618230800502,10.5626177321093,3.12257749677366,0.00310966278100896,0.0235017986858764,-2.02234602276868

"AQP8",0.330630051056207,12.2445232820759,3.36764536837801,0.00154814887212528,0.0144683603328538,-1.3838465200817

"MYH15",0.331086073944364,11.1973906614559,4.19023752737821,0.000126234228340307,0.00260762599397199,0.946400184564786

"ZNF705G",0.331168312446353,10.5210248397685,3.85381355158784,0.000361652878955251,0.0053913402930254,-0.0373756322927434

"TMEM132D",0.331185277796989,9.35040873324739,2.88422313231405,0.00596963435569708,0.0363852773386934,-2.61350432579498

"NFIA",0.331470839042799,10.8250650631671,2.90004252148271,0.00572161072113571,0.0354506317148409,-2.57524147246277

"BMP3",0.331801314165578,8.04241119336048,3.35646653118382,0.00159908506386073,0.0147529823142881,-1.41360460536814

"S100P",0.331850536216306,9.6410928082787,2.84952712664142,0.00654905355506888,0.0387940418642433,-2.69692327076437

"XRCC6",0.331949795927041,13.0641196810419,2.8158475727384,0.00716108881528812,0.0413107764645886,-2.77723251158911

"SYT17",0.332224914359662,10.2732664420517,4.07848981402139,0.000179729437077586,0.00334914572904265,0.615491448240459

"DCAF12L1",0.332398433650045,8.44397425606805,3.73771966685121,0.000515772667237621,0.00688199670872586,-0.367630346483982

"MRGPRX3",0.332815864930081,10.7529889547512,3.83036112607324,0.000388680650384291,0.00568528876101765,-0.104498450437177

"CDH17",0.333639538323714,9.32145410153659,3.68489669113515,0.000605259520050404,0.00761426691573957,-0.516181239521331

"RAG2",0.333960465334281,9.84341537794692,5.48416885559798,1.74185373742246e-06,0.000148931211000221,4.99107152431723

"ZNF658",0.334374408480517,10.1909143229118,4.60821732305208,3.27363642467596e-05,0.00106090728262732,2.21544734750592

"ATP1A2",0.334406111804842,9.28007844718829,3.5595006136841,0.00088134308800844,0.00983678807050806,-0.864305335364079

"MKRN1",0.334694433373738,12.1631663373979,3.60603287263995,0.000767141325083839,0.00899259861606419,-0.735881360511218

"ZNF502",0.334802289481559,12.7749319137771,3.34159249213748,0.00166933250730121,0.0152360659301589,-1.45310857763615

"KRTAP20-1",0.33496909821325,13.5434331520666,3.47624007861481,0.00112744815830348,0.0115581204033759,-1.09178330630017

"PSG2",0.335022285197516,11.6769544569073,3.46594220065639,0.00116210030395509,0.0117984178986678,-1.11970754538223

"ZSCAN12",0.335084776159176,11.1324046796354,4.09911306071821,0.000168428606560991,0.00320533972635061,0.67626639795852

"RPS24",0.335160667588067,12.2177662478069,3.18622707376062,0.00260098195247767,0.0206810726378448,-1.85937855927488

"OR4K17",0.335331710956991,10.2209456682822,4.28120570318063,9.44456130885742e-05,0.00215777467757536,1.21856584084672

"BCO2",0.336110371243244,11.9388902765715,3.70473412568049,0.000570029463874662,0.00734223285837485,-0.460522714077953

"TYSND1",0.336504543812628,10.1273793134873,4.57596785850974,3.63807630677663e-05,0.0011259783226977,2.11595609907903

"MARS2",0.336760690390305,8.27620236308618,3.4279779436138,0.00129883634946389,0.0128440483446984,-1.22224395199717

"ASCL4",0.33693343295411,9.65040880360688,3.4378826379704,0.0012617530028331,0.0125676500376845,-1.1955551592246

"ORAI1",0.337022012981159,9.35908054481785,3.23111554111813,0.00229064154697798,0.0189885480231182,-1.74321398139973

"DSG1",0.337082519293977,9.85579832493298,3.94601534130928,0.000271949742250595,0.00442666873441392,0.22843884820263

"GOLGA6A",0.337113527767389,12.3397482058358,3.56519376927962,0.000866542704083678,0.00974942291405843,-0.848641871627171

"PRAMEF2",0.33746709837518,10.1286991094677,5.15232856012618,5.36057712595847e-06,0.000336281587536502,3.92553787258242

"CHN1",0.337930792259884,9.74038384692093,4.59210443989312,3.45100857886016e-05,0.00109265650384477,2.16570829263336

"ADCK2",0.337933356395697,9.71669508766029,2.96972976921877,0.00473912004746591,0.0312258263458997,-2.40500843329772

"BEND4",0.338061697044786,9.67390198472152,3.9080776911225,0.000305894083206274,0.00477499062345292,0.118699529519821

"PRSS58",0.338798404455506,9.4586693726781,3.24098128261864,0.00222729699182525,0.0186013612916722,-1.71754855494886

"SCGB1D1",0.338891611726654,8.76527226856486,3.75489706059716,0.000489522202917444,0.00669500205503835,-0.319088186148135

"APOC1",0.339356069837418,9.22783933678398,2.71324870935425,0.00936740908124483,0.0497103473908006,-3.01774995677071

"COL14A1",0.339483807699981,9.93302489682952,3.46921130620687,0.00115099116871763,0.0117405617385156,-1.11084797637933

"CT55",0.339782503216062,10.6365772287101,3.42632448521336,0.00130512724555802,0.0128848903398385,-1.22669497256099

"NCMAP",0.339984845848566,10.6445972676619,3.9059412069593,0.000307922886843541,0.00479828616963772,0.112534579739523

"NMS",0.340127428170149,10.1362124885985,5.86646140304684,4.71154453244132e-07,6.65670540423426e-05,6.23210428319379

"KCNS1",0.34024522404855,11.917376731265,3.78034529269582,0.00045298380654508,0.00632280767636304,-0.24696449232458

"PRSS3",0.340335294230753,12.3137327061638,3.98795618251768,0.00023866597710302,0.00403180021295423,0.35033956700141

"TLR7",0.340336735499285,10.3050953875183,3.5633946533862,0.000871193917332236,0.00978329440499459,-0.853593230364642

"HMGB1",0.340397595417377,13.1411362977098,2.74968663105328,0.00852052752737749,0.0464423269156782,-2.933051363202

"NDUFV3",0.341155676205432,13.1819429001191,5.70730640930674,8.13051855859822e-07,9.21928878869697e-05,5.71408713444974

"ALDH1A1",0.341237889743399,10.8095754662317,3.589332379667,0.000806392559719426,0.00927092320104166,-0.782077543572993

"PRAMEF10",0.341248740452951,10.3833624613317,4.7305583798788,2.18927167773933e-05,0.000805841173725901,2.59498491974715

"NTRK2",0.341276867131562,10.5169792786435,6.27323718896891,1.16218080157691e-07,2.75696120162003e-05,7.56151394741936

"NXF2",0.341441794292933,9.70950626730088,2.83281296289896,0.00684644725189413,0.0400379519088375,-2.73686089167985

"C6orf201",0.342070724243033,9.17475149494786,3.53412730674202,0.000950299020883969,0.0103657921857276,-0.933946224698078

"EPYC",0.342151477750834,7.29558136288287,3.13295643778103,0.00302076913142349,0.0230245159744503,-1.99591379230503

"HNMT",0.342155436218201,9.4207598613368,4.26512265977676,9.94319370666277e-05,0.00222415212248918,1.17027204163773

"OR1D5",0.342266307811553,11.7642405145715,3.301497968957,0.00187358611108294,0.016478162212961,-1.55907434890962

"OR4S1",0.342779342004198,12.0317956747312,4.22416365929585,0.000113317887629216,0.00241902588520171,1.04761893854458

"KRT37",0.343020547078336,8.37543743349428,3.21227279420446,0.00241638883767626,0.0196217793541492,-1.79209875721529

"GABRQ",0.343063503207279,10.8959711657153,4.30833293081135,8.65823035775091e-05,0.0020246677499321,1.30018929449988

"OR7A17",0.343307206163628,11.6575122224694,3.33253873568492,0.00171351709119281,0.0154813672951253,-1.47710345446599

"OR2S2",0.344142634075686,7.58015583175339,2.7327964802031,0.00890389013457406,0.0479330997945818,-2.97241164407653

"LPAR3",0.344180974150222,10.3151707558966,4.72286217856739,2.24558162087381e-05,0.000811539588198213,2.57101419659197

"ZNF485",0.344277172777751,10.5932947449469,4.16219061501759,0.000137984454840016,0.00278286799620412,0.86298358499497

"NRXN2",0.344733908962045,10.1010652977539,3.5116747042572,0.00101559570971889,0.010807847502178,-0.995339702312102

"PTPN22",0.34534513131298,9.15678946277903,3.46984114557211,0.00114886251539377,0.0117401424824426,-1.10914050959626

"KEL",0.345398202070726,9.33587894787345,3.0348018852858,0.00396622456117126,0.0277589167350519,-2.24362778095034

"OR4D10",0.345464743500509,11.8000180981578,5.16678629303804,5.10574626313778e-06,0.000327371666312802,3.97166581423562

"OR1N1",0.345862263681642,11.6173296645581,4.71901340109922,2.27427293170057e-05,0.000815320009522874,2.55903137169084

"OR52E4",0.346599135266803,10.5284513793523,4.39694960809199,6.5095314745409e-05,0.00169750741323706,1.56824503663138

"OR2B6",0.346695455594931,10.5286691835441,5.17043187575667,5.04340331777039e-06,0.000325556077233322,3.98330190528102

"C5orf47",0.347254543392059,7.49506564441487,2.82849379613825,0.00692531040135628,0.0403015217208401,-2.74715491764591

"ADORA3",0.347409692920126,10.6843643233849,4.0482096035006,0.000197666166227504,0.00355560170560382,0.526506958654182

"EDN1",0.347566123196058,11.2138771278816,4.55712881388773,3.86907271360995e-05,0.00118314259442339,2.05794861648915

"VSIG2",0.347820114251267,11.8365887501603,4.89257766575413,1.2794794813424e-05,0.000580929148267364,3.10236121603494

"PDE5A",0.347991041227196,10.6980737296696,3.03685815259186,0.00394384441818292,0.027656382907438,-2.23849075409863

"WNT8B",0.348035843146732,9.71755712305748,4.63155274442485,3.03250786133584e-05,0.00101451049383628,2.28758649006123

"TANGO6",0.349042357486596,11.7803654544645,3.68024796611329,0.000613813073220568,0.00769415330456979,-0.529201635794083

"SYT16",0.349101203417135,8.08297226731162,3.79149374729294,0.00043781810283777,0.00618651504080953,-0.215290519294065

"PLAGL2",0.349122617407138,10.2430439612665,5.11811279184894,6.0148204888377e-06,0.000358743309498789,3.81649237298502

"OR12D2",0.349515122813436,10.7071994115023,3.41264579621426,0.00135829580011839,0.0132489386959203,-1.26346964457853

"TMC5",0.349936782364068,8.15296682072084,3.00266842359093,0.00433180193945581,0.0294420991242116,-2.32360796491794

"SEMA3A",0.350122643784986,6.74130646018566,2.85689181689399,0.00642186112903444,0.0382553026098226,-2.67927438511266

"SGSM1",0.350218792834534,10.0076430678339,4.15807695119678,0.000139794982452903,0.00280672552312007,0.850768974753811

"ACTR3C",0.350316535054157,9.70189276495968,4.09125951493505,0.000172648266621834,0.00326480427230231,0.653106622167799

"UBTFL1",0.350710325439753,10.1493977186131,4.42686952960828,5.90933588048855e-05,0.00159204984286234,1.65922384613037

"C1orf52",0.351326973309178,12.6820594492445,5.29991962246149,3.25644318870699e-06,0.000244766857994871,4.3977798864402

"OR13C2",0.35152720240521,6.73274930369372,3.5872825077139,0.000811340961356512,0.0093178243222289,-0.787739775044754

"CXorf38",0.352318129053218,12.9008817480352,3.07374138655314,0.00356199421694046,0.0258082278440048,-2.14596401542107

"TAS2R3",0.352558612021527,9.57762516254931,4.98234238073778,9.48340801845128e-06,0.000466067818796909,3.38557351426944

"LGALS13",0.352828935735509,9.11333049327406,3.8589600875103,0.000355969354154087,0.00533775002218144,-0.0226188188390486

"DDX53",0.352904439424135,8.23453458530039,3.43189755048812,0.00128403872674983,0.0127188088498492,-1.21168763732657

"PRAMEF5",0.35297145151528,10.8571397879598,4.4283631039184,5.88083690210501e-05,0.00158759725733031,1.66377153258385

"CA13",0.353246059453973,9.67091127703585,4.53131544355507,4.20906826407542e-05,0.00126760980094352,1.97860294884249

"GFRA1",0.354222958432729,11.5068851871381,4.66320264653875,2.7331133055094e-05,0.000945031344378622,2.38562364384882

"ZNF705B",0.35476039933145,9.50594730618204,3.76450605559648,0.000475403919663558,0.00656211475220787,-0.291884154609299

"CALN1",0.355008370637938,8.78993418334641,3.66420818123067,0.00064422668042435,0.00796445824886744,-0.574060142739623

"RETNLB",0.355333924788317,7.72226821991226,4.28347307115671,9.37625541054708e-05,0.00214765471241071,1.22538018071496

"SGCG",0.355933079298934,9.67439015012994,4.45964950744752,5.31383247036948e-05,0.00147950291193017,1.75916514003444

"APBA3",0.356371285499328,9.42928988676798,3.06534179498201,0.00364577072801242,0.0262451479088186,-2.16709925999995

"IFNA4",0.356729400234318,13.2931003379817,3.08414365638944,0.0034607498292465,0.0253296884419731,-2.11973785178508

"ADAM30",0.357149893661154,10.3964105038597,3.63487080480887,0.00070363813194953,0.00844224181250513,-0.655838705653998

"RPRD1B",0.357349809738697,11.362318080738,4.27323121188793,9.68867361933921e-05,0.002194983337441,1.1946109695836

"SLC25A20",0.357531114084425,11.9190514560285,4.97818587970575,9.6161098355802e-06,0.000470007073357088,3.37242860723215

"FCRL4",0.357961101927009,9.78434652379795,3.81485492041574,0.000407610367506961,0.00589429261227009,-0.148766844137625

"OR9G1",0.358548993541863,8.27828847193839,3.5812222712119,0.000826141149064604,0.00942713619305472,-0.804469264751618

"AURKA",0.358585568436528,9.52972713869231,4.50968173188017,4.51641509655055e-05,0.00132016584415348,1.91222829881681

"TTLL13P",0.358612003656861,9.61547780902916,3.59241441287619,0.00079900691110728,0.00923348490490835,-0.773560924175251

"CAPN11",0.358751747143186,9.09485913571509,3.62205163735767,0.000731218076712306,0.00868153076063811,-0.691461992440332

"RAC2",0.358922064790614,11.4867246069785,3.4334325397235,0.00127828761644241,0.0126758798062851,-1.20755169949671

"SLC4A9",0.359303785993028,13.1280122757275,3.74195680024516,0.000509174335624235,0.00683317306075165,-0.355667111901433

"CCL5",0.359387293535898,10.0637724395298,4.2379362056589,0.000108450075081806,0.00235444586545926,1.08880668780143

"LGR5",0.36037541972396,9.72094669372591,5.70369452858016,8.23166322738033e-07,9.22069776122745e-05,5.7023511055027

"S100A5",0.360401197457602,11.5772580474363,4.01296973269409,0.000220731903013142,0.00382252953824017,0.42332636241898

"CACNG6",0.360512398582003,12.6180329622025,3.32115711147322,0.00177063926366265,0.0158088516626975,-1.50721275364046

"CHRM2",0.360696624410904,8.7125136435833,3.54240626092073,0.000927251195725583,0.0101889414252672,-0.911253685339562

"PPARGC1A",0.360767218395647,8.92612750040565,3.09046847896548,0.00340051986745916,0.0250131167388886,-2.10376384510171

"TRIML2",0.360877149253227,10.4639806890396,4.03807766984637,0.000204046891404488,0.00363203466699989,0.496799091151553

"ICA1",0.360896573293953,10.8078074514664,6.75032150841708,2.24352907246454e-08,9.55583132793289e-06,9.12352440036466

"SVEP1",0.361113676859508,9.61282071780894,3.05355410198846,0.00376646459418695,0.0267480290295396,-2.19669651382115

"CRYGS",0.361839254992468,10.491144047501,4.86865381781904,1.38548767416608e-05,0.000604296052279274,3.02712417144517

"IQCF5",0.362174519833614,10.5190474726513,5.51764380205834,1.55412697904188e-06,0.000139008887640401,5.09925214034971

"OR4N4",0.362261001323201,11.9272016894142,3.70029942937227,0.000577729834826304,0.00741707537259892,-0.472978847073189

"C4orf45",0.362652098322595,10.2186548085414,5.13219450961422,5.73652053183104e-06,0.000347866494216697,3.86134988057403

"LEFTY2",0.362712432233234,10.9182601134188,4.29260134624363,9.10610121649106e-05,0.00210466094339611,1.25282902812498

"MTCP1",0.363540083108411,10.3226956682497,2.71250099749831,0.00938557278560668,0.0497919666553138,-3.01947956393684

"NDUFB9",0.36402015056496,13.6552229705201,2.82005805774458,0.007081773545613,0.0410258152001971,-2.76722873640915

"PMEL",0.364201966909631,9.86903867039278,3.93397091813998,0.000282310782070267,0.00451425717505946,0.193544178627944

"JAM2",0.364475038369116,10.1875351754008,4.07695254957828,0.000180600678997389,0.00335838414405851,0.610966726961762

"ABCA4",0.364852118017101,16.98871899386,3.56217819663414,0.00087435233075418,0.00981260278849531,-0.856940270448134

"C1orf115",0.36519140979364,11.0947438610237,3.90706103105023,0.000306857885696171,0.00478585938728754,0.115765695383048

"WFDC9",0.365298676383523,10.6612660794819,4.03557926576948,0.000205650744592009,0.00364968866072069,0.48947870994753

"LPCAT2",0.365367817321461,8.72278086907879,2.74999144967805,0.00851375110593527,0.0464342581394567,-2.93233944686677

"DEFB112",0.365568548498366,8.56116963160043,3.54251598566291,0.000926949353818765,0.0101889414252672,-0.910952733154194

"KCNJ14",0.365634149920652,11.131514165741,3.14744058228772,0.00290071024556427,0.0224053564693002,-1.95893386277265

"CD84",0.36635144785785,10.049122552092,3.9227195893131,0.000292333932109171,0.00461865048636788,0.160993076158635

"SLC17A4",0.366390974233349,10.9493806081722,5.14453646240185,5.50309203285346e-06,0.000340347944274862,3.90068940071309

"NKG7",0.366491109915305,10.5112249022618,3.36238679358794,0.0015719142418627,0.0146001941187341,-1.39785212340184

"TMEM81",0.366833978504117,8.26733542448767,3.82907074395079,0.000390223000149396,0.00570318566149717,-0.108185745714144

"ARSF",0.36724136670086,10.8625495877267,4.19588037670238,0.00012399072620191,0.00256934730779925,0.963211804741957

"OR4C15",0.367656364030504,11.2327547232252,4.72436518312662,2.23447406397859e-05,0.000810801349503307,2.5756945091944

"IFIH1",0.367921928311517,9.96001602403013,4.13624333283621,0.000149798023927542,0.00295776694264879,0.786026222632422

"OR10H1",0.36820492832835,12.8664317975049,3.79320800735586,0.000435529977450992,0.00615904803685439,-0.210415945501883

"OR4X2",0.368491285840101,11.4351563059546,5.43672569662593,2.04703137544343e-06,0.000165698390386007,4.83794405190899

"OR9A4",0.368563999218553,10.3814758561674,3.52412028553893,0.00097888950125862,0.0105597147304781,-0.961335970089239

"CKMT1A",0.368868920661015,12.4473042640003,3.34820320448865,0.00163775668393024,0.0150322366951401,-1.43556401152621

"PTH2R",0.36892973135021,7.49213464913359,3.36393941813195,0.0015648615252675,0.0145703927144278,-1.39371822989988

"CNTN5",0.368981533886708,6.35418993476891,4.15145801381859,0.000142756744693393,0.00284068454484995,0.831126427966144

"IP6K3",0.369042754666731,8.92565818505351,4.40399428091662,6.3630661202411e-05,0.00168332524813167,1.58964498045859

"RHOH",0.369055493948208,11.7947882803305,3.20242352019313,0.00248468791047888,0.0199858732151784,-1.81758084605464

"GMFG",0.369072973348764,11.9860151615254,4.23579944948593,0.000109191717794594,0.00236651883340241,1.08241292321833

"UCP2",0.36910183557935,12.2289251140374,2.93939507544751,0.00514565333762385,0.0330522773991932,-2.47944428443154

"DGAT2L6",0.369251274153811,7.30890629182697,2.77797872433068,0.00791236030165801,0.0442463936968928,-2.86673717065901

"ARHGAP42",0.369297394579908,11.3096147759883,6.05333439840195,2.47860648009971e-07,4.43397913225036e-05,6.84211233795962

"FLT1",0.369374214712563,11.2832995635201,4.92369897454907,1.15345918890817e-05,0.000540276694789056,3.20038940643789

"PPARG",0.369479940623032,8.55370493329063,3.47459339292536,0.00113292217061408,0.0116009414482629,-1.09625170082347

"OR10G2",0.369532216372772,11.2334413740517,5.8146755332835,5.62776746524356e-07,7.33103218908613e-05,6.06337510898839

"DPRX",0.369576726865027,9.90636065973701,4.38100203980437,6.85337281022944e-05,0.00174359593602411,1.51984883686318

"OR52B6",0.369923444299811,11.1782949014368,4.58928268209485,3.48302593582038e-05,0.00110001546614552,2.1570039162307

"SALL4",0.370218443941914,11.777610427993,4.77801569498811,1.8714752269042e-05,0.000723084672442533,2.74306578944064

"MFAP5",0.371115074592639,11.3096188080726,3.17540541175124,0.00268152806399123,0.021197461571692,-1.88723199195595

"KCNK18",0.371180112551054,9.33563853257921,5.31714798964015,3.07186025260808e-06,0.000232849610419093,4.4530898469921

"MC4R",0.371296195517946,9.41819941545703,4.34627721892718,7.66457484673982e-05,0.00187289104396769,1.41470490175373

"LEMD1",0.371422198681794,9.19641049944052,5.54334676431734,1.42373829260881e-06,0.000131965048271912,5.1823896338411

"MCTP1",0.372024355412101,8.80781702837086,3.74407979500577,0.000505898906618418,0.00680346549911663,-0.349670372159411

"TRIM42",0.372083673732815,12.4316980137494,4.80021636797642,1.73883356487917e-05,0.000695381679975646,2.81249451209953

"CRABP1",0.372236137261446,9.75146118334322,3.19229128714899,0.0025568501309265,0.0204376639821913,-1.84374427060156

"IFITM1",0.372364915969051,14.4258133734666,3.13511171580822,0.0030026122689439,0.0229056421659435,-1.99041792662479

"ITIH1",0.37245646869294,9.77302724215171,3.39665961308418,0.00142304864021338,0.0136644751072341,-1.3063397331399

"DEFB125",0.373610517146929,9.07034722359121,2.83063808438161,0.0068860533368075,0.0401823751656779,-2.74204571644269

"SPATA31D3",0.373687477350957,9.98319324218685,3.40274419048318,0.00139806368234968,0.013519331080369,-1.29003654043465

"ANKS4B",0.373824355954502,9.2065298533974,4.06080200640047,0.000190005348539515,0.00347281869683164,0.563476059045749

"CPLX1",0.373880797387317,10.1474425589074,2.98489707774962,0.00454730376421546,0.0303541389634988,-2.36759975779319

"BLNK",0.374408062616011,8.47428324253589,4.39091477466158,6.63761724778064e-05,0.001713424746689,1.54992314864479

"OR4N2",0.374463278437181,11.3421490896906,3.03053999869408,0.00401299016611199,0.0279955197609676,-2.25426770875174

"ADAMTS9",0.374811458783103,9.36725443891849,3.62414431035009,0.000726646014322003,0.00864868300080261,-0.685651263883237

"SIX2",0.375026967641363,12.2302064495901,3.36775865109947,0.00154764068673794,0.0144683603328538,-1.38354466289404

"GLRA2",0.375943241142676,7.91526190793315,3.01078916569745,0.00423654389944608,0.0290151354583426,-2.30344826253229

"SLC16A12",0.376418593336901,11.3575437613465,5.91127388334312,4.03954397732687e-07,6.08514173689648e-05,6.37823399685687

"RAB42",0.37666340327546,9.39166940539546,4.61680882723189,3.18274745524653e-05,0.00103520307685282,2.2419928038292

"OR10T2",0.377266122500922,9.14772994718276,5.04385899690174,7.71815122978436e-06,0.000412149275670485,3.58045544247894

"STRA8",0.377655617012842,9.25521796227761,4.45096462546571,5.46563253202554e-05,0.00150151868608766,1.73265948810827

"NUP214",0.377887360825538,13.2541545417807,3.49644877388685,0.00106229301855333,0.0111130759116377,-1.03684814114091

"FHDC1",0.37830079640986,9.05867051356203,3.76881431213645,0.000469202009989118,0.0064856436680034,-0.279675541792966

"TMEM132C",0.37868473518887,11.0140031498942,4.54759989620011,3.99132583211086e-05,0.00121636844651842,2.02863993002668

"SLC2A9",0.378847707071321,11.466860086429,4.32034375614883,8.33082621936998e-05,0.00198178391274348,1.33639478096022

"OR9A2",0.37904576708762,10.12000457303,4.08798204100187,0.000174439436828113,0.00329171633482923,0.643447315713247

"FAM155A",0.379438344153105,11.4113454999008,3.505560325797,0.00103410753734966,0.0109268456796504,-1.0120206904087

"PGLYRP3",0.379596120326461,10.9540298060673,4.26408161610806,9.97633954675178e-05,0.00222527104927484,1.16714857408538

"MLIP",0.380270329845539,9.5188520716591,2.97541279839453,0.00466637899033848,0.0309173532437648,-2.39100663420098

"LGALS16",0.380928807919172,11.6047308891241,3.7522476863186,0.000493485342565644,0.00671099416787068,-0.326582581434797

"GIMAP5",0.381802993401784,9.80894759214281,4.88403897284647,1.3163648924798e-05,0.000587243181086563,3.07549615202464

"OR2L5",0.381893921744565,10.7930337313692,4.11748017764288,0.00015894744950536,0.00307063814708573,0.730506718258443

"RPS27",0.382358815857305,14.6371783159741,2.83059353031953,0.00688686691586832,0.0401823751656779,-2.74215190277873

"ABCA12",0.382373811765953,12.1197447165697,3.37938928559512,0.00149630179242358,0.0141476441673707,-1.35252151031622

"CPA4",0.382665103612162,8.44858319384282,2.74373351618226,0.00865387380312156,0.0469545491246714,-2.94694391533535

"CLIP4",0.382739551666248,8.43960009926711,3.26466407063948,0.00208193748239117,0.0177351331535694,-1.65574310069912

"RGL1",0.382809640450482,11.9696148138487,3.55828362927391,0.000884538105893906,0.00985887986064553,-0.867651812786409

"ADH1C",0.383162960236644,10.2666660317128,3.87860389952401,0.000335058532708733,0.00511858419438645,0.0337949970584939

"LCNL1",0.383663854871182,12.315431394403,3.47694360094442,0.0011251171738522,0.0115474590493643,-1.08987388414998

"SLC44A3",0.383807543902581,10.5706286894231,3.94293340045778,0.000274565346639525,0.00444841457123646,0.219505147089468

"PAH",0.384148472100566,7.65311502604868,3.02275968979393,0.00409969957011275,0.0284151590894021,-2.27366623931148

"C1QTNF7",0.384718569963749,10.2286935583764,4.88463685615338,1.31374840778435e-05,0.000587243181086563,3.07737682081198

"GNG4",0.384719747830115,10.5541471742615,4.41299689910981,6.18057434175391e-05,0.0016477540148977,1.61701164393134

"DPEP2",0.384801644158641,11.0444543933086,4.66282983341294,2.73646507008847e-05,0.000945031344378622,2.38446754998306

"RPL32",0.386236091540994,14.7019500560099,3.42523475888396,0.00130928918152649,0.0128975078019424,-1.22962777947414

"KCNA3",0.387167484500626,7.64015588832533,5.93103091413162,3.77445311234861e-07,5.83259132298879e-05,6.44269307610398

"ZNF391",0.388494994068191,9.48464745927379,4.851568153015,1.4664434376464e-05,0.000626090850979867,2.97345739009038

"FAM71C",0.38855203337949,10.3146033116518,5.69086716233608,8.6010863468635e-07,9.54939778007805e-05,5.66067918502085

"GABRA3",0.388998933433033,12.6797076213833,3.35370529134002,0.00161190981349789,0.0148406869035841,-1.42094603350253

"HBM",0.389308844537808,12.6893207544815,3.84629291607522,0.000370116062275599,0.00548096542884784,-0.0589223495698574

"CLEC17A",0.390509274314477,12.0761390883386,3.88491076201022,0.000328600268666702,0.0050285117247037,0.0519368638060964

"OVCH2",0.390756570822402,8.86640989136136,5.07059126786659,7.05612743946171e-06,0.000389589702976946,3.6653329906439

"OTOA",0.391236238108391,9.96451780792292,5.89490271625741,4.27319346186204e-07,6.21366020669221e-05,6.32483663232405

"SPIC",0.391270369149076,10.4377781825106,4.87307499523993,1.36526615177501e-05,0.000603043115780324,3.04102016378272

"TRIM49B",0.39152750636794,12.0747853586084,3.73904924802721,0.000513693348293782,0.00687319394736534,-0.363877122608265

"EDEM1",0.391870097864082,8.71515293329584,3.65238991234496,0.000667556450942788,0.00814036629237596,-0.607045907910853

"KDM4E",0.391988969517483,10.4037814516716,4.96289270588086,1.01203173910295e-05,0.00048277962082167,3.32408938106747

"RIPPLY1",0.392053016239544,8.95578204567226,4.49521619495024,4.7340743321078e-05,0.0013571771751134,1.86791015346061

"CCDC172",0.392288276119681,12.5540779679983,4.81183061306493,1.67317233405556e-05,0.000678716097141041,2.84885482356295

"PRSS54",0.392492877102875,10.8211763292229,5.57316412407511,1.28602959481988e-06,0.000124355586063421,5.27891232863628

"HAS1",0.392811909167898,10.8606687958618,3.01280114878908,0.00421324809532619,0.028910930255961,-2.29844798538493

"FCGR1A",0.393015828765259,10.9611542435682,6.05757094482425,2.44273426443016e-07,4.41394679357485e-05,6.85595884682367

"SRP14",0.393207547950576,13.5164516764461,3.07987397833963,0.00350197452803379,0.0255388594912338,-2.1305094602814

"TEKT1",0.393219846564762,8.64329537266148,4.3977874879157,6.49194002398418e-05,0.00169750741323706,1.57078961941072

"COCH",0.393811961200178,8.8838461755444,4.03220705557589,0.000207834940704882,0.00367750668078105,0.479601326351925

"ST3GAL6",0.394838453542867,7.26803527537796,2.72498106962689,0.00908661154560514,0.0487407478078951,-2.99056617929568

"HAUS4",0.394999227051795,7.72632590350208,3.10255213171426,0.00328818044126644,0.024417708557001,-2.07318690276405

"TBC1D3L",0.395079543157236,12.8539872317733,2.76170694145496,0.00825704550707854,0.0456318464862922,-2.90493522951787

"OVOL2",0.395509362677645,10.9067558251447,6.3837218178859,7.94060876343367e-08,2.08791477661592e-05,7.92329504011168

"LRRC74A",0.395848189578635,7.85596721991232,2.73754783212913,0.0087944683636672,0.0475012815693365,-2.96135661600147

"DOK6",0.395914971336373,9.07729962558323,4.22315010826396,0.000113684409726648,0.00242107191142858,1.04459004895519

"SERPINA9",0.396745677682908,10.2208442201087,3.35183549581866,0.00162064965070711,0.0149134781900717,-1.42591531103671

"PRLR",0.397643958668871,8.27680612308287,4.86823675658078,1.38741037746526e-05,0.000604296052279274,3.02581351392797

"HYPM",0.397817002703842,10.725035238234,3.67404440083258,0.000625408590699286,0.00778561884413328,-0.546563427157505

"VN1R1",0.398048186057006,7.63418778033827,2.83381718441013,0.00682823109909908,0.039957548620145,-2.73446593789023

"FAM229B",0.398239183476726,12.2745402818548,3.03644179709945,0.0039483663910316,0.0276692814941401,-2.23953108898202

"ALLC",0.398333167152527,10.7985791459649,3.03578530878393,0.00395550628956005,0.027705580271707,-2.2411712472635

"FOXD4L5",0.398363342863313,12.4893013383985,3.19138384190636,0.0025634086911443,0.0204535317019984,-1.8460849551258

"KRT8",0.398616908952135,12.5027003971903,4.7412924237711,2.11304599630334e-05,0.000787505829747299,2.62843792205022

"TAS2R4",0.399379750767634,11.3137511035674,4.496827816254,4.70932530978239e-05,0.00135660419431074,1.87284513759736

"UPP2",0.400212616499001,8.52953040764613,3.93192473275319,0.000284108527943761,0.00452575018378089,0.187621078020327

"TAS2R60",0.401532571775729,11.5857407299955,5.18984829727023,4.72382754719449e-06,0.000310678496293244,4.04530752619049

"RBMS3",0.401745806103829,12.628320317421,3.59966862020997,0.000781878256894629,0.00910278170243893,-0.753499606205254

"GMPPB",0.402357154786941,10.2765207930059,2.93456839503024,0.00521326503443771,0.0332709221108345,-2.49124084213595

"CARD18",0.402492112307492,10.1592271599108,4.05336517690783,0.000194494116899776,0.00352513197286737,0.541636560273339

"ANKEF1",0.402615633024073,9.38153124620362,4.39844112521608,6.47824904643087e-05,0.00169750741323706,1.57277479908882

"PAX1",0.403371459326609,10.5033415349115,3.69264829869903,0.000591250913543125,0.00751561611683742,-0.494450933057409

"ATP6V1E1",0.403756768752135,10.0221697473181,3.12808901852205,0.00306215536125533,0.0232508052875623,-2.00831667375074

"MPEG1",0.404192581234259,10.5187880403446,6.39868293754256,7.54135847842572e-08,2.08791477661592e-05,7.97229241490069

"ENPP2",0.404651604587126,10.9212873345342,3.27668010807482,0.00201166780092234,0.017326300091815,-1.62427980863621

"ABLIM1",0.404863449246271,10.8872940384217,5.46906334187535,1.83377116273525e-06,0.000153291272570892,4.94229179992826

"METAP1",0.404997181823932,11.9316261429925,5.03004084113951,8.08402382269001e-06,0.000424174067541197,3.53662615001817

"CCDC152",0.405583260865491,8.45315046542668,2.72093735376813,0.00918250369254085,0.0490931884506465,-2.99994486923706

"ACKR2",0.405624066225528,13.4664118370747,4.07651616912355,0.000180848743106751,0.00335950484469021,0.609682441383629

"ARL14EPL",0.406189145311618,11.6673255684824,4.75423195664708,2.02462010486966e-05,0.000764101878818846,2.6687959854224

"ERAS",0.40619359849974,12.8198642303654,3.04533156151425,0.00385286480732929,0.027183795989889,-2.2172982914141

"ANKRD30BL",0.406496587075381,11.6311618974999,6.10186527276134,2.09731765267605e-07,4.0342919880346e-05,7.00076511724211

"ZW10",0.406819355894094,7.7888773575278,3.14816870858801,0.00289479516668118,0.0223886687145524,-1.95707201309971

"P2RY12",0.408385381480629,8.66786988051019,4.42328596975577,5.9782658011814e-05,0.00160819844988472,1.64831485519132

"TCP11",0.409055599007951,11.5083193683709,4.45057270219418,5.47258194046148e-05,0.00150151868608766,1.73146381775208

"KCTD1",0.409705799848165,13.0958721869108,3.68708224790967,0.000601277842946353,0.00759125747617382,-0.510056830797578

"CD163L1",0.40982077158117,8.3984541831848,3.55802494279725,0.000885218677668413,0.00986032187099018,-0.868363070550919

"CALCRL",0.410067294366289,9.19297202715826,3.59145257258212,0.000801304835779512,0.0092518061325473,-0.776219216038823

"BST2",0.410296131522902,10.1581621846271,5.23643094040418,4.03648170781556e-06,0.000279314798685643,4.1942797990404

"THBS4",0.410547219398365,11.7804935211681,3.62615049937493,0.00072228865852953,0.00860254448231343,-0.680078981636425

"RGPD1",0.410673855012258,11.258811790681,4.81344134864282,1.66426082229088e-05,0.000676872691252389,2.85389958146519

"OR5AK2",0.410935931227764,9.29178039411332,4.88989122416327,1.29097322433073e-05,0.000584016334028942,3.09390749519273

"PCP4L1",0.411588183977843,12.4878899406664,5.82913744723176,5.35539028466572e-07,7.20320126333722e-05,6.1104791112673

"TIE1",0.411785251026055,9.49003682477064,2.97479487125177,0.00467423738348328,0.0309464961336537,-2.39252994038455

"CYP2C19",0.412234483863985,10.7646099587048,4.04109058357295,0.000202128881943915,0.00360506836400268,0.505629735142263

"KRTAP19-4",0.412990703461062,11.5179957077029,4.10254910050421,0.000166614104732119,0.00318676293392746,0.686405311884467

"CNTNAP3B",0.413185410935656,11.8876471364919,4.59681794948264,3.39816878895801e-05,0.0010845467200988,2.18025231713344

"FOLR2",0.414284881707669,12.5099334546986,3.90083475482182,0.000312824817651011,0.00484932683098694,0.0978061885989554

"KCNAB3",0.414691404830553,16.3336911878577,6.58054905974406,4.02800175549572e-08,1.41288085106006e-05,8.56787961101482

"OR2G6",0.414698981333938,10.0464852651923,4.51837421505238,4.39036745307482e-05,0.00129602777835075,1.93888415319105

"FOSB",0.414905969211059,12.9869938112947,2.87444778403961,0.00612786111726585,0.0370717982843317,-2.63707692461471

"RERGL",0.414978066345139,8.80166523844013,4.13375699770418,0.000150980334417665,0.00296544380383387,0.778662921163585

"CYGB",0.415040799882828,14.2788513903726,3.26886144935916,0.00205713104455308,0.0176160925112542,-1.6447604943022

"OR4K15",0.415245751811465,7.7010700083538,3.2318323678792,0.00228598222138329,0.0189675027636019,-1.74135079901399

"SYCN",0.416083580421718,13.215465193272,3.86970422030531,0.000344380868985816,0.00520763259956657,0.00821923908122635

"DEFB4A",0.416679972771274,12.1975162247912,4.24430574518595,0.000106268283655487,0.0023211640125922,1.10787402560547

"CD53",0.417558243239787,10.5514235118212,2.73239243160651,0.00891325292797831,0.0479690678786414,-2.97335112002352

"SEL1L2",0.417910669587004,10.2946479019637,4.17050160920866,0.000134395900413052,0.00272895375991952,0.88767704182102

"CYFIP2",0.418655856981081,9.92574116035096,4.33350840322104,7.98584424943848e-05,0.00192791859349804,1.37612434263325

"HBG1",0.418827772357377,11.7885893938417,3.61024563531423,0.00075753322637527,0.00889206816707822,-0.724209848014281

"OR10H4",0.420090801405181,11.6606529811838,4.38842916239853,6.69108601337355e-05,0.00171949588060604,1.5423795796071

"NDNF",0.420345380763267,9.66956123800225,2.71398094504711,0.00934965272743126,0.0496647293400528,-3.01605581942943

"KRTAP19-1",0.420617354513158,14.6694786676709,2.89584479155374,0.0057864721740577,0.0356300025647161,-2.58540852615651

"GRAP2",0.421043275382479,9.04482216038959,4.68619598309626,2.53400018177677e-05,0.000890584071744689,2.4569844831328

"VNN3",0.421089462624685,9.30298105280516,6.42924209197271,6.78712083188759e-08,1.9861536867617e-05,8.07237525199069

"SLAMF7",0.421786335715355,9.72073744908738,5.85777447872587,4.85415103345166e-07,6.73146572383075e-05,6.20378961560952

"GAGE1",0.421990626157633,8.95654050090762,2.8175619493477,0.00712869525045582,0.0411371707533562,-2.77316054249851

"TECTA",0.422178162049395,8.40819807756475,3.94846906480138,0.00026988455721009,0.00440507741234607,0.235553879886027

"OR2A25",0.422423623013557,8.8796604697509,5.90943825348826,4.06509558350627e-07,6.08514173689648e-05,6.3722460874174

"ITGB3",0.423165868115584,11.3708493541094,4.52028581076031,4.36311349186569e-05,0.00129224730556267,1.94474860680429

"DUSP26",0.423273302962826,12.7663500024161,5.2352360210685,4.05280978534338e-06,0.000279314798685643,4.19045475699618

"ACVRL1",0.423341994411931,10.7741338517744,3.80705313588452,0.000417467913634531,0.00598884002165848,-0.171006124128177

"NR2F1",0.423954282992003,9.32260305248787,4.81422245577394,1.65995612119799e-05,0.000676872691252389,2.85634615914855

"GSN",0.423979247676304,10.9663553959858,3.05566841523662,0.0037445437504602,0.0266240632559549,-2.19139321422651

"ZFP37",0.424051867878552,8.27660340787389,3.80005395147122,0.000426506703414822,0.00606983167652168,-0.190938146976332

"IGSF21",0.424993086584122,10.4321262543209,3.83999681291811,0.000377347990908536,0.00555129786954178,-0.0769448029188524

"DEFB108B",0.425107871929546,10.8934285366338,5.74199178737644,7.21998134050548e-07,8.60820965922864e-05,5.8268379161607

"DYNC1I1",0.425548970351333,11.4688330673226,3.18813564823872,0.00258701547300453,0.020605129027862,-1.8544600185036

"CCR1",0.425759114635264,9.5050264864089,4.1418986679107,0.000147142201356871,0.00291497988933894,0.802781703888082

"GPRC5D",0.425841112938006,11.4346584513563,5.99539424444057,3.0252960119424e-07,5.15424003406072e-05,6.65281218455607

"FAM181A",0.426918554499281,14.0421022355462,3.4870927262411,0.00109199782609268,0.01128848467073,-1.06230391764727

"CARD16",0.427147381046087,11.2729347423907,7.77337814948705,6.74487917210687e-10,1.2065914350982e-06,12.4450295813619

"MBOAT4",0.429960504899679,9.73310174186917,5.61322130327514,1.12162970818563e-06,0.000114912481795018,5.40870737636062

"PRKX",0.430103144444692,8.66322792509392,2.80073556331381,0.00745259417402071,0.0424990300220135,-2.81305179365856

"ANKRD34C",0.430389324690283,9.68714048400718,4.84590359133499,1.4942978039102e-05,0.000630459750333717,2.9556769067752

"CLCN1",0.431526614346085,12.0460269874908,4.87815301081799,1.34239883714168e-05,0.000597367482528046,3.05698511032111

"TACSTD2",0.432454437804434,8.77632613544489,4.69123576850612,2.49229939540529e-05,0.000879383508568151,2.47264094986622

"BTG2",0.433906292925094,11.1113211325165,4.64487605764567,2.90274345903895e-05,0.000991526560808775,2.32882905814795

"CTC1",0.434440359886404,11.7293675998454,4.17295614576054,0.000133353550754108,0.00271394956705373,0.894973943741897

"MYO16",0.434451323640226,7.74525778729987,3.98615476218693,0.000240010686305715,0.00404670232546932,0.34509138119227

"SSH2",0.4349655182542,9.31210098202033,5.27857228873849,3.50045014458152e-06,0.00025352045601789,4.32929768218995

"PRAMEF6",0.435364211667006,11.2439846822161,4.38419447720965,6.78315040764405e-05,0.00173100966679521,1.52953153975679

"TBC1D28",0.435982851760112,12.4737919529234,3.91437874584552,0.000299985597735158,0.00471567869761357,0.1368910180013

"OR2A7",0.436122913400245,10.6421630148834,7.08781868396546,7.02310238441876e-09,4.48700994838811e-06,10.2256343534124

"AMH",0.436830426338124,10.8126092598836,4.7785172097854,1.86837177231356e-05,0.000723084672442533,2.74463310489506

"GLRA1",0.436894508239627,10.7215564851317,4.78565047474372,1.82477409454e-05,0.000715863679325133,2.76693116769953

"ZNF816-ZNF321P",0.436961523589625,9.11359305734824,2.76816864578871,0.00811853844818282,0.0450333439688504,-2.88978525068531

"XAGE3",0.437056337577214,11.387971383713,3.67468460778471,0.000624202264456692,0.00777636751492877,-0.544772407659951

"PDYN",0.437232426236466,9.17877993719203,4.85080296229747,1.47017603793724e-05,0.000626189979587125,2.97105517933079

"EPHA4",0.437404400106146,10.5128784375776,5.29113237409681,3.35477539083651e-06,0.000247989987465596,4.36958347299291

"OR4C3",0.438561116037286,11.9765022686821,5.21680389644847,4.31311379682216e-06,0.000293373736545063,4.13147612540088

"PSG8",0.438715768195697,11.8318350959827,5.06524431641162,7.18389683333134e-06,0.000391807105034952,3.64834687976683

"AGXT2",0.438729082091408,9.29280436129237,4.28360579608493,9.37227194691437e-05,0.00214765471241071,1.22577911704437

"SERPINB4",0.439338334073216,7.65707171412167,2.99792118004736,0.00438841327543048,0.0296578599788385,-2.33537634335277

"MAB21L3",0.43967847311934,11.6250158493223,4.24476698798129,0.000106111963042794,0.0023205830157366,1.10925522417696

"OR5AP2",0.439773044979258,10.597062019389,4.36321714233879,7.25776990672093e-05,0.0018097304867696,1.46595695291087

"RPL7",0.440313085669887,12.8478691199392,4.52456267893197,4.30273609091372e-05,0.00128715126973839,1.95787252834947

"NLRP10",0.440514686890274,7.62767230711681,3.38801350650664,0.00145928418881533,0.0139227385886493,-1.32947708508249

"OR2L8",0.4407221890401,11.12169208794,5.58460625252708,1.23677022309515e-06,0.000123601019670107,5.31597330419561

"CGNL1",0.441528142775725,10.3004883062007,3.52092479719041,0.000988191138725991,0.010640348366991,-0.970073072759793

"SAMD5",0.441934632517698,8.55976960658064,5.09589500437074,6.4812689725241e-06,0.000372808426525671,3.74577772573857

"RGS16",0.442283840690701,12.4698450754456,3.5745278101761,0.000842790843429133,0.0095664247449897,-0.82293158446251

"GPR85",0.442324356686636,7.27227437827235,2.90436946346345,0.00565546203541005,0.0352265182282209,-2.5647510008244

"APBB1IP",0.443556643783159,11.3085542487654,4.42981577566042,5.85324732634408e-05,0.00158409593677714,1.66819523075628

"ZNF804B",0.443582039227675,8.63241481191141,5.36500686800546,2.61173089772854e-06,0.000204022943360113,4.60692405968803

"SPINT3",0.443791140519075,11.9090489772655,4.84262158080004,1.51067406327609e-05,0.000634469796364506,2.94537779501049

"FCER1A",0.444274632502616,10.3766077029897,4.0231655919837,0.000213802748900708,0.00373853038695633,0.453136986904704

"ABCC12",0.444727524638218,10.1513238592168,5.18705240264289,4.768580294728e-06,0.000311707684631351,4.0363756782043

"PPP6R1",0.444874975408078,14.8585491431706,2.8181743628757,0.00711715652233454,0.0411103690758936,-2.77170552725067

"OR6F1",0.445262045373426,7.28823302680592,2.76183761941881,0.00825422291013343,0.0456303441407222,-2.90462909170531

"PLK2",0.446267630083277,8.7961883858259,4.76477728922068,1.95525470744434e-05,0.000742623173279656,2.70171190886106

"OR8G1",0.446698406133509,11.1886560340993,3.97638733487792,0.000247430555893487,0.00414058485910064,0.316654573475601

"GAL3ST3",0.446928034837946,10.263938765156,4.18068448113495,0.000130122727695242,0.00266945582080295,0.917960880063062

"FABP12",0.447647047965566,8.13690383768489,3.05992038851123,0.00370082214511537,0.0264604345939124,-2.18072084116411

"RPL21",0.448118276281724,13.8329194159163,3.38881662497602,0.00145588169398806,0.0138976881663567,-1.32732935660173

"FNDC7",0.448129936373443,7.04685713711527,3.72875878148023,0.000529999273191638,0.0070282853952003,-0.392907737144165

"MS4A6A",0.450047478294405,9.51409125576032,2.94091651814179,0.00512451134422046,0.0329282986482614,-2.47572313219453

"UGT1A8",0.450838487587086,8.28465355467179,3.7817448808951,0.000451052460308554,0.00631367563572748,-0.242990698185183

"PPBP",0.45111471090706,12.1339130032717,5.83753250869107,5.20333744734848e-07,7.05170481784977e-05,6.13782849096904

"ATOH1",0.451186949432437,11.2420557651803,3.17570708281009,0.00267925115891428,0.0211888258098221,-1.88645633647168

"MAGEC3",0.451218873354311,11.1099935077333,3.50710360556001,0.00102940492554792,0.010896464327294,-1.00781191805658

"THAP5",0.451360809252685,7.25009665146564,5.04905530101564,7.58483575776356e-06,0.000407380595871594,3.59694533735303

"FYN",0.451616136644809,11.0051172336718,4.99224104119778,9.17460884904439e-06,0.000455901604723764,3.41688971153

"NMUR2",0.451905950335883,12.2478992216209,4.19580240193421,0.000124021461583326,0.00256934730779925,0.962979430979695

"OR1A2",0.451943247457063,9.59125692667018,5.16950211017687,5.05923134165484e-06,0.000325556077233322,3.98033406921297

"CLEC14A",0.453024616154933,10.6428002641323,5.08399239792776,6.74567301661255e-06,0.000380673011338113,3.70792507020933

"OR52K2",0.453137590973887,11.8688420406907,5.51050933698265,1.59237529284339e-06,0.000139885255627801,5.07618655793042

"LYZL1",0.453903944753058,10.246013509819,7.02008421451961,8.86406749069619e-09,5.05954410191713e-06,10.0048051403048

"OR5AN1",0.453978309499558,7.62341291671667,4.28615285922687,9.29614605771634e-05,0.0021375161545821,1.23343589032337

"AMIGO2",0.454386991515284,7.52098652730988,2.90808799579022,0.00559918448949791,0.0349471410670464,-2.55572711853153

"PLAC8L1",0.455617714695624,12.7984684320264,4.38973419999462,6.66296069016378e-05,0.00171748852718069,1.54634002563356

"CER1",0.455890035092201,7.69318315413019,3.34205856198837,0.00166708752151229,0.015229716539982,-1.45187232099471

"ALDH3B2",0.455910188741148,12.1951845997258,5.03746369466999,7.88540627173456e-06,0.000418581699688604,3.56016659779655

"AWAT1",0.456676793039524,9.99474055510596,4.14748059347712,0.000144565769913346,0.0028734856199776,0.819329417999002

"OR52M1",0.457195331958721,10.4343424432822,4.32570061741979,8.1887284560714e-05,0.00196101958970095,1.3525554595561

"PDE6H",0.457634830968727,9.76367464654469,5.68928475908433,8.64778601583456e-07,9.54939778007805e-05,5.65553933027996

"KRT2",0.458472066129019,8.85016123782435,5.4772893311774,1.7831348665156e-06,0.000150464620882536,4.96885283885842

"OR52N4",0.459422296119708,7.64862046532783,3.15444615790913,0.00284426860593837,0.0220455464001869,-1.94100903990471

"AKR1E2",0.460414073938749,10.5127386989858,4.85351739651482,1.45697712933745e-05,0.00062430863631633,2.97957727113293

"IGSF10",0.460704612972338,9.39034246013921,5.05624601114201,7.4040939631564e-06,0.000401369202748197,3.61977135237057

"RPL39",0.461770129267148,13.8460553260284,3.1813737116147,0.00263681907496809,0.0209179851140152,-1.87187775541401

"GUCY2F",0.461843546589202,10.5902744894896,2.94940841442646,0.00500798967383923,0.0323889831074873,-2.4549298954048

"GOLGA8M",0.461916505635946,11.999932630836,4.59649024928086,3.40181659710895e-05,0.0010845467200988,2.17924099866151

"TMEM213",0.462081364834935,8.66779597561628,5.10220040621371,6.34537562325671e-06,0.000370333423814898,3.76583893420169

"HP",0.462263451101636,12.1536775459392,5.52300297974228,1.52599698883045e-06,0.000138571371234456,5.11658148186813

"SLAMF6",0.462867189388293,10.555762552883,5.96287708462351,3.38315314180325e-07,5.55240610584573e-05,6.54663384005389

"CCDC58",0.462938936114218,10.2621063840763,4.34612050628215,7.66844025060307e-05,0.00187289104396769,1.41423112910288

"ALAS2",0.463091327478613,10.6545857060312,4.13314447717728,0.000151272991971834,0.00296723964186858,0.776849232424249

"MBD3L2",0.463175343689151,9.75096740462912,3.3753536810655,0.00151392902689644,0.0142390517151159,-1.36329305831314

"CSF2RA",0.464865543152257,10.516932538628,5.13061369584712,5.76711728791441e-06,0.000348540409336151,3.85631271204068

"CPVL",0.465929986249101,8.5654932857352,4.07983173129616,0.000178972244669871,0.00334199841847529,0.619441814917448

"OR2V2",0.466916807003294,11.57420020186,5.12910619959566,5.79644516129023e-06,0.000349133358553269,3.85150950915423

"RPL31",0.467757990616516,14.6555225309475,2.89187449121427,0.00584844899561701,0.0358666109299255,-2.59501552451716

"ARHGDIB",0.468136864966089,10.5624858048311,3.4687762004489,0.00115246388355041,0.0117405617385156,-1.11202742601482

"RPL26",0.47119257548877,13.8520870699391,3.28453521424639,0.00196695265794714,0.0170561396500322,-1.60367378306468

"ESR1",0.471923570527949,9.96777016494955,4.64470712616439,2.90435417219407e-05,0.000991526560808775,2.32830588015119

"IFI44L",0.472048965407561,9.24835374483107,3.51147021938065,0.00101620965345246,0.0108079515401968,-0.995897832581642

"OR4C12",0.472217460230921,11.2417772184842,4.62260502494452,3.12283299059454e-05,0.00102315676499534,2.25991096166275

"CDH18",0.474173963198218,10.504957240781,4.8531453675785,1.45877919380751e-05,0.00062430863631633,2.97840918679135

"IFITM5",0.474947251424377,11.2807975147134,2.77820003472258,0.00790776539384253,0.0442463936968928,-2.86621655740003

"SLC51A",0.476196005448307,8.32697123457579,3.3723357790467,0.00152723992966908,0.0143190750009697,-1.37134328875083

"TEK",0.477883817656528,9.31610612861225,3.28470939041918,0.00196597192156444,0.0170559028636597,-1.60321653432903

"CEACAM6",0.478499388560829,10.0783155803626,3.65278734813321,0.000666758880228998,0.00813847124924813,-0.605937551704684

"SERPING1",0.47888180503733,13.1274097076348,4.30254770014699,8.82037723283279e-05,0.00205720636659903,1.28276465025581

"TAS2R39",0.48006113285305,12.6836059878377,5.18669536720776,4.7743253166186e-06,0.000311707684631351,4.03523516080807

"SLC36A3",0.480486679056041,11.3936408666024,3.27204781182725,0.00203848696147055,0.0175067178366522,-1.6364175235817

"EEF1B2",0.481446119292725,14.0229559513135,3.12663514304188,0.00307462045345884,0.023315763158934,-2.01201898064535

"NNT",0.481647364166047,7.42696408869217,3.62905715602312,0.000716019970924373,0.00854495080711548,-0.672002671087155

"EPHX1",0.481688976669259,10.8570937484754,3.21038244044682,0.0024293581886109,0.019697838708861,-1.79699325011476

"DUSP27",0.482069295747126,11.0751118078506,5.54946544372117,1.39433471446825e-06,0.000129984673327833,5.20218995112735

"EVPLL",0.482584546382737,10.4613091744601,3.35881351409397,0.00158826068004408,0.0146758240213371,-1.407361773955

"MTMR8",0.482608849258314,10.6140124397683,4.96783658998089,9.95453296424762e-06,0.000477417265944841,3.33971181528508

"CPA2",0.485190375534518,8.34564717940381,3.49863004565662,0.00105547944338182,0.0110741769868958,-1.03090784980146

"PRAMEF4",0.485544355852284,10.8257794354463,5.61236462502469,1.12491697904846e-06,0.000114912481795018,5.40593007985229

"STAC",0.486270699543701,8.1666603348714,4.39708287882389,6.50673031738172e-05,0.00169750741323706,1.56864975818599

"GRID2",0.486420830215124,13.0377076294097,3.90408671476938,0.000309694453733487,0.00481331371228353,0.107184649125271

"TM4SF18",0.486603155257212,10.2471713337275,4.83814814913383,1.53327996898874e-05,0.000640860873019617,2.93134324811036

"KRTAP21-2",0.486959766865668,13.2855571597774,3.66210545132491,0.000648319477260106,0.00798745670021077,-0.579933169548

"HEYL",0.48764705514972,13.1560963377594,3.40611958242284,0.00138438411251325,0.0134301775427058,-1.280985098267

"LCE5A",0.488639160462474,9.26741327869339,4.0596080107079,0.000190719190951636,0.00347431324535012,0.559968475186329

"PCSK6",0.489018803434819,9.88934032170487,4.82479139242078,1.60277633655738e-05,0.00066370522881192,2.88946175330962

"FRG2B",0.489755925157562,12.5700313910343,4.0842500252995,0.000176500994751832,0.00332086558276476,0.632452582659456

"IMPG2",0.490461638882383,10.0554707602967,5.67667839894633,9.02893696089729e-07,9.90911983395654e-05,5.61459901805276

"CR1L",0.491178794890269,10.6133913432458,5.93888500743781,3.67394948065077e-07,5.81621966897006e-05,6.46832309965075

"LAMA1",0.491336707067122,8.31759384428327,3.87219928774906,0.000341742328559422,0.00518526591653901,0.0153866346047957

"TRIM51",0.491671304121658,12.6612058304793,4.40281892534938,6.38727700697645e-05,0.00168478831577138,1.58607363783446

"GDF3",0.492942989188768,10.2824049178305,5.26824286927711,3.62493135773127e-06,0.000259385588233819,4.29618166185177

"IGLL1",0.49295047896943,11.7331019239292,3.54614906788536,0.000917008032089504,0.0101324006708148,-0.900985028739767

"C14orf178",0.493028891919555,7.4879295837569,2.81940740741092,0.00709397675811477,0.0410347707519617,-2.76877530723559

"SMTNL2",0.494516641857679,8.80921918732191,4.01861678712187,0.000216867679589993,0.00377625011834826,0.439832919920937

"POU3F2",0.494596755924507,10.3659303526214,5.02425021247527,8.24238768586412e-06,0.000428628120094254,3.5182682548944

"KRT18",0.495823776300751,12.1494694796093,4.90250400243184,1.23787808049091e-05,0.00056635296628905,3.13360892334315

"OR2J2",0.497622537990896,13.4776762925586,3.90986117790451,0.000304210389404678,0.00476117205254618,0.123847125453593

"OR7E24",0.497850697802033,14.4551438108396,3.31405908932431,0.00180717330630009,0.0160518983497529,-1.525958905581

"PCDH7",0.498026372501084,9.83929021300373,3.19856693073927,0.00251192488815797,0.0201359357037721,-1.82754539813527

"EPB41L3",0.498084723216552,10.4146275006573,4.49028429891584,4.81060513744275e-05,0.00137471110708807,1.85281206538321

"LMO2",0.498418613899304,9.12438783842924,3.98794260940941,0.000238676081699286,0.00403180021295423,0.350300019532322

"TMEM100",0.498909990013544,10.5484779180955,4.62509942615614,3.09739057473012e-05,0.00102315676499534,2.26762439866686

"NAPEPLD",0.50038801620137,8.08479989742976,3.75210964608623,0.000493692678457031,0.00671099416787068,-0.326972987379483

"OR7A10",0.501779992062833,11.781055439736,5.93582944841865,3.71272868188114e-07,5.82605292896243e-05,6.4583516321309

"MYRIP",0.502524976232693,7.59643084805323,3.19208823653479,0.0025583163012856,0.0204402502517633,-1.84426805989534

"TFPI",0.503674537196931,8.98079908714619,3.83803762809733,0.000379626072966641,0.00557564106674896,-0.0825499489994934

"OR4A47",0.504009445407203,10.8294217957043,5.47855525316199,1.77546641910278e-06,0.000150464620882536,4.9729410070241

"PRR20B",0.504609236267292,14.3970646414183,3.37637450122297,0.00150945158279353,0.0142044078719587,-1.36056907145498

"PTN",0.504626388940778,12.6639055928578,2.92064045085996,0.00541304249668789,0.0341565124276207,-2.52520798932371

"GRXCR1",0.505692372130726,7.41682931613635,3.80474383597651,0.000420429542903262,0.00601685127439717,-0.177584497856902

"DENND4A",0.506320946080992,11.7665231444447,4.96808478651779,9.94628109733122e-06,0.000477417265944841,3.3404962148642

"OR4A15",0.506883228922351,13.0916938687964,4.48411308373308,4.90807447796595e-05,0.00139365943391005,1.83392852449798

"TAS2R40",0.506956325678122,11.7114482360926,5.50584389188155,1.61789064380798e-06,0.000141182662083321,5.06110594085833

"TIMD4",0.506981995133627,10.8684790525743,3.40134723017251,0.00140376277196805,0.0135665439876126,-1.29378109845891

"EPHB1",0.507996243602314,10.086248218585,5.97194393580234,3.27933083908086e-07,5.48261209161846e-05,6.57623516474807

"EMB",0.509312789430458,8.61590563155813,3.72638131420756,0.000533836753953718,0.00705824515260758,-0.399608977248453

"CASP1",0.510503826008794,10.2218088063858,4.86027035075275,1.42464497544748e-05,0.000612851780376589,3.00078450843503

"FOXD4",0.511130476340714,11.550534525317,5.46641449152118,1.85037816073147e-06,0.000153960069382908,4.93374035288487

"GPRC5B",0.513313673166284,12.519535831888,4.06163849279188,0.000189506792024326,0.00346989457781286,0.565933669412182

"STMN2",0.513339135621466,10.4686821838774,4.12374729484715,0.000155832618451921,0.00303432296833292,0.749038629811403

"RASL11B",0.514108636999474,11.5680892648613,5.57793779686347,1.26524603913561e-06,0.000124290076671545,5.29437280753784

"SCGB2A1",0.515000776159832,8.55956919341641,4.84099109374294,1.5188752648734e-05,0.000636326923028577,2.94026200787903

"SLC26A8",0.515658553181908,8.20135575465879,4.05229483593781,0.000195148571945776,0.00352984105514459,0.538494810570305

"HIST2H2AB",0.516106050547313,13.7480277363542,2.719522513121,0.00921627476204805,0.0492555750525821,-3.00322400210555

"CCIN",0.516524479143467,13.6057064453209,4.67851235373239,2.59889893145355e-05,0.000911462695651372,2.43312524463644

"UGT2B11",0.516541502646449,13.0081771181056,3.99640716706374,0.000232454393963822,0.0039603587177322,0.374975011031406

"SMIM10",0.516666187046733,9.87489398783858,6.33061497165551,9.53617825700269e-08,2.40271398365523e-05,7.74938211823071

"CYTIP",0.516859776474815,11.3378048160303,5.62378569585507,1.08186759019777e-06,0.000112580281500558,5.44296155280871

"PRPS2",0.51687191814497,8.3239542235971,2.74086340993771,0.00871884848927314,0.0472211567134748,-2.95363415514168

"VN1R4",0.517080871189588,9.31073726082602,6.63450244359645,3.34427130114349e-08,1.30649799439843e-05,8.74451766423875

"CD200",0.517097433772083,9.5417313480023,4.46150074302054,5.28201273516235e-05,0.00147410180685366,1.76481745375074

"TMEM150C",0.517129839577333,10.4714827600591,4.97034317617673,9.87150496381145e-06,0.000476109494578781,3.34763410723296

"CLRN2",0.51738608596669,12.0597334677323,6.19308050960389,1.53191251503357e-07,3.30173288932959e-05,7.29914854811161

"ZDHHC7",0.518682117299116,13.8672920321315,3.34361207368974,0.00165962519727251,0.0151784433302699,-1.4477508709881

"FAT3",0.518972149878408,12.8901091767987,5.78544981168007,6.22099262056936e-07,7.78233125799757e-05,5.96822339739579

"ENPP5",0.521145267755049,7.85050442287,3.89454966076015,0.000318961817217235,0.00491464939552034,0.0796910336850969

"TEX37",0.521208347721933,12.4732847827296,4.97703372535812,9.65321693361733e-06,0.000470535143666159,3.36878544554074

"KRTAP6-1",0.521238143225904,13.8603958495978,4.06611731373114,0.000186858880557057,0.00342842924542072,0.579096386083755

"ZNF214",0.522177754666556,7.94626146876935,2.72449627049627,0.00909805909388856,0.0487875237201956,-2.9916911087677

"OR1S1",0.52335803494757,11.4899590907245,5.5305761190597,1.48710555094573e-06,0.000136424775389067,5.14107453088587

"TLL2",0.524713679288419,8.90347007072771,5.84973357872855,4.98997809751925e-07,6.81417696080319e-05,6.17758445092692

"LRRC37A3",0.525486110853045,11.481711300748,4.86971673345075,1.38059935965006e-05,0.000604296052279274,3.03046463887791

"SLAMF9",0.525574602879331,13.7060794948663,5.93044157296561,3.78210404978869e-07,5.83259132298879e-05,6.44077001790918

"SLPI",0.525869563563125,10.9239056066887,7.18348452227502,5.05675266581704e-09,3.93305427994787e-06,10.5371423514567

"IQCC",0.527688884480089,8.42544392874731,4.09137466962095,0.000172585659422504,0.00326480427230231,0.65344606627063

"CYSLTR2",0.529728718582142,9.46342105117075,6.97464701615211,1.03632527476682e-08,5.53051018176855e-06,9.85655536987957

"KRTAP13-2",0.531264187217264,9.77567693921424,8.44344610983888,7.05956401666037e-11,2.52577081388075e-07,14.5757337819972

"MED12L",0.53291992370148,7.50840302353412,4.05155787908435,0.000195600422388662,0.00353444035970785,0.536331854164183

"KRTAP6-2",0.535389476044262,13.0288208132721,4.33213754500273,8.02110719405611e-05,0.00193185749891144,1.37198500039175

"COL25A1",0.535919441072377,12.6807235041976,4.60739578453355,3.2824596241402e-05,0.00106184304188506,2.21290989243082

"MS4A3",0.539618078377271,7.44700748738936,3.33845175454915,0.00168453601761947,0.0153045529807997,-1.46143675660448

"KLK1",0.539706300554782,10.8882433216604,3.01516841440294,0.00418599232162121,0.0287901640297892,-2.2925619311399

"SMCP",0.539792391746316,13.1487256728062,4.47788277552634,5.00843719168269e-05,0.00141318506186138,1.81487377861676

"KRT1",0.540002303652031,9.15660527025921,4.38495357218993,6.76655671591089e-05,0.001729241901299,1.53183428396081

"LHFPL4",0.540413026596559,12.9502907842662,4.37051229856302,7.08914723915914e-05,0.00178158570199983,1.4880524982002

"HCLS1",0.545156196450945,10.8375943126602,3.21533029880355,0.00239555034117963,0.0194968153109019,-1.78417850987479

"PSG6",0.545605017702812,13.0369230795163,5.02001490436961,8.36015072613249e-06,0.000432239122369318,3.50484461540831

"QDPR",0.545620215451008,9.56463313568964,3.30455279754526,0.00185722482945585,0.0163745169907027,-1.55102771892817

"KRTAP7-1",0.546089200853535,11.1969163532755,5.73820426782804,7.31425942312457e-07,8.60820965922864e-05,5.81452177177439

"CRYGB",0.546466073409036,7.39354827413602,3.04667403222681,0.00383863259844851,0.0271205760480432,-2.21393716724563

"CADM4",0.54758592388966,10.6164724574664,3.11199642418117,0.00320281946680964,0.023922854881736,-2.04923552450001

"OR2M3",0.54762115282373,11.1624344697868,7.16748975312422,5.34208881042672e-09,3.98185944707182e-06,10.4850932959655

"DGKK",0.549005717555824,10.8964425043806,3.73236585991753,0.000524227717640227,0.00696468177930893,-0.38273644245587

"MGST2",0.5502130113733,10.5436663618581,3.5723641352198,0.000848240475875673,0.00961456521527742,-0.828894632386575

"SMNDC1",0.550297545490402,9.33775192899354,3.74779270042678,0.000500219208854368,0.00676651661869165,-0.339178477031282

"KRTAP13-1",0.550708376213242,10.9348090860159,7.90913343572495,4.25662785033169e-10,8.46075729050928e-07,12.8802358350802

"TSPAN11",0.551663615924204,11.7273640957522,3.5758071808717,0.00083958429595484,0.00955427701675326,-0.819404732567725

"SEMA6D",0.55173410201154,7.59419711546168,4.20095113318636,0.000122007821047542,0.00254085903459777,0.978327155932925

"LCP1",0.555142742713159,10.1845923096243,4.84692933931838,1.48921556100996e-05,0.000629800878744852,2.95889617453412

"TTC38",0.557412749569608,10.0051301777569,3.63817770247919,0.000696687092995434,0.00839910704543005,-0.646638198081364

"PRSS35",0.557558316000137,6.8573577828212,2.8960880650005,0.00578269460620445,0.0356300025647161,-2.58481958221298

"LGALS3BP",0.558036610102182,8.88897456561858,2.75803304744986,0.00833676629916564,0.0459305242764934,-2.91353784107826

"MSL2",0.558913463301247,7.53015121257795,3.45546557440245,0.00119839574816637,0.0120846119159798,-1.14806806990896

"APEH",0.558931395219178,9.74771049505917,4.06768301319572,0.000185941726370852,0.00341510425364288,0.583699324838027

"MRVI1",0.559439979382317,10.134040604389,6.04137137351774,2.5827618308542e-07,4.57455706852977e-05,6.80301653798248

"CABS1",0.559534487761281,8.66158805365143,4.83501627225429,1.54930432307351e-05,0.000646049068425687,2.92151985064556

"ANO5",0.560495896774208,8.7665714064394,3.07463516846641,0.00355318700363744,0.0257652867077706,-2.14371286091962

"GSTA5",0.563926098199076,10.8156698299654,6.16286527246788,1.69995513141527e-07,3.57770557010445e-05,7.20028413243548

"HMGN4",0.564223354680392,10.7807660704015,3.96474421439183,0.000256565821282369,0.00425366633634874,0.282799483682859

"TRHR",0.568225885914318,12.8182032236819,4.48941173153151,4.82427073497917e-05,0.00137641753075028,1.85014148868869

"RHOB",0.568641855702047,11.7646725491436,3.90808063414205,0.000305891297451931,0.00477499062345292,0.118708022892331

"ZFHX4",0.570884921800162,7.70599793686659,3.08825547848046,0.00342148082283697,0.0251259730869173,-2.10935540511005

"VAT1L",0.570894540406635,8.16308861359058,2.72262386385892,0.00914239715818241,0.0489226272099088,-2.99603450985827

"KRT34",0.571210924821905,9.51706704884083,5.73835111383394,7.31058145703104e-07,8.60820965922864e-05,5.81499926259307

"IRX3",0.571456714488365,9.99255892007724,3.57540756597473,0.000840584612617276,0.00955957923401809,-0.82050642848824

"MFAP4",0.575009043520124,10.8000804252991,3.97328104949376,0.000249836478410523,0.00417131548517791,0.307617781730585

"KCNJ8",0.575780381857326,10.2675205332858,7.41432455556436,2.29313338574354e-09,2.41305077279801e-06,11.2866504580556

"RNF121",0.581608599296668,13.377976925059,4.0286127818759,0.000210187735733454,0.00370083504383441,0.469077649818603

"PDE1A",0.583989041693135,11.3356936800832,7.01402882046849,9.05055683723786e-09,5.05954410191713e-06,9.98505308076798

"IER2",0.58417296530194,12.3917442928433,4.69648686438274,2.44956716973417e-05,0.000869450537686002,2.48895965990274

"ID4",0.587352624374008,9.35503541719295,5.80557405707962,5.80620827888136e-07,7.41908999292204e-05,6.03373719678693

"HHIP",0.588919623064449,10.4188768868708,5.55113807863547,1.38640224330034e-06,0.000129984673327833,5.20760327509141

"EDDM3B",0.590081238544709,7.91605889726335,4.80212975908679,1.72784440322693e-05,0.000693036065679967,2.81848287719291

"RPL7L1",0.591412423959934,13.1714791164753,4.96494545452097,1.00511541555006e-05,0.000480762290608955,3.33057545773065

"HMBS",0.593805738974373,14.9160420388357,3.6174660520791,0.000741333323113307,0.00875360515985079,-0.704188477393175

"USP41",0.595599737557688,13.3876952635169,3.94736423936098,0.000270812551852943,0.00441619484056271,0.232349972450956

"KRTAP5-7",0.596554216599982,13.7908748509131,3.50795760080263,0.00102681146161797,0.0108818899507606,-1.00548248892883

"MGST1",0.59830543147841,8.06483117406377,4.86858015681296,1.38582706952636e-05,0.000604296052279274,3.02689268185653

"RGS5",0.599369997370713,8.97092640534856,3.64059531714173,0.000691647063229909,0.00837136286476309,-0.639909037752107

"CRB1",0.599999513838611,8.62390424156223,6.31792609645864,9.9625921697569e-08,2.47528904617752e-05,7.70783245318431

"KRTAP21-1",0.602390191843604,13.7478322124045,4.41532587754456,6.13420289174055e-05,0.00163783217209473,1.62409485516094

"NPY1R",0.607742097834875,7.18101263859407,3.29921602346138,0.00188589705203835,0.016553882416052,-1.56508222979976

"FAM20A",0.609247729339579,11.6498291150684,5.1396933536282,5.59355109749598e-06,0.000343859228807923,3.8852495090866

"COL22A1",0.61172519975511,10.6601636605468,4.84742768941726,1.48675254601745e-05,0.000629800878744852,2.96046029782964

"OR2F1",0.612878843494396,13.2096754357504,4.82314122129898,1.61157462148318e-05,0.000664987252521131,2.88428985475144

"OR6M1",0.614150383583839,12.729727490669,5.95450102436713,3.48197276166377e-07,5.66263733940028e-05,6.51929112074564

"S1PR1",0.614980653907983,11.4396825643953,4.05670356166595,0.000192466528128946,0.00349901800985642,0.551438056328688

"OR5L2",0.615334387180109,11.370754719007,6.63318068363939,3.3595454045686e-08,1.30649799439843e-05,8.74019085945211

"PENK",0.615757458011652,10.4840151161128,2.96768615088515,0.00476553588993866,0.0312961349247844,-2.41003912721532

"PCDH8",0.618008422770357,12.6355614202962,4.26710983048183,9.880221834758e-05,0.00222044332163299,1.176235061123

"NXPH1",0.618616288779765,7.87986036701031,3.76357386828897,0.000476756194766053,0.00657318000154863,-0.294524824106975

"CXCL14",0.623278088454192,10.3383391115766,6.53326517257387,4.74131621774041e-08,1.63110395806073e-05,8.41304837532365

"C1orf54",0.62476869393681,12.2403602612058,3.89879710374628,0.000314801843055334,0.00487153128928795,0.0919316430761281

"PARM1",0.635811484559971,7.61735887873956,4.37491154587736,6.98931756542766e-05,0.00177050985412737,1.50138383976084

"ACSS3",0.63956579931954,7.39504950121212,3.02246318208996,0.00410303855400713,0.0284162821109692,-2.2744048713022

"CD5L",0.640794902351907,9.20193653224177,5.70686904577075,8.14270014318364e-07,9.21928878869697e-05,5.71266596456017

"NR4A2",0.643522083589907,10.6852835768325,5.74870342279964,7.05587533290331e-07,8.52855093448022e-05,5.8486650643673

"OR2M5",0.643613076995015,13.126968897153,5.3404922270162,2.83818206329589e-06,0.000217906604851074,4.52809188794458

"CCDC3",0.646491142024534,12.5029821579744,3.55158156980633,0.000902333029833675,0.0100011372804799,-0.886069907308736

"FZD4",0.647308425627347,9.82337126802106,3.71438015940017,0.000553619535871049,0.00720633272729569,-0.433402191337136

"MAOA",0.651103630655193,11.3438129069219,6.41651697383366,7.09156997680742e-08,2.01366817960489e-05,8.03069957015583

"SOST",0.651825061611735,10.9624947102513,3.42152961597185,0.00132353486438398,0.012980655257108,-1.23959549056661

"UCK2",0.652798832607274,8.0881115630446,3.97328766751271,0.000249831328527307,0.00417131548517791,0.307637031334089

"AHR",0.654508045751644,9.98178202518299,3.77705234714541,0.000457559354642786,0.00637482811153021,-0.256311080695886

"CCR10",0.657256374121998,12.4310557039815,4.52102812583432,4.35257489743987e-05,0.00129203642979992,1.94702614356552

"CX3CR1",0.660951920189863,9.43462157917473,3.51448400600073,0.00100719689870527,0.0107518649817383,-0.98767002505303

"GABBR2",0.660976863038295,8.64255564353827,5.13455874732114,5.69105964442452e-06,0.000347465412897987,3.86888407014517

"SPAM1",0.661102969594735,7.90942769947714,3.61617637493955,0.000744202289914877,0.00878168520071717,-0.707766184185342

"CEP44",0.661169603855415,8.72191862268891,6.79446883599065,1.92696791221549e-08,8.61788224540572e-06,9.26791053068107

"RABIF",0.667516350476008,8.62561806952667,3.62190552681984,0.000731538326892639,0.00868153076063811,-0.691867630383635

"CCL3",0.67502179563634,11.4388326193212,3.70903835675074,0.000562650076942973,0.00731278605451091,-0.44842559234147

"DUSP1",0.677682497280086,10.5693100562391,3.2696378942025,0.0020525730694946,0.0175854787548797,-1.64272795527802

"HOXC8",0.678309162681904,9.48024278081244,3.14294949179726,0.00293744713266471,0.0225915699725877,-1.9704117537312

"SLC7A2",0.680126809253242,10.2656028748851,3.13870685968897,0.00297255406765256,0.0227734559812577,-1.98124513472132

"PHLDA3",0.68422331773534,10.3042411923815,4.25837521779687,0.00010159947831306,0.002257540822882,1.15003307903748

"GGT5",0.68480524501323,10.0737218713509,3.71426468008456,0.000553813275748883,0.00720633272729569,-0.433727086722174

"CCL14",0.68910335872466,11.6947384538407,5.43761945820081,2.04081926462228e-06,0.000165698390386007,4.84082658244903

"TPPP",0.693875197131245,10.7830533285853,3.10543204477734,0.00326192649860625,0.0242731294232808,-2.0658881783354

"CD1A",0.695351686028376,12.486695197181,5.81114579915043,5.69631323317666e-07,7.33103218908613e-05,6.05188032601343

"ABCC9",0.698278133471357,8.29994040466871,3.93482572139417,0.000281563029405546,0.00450929367326394,0.196019014724897

"CADM3",0.706240865085123,9.87534739526955,5.99155167992553,3.06553653222112e-07,5.1735267004626e-05,6.64026266246567

"CCL18",0.7078550382204,8.67770884277335,4.0939176652456,0.000171208692128981,0.00324444098887218,0.66094319257596

"LCN2",0.715250997560048,6.87409491532926,3.14444150454616,0.00292519417482607,0.0225360889722065,-1.96659976232495

"IGFBP4",0.715973152356344,11.0840503706287,3.66094603669464,0.000650586854931341,0.00800436605768002,-0.583170706727405

"OR51A2",0.717373176821674,10.7535347570506,7.52458925858882,1.57337714227276e-09,1.85198949788474e-06,11.643431173425

"GRM7",0.723171922708808,8.432692869996,3.43835544199041,0.00126000830344077,0.0125642634003634,-1.19428005283976

"CSF1R",0.726867632071688,12.293470412901,2.9725654774009,0.00470269233700975,0.0310861445368239,-2.39802406526293

"LBP",0.732604420148382,8.15050575396051,3.1764378540446,0.00267374312129439,0.0211639781844404,-1.88457718899884

"TRMT6",0.741223986276038,8.42101707350647,2.90981346575831,0.00557324828412986,0.0348357227654784,-2.55153720829466

"DEFB1",0.746735641623291,12.8483585078993,4.87500556408734,1.35652756967583e-05,0.000600666378562648,3.04708918205993

"MEPE",0.749639472261485,10.6122051721725,5.26525244226781,3.66178024768468e-06,0.00026097843366865,4.28659693677205

"C1QA",0.749717104478883,11.8470502750178,2.83087286332902,0.00688176763093352,0.0401788319679405,-2.74148614530435

"NR2F2",0.752974944745763,9.96735549376522,3.42448190099671,0.00131217191796562,0.0129035314772825,-1.2316536496481

"NMNAT2",0.753024024499188,7.6723465394617,4.58720721098676,3.50676119669729e-05,0.00110056931662663,2.15060281121728

"CD34",0.755097571586747,10.1714258557231,4.0197795015462,0.000216080227957573,0.00377117970530052,0.443232900363924

"SRGN",0.756280984398293,9.82313614030784,4.06006943192168,0.00019044301758577,0.00347281869683164,0.561323930579363

"KLF4",0.758666180023665,13.0341604062622,4.91340918950275,1.19370961133852e-05,0.000552357900557264,3.16795858596356

"F13A1",0.769543485238938,10.5911164248946,4.04505455850182,0.000199632054749887,0.00358196371857646,0.517252425855083

"ACKR3",0.780917166317286,12.1851257205689,4.24776193843136,0.000105102376022316,0.00230696491369719,1.11822515538942

"LPL",0.787187722100557,9.67176044572808,3.72400626472726,0.000537697020160391,0.00709355604251419,-0.406301195173052

"HBB",0.788565534726205,10.9264925059661,2.86471343736806,0.00628929354533835,0.0376915149857815,-2.66049625138324

"SLITRK6",0.788866923771089,8.67593959958093,6.29460141341638,1.07967254962457e-07,2.61003543786946e-05,7.63146056393969

"C11orf96",0.792939591494346,11.2203910135408,4.02287018383086,0.000214000509600497,0.00373853038695633,0.452272785994742

"ROPN1L",0.797247050411459,8.16568385872242,3.62835535700255,0.000717528719391998,0.00855724750746897,-0.673952977662178

"TIMP3",0.79853997343668,12.8790163665814,3.10308441228627,0.00328331312241122,0.024401823201834,-2.0718382409443

"OR6B2",0.8061780954617,8.69244336210732,4.11623366261817,0.000159574155723385,0.00307610137040478,0.726822236709811

"GFPT2",0.807726891534557,11.142764880484,3.33867135325926,0.00168346873305131,0.0153026281329039,-1.4608546060312

"KLF2",0.808227912838829,12.5607210340078,5.24974184247269,3.85893644227574e-06,0.000271781551243586,4.23690195246816

"S100A12",0.81453135835671,11.7401220426836,7.56378566837735,1.37643911791633e-09,1.85198949788474e-06,11.7700453434628

"GPR34",0.822611980118971,9.01048728701423,4.59720843055488,3.39382714131364e-05,0.0010845467200988,2.18145741643227

"CAPN6",0.825242597392733,7.79444585602125,2.93447276016786,0.00521461301706979,0.0332709221108345,-2.491474445078

"FAM90A1",0.83428746469834,11.6764174358548,5.65733323887174,9.64654329829293e-07,0.000102262646234301,5.5517977719309

"PLAT",0.839000129658768,9.18458555480936,4.50719613780109,4.55310503947906e-05,0.00132424665110751,1.90460951536165

"TSC22D3",0.846326596721264,12.6640994795539,3.38474925352638,0.00147319158932512,0.0140105924196901,-1.33820340453153

"SERPINF1",0.850411863762416,13.9598448537189,5.44247647796163,2.00738580390739e-06,0.0001647253424133,4.85649276695244

"HEY1",0.853891350947944,9.71141806920712,4.87061350614049,1.37648834311909e-05,0.000604296052279274,3.0332831258622

"TMEM176B",0.85451527541454,11.3052558991309,6.58934647689278,3.90765219078036e-08,1.3980798008174e-05,8.59668414842728

"CDNF",0.854916204671291,8.47967500913022,3.46686415446273,0.00115895698536421,0.0117817081539558,-1.11720944900655

"CYP1B1",0.876825269276871,11.3793082238817,5.47071408294907,1.82349667967494e-06,0.000153148038040868,4.94762135276737

"UHRF1BP1",0.881052222888421,9.55883156825256,5.35088908155753,2.73986006620359e-06,0.000211264468639293,4.56151661955636

"TRAPPC6B",0.882645922788683,6.46831216127844,2.94795734652973,0.00502772363833641,0.0324931171120665,-2.45848582281213

"CFI",0.907299321389493,9.83909654476034,3.91482689887714,0.000299569595902504,0.00471328100360589,0.138185395689688

"C16orf54",0.911059288712803,8.33673864136757,4.91474809112787,1.1883949409614e-05,0.000552357900557264,3.17217739570814

"PRG4",0.916552619492466,11.2230231420028,3.4101072158841,0.00136838754125397,0.0133183268365028,-1.27028515455585

"APOE",0.936099163015928,11.8213347219613,3.75373962128849,0.000491249805743223,0.00670379043406844,-0.322362615824021

"GZMA",0.973195295093151,8.07255740910311,5.57113312055264,1.29497461075366e-06,0.000124547316192323,5.27233514185158

"IL13",1.0006336987763,7.55177821341079,3.23025499408847,0.0022962469115526,0.0190085890794838,-1.7454503868047

"ACKR1",1.00313061222858,7.87858027687766,3.2268213474958,0.00231874217470946,0.0191063927975023,-1.7543701585451

"C1QB",1.00833875747464,13.4607170846058,4.76941873766457,1.92546966220833e-05,0.00073599843562489,2.71620675455566

"TSPAN7",1.01104881214587,10.3582226962349,4.17525779916784,0.000132383272978427,0.00270082095473392,0.901818017501616

"ZNF354B",1.01320669751032,8.70488397765987,7.13096005667028,6.05585233956713e-09,4.16665932701986e-06,10.3661699745716

"LTF",1.04663412105359,11.4804283602063,7.14815158387634,5.70874053203736e-09,4.08494637510465e-06,10.4221461178965

"EGR1",1.05610685793304,12.3925059358989,4.01810506627439,0.000217215125036635,0.00377625011834826,0.438336701475027

"IGLL5",1.09025164864265,11.5231189099372,3.89650930861932,0.000317035964392104,0.00489340497585016,0.0853376976904352

"NR4A1",1.0948332129708,8.18323687389954,4.7879681647599,1.81082519198558e-05,0.00071352096606674,2.77417828191498

"GIMAP1",1.11020627119065,7.87292222666322,4.5430134609638,4.05150992077432e-05,0.0012242814353502,2.01454084364222

"TMEM176A",1.11434975917759,11.8728911319824,7.58174931526729,1.29465881409942e-09,1.85198949788474e-06,11.8280336312964

"SPARCL1",1.13855284568408,10.2617953442531,3.67466948059895,0.000624230742436006,0.00777636751492877,-0.544814728819204

"CPE",1.15235874127592,9.52743785029748,3.50150958063043,0.00104654864926503,0.0109933698101598,-1.02306277604274

"KCNK2",1.16521979513328,9.02092987836364,4.91273290144226,1.19640298151738e-05,0.000552357900557264,3.16582775958488

"QRFPR",1.18673511138183,6.55026124755627,5.15416929006232,5.32744703261875e-06,0.000336281587536502,3.93140912030273

"WIF1",1.19321462725406,10.7387740020035,3.15738731184281,0.00282088271136424,0.0219406882315291,-1.93347616590239

"EFEMP1",1.21744855445028,12.0910361068869,5.13791384656414,5.62715637112549e-06,0.000344740412065288,3.87957728001279

"HBD",1.22422900140222,14.7833387527829,3.29165684675946,0.00192722868989553,0.0168176556261176,-1.58496606629014

"CAMP",1.25235183326625,8.65016031400164,4.82009293691773,1.62795244765428e-05,0.00066794590220384,2.87473743252779

"PLA2G2A",1.27477395119566,12.2994898463293,4.55843868866613,3.85255693613172e-05,0.00118010943545309,2.06197916220406

"DNASE1L3",1.27721775403246,8.1888013603361,3.1140170806697,0.00318482892942724,0.0238282746627034,-2.04410496082142

"DEFA4",1.28931110288998,9.46336837080836,7.26531869867116,3.81929812228568e-09,3.25349638616993e-06,10.80321780196

"S100A9",1.30743526182505,9.26190824601484,4.8096797421759,1.68514542468978e-05,0.000680486828493804,2.84211917361238

"CFD",1.40600137455108,14.0109596782334,4.99051057352857,9.22786792952681e-06,0.000457277920751538,3.41141387567893

"DEFA3",1.96491074253514,12.5631633019354,8.55447123779866,4.87618680457491e-11,2.18075264367601e-07,14.9242127524429

"CSN1S1",2.06642804650702,11.2062076903369,8.61352770173257,4.00699496406276e-11,2.18075264367601e-07,15.1090000279842

"S100A8",2.12667440118991,12.105816336403,7.50951946022731,1.65642752340297e-09,1.85198949788474e-06,11.5947216010786
