# Supplementary material for: Identification and development of a novel 5-gene diagnostic model based on immune infiltration analysis of osteoarthritis
Source: J Transl Med. 2021 Dec 23;19:522. doi: 10.1186/s12967-021-03183-9 (PMC8705150; doi:10.1186/s12967-021-03183-9)
Supplement: Supplementary file 2 — Additional file 2: Table S2. 142 genes contained in the yellow module [file 12967_2021_3183_MOESM2_ESM.doc]

Genes GS.Normal GS.OA GS.ImmuneScore p.GS.Normal p.GS.OA p.GS.ImmuneScore Module

CSN1S1 -0.788313086784098 0.788313086784098 0.485647898966208 2.11881942977651e-10 2.11881942977651e-10 0.000832577206902463 yellow

DEFA3 -0.786383578106494 0.786383578106494 0.406018909316933 2.51184305973545e-10 2.51184305973545e-10 0.00624597010834905 grey

KRTAP13-2 -0.80260438956227 0.80260438956227 -0.0174102131563118 5.67449015461969e-11 5.67449015461969e-11 0.91068858441097 turquoise

KRTAP13-1 -0.779007839852281 0.779007839852281 0.124028347954346 4.73863446262736e-10 4.73863446262736e-10 0.422477359355831 blue

CARD16 -0.785783355826403 0.785783355826403 0.221818218981623 2.64744168088109e-10 2.64744168088109e-10 0.147864698315775 blue

TMEM176A -0.750837096977214 0.750837096977214 0.617907142526609 4.35666576114985e-09 4.35666576114985e-09 7.86328722429837e-06 yellow

S100A12 -0.753728466152193 0.753728466152193 0.264336546751158 3.5172683996478e-09 3.5172683996478e-09 0.0829317084261171 blue

OR51A2 -0.754253350740917 0.754253350740917 0.189980636811608 3.38217433611346e-09 3.38217433611346e-09 0.216757327704597 blue

S100A8 -0.744610925122803 0.744610925122803 0.730715172755631 6.84079042687621e-09 6.84079042687621e-09 1.79030550653499e-08 yellow

KCNJ8 -0.754766949092831 0.754766949092831 0.364599894475487 3.25470262369727e-09 3.25470262369727e-09 0.0149631602775573 yellow

DEFA4 -0.735314221852423 0.735314221852423 0.442013768937195 1.31072044216407e-08 1.31072044216407e-08 0.00266471008639283 grey

SLPI -0.746442892164332 0.746442892164332 0.329682798858699 5.99838660639654e-09 5.99838660639654e-09 0.0288582989034385 turquoise

OR2M3 -0.744183830535005 0.744183830535005 0.156285096310151 7.05249596605894e-09 7.05249596605894e-09 0.311023821550935 blue

LTF -0.731200169612181 0.731200169612181 0.401361941475099 1.73291030459447e-08 1.73291030459447e-08 0.00692841170285608 blue

ZNF354B -0.730654126754529 0.730654126754529 0.19551963564843 1.79765433855019e-08 1.79765433855019e-08 0.203404569152357 grey

OR2A7 -0.749910382086128 0.749910382086128 0.102255631257228 4.66315515106066e-09 4.66315515106066e-09 0.508936186953742 blue

LYZL1 -0.744158154746004 0.744158154746004 -0.0229849219169989 7.06541650459345e-09 7.06541650459345e-09 0.882267814813425 blue

PDE1A -0.734240283340935 0.734240283340935 0.240102632896367 1.41051988911485e-08 1.41051988911485e-08 0.116444573182292 blue

CYSLTR2 -0.735254054363907 0.735254054363907 0.0274042819527185 1.31613238551113e-08 1.31613238551113e-08 0.859838801473772 blue

CEP44 -0.719487856367481 0.719487856367481 0.235047896876288 3.73507568695145e-08 3.73507568695145e-08 0.124574287019605 blue

ICA1 -0.74299817156826 0.74299817156826 0.49016457768214 7.67279624843473e-09 7.67279624843473e-09 0.000731533161719549 yellow

VN1R4 -0.717205111594675 0.717205111594675 0.0185318716623168 4.31887480271948e-08 4.31887480271948e-08 0.904960175849765 blue

OR5L2 -0.712169438034226 0.712169438034226 0.0938445671707875 5.92067508969313e-08 5.92067508969313e-08 0.544572239264959 blue

TMEM176B -0.704129141857811 0.704129141857811 0.714562579171006 9.66582668293832e-08 9.66582668293832e-08 5.100646872924e-08 yellow

KCNAB3 -0.723605764719309 0.723605764719309 0.141279256526767 2.86389627249704e-08 2.86389627249704e-08 0.36031677009274 turquoise

CXCL14 -0.706175219660835 0.706175219660835 0.285926382422836 8.54550236326345e-08 8.54550236326345e-08 0.0599022819647595 blue

VNN3 -0.713494768848166 0.713494768848166 0.109364161522789 5.45249098332653e-08 5.45249098332653e-08 0.479765595573349 blue

MAOA -0.69845471023778 0.69845471023778 0.355323386312531 1.35300053940009e-07 1.35300053940009e-07 0.0179372271984251 yellow

MPEG1 -0.713737326863497 0.713737326863497 0.750551916983478 5.3706364371124e-08 5.3706364371124e-08 4.44891222671899e-09 yellow

OVOL2 -0.714008262230795 0.714008262230795 0.0838707224451469 5.28056138967622e-08 5.28056138967622e-08 0.588318269236816 blue

SMIM10 -0.69914144985533 0.69914144985533 0.168209203263105 1.29957050667329e-07 1.29957050667329e-07 0.275074004626859 brown

CRB1 -0.694318290320316 0.694318290320316 0.251369013942636 1.7205857522209e-07 1.7205857522209e-07 0.0997761715087842 blue

SLITRK6 -0.688136037042338 0.688136037042338 0.355830056255304 2.44624727757981e-07 2.44624727757981e-07 0.0177628007200242 grey

NTRK2 -0.716652454488215 0.716652454488215 0.152342058118273 4.47248261888731e-08 4.47248261888731e-08 0.323540043285369 blue

CLRN2 -0.690523391453038 0.690523391453038 -0.0782967319250746 2.13761449343229e-07 2.13761449343229e-07 0.613427568851312 blue

WT1 -0.722905862973904 0.722905862973904 -0.0849034290554203 2.99712944026022e-08 2.99712944026022e-08 0.58371696237033 blue

GSTA5 -0.686232153385557 0.686232153385557 -0.077995260939081 2.72154985698598e-07 2.72154985698598e-07 0.614798512957362 blue

ANKRD30BL -0.694080492849119 0.694080492849119 -0.0777951847801457 1.74431298776777e-07 1.74431298776777e-07 0.615709079243203 blue

MFSD9 -0.712575948197197 0.712575948197197 0.0912719560985893 5.77323054520652e-08 5.77323054520652e-08 0.555705384703132 blue

FCGR1A -0.692822581689606 0.692822581689606 -0.0407678743542267 1.87497326947333e-07 1.87497326947333e-07 0.792745101492136 blue

ARHGAP42 -0.696085100639542 0.696085100639542 0.0412758047557664 1.55347694575424e-07 1.55347694575424e-07 0.790220719662735 blue

MRVI1 -0.678675740138035 0.678675740138035 0.340732108284583 4.12363634944148e-07 4.12363634944148e-07 0.0236175670474888 blue

SLC45A2 -0.709069803070981 0.709069803070981 0.0199377254986917 7.16596761799535e-08 7.16596761799535e-08 0.897787116143423 blue

GPRC5D -0.684711038299314 0.684711038299314 0.0206694123202324 2.96191493205144e-07 2.96191493205144e-07 0.894056991442968 blue

CADM3 -0.670946763415842 0.670946763415842 0.530081456753728 6.23017033207999e-07 6.23017033207999e-07 0.000214988437628706 yellow

EPHB1 -0.676742393882223 0.676742393882223 0.106656381059751 4.57730709990871e-07 4.57730709990871e-07 0.490772448166333 blue

SLAMF6 -0.679188946292365 0.679188946292365 0.0996966915455037 4.01042813557756e-07 4.01042813557756e-07 0.519651860409138 blue

OR6M1 -0.67096439163847 0.67096439163847 0.00165372355780037 6.2243949973328e-07 6.2243949973328e-07 0.991499688135647 blue

CR1L -0.67556641210086 0.67556641210086 0.14955944904526 4.87548675814279e-07 4.87548675814279e-07 0.332560403010074 blue

OR7A10 -0.674702109993023 0.674702109993023 -0.0190391793922821 5.10602699424245e-07 5.10602699424245e-07 0.902370857768057 blue

KCNA3 -0.68485606352433 0.68485606352433 0.215040743306943 2.93817474875228e-07 2.93817474875228e-07 0.160970112267413 blue

SLAMF9 -0.673012247915783 0.673012247915783 0.245983300960608 5.58628151031896e-07 5.58628151031896e-07 0.107499358457818 blue

SLC16A12 -0.684996031735665 0.684996031735665 -0.0260523641718406 2.9154302144395e-07 2.9154302144395e-07 0.866689287918925 blue

OR2A25 -0.679164725868416 0.679164725868416 0.163684678704618 4.0157053491599e-07 4.0157053491599e-07 0.288378650239116 blue

OTOA -0.681767656213436 0.681767656213436 0.0688941057891633 3.48400810934723e-07 3.48400810934723e-07 0.656779143952065 blue

NMS -0.688103308287286 0.688103308287286 -0.0410435274101584 2.45075268060565e-07 2.45075268060565e-07 0.791374861756637 blue

SLAMF7 -0.675632280223287 0.675632280223287 0.246128959615455 4.85831979838618e-07 4.85831979838618e-07 0.10728465027677 blue

TLL2 -0.667628562965317 0.667628562965317 0.0977108656681581 7.4103405272271e-07 7.4103405272271e-07 0.528044191215465 turquoise

PPBP -0.671548769967575 0.671548769967575 0.054722800161533 6.03572992675383e-07 6.03572992675383e-07 0.724234892841623 blue

PCP4L1 -0.674689965319714 0.674689965319714 0.0212140332730347 5.10933733443549e-07 5.10933733443549e-07 0.891281985506265 blue

OR10G2 -0.679098220066148 0.679098220066148 -0.00747830327958157 4.03022900804446e-07 4.03022900804446e-07 0.961574366136498 blue

CD1A -0.659386384307386 0.659386384307386 -0.0143287477193296 1.12968398286999e-06 1.12968398286999e-06 0.926448091250006 blue

ID4 -0.661973430527916 0.661973430527916 0.487143881155392 9.91027994817798e-07 9.91027994817798e-07 0.000797808472996997 yellow

FAT3 -0.663530754569163 0.663530754569163 0.393355351573055 9.15347453299772e-07 9.15347453299772e-07 0.00825306172022473 grey

CNDP1 -0.68617276827351 0.68617276827351 -0.0334493110967266 2.73058307825179e-07 2.73058307825179e-07 0.829338250288379 blue

NR4A2 -0.656404888921098 0.656404888921098 0.338583452155463 1.31167401856984e-06 1.31167401856984e-06 0.0245690925715046 yellow

DEFB108B -0.667110175667949 0.667110175667949 -0.13083776337214 7.61242164287939e-07 7.61242164287939e-07 0.397250256029252 blue

KRT34 -0.657978652394944 0.657978652394944 -0.0187388785641256 1.21247954686458e-06 1.21247954686458e-06 0.90390348554179 blue

KRTAP7-1 -0.658986849977741 0.658986849977741 0.199354633476311 1.15263369544773e-06 1.15263369544773e-06 0.194504637950856 blue

NDUFV3 -0.676007320553274 0.676007320553274 0.0420165795965636 4.76163911934347e-07 4.76163911934347e-07 0.786542922409254 blue

CD5L -0.653621877158307 0.653621877158307 -0.0974772651493605 1.50569136880656e-06 1.50569136880656e-06 0.529035770626813 blue

LGR5 -0.672355901972898 0.672355901972898 0.0167473113690021 5.78386522461517e-07 5.78386522461517e-07 0.914076205549972 turquoise

FAM71C -0.667404648343059 0.667404648343059 0.0526274917580982 7.4970101278219e-07 7.4970101278219e-07 0.734401232892488 blue

PDE6H -0.660598212713059 0.660598212713059 -0.0427973634054386 1.06263828917939e-06 1.06263828917939e-06 0.782671452313729 blue

IMPG2 -0.65751462364143 0.65751462364143 -0.049559235978186 1.24097988528169e-06 1.24097988528169e-06 0.749369131528887 blue

FAM90A1 -0.646840741970022 0.646840741970022 0.0546552782143048 2.09500044335544e-06 2.09500044335544e-06 0.724561787138256 blue

CYTIP -0.652319399116445 0.652319399116445 0.467627642769732 1.60533184884989e-06 1.60533184884989e-06 0.0013712846343806 yellow

MBOAT4 -0.657340037371895 0.657340037371895 0.0304358281051515 1.25186254858029e-06 1.25186254858029e-06 0.844514878883034 blue

PRAMEF4 -0.653216066899359 0.653216066899359 0.190917482891295 1.53610460327713e-06 1.53610460327713e-06 0.214457219066853 blue

OR2L8 -0.65433157739269 0.65433157739269 0.14889474541311 1.45383926979151e-06 1.45383926979151e-06 0.334738102779604 blue

RASL11B -0.649175214219679 0.649175214219679 0.319689565485887 1.87154475711903e-06 1.87154475711903e-06 0.0343961294743025 blue

PRSS54 -0.658174671797084 0.658174671797084 0.0512845109724309 1.20062329763711e-06 1.20062329763711e-06 0.740941114234574 blue

GZMA -0.639671524489655 0.639671524489655 0.572888614007889 2.94481468897523e-06 2.94481468897523e-06 4.82326832092622e-05 yellow

HHIP -0.644245533853803 0.644245533853803 0.618874440709685 2.37221994990147e-06 2.37221994990147e-06 7.53923886038091e-06 yellow

DUSP27 -0.648869431204457 0.648869431204457 -0.0569542246691537 1.89950345528792e-06 1.89950345528792e-06 0.713459465786725 blue

LEMD1 -0.658586107206009 0.658586107206009 -0.00627821954682903 1.17608601100578e-06 1.17608601100578e-06 0.967737185372903 blue

OR1S1 -0.645311295318377 0.645311295318377 0.0324809093340634 2.25451046994752e-06 2.25451046994752e-06 0.834208942750192 blue

HP -0.648198738959794 0.648198738959794 0.205077375504379 1.96218518631649e-06 1.96218518631649e-06 0.18174154727521 blue

IQCF5 -0.657930790122461 0.657930790122461 -0.0410988736033875 1.21539092476975e-06 1.21539092476975e-06 0.79109981691179 blue

OR52K2 -0.647915377864428 0.647915377864428 -0.0986030797000872 1.98923802284405e-06 1.98923802284405e-06 0.524265372542922 blue

TNFRSF19 -0.667710844971738 0.667710844971738 0.149073747825329 7.37872616169723e-07 7.37872616169723e-07 0.334150786255601 blue

TAS2R40 -0.644306593631013 0.644306593631013 -0.120797105893488 2.36532562604673e-06 2.36532562604673e-06 0.434757521416607 blue

RAG2 -0.660055259809987 0.660055259809987 0.230553415625899 1.09220828567139e-06 1.09220828567139e-06 0.132154811081019 blue

OR4A47 -0.642455320533168 0.642455320533168 0.136908526635759 2.58280362496316e-06 2.58280362496316e-06 0.375516523705919 blue

KRT2 -0.645069170187735 0.645069170187735 0.0892391004852151 2.28076809319469e-06 2.28076809319469e-06 0.564577736969992 blue

CYP1B1 -0.63342899674475 0.63342899674475 0.79974813830555 3.93335351421199e-06 3.93335351421199e-06 7.44611194218767e-11 yellow

ABLIM1 -0.648947431840881 0.648947431840881 0.358602629619047 1.89233514185039e-06 1.89233514185039e-06 0.0168334703769881 blue

FOXD4 -0.641205601608009 0.641205601608009 -0.0272369772285739 2.73990522071997e-06 2.73990522071997e-06 0.860686033441678 blue

SERPINF1 -0.631678655260671 0.631678655260671 0.610638894277163 4.26100898364928e-06 4.26100898364928e-06 1.07400307753373e-05 yellow

CCL14 -0.633593875253631 0.633593875253631 0.390417858368289 3.90371906549951e-06 3.90371906549951e-06 0.00879104494471885 yellow

OR4X2 -0.650738121204137 0.650738121204137 -0.0975131456512286 1.73449382916786e-06 1.73449382916786e-06 0.528883406979143 blue

A1CF -0.659757037972053 0.659757037972053 0.0290434256249002 1.10877242250087e-06 1.10877242250087e-06 0.851546536256199 turquoise

ZNF804B -0.63764414439743 0.63764414439743 0.259143873853271 3.23735112468911e-06 3.23735112468911e-06 0.0893881258404583 blue

OCLM -0.653428542587888 0.653428542587888 0.268490013002817 1.52011052653736e-06 1.52011052653736e-06 0.0780344337241811 blue

UHRF1BP1 -0.624796096325034 0.624796096325034 0.286192404266894 5.80891370397784e-06 5.80891370397784e-06 0.059654334602401 grey

OR2M5 -0.627450548177676 0.627450548177676 -0.0549008943130133 5.15909991397393e-06 5.15909991397393e-06 0.723372917444778 blue

KCNK18 -0.640928785465844 0.640928785465844 0.206336017825332 2.77587717596266e-06 2.77587717596266e-06 0.179016748158068 blue

C1orf52 -0.642295448495611 0.642295448495611 0.135960490881828 2.60242592028577e-06 2.60242592028577e-06 0.378863080811969 turquoise

EPHA4 -0.63239514928749 0.63239514928749 0.554991040457414 4.12395480660609e-06 4.12395480660609e-06 9.23657128132275e-05 yellow

SSH2 -0.631598541585627 0.631598541585627 0.372575074882468 4.27659188968941e-06 4.27659188968941e-06 0.0127515922524967 grey

TMEM64 -0.657543707044968 0.657543707044968 0.337001504951261 1.23917553627331e-06 1.23917553627331e-06 0.0252899294463712 blue

GDF3 -0.627190066954362 0.627190066954362 -0.117705713707824 5.21976427857311e-06 5.21976427857311e-06 0.446689787639712 blue

MEPE -0.619961361634956 0.619961361634956 0.354051691468167 7.18981295539186e-06 7.18981295539186e-06 0.0183814005586523 grey

LDHAL6A -0.645003163666911 0.645003163666911 0.360055915582701 2.28797515037586e-06 2.28797515037586e-06 0.0163630023753661 brown

KLF2 -0.618077096195074 0.618077096195074 0.612060761967681 7.80543958321194e-06 7.80543958321194e-06 1.01107220860023e-05 yellow

BST2 -0.6303083562088 0.6303083562088 0.573619977546359 4.53489796325873e-06 4.53489796325873e-06 4.69310608353505e-05 yellow

DUSP26 -0.629106241871374 0.629106241871374 0.0244418340307239 4.78844727103339e-06 4.78844727103339e-06 0.874862797914648 blue

OR4C3 -0.626478691719707 0.626478691719707 -0.182827569792176 5.38880533268244e-06 5.38880533268244e-06 0.234883832255386 blue

TCTE3 -0.641041475880341 0.641041475880341 -0.0324933441288505 2.76118084654458e-06 2.76118084654458e-06 0.834146361182013 blue

IDO1 -0.638740946500714 0.638740946500714 -0.13166175029747 3.0759174433007e-06 3.0759174433007e-06 0.394258206499228 turquoise

TAS2R60 -0.627383637677646 0.627383637677646 -0.16300247654447 5.17462057693496e-06 5.17462057693496e-06 0.290420368835772 blue

ABCC12 -0.62369102273988 0.62369102273988 0.0313698519379389 6.10103046916214e-06 6.10103046916214e-06 0.83980468969447 blue

TAS2R39 -0.621523691607481 0.621523691607481 0.0603023420548231 6.71362136324418e-06 6.71362136324418e-06 0.697395001772834 blue

OR2B6 -0.632332071310152 0.632332071310152 0.190470778778111 4.13585531962578e-06 4.13585531962578e-06 0.215551825353772 blue

OR1A2 -0.621819953035438 0.621819953035438 -0.00103994475164891 6.62666716923272e-06 6.62666716923272e-06 0.994654516407821 blue

OR4D10 -0.632213160903613 0.632213160903613 -0.0413117703892725 4.15837558371579e-06 4.15837558371579e-06 0.790042052822119 blue

QRFPR -0.608588474935871 0.608588474935871 0.108313416604892 1.17111026254883e-05 1.17111026254883e-05 0.484021289830626 grey

PRAMEF2 -0.632255080196401 0.632255080196401 0.0805019663451209 4.15042367137666e-06 4.15042367137666e-06 0.603439063010874 blue

SLC17A4 -0.627489848800115 0.627489848800115 -0.0648700876718548 5.15000370034608e-06 5.15000370034608e-06 0.675689034601634 blue

FAM20A -0.613075042706223 0.613075042706223 0.372140061775931 9.68272951237854e-06 9.68272951237854e-06 0.0128645912475361 brown

EFEMP1 -0.607267432694711 0.607267432694711 0.549430616592335 1.23788135699688e-05 1.23788135699688e-05 0.000112184545317895 yellow

GABBR2 -0.611528336020519 0.611528336020519 0.12804497211781 1.03422868318883e-05 1.03422868318883e-05 0.407489093542275 turquoise

C4orf45 -0.626936693429475 0.626936693429475 -0.0608047229273671 5.27940363163894e-06 5.27940363163894e-06 0.694995586303921 blue

CSF2RA -0.617935554622353 0.617935554622353 0.270406257094809 7.85358912309285e-06 7.85358912309285e-06 0.0758529264445486 turquoise

OR2V2 -0.617697117090276 0.617697117090276 -0.0917217563468122 7.93531934953423e-06 7.93531934953423e-06 0.55375114456956 blue

PLAGL2 -0.627591943268493 0.627591943268493 0.197026081130317 5.12644276885722e-06 5.12644276885722e-06 0.199875079970832 grey

TMEM213 -0.615825698829459 0.615825698829459 -0.0312971135124733 8.60450323970013e-06 8.60450323970013e-06 0.840171306081399 blue

RNASE2 -0.635703115960297 0.635703115960297 0.163080724654456 3.54235549197726e-06 3.54235549197726e-06 0.290185710412715 turquoise

SAMD5 -0.616581408986202 0.616581408986202 0.431635594704321 8.32826100079246e-06 8.32826100079246e-06 0.00343884224992639 blue

CLEC14A -0.614905286697719 0.614905286697719 0.372069296844398 8.95233158934138e-06 8.95233158934138e-06 0.0128830536330878 yellow

PRAMEF15 -0.628250520573732 0.628250520573732 0.0340544112541974 4.97683145123806e-06 4.97683145123806e-06 0.826298020751976 blue

GREB1L -0.634309782985698 0.634309782985698 -0.0638709577933333 3.77741947777925e-06 3.77741947777925e-06 0.680415493523563 blue

OVCH2 -0.618673171017484 0.618673171017484 0.030301394358572 7.60563416289641e-06 7.60563416289641e-06 0.845193260193023 blue

PSG8 -0.614312808426178 0.614312808426178 -0.134148407525512 9.1830271483879e-06 9.1830271483879e-06 0.385308729226905 blue

IGSF10 -0.612201763304204 0.612201763304204 0.264476137720813 1.00502009429253e-05 1.00502009429253e-05 0.0827633130931455 blue

THAP5 -0.612177278441355 0.612177278441355 0.187088762019497 1.00606864692725e-05 1.00606864692725e-05 0.223965053303961 grey

PSRC1 -0.636734103561977 0.636734103561977 0.217197481510181 3.37719676000449e-06 3.37719676000449e-06 0.156711146680446 grey

OR10T2 -0.617837628143094 0.617837628143094 0.0146389330265564 7.88706155944286e-06 7.88706155944286e-06 0.924860353807291 turquoise

ALDH3B2 -0.610958526846863 0.610958526846863 0.372400876468293 1.05955053305428e-05 1.05955053305428e-05 0.0127967400118025 turquoise

METAP1 -0.614000583131502 0.614000583131502 0.279173579144567 9.30678810104196e-06 9.30678810104196e-06 0.0664768215084422 blue

SALL2 -0.624778442686447 0.624778442686447 0.00678847967350961 5.81347717773723e-06 5.81347717773723e-06 0.965116586518714 blue

POU3F2 -0.607863298393672 0.607863298393672 0.137986478834563 1.20734225214646e-05 1.20734225214646e-05 0.371732820125919 grey

PSG6 -0.60551102936489 0.60551102936489 -0.0951300316565674 1.33207763521943e-05 1.33207763521943e-05 0.539049626394166 blue

OR51I1 -0.630810802197626 0.630810802197626 -0.130131595923162 4.43263974977206e-06 4.43263974977206e-06 0.399824951240665 blue

FYN -0.607515860272585 0.607515860272585 0.71495585906557 1.22506377725651e-05 1.22506377725651e-05 4.97648572238923e-08 yellow

CFD -0.595546956569888 0.595546956569888 0.781075330114822 2.00302101500838e-05 2.00302101500838e-05 3.97603489409631e-10 yellow

TAS2R3 -0.615567709112385 0.615567709112385 -0.209809068108791 8.70072270716147e-06 8.70072270716147e-06 0.1716501701329 blue

SLC25A20 -0.614571147316924 0.614571147316924 -0.0589324722937631 9.08177247730128e-06 9.08177247730128e-06 0.703952395746344 blue

TEX37 -0.602924943185142 0.602924943185142 -0.0771938828400189 1.48279750450999e-05 1.48279750450999e-05 0.618449093001449 blue

TMEM150C -0.602539493123412 0.602539493123412 0.319306016552789 1.50655702826207e-05 1.50655702826207e-05 0.0346249869869354 blue

DENND4A -0.602792376991021 0.602792376991021 0.0192564951675403 1.49092992004002e-05 1.49092992004002e-05 0.901261974300261 blue

MTMR8 -0.603838393911755 0.603838393911755 -0.0303479417853021 1.42785877089828e-05 1.42785877089828e-05 0.844958359419387 blue

RPL7L1 -0.599738624188752 0.599738624188752 -0.0174173260561695 1.6899852503151e-05 1.6899852503151e-05 0.910652243797392 blue

KDM4E -0.609540982814191 0.609540982814191 -0.132070359419007 1.12503814224695e-05 1.12503814224695e-05 0.392779359137743 blue

OR10A4 -0.616702357377816 0.616702357377816 0.0625530830441682 8.28481496807902e-06 8.28481496807902e-06 0.686668324371581 blue

FLT1 -0.608490627077305 0.608490627077305 0.277343472827976 1.17594000641971e-05 1.17594000641971e-05 0.0683538425658506 blue

OR2T8 -0.615503324900865 0.615503324900865 -0.10670076388979 8.72488922470886e-06 8.72488922470886e-06 0.490590988585072 blue

C16orf54 -0.591404713710869 0.591404713710869 0.437065884153592 2.36379003128919e-05 2.36379003128919e-05 0.00301220314593132 grey

KLF4 -0.59266438677331 0.59266438677331 0.457969289967892 2.2482347857567e-05 2.2482347857567e-05 0.00177226322050207 yellow

KCNK2 -0.590051521592293 0.590051521592293 0.733707494954181 2.49396757908304e-05 2.49396757908304e-05 1.46262619827447e-08 yellow

KRT18 -0.597853333894323 0.597853333894323 -0.0575859918863473 1.82475913979332e-05 1.82475913979332e-05 0.710418571465739 blue

FCER1G -0.612030026499206 0.612030026499206 0.663290468312982 1.01239588952568e-05 1.01239588952568e-05 9.2666386414555e-07 yellow

VSIG2 -0.608393681797953 0.608393681797953 -0.0849342666113797 1.18074324656164e-05 1.18074324656164e-05 0.583579811962717 blue

OR5AK2 -0.6017418606249 0.6017418606249 0.234059440277526 1.55683772934587e-05 1.55683772934587e-05 0.126212735472291 blue

C1QTNF7 -0.60355085892181 0.60355085892181 0.162821376654894 1.44494732133102e-05 1.44494732133102e-05 0.290963941524471 blue

GIMAP5 -0.603781085868938 0.603781085868938 0.187228274799488 1.43124977523415e-05 1.43124977523415e-05 0.223613585699961 blue

CLCN1 -0.599269462021306 0.599269462021306 -0.00619278407646324 1.72264233924781e-05 1.72264233924781e-05 0.968175999243879 blue

SIGLEC8 -0.620722901037205 0.620722901037205 0.03275978549622 6.95394422056805e-06 6.95394422056805e-06 0.832805664674927 blue

DEFB1 -0.5897189762031 0.5897189762031 0.493828749459542 2.52694802840522e-05 2.52694802840522e-05 0.000657778956450054 yellow

SPIC -0.601948661198879 0.601948661198879 0.0182150407794435 1.54365572033677e-05 1.54365572033677e-05 0.906577788702181 blue

HEY1 -0.588299234133784 0.588299234133784 0.426574879071469 2.67229430314078e-05 2.67229430314078e-05 0.00388323739799251 yellow

LRRC37A3 -0.593971996130501 0.593971996130501 0.002031785559773 2.1337782435237e-05 2.1337782435237e-05 0.989556506955002 blue

CRYGS -0.60456694694645 0.60456694694645 -0.15001556436444 1.38538747188185e-05 1.38538747188185e-05 0.331071193302562 blue

MGST1 -0.591745503417644 0.591745503417644 0.616377945189486 2.33200258327868e-05 2.33200258327868e-05 8.40182082985065e-06 yellow

PRLR -0.600974193017255 0.600974193017255 -0.0292374407136853 1.60667980189958e-05 1.60667980189958e-05 0.850566061403113 blue

CASP1 -0.59374655085969 0.59374655085969 0.649272443119403 2.15312251654708e-05 2.15312251654708e-05 1.86273489233868e-06 yellow

AKR1E2 -0.595469322607568 0.595469322607568 -0.101351259526079 2.00929015500684e-05 2.00929015500684e-05 0.5127104894256 blue

CDH18 -0.59473497378294 0.59473497378294 0.0536684026387887 2.06948699698033e-05 2.06948699698033e-05 0.729345048267241 blue

ZNF391 -0.600345834844448 0.600345834844448 0.332927530492805 1.64856245296569e-05 1.64856245296569e-05 0.0272278698421355 grey

PDYN -0.596584011874122 0.596584011874122 -0.0932932426938831 1.92097844200037e-05 1.92097844200037e-05 0.546949112160702 blue

COL22A1 -0.589701342409341 0.589701342409341 0.402665302175353 2.52870797662286e-05 2.52870797662286e-05 0.00673120382981452 blue

LCP1 -0.591117028230523 0.591117028230523 0.861365618300856 2.39093263919344e-05 2.39093263919344e-05 6.23630967612749e-14 yellow

ANKRD34C -0.596607696366801 0.596607696366801 -0.0112536702469758 1.9191412647761e-05 1.9191412647761e-05 0.942202708796275 blue

SPINT3 -0.595487980333239 0.595487980333239 -0.110355850445947 2.00778185597861e-05 2.00778185597861e-05 0.475767204552563 blue

SCGB2A1 -0.591968612032995 0.591968612032995 -0.0121592588182721 2.31140461187502e-05 2.31140461187502e-05 0.937560552060609 blue

TM4SF18 -0.592895009451367 0.592895009451367 0.244241985938896 2.22764813496155e-05 2.22764813496155e-05 0.110091605823171 blue

CABS1 -0.590001031513324 0.590001031513324 0.0339292285285683 2.49894944969295e-05 2.49894944969295e-05 0.826926777120551 blue

PCSK6 -0.591665410095564 0.591665410095564 0.631051740086445 2.33943794204415e-05 2.33943794204415e-05 4.38436224359269e-06 yellow

OR2F1 -0.587676268140649 0.587676268140649 -0.017485199408167 2.73845467088524e-05 2.73845467088524e-05 0.910305480201171 blue

PRAMEF11 -0.607720660298005 0.607720660298005 -0.126227758707671 1.21458898854556e-05 1.21458898854556e-05 0.414231922244165 blue

CAMP -0.58241527556732 0.58241527556732 0.438860233467447 3.35998058884873e-05 3.35998058884873e-05 0.00288183791024901 grey

NR2F1 -0.594330217742982 0.594330217742982 0.101013587416215 2.10336858810416e-05 2.10336858810416e-05 0.514123324772314 blue

RGPD1 -0.59521421026579 0.59521421026579 0.0192696142231231 2.03001804603015e-05 2.03001804603015e-05 0.901195038446622 blue

CCDC172 -0.596562748645854 0.596562748645854 0.0976756869646596 1.92262917860939e-05 1.92262917860939e-05 0.528193458161119 blue

S100A9 -0.581448339939263 0.581448339939263 0.736925730342267 3.48735846807152e-05 3.48735846807152e-05 1.1732789897529e-08 yellow

EDDM3B -0.586460662018947 0.586460662018947 0.0245705504546804 2.87189219751729e-05 2.87189219751729e-05 0.874209071685445 blue

TRIM42 -0.597454027941445 0.597454027941445 -0.126502349685935 1.85454038121021e-05 1.85454038121021e-05 0.413208983991567 blue

ACPP -0.60397719758533 0.60397719758533 0.188601933402792 1.41967609887341e-05 1.41967609887341e-05 0.220173288386483 turquoise

NR4A1 -0.580295470028898 0.580295470028898 0.384063820369221 3.64499465031865e-05 3.64499465031865e-05 0.0100588698413723 yellow

GLRA1 -0.591040182346591 0.591040182346591 -0.0230869160726532 2.39823107830749e-05 2.39823107830749e-05 0.881749080803677 blue

AMH -0.590430582556538 0.590430582556538 -0.172320687927255 2.45685508482791e-05 2.45685508482791e-05 0.263339819713107 blue

SALL4 -0.595687622283379 0.595687622283379 -0.0447246642818364 1.99170757158064e-05 1.99170757158064e-05 0.773137406569601 blue

PRR11 -0.605418617305586 0.605418617305586 0.0895558537623069 1.33721143848825e-05 1.33721143848825e-05 0.563190964478284 blue

C1QB -0.579143999247821 0.579143999247821 0.823465457291018 3.80891768044782e-05 3.80891768044782e-05 6.7574977108743e-12 yellow

PLK2 -0.588692530474863 0.588692530474863 0.431359706504208 2.63128252287049e-05 2.63128252287049e-05 0.00346186576232092 yellow

ARL14EPL -0.590412333456545 0.590412333456545 0.0625078112778005 2.45863013146553e-05 2.45863013146553e-05 0.686883492731751 blue

TCHH -0.606967426330207 0.606967426330207 0.0953802410890097 1.25352435508372e-05 1.25352435508372e-05 0.537977830699635 blue

KRT8 -0.58986924758541 0.58986924758541 -0.06225286958361 2.51199558693428e-05 2.51199558693428e-05 0.688095639747776 blue

GML -0.596749093337358 0.596749093337358 -0.0992507584853936 1.90820675964237e-05 1.90820675964237e-05 0.521530633478452 blue

ARHGAP6 -0.609477852835043 0.609477852835043 0.348177335273718 1.12803935881261e-05 1.12803935881261e-05 0.0205554442121286 brown

FRMD3 -0.599048787079346 0.599048787079346 0.0379933698237462 1.73820226960718e-05 1.73820226960718e-05 0.806570549967692 blue

PRAMEF10 -0.594805126873522 0.594805126873522 0.0167932789528668 2.06366571125731e-05 2.06366571125731e-05 0.913841248569296 blue

OR4C15 -0.591181354766337 0.591181354766337 0.066415616425312 2.38483887863838e-05 2.38483887863838e-05 0.66840205969745 blue

LPAR3 -0.593729242121546 0.593729242121546 0.0291090391825163 2.15461431285248e-05 2.15461431285248e-05 0.851214926687544 blue

OR1J2 -0.601729111915927 0.601729111915927 0.193308994408234 1.55765373704113e-05 1.55765373704113e-05 0.208662740630755 blue

OR1N1 -0.59316885955831 0.59316885955831 -0.0336038697350369 2.20342721160558e-05 2.20342721160558e-05 0.828561458869263 blue

IER2 -0.577727412635519 0.577727412635519 0.575583182904474 4.01981780977135e-05 4.01981780977135e-05 4.35941043680242e-05 yellow

OR1L8 -0.603060702303153 0.603060702303153 0.0128407596576157 1.47451144539938e-05 1.47451144539938e-05 0.934068436176792 grey

TACSTD2 -0.583116990046145 0.583116990046145 -0.0726970134237366 3.27022193072068e-05 3.27022193072068e-05 0.639100705673599 grey

GRAP2 -0.583395019110454 0.583395019110454 0.0657921868579183 3.2352691657365e-05 3.2352691657365e-05 0.671337876149779 blue

CCIN -0.57814224684834 0.57814224684834 0.350337861478534 3.95697879720769e-05 3.95697879720769e-05 0.0197319976233801 turquoise

GFRA1 -0.587108380137347 0.587108380137347 0.0471801197890535 2.80006888534342e-05 2.80006888534342e-05 0.761038469963402 turquoise

DPEP2 -0.584055017907116 0.584055017907116 0.233348231315948 3.15366098900905e-05 3.15366098900905e-05 0.127401590452271 blue

BTG2 -0.578951305187024 0.578951305187024 0.36793097233783 3.83699866785425e-05 3.83699866785425e-05 0.0140027439824751 yellow

ESR1 -0.576976055041537 0.576976055041537 0.236529207241299 4.13596009359408e-05 4.13596009359408e-05 0.122148893881163 grey

WNT8B -0.584924381918514 0.584924381918514 -0.0968519744141031 3.04903938769437e-05 3.04903938769437e-05 0.531694468342583 blue

TMEM100 -0.574148810568217 0.574148810568217 0.459293482488992 4.60099558282349e-05 4.60099558282349e-05 0.00171176839675851 yellow

CXorf56 -0.595284134875528 0.595284134875528 -0.101269483036224 2.02431725016197e-05 2.02431725016197e-05 0.513052466902544 blue

OR4C12 -0.575025783853233 0.575025783853233 0.0709868502834133 4.45187935553762e-05 4.45187935553762e-05 0.647026986328035 blue

RAB42 -0.580623740017047 0.580623740017047 0.186923972101301 3.59945844294168e-05 3.59945844294168e-05 0.224380690463257 blue

ZNF658 -0.584467259024731 0.584467259024731 -0.00812476141957481 3.10364803628242e-05 3.10364803628242e-05 0.958255488063813 blue

COL25A1 -0.571361508834785 0.571361508834785 0.0408346058784523 5.10573779225701e-05 5.10573779225701e-05 0.792413329974999 blue

GPR34 -0.565929108563316 0.565929108563316 0.502378384657889 6.23735287719609e-05 6.23735287719609e-05 0.0005109344092432 grey

CNTNAP3B -0.575971826685018 0.575971826685018 -0.0917918588731386 4.29597874216202e-05 4.29597874216202e-05 0.55344686364236 blue

GOLGA8M -0.573196896592257 0.573196896592257 -0.0690125138160475 4.76800602330778e-05 4.76800602330778e-05 0.656225838514674 blue

CHN1 -0.582507086442486 0.582507086442486 0.32257495999721 3.3481099897715e-05 3.3481099897715e-05 0.0327140865892305 blue

OR52B6 -0.578739555654428 0.578739555654428 -0.00142641549633635 3.86807472021893e-05 3.86807472021893e-05 0.992668041097993 blue

SPZ1 -0.593578453426705 0.593578453426705 0.0904754461705813 2.16765054523715e-05 2.16765054523715e-05 0.559173907873678 blue

NMNAT2 -0.565715529110071 0.565715529110071 0.207442203326468 6.28618882652753e-05 6.28618882652753e-05 0.176646280114221 grey

TYSND1 -0.581182677479193 0.581182677479193 0.0156520052126475 3.52312028411616e-05 3.52312028411616e-05 0.91967680876629 blue

OR8D1 -0.593141744841271 0.593141744841271 -0.0443456927431513 2.20581458544966e-05 2.20581458544966e-05 0.775009561638638 turquoise

RGS6 -0.589075550727937 0.589075550727937 -0.0326567917235612 2.59189710090245e-05 2.59189710090245e-05 0.833323859898583 blue

PLA2G2A -0.560726898965886 0.560726898965886 0.461776959183295 7.53081793437512e-05 7.53081793437512e-05 0.0016032271408969 yellow

EDN1 -0.578092939901696 0.578092939901696 -0.00460349529060853 3.96440049981103e-05 3.96440049981103e-05 0.976340464542754 blue

TNFRSF1A -0.587887161186846 0.587887161186846 0.360342769093381 2.71589129725848e-05 2.71589129725848e-05 0.0162714677924196 turquoise

TMEM132C -0.574127631786226 0.574127631786226 -0.0281753535699162 4.60465246872028e-05 4.60465246872028e-05 0.855936114797112 blue

DCC -0.582380783337727 0.582380783337727 0.13814755611907 3.36445016778651e-05 3.36445016778651e-05 0.371169388922466 blue

GIMAP1 -0.559839532236638 0.559839532236638 0.722079794154157 7.77438929380102e-05 7.77438929380102e-05 3.16182072005096e-08 yellow

CA13 -0.57506139035316 0.57506139035316 0.120169512323355 4.44591932536334e-05 4.44591932536334e-05 0.437165473093903 blue

RPL7 -0.567852997407156 0.567852997407156 0.241070572083608 5.81278605731773e-05 5.81278605731773e-05 0.114934716496425 brown

COL28A1 -0.582017497706975 0.582017497706975 0.119632080296989 3.41185692208419e-05 3.41185692208419e-05 0.439233360760956 blue

CCR10 -0.561215582751262 0.561215582751262 -0.178177863606897 7.39966760620174e-05 7.39966760620174e-05 0.247206367854709 blue

OR52L1 -0.585748355962121 0.585748355962121 0.0413058559396967 2.95282171762522e-05 2.95282171762522e-05 0.790071433362467 blue

ITGB3 -0.568433101018796 0.568433101018796 0.3116831411041 5.69004116853008e-05 5.69004116853008e-05 0.039439664931525 yellow

OR2G6 -0.568782995026719 0.568782995026719 0.0221279036357627 5.61715448864691e-05 5.61715448864691e-05 0.886628442373624 blue

DNAH8 -0.576128714310244 0.576128714310244 -0.124339186775847 4.27061241453527e-05 4.27061241453527e-05 0.421306455879902 blue

AURKA -0.572489738102757 0.572489738102757 0.169312003294987 4.89563797650088e-05 4.89563797650088e-05 0.271893394875444 grey

PLAT -0.558074993225629 0.558074993225629 0.441788739085042 8.28016985849148e-05 8.28016985849148e-05 0.00267971217403413 grey

CCR8 -0.577588280949031 0.577588280949031 -0.10844654457888 4.04109681516582e-05 4.04109681516582e-05 0.483481009867254 blue

TAS2R4 -0.567844527748411 0.567844527748411 -0.140648866466164 5.81459591467441e-05 5.81459591467441e-05 0.362485747964822 blue

RIPPLY1 -0.568232214813864 0.568232214813864 0.193187536767313 5.73227673298937e-05 5.73227673298937e-05 0.208954361837048 turquoise

EPB41L3 -0.562257794391949 0.562257794391949 0.470709687147215 7.12688709774133e-05 7.12688709774133e-05 0.00126151842810709 yellow

TRHR -0.560139683609061 0.560139683609061 0.0583975004139069 7.69121013363445e-05 7.69121013363445e-05 0.706519041256948 blue

OR4A15 -0.561400958244122 0.561400958244122 0.163339213041445 7.35046422769166e-05 7.35046422769166e-05 0.289411403445463 blue

SMCP -0.559841927937794 0.559841927937794 -0.173212231061384 7.77372215732393e-05 7.77372215732393e-05 0.260839959312004 blue

MUSK -0.581480287976171 0.581480287976171 -0.0998072397440456 3.48308026038897e-05 3.48308026038897e-05 0.519186627848651 blue

CD200 -0.559042798923215 0.559042798923215 0.458404704664274 7.99917149852667e-05 7.99917149852667e-05 0.00175216593422936 yellow

SGCG -0.568062540395508 0.568062540395508 0.11452118046069 5.76817268748911e-05 5.76817268748911e-05 0.459167053325875 blue

CFAP52 -0.577571716163126 0.577571716163126 -0.081054775824399 4.0436371034153e-05 4.0436371034153e-05 0.600946232408975 blue

GABRG3 -0.580419705014802 0.580419705014802 0.0179067607700929 3.62769971639313e-05 3.62769971639313e-05 0.908152102835192 blue

STRA8 -0.565263181720323 0.565263181720323 -0.0768944334340388 6.39077310593817e-05 6.39077310593817e-05 0.619815537258825 blue

TCP11 -0.562929582770871 0.562929582770871 -0.13762880370088 6.95594756770699e-05 6.95594756770699e-05 0.372985756161834 blue

FETUB -0.573254439255298 0.573254439255298 -0.0285957991696721 4.75775526058134e-05 4.75775526058134e-05 0.853809503316444 blue

CACNG5 -0.570344769424674 0.570344769424674 -0.192563902999763 5.3020915820691e-05 5.3020915820691e-05 0.210456195532 turquoise

STAB2 -0.579457647593463 0.579457647593463 -0.0387217805108182 3.76361172522408e-05 3.76361172522408e-05 0.802935027612488 blue

APBB1IP -0.55906898912548 0.55906898912548 0.688740471854302 7.99168940255611e-05 7.99168940255611e-05 2.36441356138092e-07 yellow

PRAMEF5 -0.565401279672862 0.565401279672862 -0.11421345000101 6.35867741233497e-05 6.35867741233497e-05 0.460382652366301 blue

UBTFL1 -0.565491428272566 0.565491428272566 0.0507027786743735 6.33780509125827e-05 6.33780509125827e-05 0.743779602047288 blue

P2RY12 -0.560446761129932 0.560446761129932 0.391041216143792 7.60695096722759e-05 7.60695096722759e-05 0.0086744243527818 blue

KRTAP21-1 -0.552812631781817 0.552812631781817 0.0505204121472546 9.97155533027704e-05 9.97155533027704e-05 0.744670129326631 blue

GNG4 -0.56114474209054 0.56114474209054 0.0082285931975928 7.41854961019643e-05 7.41854961019643e-05 0.957722485366711 blue

KRTAP23-1 -0.570956096797092 0.570956096797092 -0.15038990851286 5.18322164934705e-05 5.18322164934705e-05 0.329852076278147 blue

APOL4 -0.56704348182033 0.56704348182033 -0.0483576817614337 5.98811065513849e-05 5.98811065513849e-05 0.755255911691757 blue

IP6K3 -0.561578803083077 0.561578803083077 -0.193498890793426 7.30353995368851e-05 7.30353995368851e-05 0.208207367042364 blue

TRIM51 -0.554580656828889 0.554580656828889 0.161942858503685 9.37114599869807e-05 9.37114599869807e-05 0.293610173010053 blue

ANKEF1 -0.558508239992235 0.558508239992235 0.0858253069406532 8.15328613382695e-05 8.15328613382695e-05 0.579623165031589 blue

OR1C1 -0.575704823918514 0.575704823918514 -0.0596430718148026 4.33946566494063e-05 4.33946566494063e-05 0.700548161602931 blue

TEKT1 -0.559090686795768 0.559090686795768 -0.0993193158153805 7.98549557299333e-05 7.98549557299333e-05 0.521241573718121 blue

STAC -0.554251745551329 0.554251745551329 0.162797874624031 9.48028866018552e-05 9.48028866018552e-05 0.291034531356357 grey

OR52E4 -0.563066285225382 0.563066285225382 -0.107863704435699 6.9216238446509e-05 6.9216238446509e-05 0.485848710503578 blue

S1PR5 -0.571300888145581 0.571300888145581 -0.00835260112844109 5.11725621479941e-05 5.11725621479941e-05 0.957085935724945 blue

BLNK -0.559886100505933 0.559886100505933 0.469251544836061 7.76143065710658e-05 7.76143065710658e-05 0.00131243583759455 blue

PLAC8L1 -0.554817616900293 0.554817616900293 -0.0590061585452276 9.29322511717698e-05 9.29322511717698e-05 0.703599123477961 blue

OR10H4 -0.556498169548231 0.556498169548231 -0.19290522843621 8.75729204410973e-05 8.75729204410973e-05 0.209633286535797 blue

TYR -0.570154543663476 0.570154543663476 0.227570474577062 5.33958444962808e-05 5.33958444962808e-05 0.137372727956003 blue

TYW1 -0.571652438360807 0.571652438360807 0.075086908817723 5.05078741644647e-05 5.05078741644647e-05 0.628090374680782 blue

KRT1 -0.551476261415253 0.551476261415253 -0.106152839159492 0.000104483570533569 0.000104483570533569 0.492833615730418 blue

PRAMEF6 -0.555275329504732 0.555275329504732 -0.0808994041385265 9.14437926161448e-05 9.14437926161448e-05 0.601646410119884 blue

DPRX -0.559353396694546 0.559353396694546 0.203458013669996 7.91084826111187e-05 7.91084826111187e-05 0.185290707340171 turquoise

PARM1 -0.548602649409879 0.548602649409879 0.349853482259975 0.000115445434501031 0.000115445434501031 0.0199141545479274 yellow

FAM161B -0.572740686285426 0.572740686285426 -0.190834336340923 4.84999270911305e-05 4.84999270911305e-05 0.214660668712309 blue

LHFPL4 -0.550153034138922 0.550153034138922 0.0517031147560653 0.000109407957804321 0.000109407957804321 0.738900688960566 blue

SGPP2 -0.572849274663916 0.572849274663916 -0.0132774546327517 4.83036215032165e-05 4.83036215032165e-05 0.931831382001017 blue

TRIM77 -0.563828931201249 0.563828931201249 0.140662697600913 6.73295194529361e-05 6.73295194529361e-05 0.362438074909484 blue

TRIM61 -0.572375710933063 0.572375710933063 -0.112017083061395 4.91650781523081e-05 4.91650781523081e-05 0.469108936199752 blue

OR5AP2 -0.553106712921818 0.553106712921818 -0.0558524431295075 9.86932351516648e-05 9.86932351516648e-05 0.718773175183939 blue

CDRT1 -0.566372392608151 0.566372392608151 -0.17607901011835 6.13709609182708e-05 6.13709609182708e-05 0.252909072258074 blue

MC4R -0.555902058036095 0.555902058036095 -0.134571581808179 8.94408733599872e-05 8.94408733599872e-05 0.383797732546648 blue

CCDC58 -0.55047172146393 0.55047172146393 0.182148835707957 0.000108203062663567 0.000108203062663567 0.23665596615568 grey

RCSD1 -0.568438461658333 0.568438461658333 0.0576675151182922 5.68891801301084e-05 5.68891801301084e-05 0.710026496804928 blue

KRT23 -0.567906829869543 0.567906829869543 -0.070599688449526 5.80129475702008e-05 5.80129475702008e-05 0.648826818071022 blue

CYFIP2 -0.551443353414301 0.551443353414301 0.099603887914088 0.000104603553409925 0.000104603553409925 0.520042576479161 grey

KRTAP6-2 -0.546778419621818 0.546778419621818 0.0946316330099146 0.000122935886918072 0.000122935886918072 0.541187632281415 blue

OR52M1 -0.548816036682554 0.548816036682554 0.206728250625401 0.000114596890596313 0.000114596890596313 0.178173629731036 turquoise

HSD3B1 -0.55881986620172 0.55881986620172 -0.146680211349865 8.06311823751382e-05 8.06311823751382e-05 0.342057065777137 blue

APIP -0.5665165027782 0.5665165027782 -0.0239676260662974 6.10482082275237e-05 6.10482082275237e-05 0.877271916980918 blue

SLC2A9 -0.552821649910541 0.552821649910541 0.10886627662752 9.96840609149373e-05 9.96840609149373e-05 0.48177965744533 blue

TSGA13 -0.564206932655682 0.564206932655682 -0.0152393109117242 6.64118569707649e-05 6.64118569707649e-05 0.921788039932329 turquoise

OR11G2 -0.561279471696376 0.561279471696376 -0.0292171696099659 7.38267607699872e-05 7.38267607699872e-05 0.85066849308294 blue

GABRQ -0.554874628730986 0.554874628730986 -0.165571155156157 9.27456577141163e-05 9.27456577141163e-05 0.282781403058951 blue

SERPING1 -0.545800725091573 0.545800725091573 0.473222372239462 0.000127129513574904 0.000127129513574904 0.00117788708194396 brown

CCR2 -0.562832902961592 0.562832902961592 -0.0796484564719161 6.98031581257237e-05 6.98031581257237e-05 0.607296638398489 blue

LEFTY2 -0.551472611664645 0.551472611664645 -0.0876419264639366 0.00010449687138819 0.00010449687138819 0.571594310346136 blue

ZSCAN4 -0.55448314356756 0.55448314356756 -0.137043934010446 9.40338404118457e-05 9.40338404118457e-05 0.375039977480457 blue

CCDC65 -0.564937080757102 0.564937080757102 -0.0538806104538457 6.46714999035008e-05 6.46714999035008e-05 0.728315639511321 blue

OR5AN1 -0.545253869931425 0.545253869931425 0.182139826454666 0.000129531366944357 0.000129531366944357 0.236679549864629 grey

AGXT2 -0.545702734769037 0.545702734769037 0.166107633892374 0.000127556902895968 0.000127556902895968 0.281202700053452 blue

RETNLB -0.551248198630218 0.551248198630218 0.197251648835984 0.000105317666597038 0.000105317666597038 0.199350325538577 turquoise

OR4K17 -0.553051545119462 0.553051545119462 0.1784136156909 9.88842864518938e-05 9.88842864518938e-05 0.246571282149537 turquoise

RPRD1B -0.550067910587495 0.550067910587495 0.156783429090695 0.000109731849938069 0.000109731849938069 0.309464203353409 turquoise

PCDH8 -0.539082379364871 0.539082379364871 -0.117416332968483 0.000159640850907771 0.000159640850907771 0.447815864508742 blue

HNMT -0.550740779518591 0.550740779518591 0.656212354078435 0.000107195207352277 0.000107195207352277 1.32431231439062e-06 yellow

OR1L6 -0.5555683578815 0.5555683578815 -0.179411290197292 9.05022992565795e-05 9.05022992565795e-05 0.243895878319577 blue

PGLYRP3 -0.547361302151875 0.547361302151875 -0.208839225317584 0.000120495890586723 0.000120495890586723 0.173684895242373 blue

GABRP -0.562064393735125 0.562064393735125 0.0494131755983456 7.17680333168782e-05 7.17680333168782e-05 0.750083982395157 turquoise

PHLDA3 -0.537350657612423 0.537350657612423 0.311067381469665 0.000169160469868177 0.000169160469868177 0.0398513680528015 grey

PHB -0.553483077052263 0.553483077052263 0.109811981790547 9.73987799288293e-05 9.73987799288293e-05 0.477957845014753 brown

GABRA6 -0.559335240691521 0.559335240691521 0.313454712346716 7.91598666264454e-05 7.91598666264454e-05 0.0382745416329648 blue

ACKR3 -0.535432435249365 0.535432435249365 0.31034080618739 0.000180303352963487 0.000180303352963487 0.040341675940025 brown

ZNF705E -0.553659711839815 0.553659711839815 -0.230506771833604 9.67966069858691e-05 9.67966069858691e-05 0.132235247234002 blue

MAB21L3 -0.541981996066695 0.541981996066695 -0.239753670429367 0.000144782722842242 0.000144782722842242 0.116992575080594 blue

DEFB4A -0.543096626032797 0.543096626032797 -0.196954721566037 0.000139413271482425 0.000139413271482425 0.200041291116944 blue

CCL5 -0.546443991848003 0.546443991848003 0.608446774661233 0.000124355999083739 0.000124355999083739 1.17811048464906e-05 yellow

GMFG -0.545428622189391 0.545428622189391 0.757421559010423 0.000128759393770717 0.000128759393770717 2.66454298950298e-09 yellow

CTAGE15 -0.555808785822152 0.555808785822152 -0.0246763665347726 8.97364052455793e-05 8.97364052455793e-05 0.873671713899281 blue

OR6K2 -0.548488686062216 0.548488686062216 0.103258403090058 0.000115900948910204 0.000115900948910204 0.504767689280669 blue

OR4S1 -0.546640619168214 0.546640619168214 -0.000579167286132998 0.00012351924867167 0.00012351924867167 0.997022972020047 blue

DOK6 -0.542288848817376 0.542288848817376 0.291286534650235 0.000143286134040903 0.000143286134040903 0.0550625973565925 grey

NCR1 -0.556206503912966 0.556206503912966 -0.158489050741428 8.84823916734824e-05 8.84823916734824e-05 0.304163930034967 turquoise

ELAVL4 -0.554172485364712 0.554172485364712 0.00233730516359458 9.50676183848354e-05 9.50676183848354e-05 0.987986225766599 blue

RAB44 -0.552331449372187 0.552331449372187 -0.186099004815922 0.000101409075918465 0.000101409075918465 0.226469429642621 blue

MRO -0.54866594926187 0.54866594926187 -0.193529908630736 0.000115193126410005 0.000115193126410005 0.208133051955219 blue

SEMA6D -0.534209825464989 0.534209825464989 0.268959817556979 0.00018774800253405 0.00018774800253405 0.0774950940934275 grey

ARSF -0.541670771509309 0.541670771509309 -0.173070440107006 0.00014631510183263 0.00014631510183263 0.261236475363826 grey

NMUR2 -0.536782168007944 0.536782168007944 -0.00657626748448412 0.000172395911774918 0.000172395911774918 0.966206425375077 blue

CELA2B -0.547943111137594 0.547943111137594 -0.101983363125927 0.000118104323414063 0.000118104323414063 0.510070991775937 blue

MYH15 -0.544493517443406 0.544493517443406 0.0215473040249092 0.000132939386828311 0.000132939386828311 0.889584502355103 blue

RNF152 -0.55067104418545 0.55067104418545 -0.0304274469339934 0.000107455604220983 0.000107455604220983 0.844557168814277 brown

GAL3ST3 -0.535544088433302 0.535544088433302 0.0415513663460321 0.000179636983581765 0.000179636983581765 0.788852081434365 turquoise

TSPAN7 -0.527555326458313 0.527555326458313 0.759633288160207 0.000233371098020842 0.000233371098020842 2.25104642430952e-09 yellow

XDH -0.547087637759685 0.547087637759685 -0.0535642572669329 0.000121635942943445 0.000121635942943445 0.729850424079775 blue

CTC1 -0.535355343570201 0.535355343570201 0.010642074284623 0.000180764757607849 0.000180764757607849 0.945338904005439 blue

SEL1L2 -0.535930431187706 0.535930431187706 -0.135707800445063 0.00017734838389581 0.00017734838389581 0.379758052108528 blue

TRIM36 -0.552567698517201 0.552567698517201 0.0310495599094324 0.000100574356397058 0.000100574356397058 0.84141927630274 turquoise

ZNF485 -0.540336888960267 0.540336888960267 -0.0952993193692449 0.000153050757180203 0.000153050757180203 0.538324353687143 blue

SGSM1 -0.539374571385296 0.539374571385296 0.0642820627706364 0.000158083365769253 0.000158083365769253 0.678469252758745 turquoise

CNTN5 -0.537159776147358 0.537159776147358 0.129339840270641 0.000170240645302502 0.000170240645302502 0.402723170617061 grey

AWAT1 -0.531956029748859 0.531956029748859 -0.18124590238745 0.000202204889411945 0.000202204889411945 0.239027558544816 blue

CCR1 -0.532762559159834 0.532762559159834 0.544687344775455 0.000196919614642643 0.000196919614642643 0.000132062980922351 grey

DEFB136 -0.544140852477926 0.544140852477926 -0.235346714740332 0.000134547539205388 0.000134547539205388 0.124082132584404 blue

IFIH1 -0.535734576334519 0.535734576334519 0.725344131616866 0.000178505258685884 0.000178505258685884 2.55652054105027e-08 yellow

RERGL -0.532512723507333 0.532512723507333 0.307854345909544 0.000198543291288758 0.000198543291288758 0.0420570190567444 blue

INMT -0.546268928263342 0.546268928263342 -0.0622563971747266 0.000125105311684411 0.000125105311684411 0.688078862167468 blue

ALAS2 -0.530330688631252 0.530330688631252 0.315544117736668 0.000213247710423706 0.000213247710423706 0.036936781548068 grey

STMN2 -0.527838452635415 0.527838452635415 0.111707597095485 0.000231242366011347 0.000231242366011347 0.470345586527111 blue

SP7 -0.537414688649022 0.537414688649022 0.258047358230626 0.000168799519079084 0.000168799519079084 0.0908000897073658 turquoise

OR2L5 -0.532875906366174 0.532875906366174 0.039701163772917 0.000196186943985113 0.000196186943985113 0.798053376795494 blue

TRIM64 -0.543096020767344 0.543096020767344 0.312959051495791 0.00013941613750453 0.00013941613750453 0.0385976489513566 blue

OR6B2 -0.523080349603981 0.523080349603981 -0.0557559056247952 0.000269454356077211 0.000269454356077211 0.719239388789727 grey

KRTAP19-4 -0.529563279980926 0.529563279980926 0.0727994308265934 0.000218648773666234 0.000218648773666234 0.638627267600759 blue

ZSCAN12 -0.534892759530099 0.534892759530099 -0.21781975963101 0.000183555917213447 0.000183555917213447 0.15549776679999 blue

CCL18 -0.521787497039723 0.521787497039723 0.515498469831586 0.00028077808282314 0.00028077808282314 0.000342229439431638 yellow

IQCC -0.524368411937059 0.524368411937059 0.0991141902111809 0.000258584239638952 0.000258584239638952 0.5221066860516 turquoise

ACTR3C -0.532674601065526 0.532674601065526 0.00332162872114127 0.000197489876711672 0.000197489876711672 0.982927425834627 turquoise

OR9A2 -0.530141586348703 0.530141586348703 -0.228751328367241 0.000214567298273769 0.000214567298273769 0.135289110484013 blue

FRG2B -0.524712702176924 0.524712702176924 -0.237567408541513 0.000255746612331106 0.000255746612331106 0.120470335391185 blue

CPVL -0.525065962402365 0.525065962402365 0.43822045109393 0.000252864317229775 0.000252864317229775 0.00292773943723146 yellow

IL20RB -0.538972462821539 0.538972462821539 0.0652255448364227 0.000160230335017316 0.000160230335017316 0.674010473359428 blue

SYT17 -0.533086704826917 0.533086704826917 0.278265416571648 0.000194830910495854 0.000194830910495854 0.0674030848638902 blue

JAM2 -0.530085255545874 0.530085255545874 0.60835771010815 0.000214961809308793 0.000214961809308793 1.18253007763315e-05 yellow

ACKR2 -0.527392134871567 0.527392134871567 -0.0810390180487841 0.000234606112767002 0.000234606112767002 0.601017228173217 blue

FAM86C1 -0.541186422603657 0.541186422603657 -0.0317987102791291 0.000148729168909127 0.000148729168909127 0.837643840205137 blue

APEH -0.521402494724819 0.521402494724819 0.157685576330441 0.000284232230109088 0.000284232230109088 0.306653469753894 grey

KRTAP6-1 -0.522094024731461 0.522094024731461 0.00152698037645218 0.000278055130074849 0.000278055130074849 0.992151140235691 blue

GPRC5B -0.521862372460633 0.521862372460633 0.0429953952850332 0.000280110735928604 0.000280110735928604 0.781690343167076 blue

ANKS4B -0.527786196808028 0.527786196808028 0.0257736471552949 0.000231633934520463 0.000231633934520463 0.868102830516064 blue

SRGN -0.518153664704761 0.518153664704761 0.831997799636311 0.000314942720339152 0.000314942720339152 2.60830670903041e-12 yellow

LCE5A -0.52235693953517 0.52235693953517 -0.124531219382489 0.000275738614470407 0.000275738614470407 0.420584003302715 blue

S1PR1 -0.519379032172264 0.519379032172264 0.650954014664288 0.000303024686193733 0.000303024686193733 1.71630975197549e-06 yellow

PROKR2 -0.538684089328175 0.538684089328175 -0.049221329704088 0.000161786275644914 0.000161786275644914 0.751023231674896 blue

CARD18 -0.525270341579361 0.525270341579361 -0.00634015973984969 0.000251210191800999 0.000251210191800999 0.967419053882662 blue

SLC26A8 -0.520897155438568 0.520897155438568 -0.0551274651136176 0.00028882420086475 0.00028882420086475 0.722276803903848 blue

MED12L -0.520407580000899 0.520407580000899 0.231064290045019 0.00029333665905936 0.00029333665905936 0.131276209422186 grey

SLC25A33 -0.535982505494286 0.535982505494286 -0.161731780528807 0.000177041936446036 0.000177041936446036 0.294248283123575 blue

ADORA3 -0.528573840541213 0.528573840541213 0.089288295007758 0.000225794979368862 0.000225794979368862 0.564362255378714 blue

F13A1 -0.516625756561335 0.516625756561335 0.788146788527311 0.000330394591881366 0.000330394591881366 2.1502700576709e-10 yellow

ANP32D -0.538707920793205 0.538707920793205 0.119719419978591 0.000161657174096286 0.000161657174096286 0.438896934243029 blue

CYP4A22 -0.53859328415121 0.53859328415121 -0.0866111148798139 0.000162279048806727 0.000162279048806727 0.576143899927834 blue

CYP2C19 -0.523538001932124 0.523538001932124 -0.124067263230527 0.000265545809135951 0.000265545809135951 0.422330668270395 blue

TRIML2 -0.526445789191743 0.526445789191743 -0.154949434331399 0.000241885125285695 0.000241885125285695 0.315228605435603 blue

OTOP1 -0.535269375079996 0.535269375079996 -0.187256138978922 0.000181280552010746 0.000181280552010746 0.223543434432536 blue

WFDC9 -0.525860661544965 0.525860661544965 -0.0724593108696087 0.000246487229819762 0.000246487229819762 0.640200063435787 blue

COCH -0.523653414279307 0.523653414279307 0.156328309907796 0.0002645682558617 0.0002645682558617 0.310888379623039 blue

SRR -0.531100321739823 0.531100321739823 0.269805418474944 0.000207952470572134 0.000207952470572134 0.0765317146875765 brown

RNF121 -0.517215393821255 0.517215393821255 -0.128540171824677 0.000324352367478845 0.000324352367478845 0.405662635531408 blue

FCER1A -0.520325992808197 0.520325992808197 0.474418080170218 0.000294094807770185 0.000294094807770185 0.00113985136865479 yellow

C11orf96 -0.514347373537952 0.514347373537952 0.509587713058986 0.000354707121242757 0.000354707121242757 0.000410767626108886 brown

CD34 -0.514327213918073 0.514327213918073 0.489287623015384 0.000354929252501502 0.000354929252501502 0.000750246905886619 yellow

N6AMT1 -0.531845277029569 0.531845277029569 -0.147134141255732 0.000202940600264731 0.000202940600264731 0.340548862736484 blue

SMTNL2 -0.518187535002496 0.518187535002496 -0.118778589514651 0.000314607696004647 0.000314607696004647 0.442528451664151 blue

EGR1 -0.512732429636998 0.512732429636998 0.553127284139104 0.000372903777894061 0.000372903777894061 9.86220810354991e-05 brown

S100A5 -0.52393816235723 0.52393816235723 -0.14456918022479 0.00026217029678851 0.00026217029678851 0.349125040529318 blue

CYP4X1 -0.532664597558047 0.532664597558047 -0.00657602092457655 0.000197554827391773 0.000197554827391773 0.966207691646155 blue

KRTAP20-3 -0.533368973719268 0.533368973719268 -0.184888792611983 0.000193028403897647 0.000193028403897647 0.229557698358588 turquoise

KCTD14 -0.527103400408629 0.527103400408629 -0.0795380076961466 0.000236805700926649 0.000236805700926649 0.607796603740563 blue

UGT2B11 -0.51543178620009 0.51543178620009 0.112188585513432 0.000342941339254312 0.000342941339254312 0.468424390365184 blue

PRSS3 -0.523038524892146 0.523038524892146 -0.0774421118248283 0.000269814134472633 0.000269814134472633 0.61731734010519 blue

LMO2 -0.51507080767743 0.51507080767743 0.818685775980743 0.000346818280926525 0.000346818280926525 1.12683501963648e-11 yellow

MYO16 -0.517056481384677 0.517056481384677 0.174487892634879 0.000325970915794566 0.000325970915794566 0.257290635692614 grey

LHX9 -0.526598926868191 0.526598926868191 0.108099370115931 0.000240693567391448 0.000240693567391448 0.484890626645138 blue

OR8G1 -0.515596770902939 0.515596770902939 0.0753512430778133 0.000341182426979111 0.000341182426979111 0.626877405543884 blue

UCK2 -0.510838623282081 0.510838623282081 0.162550690059374 0.00039531038071453 0.00039531038071453 0.291777638002936 grey

MFAP4 -0.511958903378221 0.511958903378221 0.575178236285123 0.000381913789770939 0.000381913789770939 4.42641211360271e-05 yellow

DIO1 -0.526377751679004 0.526377751679004 -0.231894639975066 0.000242416230398407 0.000242416230398407 0.129857496121618 blue

HMGN4 -0.511317857399987 0.511317857399987 0.142576531253231 0.00038952866479255 0.00038952866479255 0.355878018223721 grey

UGT3A2 -0.524113934231601 0.524113934231601 -0.199320144345641 0.000260699877535821 0.000260699877535821 0.194583429663532 blue

CBLN2 -0.527227503301928 0.527227503301928 -0.080453157988292 0.000235858007605475 0.000235858007605475 0.603659373778692 blue

CHST7 -0.524383832894647 0.524383832894647 -0.0687284323717547 0.000258456534920541 0.000258456534920541 0.657553618935279 blue

TECTA -0.513814792910269 0.513814792910269 0.0108844956915726 0.000360617666642859 0.000360617666642859 0.944095694031308 turquoise

USP41 -0.509087737465917 0.509087737465917 -0.192025265596737 0.000417096312551576 0.000417096312551576 0.211759385700851 blue

DSG1 -0.518995845541977 0.518995845541977 0.0437493720573377 0.00030670712403225 0.00030670712403225 0.77795799215457 blue

PSENEN -0.525080925314884 0.525080925314884 0.0121728207225576 0.000252742883239004 0.000252742883239004 0.937491047256478 blue

HAVCR1 -0.52035860687788 0.52035860687788 -0.0781184716198486 0.000293791529799042 0.000293791529799042 0.614238053376968 blue

SLC44A3 -0.515238468779212 0.515238468779212 0.276998773174219 0.000345012701356041 0.000345012701356041 0.0687120318668797 brown

OR4C13 -0.527585325932268 0.527585325932268 0.0553272143794302 0.000233144705982314 0.000233144705982314 0.721310904117096 blue

ASRGL1 -0.521111872006888 0.521111872006888 -0.114490345243546 0.000286864975020852 0.000286864975020852 0.459288780636451 blue

ABCC9 -0.506619772094089 0.506619772094089 0.601472399967808 0.000449639448536266 0.000449639448536266 1.57416890139366e-05 yellow

PMEL -0.515592537157718 0.515592537157718 -0.0712438449687823 0.000341227461383913 0.000341227461383913 0.645833370533243 blue

SLC18A1 -0.524420677762693 0.524420677762693 -0.180056526729823 0.000258151644155091 0.000258151644155091 0.24217608871067 blue

UPP2 -0.513203239691538 0.513203239691538 0.0779213268731928 0.000367513947200464 0.000367513947200464 0.615134927632211 blue

RSPH10B -0.519194042697458 0.519194042697458 0.00594215398411084 0.000304797433418122 0.000304797433418122 0.969463339243031 blue

EIF4B -0.519424411687948 0.519424411687948 0.170746436635339 0.000302591238954033 0.000302591238954033 0.267792764252377 brown

NAT10 -0.520233383689123 0.520233383689123 -0.126582885321538 0.000294957519567016 0.000294957519567016 0.412909236975785 blue

CD84 -0.514287364387992 0.514287364387992 0.846023095900855 0.000355368708159505 0.000355368708159505 4.83121312589402e-13 yellow

CFI -0.503345115226767 0.503345115226767 0.800938657200295 0.000496338866014163 0.000496338866014163 6.65235583808863e-11 yellow

TBC1D28 -0.509803410832761 0.509803410832761 -0.141285619507384 0.000408064102649722 0.000408064102649722 0.360294917143764 blue

REG1B -0.517628956437614 0.517628956437614 -0.227126851890543 0.000320174072677242 0.000320174072677242 0.138161631773937 blue

OR2J2 -0.507362090315802 0.507362090315802 -0.0848875276091073 0.000439618993463614 0.000439618993463614 0.583787689901665 blue

RHOB -0.505665886416139 0.505665886416139 0.399762557817036 0.000462817079643296 0.000462817079643296 0.00717722664093065 brown

BEND4 -0.514959707160701 0.514959707160701 0.072664947943193 0.000348019432324243 0.000348019432324243 0.639248961772868 turquoise

C1orf115 -0.512754316118419 0.512754316118419 -0.101401595623493 0.000372651652789581 0.000372651652789581 0.512500048074187 blue

NCMAP -0.514570286627295 0.514570286627295 -0.119174671582862 0.000352259260551719 0.000352259260551719 0.440997597247867 blue

GRID2 -0.507089132928941 0.507089132928941 0.12709358227367 0.000443280024889041 0.000443280024889041 0.411011356945314 blue

C3 -0.519591219139923 0.519591219139923 0.426887538274614 0.000301002761709846 0.000301002761709846 0.00385439303577493 yellow

FOLR2 -0.509358137461872 0.509358137461872 0.850801341367688 0.000413662785260302 0.000413662785260302 2.61716326210737e-13 yellow

CWF19L1 -0.517499128931105 0.517499128931105 0.095153823550201 0.000321480486425136 0.000321480486425136 0.538947667221507 blue

C1orf54 -0.503905922121928 0.503905922121928 0.682806876999782 0.000488044570160795 0.000488044570160795 3.29061980521483e-07 yellow

IGLL5 -0.500984005882882 0.500984005882882 0.461940916043385 0.000532665581466111 0.000532665581466111 0.00159628103070005 grey

ENPP5 -0.505264273124357 0.505264273124357 0.471219967392862 0.00046846830759465 0.00046846830759465 0.00124411929118567 grey

HMGB3 -0.515207111232531 0.515207111232531 -0.160923782917332 0.000345349752440004 0.000345349752440004 0.296699215885466 blue

GIMAP8 -0.513701813830814 0.513701813830814 0.115961151196438 0.000361882848002444 0.000361882848002444 0.453501963815305 blue

CLEC17A -0.50892564969542 0.50892564969542 -0.223764025624877 0.000419166764632251 0.000419166764632251 0.144251573372599 blue

NMUR1 -0.517094099478177 0.517094099478177 -0.0350369935910012 0.000325587113200559 0.000325587113200559 0.821366553308436 blue

ADH1C -0.50869104868806 0.50869104868806 0.333749825887072 0.00042217985038645 0.00042217985038645 0.0268270727894194 blue

LAMA1 -0.503759026408237 0.503759026408237 0.109513501862121 0.000490205024737529 0.000490205024737529 0.479162342795783 grey

DRD5 -0.512055005130218 0.512055005130218 -0.223565721907828 0.000380783838495704 0.000380783838495704 0.144616785637886 blue

SYCN -0.506106904092288 0.506106904092288 -0.183837991700413 0.000456682023032192 0.000456682023032192 0.232262492914864 blue

PGBD4 -0.513702016637064 0.513702016637064 0.0290592627986199 0.000361880573326209 0.000361880573326209 0.851466493188042 blue

CTSG -0.51726713504262 0.51726713504262 0.237223911833048 0.00032382694056916 0.00032382694056916 0.121023761671043 turquoise

TRIM50 -0.518445077867747 0.518445077867747 0.1670151559406 0.000312070732310883 0.000312070732310883 0.278545272892206 blue

CD1E -0.513777550140974 0.513777550140974 -0.111212077159252 0.000361034284023874 0.000361034284023874 0.472329202718861 blue

LGALS13 -0.50864166004424 0.50864166004424 -0.0755615433262304 0.000422816647654578 0.000422816647654578 0.625913079860146 blue

HESX1 -0.514605512989566 0.514605512989566 0.166809755309352 0.000351873827195803 0.000351873827195803 0.27914528495011 turquoise

ZNF705G -0.509872909040833 0.509872909040833 -0.0630699240508823 0.000407196438935773 0.000407196438935773 0.684213612200007 blue

HBM -0.505016185201537 0.505016185201537 0.0794872049690311 0.000471990138280051 0.000471990138280051 0.608026630391929 blue

C1orf50 -0.51243302095026 0.51243302095026 -0.00676850738750245 0.000376368324672952 0.000376368324672952 0.96521915372648 blue

OAS1 -0.511308517076976 0.511308517076976 0.0257919175548874 0.000389640619818059 0.000389640619818059 0.868010157770688 blue

IGSF21 -0.502690158746077 0.502690158746077 0.712547183599107 0.000506185738478857 0.000506185738478857 5.78355004982564e-08 yellow

TFPI -0.500006597539833 0.500006597539833 0.756889532219482 0.00054838914751515 0.00054838914751515 2.77411644428275e-09 yellow

MRGPRX3 -0.507244916401745 0.507244916401745 -0.211188340629794 0.0004411872410213 0.0004411872410213 0.168786159895243 blue

TMEM81 -0.50455307875753 0.50455307875753 -0.142268109650587 0.000478628064258134 0.000478628064258134 0.356930289249533 blue

LAD1 -0.509856719238878 0.509856719238878 0.171074903801484 0.000407398415187686 0.000407398415187686 0.266859566615504 blue

LELP1 -0.507544025249657 0.507544025249657 -0.167359116151464 0.000437193920324517 0.000437193920324517 0.277542398772096 blue

DEFB119 -0.509528414616457 0.509528414616457 -0.0718034966734382 0.000411513682418806 0.000411513682418806 0.64323710191418 blue

FCRL4 -0.50365551094973 0.50365551094973 -0.0208747553717039 0.000491732624109416 0.000491732624109416 0.893010558859225 turquoise

OR5M11 -0.512101955517487 0.512101955517487 -0.145214350466246 0.000380232897381454 0.000380232897381454 0.346955516109092 blue

CES1 -0.505704802928716 0.505704802928716 0.508733287892632 0.000462272742470396 0.000462272742470396 0.000421635919612106 turquoise

BSPH1 -0.505742070217154 0.505742070217154 -0.17613601712445 0.000461752012888444 0.000461752012888444 0.252753023817374 blue

ACVRL1 -0.499375269524557 0.499375269524557 0.195646046638909 0.000558765439523175 0.000558765439523175 0.203106728008075 blue

KRT76 -0.507341537067358 0.507341537067358 -0.0380292717733811 0.000439893713707659 0.000439893713707659 0.806391267045242 turquoise

GRXCR1 -0.496590470244387 0.496590470244387 0.0947939127555841 0.000606668967840262 0.000606668967840262 0.540491044688197 grey

RAB19 -0.509539543435189 0.509539543435189 -0.251986324382999 0.000411373573653819 0.000411373573653819 0.0989189202370084 blue

ZFP37 -0.498625499045781 0.498625499045781 0.236104264312099 0.000571316985593006 0.000571316985593006 0.122840997429989 brown

CELA1 -0.508410424491364 0.508410424491364 0.205860788435744 0.00042580961210814 0.00042580961210814 0.180042096967615 turquoise

OR10H1 -0.500712748980876 0.500712748980876 -0.13598663638021 0.000536988207255458 0.000536988207255458 0.378770550954257 blue

SYT16 -0.501823653102562 0.501823653102562 0.142602532032828 0.000519482592375654 0.000519482592375654 0.355789395175847 grey

SMU1 -0.50581887750349 0.50581887750349 0.184184354446115 0.000460680460083659 0.000460680460083659 0.23136854683438 brown

CNGA3 -0.506323218620712 0.506323218620712 0.197925086012882 0.000453699645598066 0.000453699645598066 0.197789433602462 blue

RNASE11 -0.510803522608938 0.510803522608938 0.00862230042925434 0.000395736872629343 0.000395736872629343 0.955701623971734 blue

UGT1A8 -0.49576648360043 0.49576648360043 -0.0535627436337761 0.000621532083750148 0.000621532083750148 0.729857769970109 blue

KCNS1 -0.501315911364836 0.501315911364836 -0.0201732927875423 0.000527418988598023 0.000527418988598023 0.896585957315799 blue

AHR -0.491445118771124 0.491445118771124 0.854244469426546 0.000704957027958987 0.000704957027958987 1.66033539929345e-13 yellow

LTB4R -0.50448096719469 0.50448096719469 -0.0269295389762634 0.000479669186485417 0.000479669186485417 0.862243304591576 blue

FHDC1 -0.497555099136487 0.497555099136487 0.168378001598192 0.000589674348150488 0.000589674348150488 0.274585589801177 grey

ZNF705B -0.498564191907524 0.498564191907524 -0.00135549885298975 0.0005723543982098 0.0005723543982098 0.993032552591734 turquoise

NXPH1 -0.49049501861854 0.49049501861854 0.165990676907515 0.000724590797142181 0.000724590797142181 0.281546378471401 turquoise

DNAJC17 -0.503107182813268 0.503107182813268 -0.102582034672499 0.000499895958143561 0.000499895958143561 0.507577430492687 blue

SCGB1D1 -0.498708755145203 0.498708755145203 -0.0856029180478193 0.000569910862693868 0.000569910862693868 0.580609541983732 blue

APOE -0.487380250469575 0.487380250469575 0.666208225863735 0.000792435303524196 0.000792435303524196 7.9762519918284e-07 yellow

ARHGAP25 -0.506811673770773 0.506811673770773 -0.00344083135521874 0.000447029506070464 0.000447029506070464 0.98231484077196 blue

LGALS16 -0.495668801291183 0.495668801291183 -0.0743747478335949 0.000623315545106574 0.000623315545106574 0.63136313148327 blue

NAPEPLD -0.491348500069059 0.491348500069059 0.479431039862537 0.000706931663552777 0.000706931663552777 0.000992019951724941 grey

MMP26 -0.499228623199579 0.499228623199579 0.128149003432038 0.000561200733801799 0.000561200733801799 0.407105000624546 blue

SMNDC1 -0.489897567375584 0.489897567375584 0.491110385730222 0.00073718629786293 0.00073718629786293 0.000711819254124395 brown

LIN7A -0.499166994604909 0.499166994604909 0.226977615792898 0.000562227012593941 0.000562227012593941 0.13842777640095 blue

ERVW-1 -0.503840634593478 0.503840634593478 -0.202333325074053 0.000489003722022789 0.000489003722022789 0.18778458622934 blue

MCTP1 -0.495309980606082 0.495309980606082 0.393968690381494 0.000629906244942472 0.000629906244942472 0.00814438738977516 blue

SLC4A9 -0.495868706043692 0.495868706043692 -0.199606410692029 0.000619670628081834 0.000619670628081834 0.193930126898302 blue

TRIM49B -0.493730559808354 0.493730559808354 0.157269618428281 0.000659665072461627 0.000659665072461627 0.307947398238071 blue

DCAF12L1 -0.497391903373493 0.497391903373493 0.0687606472449416 0.000592519145327678 0.000592519145327678 0.657402995998247 blue

TGM4 -0.502951770740177 0.502951770740177 0.0472524242455569 0.000502231714061738 0.000502231714061738 0.760683035780056 grey

DGKK -0.488345739659256 0.488345739659256 -0.172576486871932 0.00077082211206464 0.00077082211206464 0.262620942922721 blue

FNDC7 -0.490382083447188 0.490382083447188 0.079322982391756 0.000726956836679729 0.000726956836679729 0.608770459772096 grey

OR52K1 -0.499895175071722 0.499895175071722 -0.21713146206516 0.000550207787748572 0.000550207787748572 0.156840282769052 blue

EMB -0.488506384118776 0.488506384118776 0.816296880459502 0.000767277575764788 0.000767277575764788 1.44689725016919e-11 yellow

LPL -0.485068225522592 0.485068225522592 0.574868228997519 0.000846409472078748 0.000846409472078748 4.47833952512571e-05 yellow

FMO4 -0.50045647538769 0.50045647538769 0.116651097626277 0.000541100960268665 0.000541100960268665 0.450801128396602 brown

KRT4 -0.500832037514067 0.500832037514067 -0.152074757194564 0.00053508341666856 0.00053508341666856 0.32439981513958 turquoise

FZD4 -0.485184418171989 0.485184418171989 0.627423087871011 0.000843620524416553 0.000843620524416553 5.16546444914639e-06 yellow

GGT5 -0.484814133424068 0.484814133424068 0.557584354254172 0.000852537112568297 0.000852537112568297 8.42603023062535e-05 yellow

CCL3 -0.484371045941999 0.484371045941999 0.549319067315561 0.00086331739804587 0.00086331739804587 0.000112618941006613 yellow

OR5D16 -0.499109869381895 0.499109869381895 0.0616532013457994 0.000563179801613249 0.000563179801613249 0.69094986700662 blue

BCO2 -0.49354054216689 0.49354054216689 -0.0104924949605552 0.00066332886738132 0.00066332886738132 0.946106056018538 blue

SERPINB12 -0.495320470707678 0.495320470707678 0.0401193567782869 0.00062971268355538 0.00062971268355538 0.795971234798427 blue

OR4N4 -0.491246419010933 0.491246419010933 -0.192586230639414 0.000709023312655301 0.000709023312655301 0.210402296581352 blue

MYZAP -0.496272271072495 0.496272271072495 0.346328884824114 0.000612370471990388 0.000612370471990388 0.021282687364106 grey

NBPF6 -0.49838210799387 0.49838210799387 -0.143934972287749 0.000575445511318114 0.000575445511318114 0.351265774717547 blue

C1QTNF9 -0.497341905998442 0.497341905998442 -0.0134817115111177 0.00059339314910987 0.00059339314910987 0.930785213845217 blue

SIGLECL1 -0.499861014326496 0.499861014326496 -0.199153356124634 0.000550766443151182 0.000550766443151182 0.194964782076343 blue

PAX1 -0.488293324749638 0.488293324749638 -0.0359002700018808 0.000771981783989168 0.000771981783989168 0.817039469678098 grey

NPHS1 -0.497156938816981 0.497156938816981 -0.256509077448671 0.000596636616753173 0.000596636616753173 0.0928099674146827 blue

KCTD1 -0.487435618278026 0.487435618278026 0.0724590202706338 0.000791181363697053 0.000791181363697053 0.640201407902368 turquoise

NCR3LG1 -0.492572820850932 0.492572820850932 -0.272036048383824 0.000682272103911848 0.000682272103911848 0.0740355142625042 blue

CDH17 -0.491590541934203 0.491590541934203 -0.0521036795751036 0.000701994262737852 0.000701994262737852 0.736949849526491 blue

PRKACG -0.49429726866975 0.49429726866975 0.118216405652318 0.000648845560728491 0.000648845560728491 0.444706309159744 blue

TANGO6 -0.489955327765334 0.489955327765334 -0.0794020069260728 0.000735960093897858 0.000735960093897858 0.608412477483883 blue

NOSTRIN -0.494258464299789 0.494258464299789 0.500598649130003 0.000649581305220196 0.000649581305220196 0.000538815837256978 yellow

XAGE3 -0.485104669967466 0.485104669967466 -0.0457992720245891 0.000845533818426349 0.000845533818426349 0.767835690145039 blue

SPARCL1 -0.478923105405369 0.478923105405369 0.738750914156224 0.00100617773499741 0.00100617773499741 1.03393346663535e-08 yellow

HYPM -0.486573634741543 0.486573634741543 -0.146819151216047 0.00081090567385092 0.00081090567385092 0.341594995020455 blue

TMEM97 -0.495427640113456 0.495427640113456 -0.0818545954489677 0.00062773826534593 0.00062773826534593 0.597347497739288 blue

MAS1L -0.494405295881928 0.494405295881928 -0.181470992828712 0.000646801257211674 0.000646801257211674 0.23843484282554 turquoise

CALN1 -0.487833205055364 0.487833205055364 -0.170603532350733 0.000782229018513196 0.000782229018513196 0.268199438987119 blue

KRTAP21-2 -0.482364010148491 0.482364010148491 0.0314698405838685 0.000913688864971613 0.000913688864971613 0.839300781361062 blue

IGFBP4 -0.479131006743487 0.479131006743487 0.662621721870774 0.00100036121361822 0.00100036121361822 9.58845135816603e-07 yellow

SPRY1 -0.489401016192742 0.489401016192742 0.508662022759919 0.000747803292277091 0.000747803292277091 0.000422553994412074 yellow

CEACAM6 -0.481599311569464 0.481599311569464 0.209045331726298 0.00093356057433244 0.00093356057433244 0.173251038670677 turquoise

EDEM1 -0.484546214706406 0.484546214706406 0.198735154179673 0.000859041087462749 0.000859041087462749 0.195923300789198 grey

HTR1F -0.491016226337543 0.491016226337543 0.260274375844599 0.00071376032121608 0.00071376032121608 0.0879502997464827 blue

HMGN5 -0.490312268410406 0.490312268410406 -0.0852351880553828 0.000728422946718132 0.000728422946718132 0.582242220413353 blue

LCN6 -0.493278334906739 0.493278334906739 0.177176211988556 0.000668414500552628 0.000668414500552628 0.249916991588765 grey

RGS5 -0.47816756169155 0.47816756169155 0.537136350299958 0.00102757060431619 0.00102757060431619 0.000170373638961146 yellow

CD209 -0.49405226350766 0.49405226350766 0.10945258436329 0.000653503496782421 0.000653503496782421 0.479408367760667 brown

HOXB1 -0.4914640277479 0.4914640277479 0.00811468701667727 0.000704571155695567 0.000704571155695567 0.958307204251254 blue

TTC38 -0.478505510308643 0.478505510308643 0.141422036875611 0.00101795212836573 0.00101795212836573 0.359826599753811 grey

FAM216B -0.491625683670331 0.491625683670331 0.0813448507466254 0.000701279980158063 0.000701279980158063 0.599639969066961 turquoise

MAP9 -0.489447144511183 0.489447144511183 0.112443657261377 0.000746811269837013 0.000746811269837013 0.467407267339771 blue

ADAM30 -0.484533783224922 0.484533783224922 -0.0732369795966658 0.000859343946286265 0.000859343946286265 0.636606240534077 blue

NNT -0.479035549626125 0.479035549626125 0.163790350419158 0.00100302811396711 0.00100302811396711 0.288063228510287 grey

ROPN1L -0.475286022021179 0.475286022021179 0.044752168680036 0.00111293105096843 0.00111293105096843 0.773001581618825 turquoise

THBS4 -0.48094650426908 0.48094650426908 0.392617805889383 0.000950828862132666 0.000950828862132666 0.00838539713088231 brown

ADAMTS9 -0.482369329970354 0.482369329970354 0.483999535361476 0.000913551955203436 0.000913551955203436 0.000872449891176621 yellow

CDC20B -0.488618533417109 0.488618533417109 -0.0776130860250666 0.000764811707530171 0.000764811707530171 0.616538324577492 blue

CAPN11 -0.483049618075913 0.483049618075913 -0.0228343642340735 0.000896194378671935 0.000896194378671935 0.883033627875714 blue

RABIF -0.475513644805849 0.475513644805849 0.0718818548248003 0.00110596544071352 0.00110596544071352 0.642873926151534 grey

HMBS -0.47584142223663 0.47584142223663 -0.109504106107147 0.00109600316455866 0.00109600316455866 0.479200284707 blue

SPAM1 -0.474979771408282 0.474979771408282 -0.0252438118286838 0.00112236444017654 0.00112236444017654 0.870791063284337 grey

HBG1 -0.478935351024449 0.478935351024449 0.438725767417742 0.00100583430145039 0.00100583430145039 0.0028914323705454 turquoise

MKRN1 -0.482917007056052 0.482917007056052 0.0831268934478463 0.000899554663977026 0.000899554663977026 0.591642430727382 blue

RBMS3 -0.478476833717081 0.478476833717081 0.410921600553956 0.00101876517490771 0.00101876517490771 0.00559127699578141 brown

RPE65 -0.488548721850042 0.488548721850042 0.114305561089877 0.000766345846172075 0.000766345846172075 0.460018612675574 grey

DCD -0.488870498470691 0.488870498470691 0.190160389597489 0.0007592974896824 0.0007592974896824 0.21631468469811 turquoise

TTLL13P -0.479843020792639 0.479843020792639 -0.0579737861538231 0.00098066748768995 0.00098066748768995 0.708554190487993 turquoise

CALCRL -0.477260965898847 0.477260965898847 0.508897263712708 0.00105377499513855 0.00105377499513855 0.000419530306955759 yellow

IL22 -0.484657711118547 0.484657711118547 -0.0379769178641772 0.000856329043689965 0.000856329043689965 0.806652709097005 turquoise

ALDH1A1 -0.480618515516979 0.480618515516979 0.613260485764893 0.000959612039707593 0.000959612039707593 9.60630456017431e-06 yellow

OR13C2 -0.479716349391096 0.479716349391096 0.113573629016364 0.000984145618947313 0.000984145618947313 0.462915621833853 grey

FKBP1B -0.481017708165279 0.481017708165279 0.163688177458147 0.000948931603591762 0.000948931603591762 0.288368203145352 brown

OR9G1 -0.478628746918781 0.478628746918781 0.161849474249007 0.00101446467180331 0.00101446467180331 0.293892372640687 blue

TSPAN11 -0.472083702627695 0.472083702627695 0.529764400156782 0.00121515432999882 0.00121515432999882 0.000217221446745805 grey

IRX3 -0.471751130855802 0.471751130855802 0.635217713540553 0.00122623515345472 0.00122623515345472 3.62266806059145e-06 yellow

RGS16 -0.474340922963081 0.474340922963081 0.303744223614305 0.00114227233068681 0.00114227233068681 0.0450225581879288 brown

SLC22A5 -0.481370292383599 0.481370292383599 0.231570738089076 0.000939586531870978 0.000939586531870978 0.130409535269045 brown

MGST2 -0.471744629272178 0.471744629272178 0.742556290954952 0.0012264526700357 0.0012264526700357 7.91675980909426e-09 yellow

BMP10 -0.483855913754739 0.483855913754739 -0.0408612509029977 0.000876003465471242 0.000876003465471242 0.792280868064441 turquoise

RGS4 -0.479355773148607 0.479355773148607 0.250864776133393 0.000994106602320801 0.000994106602320801 0.100480627734908 brown

GOLGA6A -0.478255212266405 0.478255212266405 -0.158729406369928 0.0010250681997657 0.0010250681997657 0.303421716766056 blue

TLR7 -0.477833117887149 0.477833117887149 0.391425320033923 0.0010371690117226 0.0010371690117226 0.00860322982497214 yellow

ABCA4 -0.476195253855645 0.476195253855645 0.135031167213941 0.00108533881146565 0.00108533881146565 0.38216069165801 turquoise

IGSF5 -0.478999434016986 0.478999434016986 -0.0495561178476138 0.00100403877116145 0.00100403877116145 0.749384390164323 blue

ATP1A2 -0.477820745881693 0.477820745881693 0.1356614848282 0.00103752561134529 0.00103752561134529 0.379922227088556 blue

RGL1 -0.47485683603313 0.47485683603313 0.490309158039258 0.00112617118506995 0.00112617118506995 0.000728488325775069 brown

CD163L1 -0.473681560772611 0.473681560772611 0.713049318465438 0.00116314861135223 0.00116314861135223 5.60585298455733e-08 yellow

SLC5A11 -0.481971221574897 0.481971221574897 -0.297372860131755 0.00092384832852243 0.00092384832852243 0.0499534713677268 blue

CCDC3 -0.468395911456756 0.468395911456756 0.302981730867231 0.00134315724435578 0.00134315724435578 0.0455909601107517 brown

MYCL -0.484015044435976 0.484015044435976 -0.257588621520619 0.000872066927348396 0.000872066927348396 0.0913959001911365 blue

NFE2 -0.480956486562996 0.480956486562996 -0.0306292953785303 0.000950562675775052 0.000950562675775052 0.843538796976939 blue

IGLL1 -0.470023123040337 0.470023123040337 0.453723962357833 0.0012852697166399 0.0012852697166399 0.00197917143576672 grey

DEFB112 -0.474010093299742 0.474010093299742 0.305996251724525 0.00115270475829722 0.00115270475829722 0.043377317036878 blue

CHRM2 -0.474266279180827 0.474266279180827 -0.0923146782643089 0.00114461878510434 0.00114461878510434 0.55118005286594 blue

OR52B2 -0.477110373688718 0.477110373688718 -0.168587080099464 0.00105818494819067 0.00105818494819067 0.273981416891597 blue

PNPLA1 -0.48088297650546 0.48088297650546 -0.128066776806475 0.000952524443044881 0.000952524443044881 0.407408571439741 blue

C6orf201 -0.474490822427443 0.474490822427443 -0.0511198021578178 0.00113757311987572 0.00113757311987572 0.741744446720981 blue

TXNRD1 -0.479446303743585 0.479446303743585 0.315184122596409 0.000991597261493468 0.000991597261493468 0.0371644955684139 blue

HTR5A -0.478494536642148 0.478494536642148 -0.0361976770829354 0.0010182631883906 0.0010182631883906 0.81554998442937 blue

KIR2DL4 -0.481299876017838 0.481299876017838 -0.202975754448131 0.000941446292867792 0.000941446292867792 0.186357159360296 blue

PRSS12 -0.477911372100791 0.477911372100791 0.115142332768436 0.00103491601213795 0.00103491601213795 0.456718661348557 brown

PCSK5 -0.475030590149781 0.475030590149781 0.201536620534073 0.00112079416753863 0.00112079416753863 0.189565580342771 blue

OR9A4 -0.471839083550711 0.471839083550711 -0.0515933222810092 0.0012232959843142 0.0012232959843142 0.739435686185084 blue

CGNL1 -0.468622943408685 0.468622943408685 0.640564437540111 0.00133494402542093 0.00133494402542093 2.82388898832375e-06 yellow

TAAR8 -0.478516313529225 0.478516313529225 -0.0250695166340132 0.00101764598256935 0.00101764598256935 0.871675703307764 blue

TRIM43 -0.477704016047115 0.477704016047115 -0.0872847873659869 0.00104089551164848 0.00104089551164848 0.573168713995152 blue

CX3CR1 -0.464372426286417 0.464372426286417 0.724053726933949 0.00149635585851719 0.00149635585851719 2.78154712562757e-08 yellow

GZMK -0.478024171546319 0.478024171546319 0.327487347191159 0.00103167610515861 0.00103167610515861 0.0300067831518288 grey

NRXN2 -0.471836488129604 0.471836488129604 0.473371196363546 0.00122338262726071 0.00122338262726071 0.00117309220327647 yellow

IFI44L -0.466795775079775 0.466795775079775 0.592294782355537 0.00140232947261812 0.00140232947261812 2.2815916235532e-05 yellow

KRTAP5-7 -0.464328506091593 0.464328506091593 -0.183095535099378 0.00149811038270947 0.00149811038270947 0.23418669077806 blue

SLC36A2 -0.478783091835408 0.478783091835408 -0.126383652955034 0.00101011191380017 0.00101011191380017 0.413650989813555 blue

MAGEC3 -0.466859982738618 0.466859982738618 -0.236394284951971 0.00139991123762192 0.00139991123762192 0.122368322507803 blue

BSPRY -0.476096299745759 0.476096299745759 -0.238789421166287 0.00108831189937204 0.00108831189937204 0.118516953863432 blue

FAM155A -0.469271413558532 0.469271413558532 0.00628010104541468 0.00131172993649929 0.00131172993649929 0.967727521720771 blue

KIR3DL2 -0.477759169737081 0.477759169737081 -0.17393842228701 0.00103930205320177 0.00103930205320177 0.25881546838696 blue

CPE -0.461131760359586 0.461131760359586 0.593635394391995 0.00163082226914727 0.00163082226914727 2.1627193190602e-05 yellow

EBLN2 -0.477305241666099 0.477305241666099 -0.028681175642895 0.00105248154003013 0.00105248154003013 0.853377794117987 blue

CPA2 -0.465119440725082 0.465119440725082 -0.0267966485611079 0.00146679212578857 0.00146679212578857 0.862916596330014 blue

TRIM39-RPP21 -0.474598817210695 0.474598817210695 0.104027732280118 0.00113419825907991 0.00113419825907991 0.50158140700838 brown

NUP214 -0.468346005459212 0.468346005459212 -0.0499115316652209 0.00134496867746901 0.00134496867746901 0.747645775520979 blue

DOK2 -0.475552153885529 0.475552153885529 0.573665820338561 0.00110479085159694 0.00110479085159694 4.68505517846404e-05 yellow

NPR3 -0.471563058110731 0.471563058110731 0.208815240870406 0.00123254118624412 0.00123254118624412 0.173735433572174 blue

HHLA1 -0.473827285456198 0.473827285456198 -0.250293219375269 0.001158505759925 0.001158505759925 0.101283745871118 turquoise

FAM181A -0.46541786108422 0.46541786108422 -0.210196596453937 0.0014551275977588 0.0014551275977588 0.170841964548365 blue

NUDT10 -0.472092618474001 0.472092618474001 -0.117244820007362 0.00121485849682299 0.00121485849682299 0.448484011662566 blue

ERP27 -0.469046740926814 0.469046740926814 0.341853058066382 0.0013197318685027 0.0013197318685027 0.0231335154241317 grey

LCNL1 -0.465941942007018 0.465941942007018 -0.16252434068572 0.00143484173451769 0.00143484173451769 0.291856924077741 blue

KRTAP20-1 -0.468536057200076 0.468536057200076 0.00459508597182413 0.00133808197586557 0.00133808197586557 0.976383671824221 blue

PPARG -0.466349027144041 0.466349027144041 0.400660486900183 0.00141925818713212 0.00141925818713212 0.00703660195843429 yellow

PTPN22 -0.46715453549141 0.46715453549141 0.543187599550949 0.0013888649877786 0.0013888649877786 0.000138983105572693 yellow

COL14A1 -0.467452775164655 0.467452775164655 0.574775757265525 0.00137775942952746 0.00137775942952746 4.4939362812385e-05 yellow

CCDC126 -0.468837753229307 0.468837753229307 0.368039622064749 0.00132721408856793 0.00132721408856793 0.0139723224716991 grey

ARHGDIB -0.462295526208373 0.462295526208373 0.80406516904025 0.0015813490107567 0.0015813490107567 4.92986157102987e-11 yellow

ANGPT2 -0.472385915935167 0.472385915935167 0.193274962470464 0.00120516236786733 0.00120516236786733 0.208744423028124 blue

CDNF -0.458240184252551 0.458240184252551 0.103597110234516 0.00175973579504958 0.00175973579504958 0.503363621569384 grey

PSG2 -0.467381184683734 0.467381184683734 -0.028271547334696 0.00138041802532317 0.00138041802532317 0.855449477755212 blue

WNT8A -0.473517327414214 0.473517327414214 -0.0633060666842904 0.00116840100484981 0.00116840100484981 0.683093131284884 blue

MSL2 -0.459217050370444 0.459217050370444 0.301668740322142 0.0017152097334662 0.0017152097334662 0.0465833783784894 grey

TSPAN16 -0.466664332667528 0.466664332667528 -0.152575115810113 0.00140729152709413 0.00140729152709413 0.322791583573777 blue

SKIDA1 -0.470909437993568 0.470909437993568 -0.108582140764956 0.00125468187000299 0.00125468187000299 0.482931037495193 grey

GRM7 -0.455864706325496 0.455864706325496 0.297758408836654 0.00187230998166064 0.00187230998166064 0.0496431861106141 grey

ASCL4 -0.46411070814845 0.46411070814845 0.0994656537850327 0.00150683798085599 0.00150683798085599 0.520624831766048 blue

PIP4K2C -0.466905363103146 0.466905363103146 0.105872748052907 0.00139820432458749 0.00139820432458749 0.493982055541804 brown

RAC2 -0.462327381987212 0.462327381987212 0.539905514358576 0.00158001370279944 0.00158001370279944 0.000155288462889074 grey

DDX53 -0.462482553924351 0.462482553924351 0.0478447896051856 0.00157352360468306 0.00157352360468306 0.757772925911379 blue

OR2W1 -0.466547349533133 0.466547349533133 0.13113945582727 0.00141172084198231 0.00141172084198231 0.396153226566965 blue

MARS2 -0.463009147444686 0.463009147444686 -0.0961550851836069 0.00155167464176255 0.00155167464176255 0.534665267381938 grey

CT55 -0.462631963224041 0.462631963224041 -0.227195062203384 0.00156729688559351 0.00156729688559351 0.138040113503575 blue

RPL32 -0.460132630558566 0.460132630558566 0.17936321854196 0.00167438629612014 0.00167438629612014 0.244024336777653 brown

NR2F2 -0.454215726413739 0.454215726413739 0.665065842830059 0.0019541576659287 0.0019541576659287 8.46021473507473e-07 yellow

WFDC1 -0.467730377423667 0.467730377423667 -0.151190212248862 0.00136749331466902 0.00136749331466902 0.327255159264953 blue

OR9Q2 -0.464181535661803 0.464181535661803 -0.0869377389820625 0.00150399484314361 0.00150399484314361 0.574700530383379 blue

SOST -0.454584883361771 0.454584883361771 0.187909036177126 0.0019355644919179 0.0019355644919179 0.221904031607318 grey

OR12D2 -0.460518658173328 0.460518658173328 0.0320111936500897 0.00165743396726939 0.00165743396726939 0.836573655634844 turquoise

PRG4 -0.452027206462838 0.452027206462838 0.545902829137768 0.00206766521385862 0.00206766521385862 0.000126685565319962 yellow

HEYL -0.455076074776182 0.455076074776182 -0.0283228277402461 0.00191106755603953 0.00191106755603953 0.855190075666781 blue

SPATA31D3 -0.458184081081254 0.458184081081254 0.054650768764083 0.00176232381103832 0.00176232381103832 0.724583620484059 turquoise

OR4F29 -0.461318074598436 0.461318074598436 0.00629509114490642 0.00162281069905325 0.00162281069905325 0.967650530538944 blue

TIMD4 -0.454191651824446 0.454191651824446 0.694966914605173 0.00195537568900088 0.00195537568900088 1.65738465062991e-07 yellow

KRT20 -0.463486480991323 0.463486480991323 0.201289220203346 0.00153210252012214 0.00153210252012214 0.190121064556056 blue

ITIH1 -0.457562159241441 0.457562159241441 -0.0937516950875649 0.00179123941603627 0.00179123941603627 0.544972283169679 blue

KDR -0.465091203478652 0.465091203478652 0.391644390715037 0.00146790013831471 0.00146790013831471 0.00856285038901554 yellow

RPL21 -0.454084667915013 0.454084667915013 0.112712988518162 0.00196079652849351 0.00196079652849351 0.466334565225213 brown

NLRP10 -0.454197245098559 0.454197245098559 -0.0738996920473215 0.0019550926445864 0.0019550926445864 0.633550148552302 grey

RHBDL2 -0.458843262927293 0.458843262927293 0.0840461863781447 0.00173212785842041 0.00173212785842041 0.587535337558645 grey

TSC22D3 -0.449557459164294 0.449557459164294 0.370909526292234 0.0022027223102133 0.0022027223102133 0.0131888686701557 brown

ABCA12 -0.455202388919433 0.455202388919433 0.0301301577991449 0.00190481246350507 0.00190481246350507 0.846057516499808 blue

PRR20B -0.451514865203452 0.451514865203452 0.102972146846651 0.00209506475983251 0.00209506475983251 0.505955877699042 blue

MBD3L2 -0.452240591736282 0.452240591736282 0.0331547512258205 0.00205634729603674 0.00205634729603674 0.830819114264466 turquoise

PGM5 -0.460704652015642 0.460704652015642 0.114538236145129 0.00164932052555851 0.00164932052555851 0.45909973056798 blue

LAT2 -0.461169150599633 0.461169150599633 0.696839735805198 0.00162921167065828 0.00162921167065828 1.48681484181792e-07 yellow

SLC1A3 -0.462592339168919 0.462592339168919 0.361334515425652 0.00156894610783427 0.00156894610783427 0.0159583359905888 blue

SLC51A -0.45162271218646 0.45162271218646 0.0256517335240094 0.00208927069114291 0.00208927069114291 0.868721256333535 grey

SIX2 -0.454217514817961 0.454217514817961 -0.100219482889675 0.00195406721068412 0.00195406721068412 0.517453567538706 blue

AQP8 -0.456584124245971 0.456584124245971 -0.209132435321745 0.00183756231758682 0.00183756231758682 0.173067919374672 blue

PTH2R -0.454063889637179 0.454063889637179 0.0371483103791285 0.00196185089692026 0.00196185089692026 0.810793318231713 grey

NKG7 -0.454003112362062 0.454003112362062 0.236168459865521 0.00196493784462268 0.00196493784462268 0.122736253421838 blue

EVPLL -0.450008533016158 0.450008533016158 0.111130172330501 0.00217748928618369 0.00217748928618369 0.472657503119145 grey

MS4A12 -0.459553988800134 0.459553988800134 -0.162107951349342 0.00170008507751119 0.00170008507751119 0.293111704619056 blue

BMP3 -0.455236751068303 0.455236751068303 0.327743772927116 0.00190311398254385 0.00190311398254385 0.0298707197157283 blue

GABRA3 -0.452068694260782 0.452068694260782 0.34127947469297 0.00206546040705489 0.00206546040705489 0.0233801543106657 turquoise

SERPINA9 -0.451568492119949 0.451568492119949 -0.296832471321328 0.00209218188674842 0.00209218188674842 0.050391005356772 turquoise

SEMG2 -0.457076461900411 0.457076461900411 -0.0230712032025223 0.00181411274093218 0.00181411274093218 0.881828992007762 blue

CKMT1A -0.452298867720067 0.452298867720067 -0.22085040655499 0.00205326588192065 0.00205326588192065 0.149686426851937 blue

MRPS10 -0.454247795505731 0.454247795505731 0.20327613486069 0.0019525362134909 0.0019525362134909 0.185692395950923 blue

ZDHHC7 -0.447697084498796 0.447697084498796 -0.108411245141221 0.00230954067924859 0.00230954067924859 0.483624236612488 blue

CXXC4 -0.454031090690214 0.454031090690214 0.188392087490091 0.00196351626000268 0.00196351626000268 0.220696460692929 brown

CER1 -0.448744715488019 0.448744715488019 0.129517100291603 0.00224883939935618 0.00224883939935618 0.402073258728613 grey

ZNF502 -0.453354888085474 0.453354888085474 -0.0818254831272554 0.00199813010038056 0.00199813010038056 0.597478320925715 blue

PSG1 -0.458410865793795 0.458410865793795 -0.131221304898061 0.00175188301143377 0.00175188301143377 0.395855907140384 blue

GFPT2 -0.444760538771546 0.444760538771546 0.406986652592504 0.00248746273293023 0.00248746273293023 0.00611171494613603 brown

MS4A3 -0.446819388219055 0.446819388219055 -0.005855030765144 0.00236150392174565 0.00236150392174565 0.96991085832209 turquoise

OR7A17 -0.451823994189825 0.451823994189825 -0.129999589221514 0.00207849469674514 0.00207849469674514 0.400307319859546 blue

ZNF772 -0.457294860467404 0.457294860467404 -0.114853389952527 0.00180379572616331 0.00180379572616331 0.457856704754564 blue

HSPA1L -0.456779511973265 0.456779511973265 -0.113528064864494 0.00182822426692573 0.00182822426692573 0.463096289649678 blue

IRX6 -0.453053494493897 0.453053494493897 -0.0270447153096322 0.00201373104815803 0.00201373104815803 0.861659839574573 blue

ADCY10 -0.4547210393851 0.4547210393851 -0.17739150098162 0.00192874638252072 0.00192874638252072 0.249332705079082 blue

CACNG6 -0.449637597296594 0.449637597296594 -0.155135263125843 0.00219822062172584 0.00219822062172584 0.314641454330127 blue

DCAF8L2 -0.456137014088357 0.456137014088357 -0.122217333067725 0.0018590898011386 0.0018590898011386 0.429335734404803 grey

OR7E24 -0.444797586036932 0.444797586036932 -0.0971963534891332 0.00248514490324919 0.00248514490324919 0.530229382699897 blue

NR5A2 -0.453100577525005 0.453100577525005 0.321050364458107 0.00201128682835341 0.00201128682835341 0.0335941876968944 grey

CYP8B1 -0.45226859183761 0.45226859183761 -0.206145015158219 0.0020548662466157 0.0020548662466157 0.179428348849592 blue

QDPR -0.443020113671364 0.443020113671364 0.317641407871471 0.00259852104424719 0.00259852104424719 0.0356328026587975 grey

TIFAB -0.451592678637439 0.451592678637439 -0.184773563729838 0.00209088281647035 0.00209088281647035 0.229853242195365 blue

OR1D5 -0.448344021096901 0.448344021096901 0.0142415170171466 0.00227188786081318 0.00227188786081318 0.926894647899374 blue

GAB3 -0.450958357062628 0.450958357062628 -0.0332228123239671 0.00212518933621098 0.00212518933621098 0.83047689372293 blue

NPY1R -0.441737066390755 0.441737066390755 0.192293024496558 0.00268316752210923 0.00268316752210923 0.211110863637821 grey

HBD -0.43876237610315 0.43876237610315 0.497887080303902 0.00288881747842447 0.00288881747842447 0.000583925083442638 brown

SLC5A7 -0.453033717289876 0.453033717289876 -0.0359155439362525 0.0020147585244037 0.0020147585244037 0.81696295862755 blue

LSM3 -0.447514368443865 0.447514368443865 0.280990335452176 0.00232027429478813 0.00232027429478813 0.0646541893829872 brown

TEK -0.441919151162502 0.441919151162502 0.527719134885935 0.00267100894540327 0.00267100894540327 0.000232137327584382 yellow

RPL26 -0.442033485039172 0.442033485039172 0.325797381608989 0.00266339920126135 0.00266339920126135 0.0309164009143099 brown

TAS2R46 -0.447759454293923 0.447759454293923 0.272577297773342 0.0023058868220018 0.0023058868220018 0.0734396028542426 blue

ANKRD22 -0.447557827966945 0.447557827966945 -0.039713940225742 0.00231771729307955 0.00231771729307955 0.797989743502278 blue

SPDEF -0.450314207053227 0.450314207053227 -0.142576693980037 0.00216053534126551 0.00216053534126551 0.355877463531498 grey

AQP7 -0.451986438006957 0.451986438006957 -0.172772520868216 0.00206983381815525 0.00206983381815525 0.262070909489085 blue

ENPP2 -0.442871014802151 0.442871014802151 0.600531817771256 0.00260823522243096 0.00260823522243096 1.6360627146744e-05 yellow

TCEAL5 -0.450887158373135 0.450887158373135 0.0613407621667835 0.00212907089915955 0.00212907089915955 0.692438659045366 brown

KRTAP19-7 -0.449637171508236 0.449637171508236 -0.181595702306587 0.00219824451852723 0.00219824451852723 0.23810688440453 blue

SLC36A3 -0.440460926061636 0.440460926061636 -0.127804928925766 0.00276975685122488 0.00276975685122488 0.408376148528094 turquoise

GJA8 -0.450036525828992 0.450036525828992 0.160805934794302 0.00217593181723053 0.00217593181723053 0.29705778592811 brown

CEACAM18 -0.449225034599959 0.449225034599959 -0.103000838126441 0.00222148299747667 0.00222148299747667 0.505836722410975 blue

DUSP1 -0.437938475784573 0.437938475784573 0.549677948827766 0.00294817315058891 0.00294817315058891 0.000111226807353182 brown

CYGB -0.441667737280539 0.441667737280539 0.480940096615811 0.00268780971471861 0.00268780971471861 0.000950999762703992 yellow

HERC5 -0.446447567743267 0.446447567743267 0.0155871437961421 0.00238382638303065 0.00238382638303065 0.920008586262868 grey

CLIP4 -0.442283550539901 0.442283550539901 0.625804550430127 0.00264682203786517 0.00264682203786517 5.55364176080422e-06 grey

GPR174 -0.444678780075876 0.444678780075876 -0.181363285616269 0.00249258463743371 0.00249258463743371 0.238718336217015 blue

SBK1 -0.446939027268764 0.446939027268764 0.0184563980684175 0.0023543606342726 0.0023543606342726 0.905345479853566 grey

HOXA11 -0.444238802429902 0.444238802429902 -0.223200100256245 0.00252030766470898 0.00252030766470898 0.145291933535948 blue

NELL1 -0.447133879744583 0.447133879744583 0.369030628851819 0.00234276737468031 0.00234276737468031 0.0136974295780573 grey

IFITM10 -0.446866896011484 0.446866896011484 -0.0616651425320264 0.00235866508933415 0.00235866508933415 0.690892989320116 blue

TLX2 -0.443141277684693 0.443141277684693 -0.0742586801380985 0.00259065037965275 0.00259065037965275 0.631897186885318 blue

SDE2 -0.445714518709359 0.445714518709359 0.204358652970197 0.00242838115374171 0.00242838115374171 0.183310725876118 grey

ZNF543 -0.448074042007317 0.448074042007317 0.11167718848647 0.00228753472615724 0.00228753472615724 0.470467187408117 blue

RASSF9 -0.445555931255362 0.445555931255362 0.1098709128313 0.00243811615156061 0.00243811615156061 0.477720221100771 grey

PRSS58 -0.441585869041465 0.441585869041465 -0.100456373814703 0.0026933006153989 0.0026933006153989 0.516458991111779 blue

ZCWPW2 -0.442967598349859 0.442967598349859 0.281108674301104 0.00260193891208599 0.00260193891208599 0.0645368604201441 brown

OR4K15 -0.437482714394443 0.437482714394443 0.0973316957845035 0.00298146564650215 0.00298146564650215 0.529654139882337 blue

ORAI1 -0.440543819270112 0.440543819270112 0.32705307479625 0.00276405857619708 0.00276405857619708 0.0302383853932272 grey

IL13 -0.432423331043263 0.432423331043263 0.0584824509573107 0.0033738412212981 0.0033738412212981 0.706111256551466 grey

ACKR1 -0.432042447119836 0.432042447119836 0.51030320019694 0.00340513449392556 0.00340513449392556 0.00040186124090089 yellow

CCR4 -0.440346333474728 0.440346333474728 -0.149948953138326 0.00277765123804604 0.00277765123804604 0.331288418351655 blue

CXCR1 -0.440209052466485 0.440209052466485 -0.182818475502613 0.00278713471210019 0.00278713471210019 0.234907516878402 blue

NBPF14 -0.440508476395347 0.440508476395347 0.0980510038090323 0.00276648686613849 0.00276648686613849 0.526602018624089 brown

HCLS1 -0.433145597740445 0.433145597740445 0.936437418137845 0.00331519221164778 0.00331519221164778 1.02136656250726e-20 yellow

KRT37 -0.438044342451997 0.438044342451997 0.010609121273599 0.00294048673075472 0.00294048673075472 0.945507906940178 blue

LRRTM3 -0.439445828609417 0.439445828609417 0.220448359276021 0.00284038004927551 0.00284038004927551 0.150448038361228 brown

EPHX1 -0.433542560710498 0.433542560710498 0.760507903268798 0.00328334079826448 0.00328334079826448 2.10478326461613e-09 yellow

CASR -0.440498196495588 0.440498196495588 0.14496449309916 0.00276719351576919 0.00276719351576919 0.347794732363039 grey

RUFY4 -0.439438729100371 0.439438729100371 0.00883252358707518 0.00284087950851396 0.00284087950851396 0.954622680284124 blue

PLCL1 -0.438195938411279 0.438195938411279 0.235919285551143 0.00292951082601396 0.00292951082601396 0.123143192396965 grey

RHOH -0.435716055181176 0.435716055181176 -0.0353198484278908 0.003113660544762 0.003113660544762 0.819948189017012 blue

ODF4 -0.441650361566174 0.441650361566174 -0.0572195054033131 0.00268897427886749 0.00268897427886749 0.712182048550199 blue

PCDH7 -0.431936285108505 0.431936285108505 0.302415693736806 0.00341390196345828 0.00341390196345828 0.0460166707751295 grey

CRABP1 -0.434424129868866 0.434424129868866 0.0400085328885859 0.00321356448450643 0.00321356448450643 0.79652288038965 grey

MYRIP -0.431136760533431 0.431136760533431 0.312186327508472 0.0034805697529443 0.0034805697529443 0.039105818533513 brown

LRFN5 -0.436781180667493 0.436781180667493 0.113520035986863 0.00303335721514685 0.00303335721514685 0.463128129123742 blue

FOXD4L5 -0.433395252567247 0.433395252567247 0.0817779287471422 0.00329512899361667 0.00329512899361667 0.597692044908342 blue

DYNC1I1 -0.432243669786541 0.432243669786541 0.511301078312605 0.00338857054548352 0.00338857054548352 0.000389729803088998 yellow

RPS24 -0.435436419001425 0.435436419001425 0.161069950312464 0.00313505024952817 0.00313505024952817 0.296254868140761 brown

NLGN4X -0.435765508220136 0.435765508220136 0.0172068498144637 0.0031098911913821 0.0031098911913821 0.911727666719475 blue

PACRG -0.436654679154844 0.436654679154844 0.240646216248445 0.00304279835602786 0.00304279835602786 0.115594817771663 blue

RPL39 -0.430650137228569 0.430650137228569 0.249787370795545 0.00352170290455922 0.00352170290455922 0.101998636406909 brown

LBP -0.427337711683179 0.427337711683179 0.385547997608755 0.00381319172436192 0.00381319172436192 0.00974948616957345 grey

ATOH1 -0.430227536684774 0.430227536684774 -0.056691789695755 0.00355776943366497 0.00355776943366497 0.714723942063112 blue

MFAP5 -0.432523781356283 0.432523781356283 0.297416379388543 0.00336563041997647 0.00336563041997647 0.0499183693070517 grey

ZC3H12B -0.435467144660092 0.435467144660092 0.365559025748922 0.00313269371358844 0.00313269371358844 0.014681057205582 brown

PGA4 -0.433565115191956 0.433565115191956 -0.0214048239996827 0.00328153916811045 0.00328153916811045 0.890310151673721 blue

CABLES1 -0.437787608046769 0.437787608046769 0.232350431308147 0.00295915738039035 0.00295915738039035 0.129083635810968 brown

INSL4 -0.438431306653531 0.438431306653531 -0.072517892659122 0.0029125409830093 0.0029125409830093 0.639929055935892 blue

WIF1 -0.4241187370853 0.4241187370853 0.281109248244447 0.00411647585758051 0.00411647585758051 0.0645362917876558 grey

MTMR12 -0.433235543495383 0.433235543495383 0.171966443853654 0.00330795150812565 0.00330795150812565 0.2643375179436 blue

OR52N4 -0.427642797579811 0.427642797579811 0.0814752337281133 0.0037854889458778 0.0037854889458778 0.599053233258938 grey

ZW10 -0.428189385594642 0.428189385594642 -0.0115493189447735 0.0037362974128404 0.0037362974128404 0.940686962260119 grey

ST6GALNAC3 -0.43384006720614 0.43384006720614 0.340548318861792 0.00325964592054815 0.00325964592054815 0.0236977338319462 blue

KCNJ14 -0.429507482608704 0.429507482608704 -0.241214120937848 0.00361996798390649 0.00361996798390649 0.114712068977978 blue

LCN2 -0.423859777028781 0.423859777028781 0.237413134897832 0.00414176645008572 0.00414176645008572 0.120718657434084 grey

HOXC8 -0.423898848160685 0.423898848160685 0.146259903584416 0.00413794200106333 0.00413794200106333 0.343457223024806 grey

NCF1 -0.432816667105712 0.432816667105712 0.738575716401939 0.00334178994249286 0.00334178994249286 1.04660496428561e-08 yellow

CIDEC -0.430752237323838 0.430752237323838 0.329181153303134 0.00351303746902557 0.00351303746902557 0.0291174522293961 grey

OR6N2 -0.434365534557677 0.434365534557677 -0.0182522184305288 0.00321816158892477 0.00321816158892477 0.906387954887814 blue

SLC7A2 -0.423412875348504 0.423412875348504 0.240316788781463 0.00418573137966318 0.00418573137966318 0.116109231725261 brown

KIF2B -0.434421895492326 0.434421895492326 0.19420482485019 0.0032137396770882 0.0032137396770882 0.206520619363585 grey

NCKAP1L -0.43380754694754 0.43380754694754 0.567803972652878 0.00326222866644673 0.00326222866644673 5.82326914256733e-05 grey

IFITM1 -0.427817089750557 0.427817089750557 0.647965151034096 0.00376974187607074 0.00376974187607074 1.98446128161566e-06 yellow

DCT -0.430533932119526 0.430533932119526 -0.216551901020172 0.00353158825326269 0.00353158825326269 0.157977260950087 blue

EPYC -0.428858296115568 0.428858296115568 0.349297034747057 0.00367685956368898 0.00367685956368898 0.0201251574275951 brown

ZNF85 -0.432707930607455 0.432707930607455 0.309687331747431 0.00335062358780422 0.00335062358780422 0.0407868536540098 grey

SGMS2 -0.430449114918428 0.430449114918428 0.465538602485732 0.00353881883888543 0.00353881883888543 0.00145043156011573 brown

ATP6V1E1 -0.4259686841494 0.4259686841494 0.338852679946197 0.00393970048146023 0.00393970048146023 0.0244481398539158 brown

EEF1B2 -0.424074544945159 0.424074544945159 0.15473070177546 0.00412078217843005 0.00412078217843005 0.315920608714639 brown

OR6C4 -0.431873096935275 0.431873096935275 0.0447608963285727 0.00341912979990172 0.00341912979990172 0.772958483278321 turquoise

AGTRAP -0.431246413335385 0.431246413335385 0.0321088425595905 0.00347135941854356 0.00347135941854356 0.8360819389804 brown

ACCSL -0.428230309233868 0.428230309233868 -0.15637770383662 0.00373263701728449 0.00373263701728449 0.31073361297525 blue

FNBP1L -0.430425210950883 0.430425210950883 0.384401050695337 0.00354085897171261 0.00354085897171261 0.00998784281544132 grey

NPS -0.42979706796319 0.42979706796319 -0.182076096334222 0.00359483988184516 0.00359483988184516 0.236846423245827 blue

DNASE1L3 -0.419223834937244 0.419223834937244 0.646371602051676 0.00461807300059944 0.00461807300059944 2.14278234594838e-06 yellow

PCSK1 -0.428406044421678 0.428406044421678 0.159267011836407 0.00371695414015134 0.00371695414015134 0.301765804787793 blue

CADM4 -0.421496590389276 0.421496590389276 0.146159506064122 0.00437890735131672 0.00437890735131672 0.34379219521615 turquoise

BCAS1 -0.430466986782372 0.430466986782372 -0.217589251844862 0.00353729420142124 0.00353729420142124 0.155946429884691 blue

TNP2 -0.429165248408021 0.429165248408021 -0.2554956784776 0.00364986286892419 0.00364986286892419 0.0941527380342001 blue

TPPP -0.419598265990435 0.419598265990435 0.488867526306595 0.00457790395151522 0.00457790395151522 0.000759362327246805 yellow

TIMP3 -0.418870918371525 0.418870918371525 0.434191451642459 0.00465621512961187 0.00465621512961187 0.00323185336357713 brown

HAUS4 -0.42327855598745 0.42327855598745 -0.065364139214488 0.00419902480887874 0.00419902480887874 0.67335641765625 grey

KRTAP19-8 -0.428086057882656 0.428086057882656 -0.0856562222132261 0.00374555350265059 0.00374555350265059 0.580373049279192 blue

OR5D13 -0.427704841924446 0.427704841924446 0.0770772915922411 0.00377987673145846 0.00377987673145846 0.618980970091719 blue

PPARGC1A -0.423065039576549 0.423065039576549 0.190738143840481 0.00422023222275311 0.00422023222275311 0.214896207487285 blue

ZFHX4 -0.418558230366297 0.418558230366297 0.661882392223406 0.00469023878588925 0.00469023878588925 9.95626686558258e-07 yellow

PLS1 -0.428293630155496 0.428293630155496 0.330431467760156 0.00372697949609083 0.00372697949609083 0.0284750926409909 grey

IFNA4 -0.422487406134621 0.422487406134621 -0.000210151263690487 0.00427807518861275 0.00427807518861275 0.998919781172289 blue

CLPSL1 -0.425829697552384 0.425829697552384 -0.315264216276829 0.00395274693715453 0.00395274693715453 0.0371137331596974 blue

SRP14 -0.420700612587653 0.420700612587653 0.512921350462868 0.00446140869941476 0.00446140869941476 0.000370732521921586 brown

KRTAP19-2 -0.4243136446074 0.4243136446074 0.0242783112372944 0.00409752992212208 0.00409752992212208 0.875693417738972 brown

ALDH1A3 -0.426061051409483 0.426061051409483 0.380939245651897 0.00393105096329228 0.00393105096329228 0.0107378334757157 brown

ANO5 -0.417112412041764 0.417112412041764 0.202311628416522 0.00485039336568432 0.00485039336568432 0.187832929843608 grey

CXorf65 -0.42605138324796 0.42605138324796 0.0117826931363909 0.00393195553667531 0.00393195553667531 0.939490633731773 grey

CXorf38 -0.421449617544573 0.421449617544573 0.40401449102415 0.0043837387264743 0.0043837387264743 0.00653220062157215 brown

APBA3 -0.420293803792688 0.420293803792688 0.0541364452713882 0.00450409510452755 0.00450409510452755 0.727075226271728 grey

CXCL9 -0.422884138801168 0.422884138801168 0.163660809902585 0.00423827328789631 0.00423827328789631 0.2884499277861 blue

MYOM3 -0.421629004908326 0.421629004908326 0.0498423828878064 0.00436531292418033 0.00436531292418033 0.747983941149563 blue

FABP12 -0.417045543731275 0.417045543731275 -0.0794660392423654 0.00485791436770101 0.00485791436770101 0.608122476465124 blue

IQCJ -0.423773414546501 0.423773414546501 -0.0432792687861626 0.00415023094301371 0.00415023094301371 0.780284533101534 turquoise

GSN -0.417066968526486 0.417066968526486 0.583262114630524 0.00485550351154543 0.00485550351154543 3.25193454965749e-05 yellow

MYH11 -0.422396150148385 0.422396150148385 0.382923909354542 0.00428727643544098 0.00428727643544098 0.010302174119457 yellow

SVEP1 -0.418719742569785 0.418719742569785 0.657787812328267 0.00467263762669662 0.00467263762669662 1.22412661754218e-06 yellow

KCNU1 -0.420414884739595 0.420414884739595 -0.134697916773971 0.00449135298864824 0.00449135298864824 0.383347316279065 blue

CRYGB -0.414065420529979 0.414065420529979 -0.0544107989285717 0.00520359229382162 0.00520359229382162 0.725745790285692 grey

ERAS -0.41631023504902 0.41631023504902 -0.0311769581003295 0.00494129092360818 0.00494129092360818 0.840776986839338 turquoise

SOHLH2 -0.418392649046142 0.418392649046142 0.163488207321303 0.00470834338941409 0.00470834338941409 0.288965698073571 blue

PDE5A -0.417281651816357 0.417281651816357 0.731365742317644 0.00483140348403832 0.00483140348403832 1.71371319824664e-08 yellow

FAM229B -0.415490044117269 0.415490044117269 -0.0713146948049098 0.00503576082432815 0.00503576082432815 0.645504461709348 blue

ALLC -0.415410663041017 0.415410663041017 0.162010182169864 0.00504498691574474 0.00504498691574474 0.293406835074922 turquoise

KEL -0.417149869282465 0.417149869282465 -0.0249202179923423 0.00484618480545176 0.00484618480545176 0.872433595717023 blue

PGPEP1L -0.422204963462288 0.422204963462288 -0.145990460819939 0.00430660966125904 0.00430660966125904 0.344356661766504 blue

OR4N2 -0.415530757999623 0.415530757999623 0.0194641557486722 0.00503103454446576 0.00503103454446576 0.900202531230944 blue

PAH -0.414302638092609 0.414302638092609 0.340359023613203 0.00517531292213388 0.00517531292213388 0.023780539870717 grey

ACSS3 -0.410511857115241 0.410511857115241 0.624547664731244 0.00564360948927678 0.00564360948927678 5.87343806796808e-06 yellow

C6 -0.41946616452017 0.41946616452017 -0.0124119545584578 0.00459204088201561 0.00459204088201561 0.936265561430974 blue

KLK1 -0.410520296375178 0.410520296375178 0.374644016745756 0.00564252734704994 0.00564252734704994 0.012225677481162 grey

HAS1 -0.412874436415964 0.412874436415964 0.493036664649396 0.00534761515465906 0.00534761515465906 0.00067313270316431 yellow

GLRA2 -0.413155675869466 0.413155675869466 0.307249910676042 0.00531329639337236 0.00531329639337236 0.0424828649253187 grey

UNCX -0.418199517034425 0.418199517034425 0.109079749355371 0.00472953735345059 0.00472953735345059 0.480915567486786 brown

RPS26 -0.415006434514594 0.415006434514594 0.216866688022785 0.00509219723998037 0.00509219723998037 0.157358970269273 brown

TMC5 -0.413145438022241 0.413145438022241 0.0697552387392623 0.0053145423161483 0.0053145423161483 0.652759335163055 grey

PCDHB11 -0.415998603996334 0.415998603996334 0.0957142692440553 0.00497700121264596 0.00497700121264596 0.536548598440212 brown

AKR1D1 -0.414837377807497 0.414837377807497 0.100570196808293 0.00511205538182555 0.00511205538182555 0.515981450049533 grey

SERPINB4 -0.41001297367402 0.41001297367402 0.230500644100666 0.0057079012282229 0.0057079012282229 0.132245817084812 blue

KLHL15 -0.414153748984087 0.414153748984087 -0.0812202518540666 0.00519304673191953 0.00519304673191953 0.60020091012619 blue

ANKRD18A -0.417648321306232 0.417648321306232 0.0683770146451436 0.00479048279444777 0.00479048279444777 0.659197567066732 blue

CLEC6A -0.416024251550862 0.416024251550862 0.0926326028643442 0.00497405375230327 0.00497405375230327 0.549803768261913 blue

HHEX -0.416193514983298 0.416193514983298 0.203203073369246 0.00495463982116506 0.00495463982116506 0.185853930542162 grey

CPLX1 -0.410164240771558 0.410164240771558 0.15807982295265 0.00568834035586884 0.00568834035586884 0.305430288170335 grey

PRAMEF20 -0.415311601046418 0.415311601046418 0.0204669773452022 0.00505652107618205 0.00505652107618205 0.895088778403717 blue

KRTAP20-2 -0.411862819974188 0.411862819974188 0.0250566249738944 0.00547265905590209 0.00547265905590209 0.871741141439284 blue

KRBA2 -0.413307084885225 0.413307084885225 -0.117937399758556 0.00529489991149321 0.00529489991149321 0.445789340612032 blue

MLIP -0.408839142467466 0.408839142467466 0.455034394186686 0.00586169014254165 0.00586169014254165 0.00191313556792354 yellow

TIE1 -0.407909047575979 0.407909047575979 0.515528784513763 0.00598608897052865 0.00598608897052865 0.000341906247035596 yellow

CSF1R -0.40433354831637 0.40433354831637 0.875729032754679 0.00648589257123508 0.00648589257123508 7.25677678525217e-15 yellow

ADCK2 -0.409723110414602 0.409723110414602 0.0768540485165434 0.00574554780841427 0.00574554780841427 0.619999918286921 grey

PENK -0.404380532556629 0.404380532556629 0.309935008317531 0.00647909735847736 0.00647909735847736 0.0406176548607819 grey

SAMD7 -0.41039432669765 0.41039432669765 0.0496677743051872 0.00565869883777354 0.00565869883777354 0.748838055980238 blue

SERPINB10 -0.412536398712574 0.412536398712574 0.0183782523681688 0.00538911990358377 0.00538911990358377 0.905744447990133 blue

ANKRD34A -0.409602898386879 0.409602898386879 -0.0357334443806132 0.00576122376052811 0.00576122376052811 0.81787525121546 blue

GUCY2F -0.403928476741202 0.403928476741202 -0.199615341331868 0.00654473367772703 0.00654473367772703 0.193909770829817 blue

TRAPPC6B -0.401015886500473 0.401015886500473 0.256042889540782 0.00698160490104945 0.00698160490104945 0.0934258223390649 grey

POMP -0.410572058569282 0.410572058569282 0.0439435126142016 0.00563589397293171 0.00563589397293171 0.776997748770069 blue

MS4A6A -0.403134159338929 0.403134159338929 0.824367641324818 0.00666146099324103 0.00666146099324103 6.12524516983714e-12 yellow

UCP2 -0.404913408811977 0.404913408811977 0.300359469596207 0.00640245957951931 0.00640245957951931 0.047590359818795 brown

GMPPB -0.4033901900723 0.4033901900723 0.00463055618599557 0.0066236411218041 0.0066236411218041 0.976201425468356 grey

CAPN6 -0.399611232962138 0.399611232962138 0.355347258470477 0.00720116336221364 0.00720116336221364 0.0179289766240086 grey

KRT33B -0.409487471861461 0.409487471861461 -0.143425460419191 0.00577631062471492 0.00577631062471492 0.352991397468648 blue

PNLDC1 -0.406379561708559 0.406379561708559 -0.0725383011773038 0.0061956407246141 0.0061956407246141 0.639834654165478 blue

CXCL3 -0.406539136988502 0.406539136988502 0.0388766389007866 0.00617348435102943 0.00617348435102943 0.80216265087869 blue

PGA3 -0.408965421652163 0.408965421652163 0.232903690984901 0.00584497481733402 0.00584497481733402 0.128148934791663 brown

SOX15 -0.409373462772726 0.409373462772726 0.179582372973695 0.00579124590171406 0.00579124590171406 0.243439077388097 brown

FUNDC2 -0.407470477211182 0.407470477211182 0.145021040257145 0.00604553790437773 0.00604553790437773 0.347604693997901 brown

PPM1H -0.40600041321676 0.40600041321676 0.00853936474084202 0.00624856078826379 0.00624856078826379 0.956127302497568 blue

PRSS23 -0.408435388624376 0.408435388624376 0.454019291009413 0.00591541311183804 0.00591541311183804 0.00196411569327042 brown

PTN -0.399946334911374 0.399946334911374 0.412928227630482 0.00714824914155256 0.00714824914155256 0.00534103630709115 brown

BHMT2 -0.406603519750015 0.406603519750015 0.129543689042475 0.00616456454914253 0.00616456454914253 0.401975825193173 blue

HLA-DQB2 -0.40536406000316 0.40536406000316 0.221060518607069 0.0063382618814858 0.0063382618814858 0.149289534217222 blue

TRMT6 -0.397036355669996 0.397036355669996 0.114378259597421 0.00761917008464595 0.00761917008464595 0.459731404679157 grey

AMIGO2 -0.399193107268671 0.399193107268671 0.15243414407473 0.00726766214400051 0.00726766214400051 0.323244181093895 grey

GPR85 -0.39896422070204 0.39896422070204 0.416290992164268 0.00730428865953583 0.00730428865953583 0.00494348951056442 grey

HIST2H2BE -0.405705507812839 0.405705507812839 0.258966882261241 0.00628999316335738 0.00628999316335738 0.0896148742208479 brown

NFIA -0.401610428394797 0.401610428394797 0.492022871652172 0.00689043268392613 0.00689043268392613 0.000693251944136237 brown

SERPIND1 -0.40398625860687 0.40398625860687 -0.186909962282656 0.00653631204437634 0.00653631204437634 0.224416050858487 blue

ZNF597 -0.402911444275312 0.402911444275312 0.342796436970201 0.00669451158054702 0.00669451158054702 0.0227326017025215 grey

GRIA4 -0.403484179492013 0.403484179492013 0.0534940662370009 0.00660980411811844 0.00660980411811844 0.730191096874543 turquoise

PRSS35 -0.396515953966239 0.396515953966239 0.243280661224326 0.00770615145663182 0.00770615145663182 0.111542947338186 grey

KRTAP19-1 -0.398377780824833 0.398377780824833 0.099698989489016 0.00739886000450675 0.00739886000450675 0.519642187602751 blue

RBP7 -0.401848037758881 0.401848037758881 0.379124000928691 0.00685428487407362 0.00685428487407362 0.0111500274703372 grey

RPL31 -0.397066000062769 0.397066000062769 0.266713352479093 0.00761424086959003 0.00761424086959003 0.0801007270202939 brown

NCAPH -0.401092770992076 0.401092770992076 -0.00484307134897427 0.00696975630812764 0.00696975630812764 0.975109545588379 blue

TMEM132D -0.399703951541085 0.399703951541085 -0.137754025603293 0.00718648886546569 0.00718648886546569 0.372546817357909 turquoise

RIPPLY2 -0.40337990080347 0.40337990080347 0.196807147836451 0.00662515741772759 0.00662515741772759 0.200385329282926 brown

FOSB -0.395952443330359 0.395952443330359 0.244149806253798 0.00780130375044379 0.00780130375044379 0.110230145860108 yellow

HPR -0.401020807126719 0.401020807126719 0.0694896948034178 0.00698084606649701 0.00698084606649701 0.653997877422818 blue

HBB -0.391633186333952 0.391633186333952 0.444166330306378 0.00856491162901209 0.00856491162901209 0.00252490010883662 brown

RAB9B -0.398263878757743 0.398263878757743 -0.201188683107793 0.00741735046023011 0.00741735046023011 0.190347128810013 blue

SEMA3A -0.395642365313838 0.395642365313838 0.276737115549412 0.00785409364600606 0.00785409364600606 0.068984920429455 brown

S100P -0.395453362776954 0.395453362776954 0.0752079303143674 0.00788642178782684 0.00788642178782684 0.627534914454869 turquoise

C20orf202 -0.397778125210488 0.397778125210488 0.0459477416582741 0.0074966549551351 0.0074966549551351 0.76710401243137 grey

SLC46A2 -0.397641536932987 0.397641536932987 -0.0370442195128613 0.00751908598818106 0.00751908598818106 0.811313830907022 blue

FAM3B -0.396858763682925 0.396858763682925 0.0931304116601297 0.00764875750133218 0.00764875750133218 0.54765205563472 grey

APOC3 -0.396557745851865 0.396557745851865 -0.178727868439179 0.00769913472373437 0.00769913472373437 0.245726440274836 blue

VN1R1 -0.39146540852059 0.39146540852059 -0.0454546166159072 0.00859582841943882 0.00859582841943882 0.769534964852765 grey

RXFP1 -0.395606682323591 0.395606682323591 0.127854011843016 0.00786018830806025 0.00786018830806025 0.40819467755794 brown

NXF2 -0.393034789240613 0.393034789240613 -0.239708625832408 0.00831035629031302 0.00831035629031302 0.117063453951079 blue

C1QA -0.387801160459365 0.387801160459365 0.926929187529047 0.00929543229960673 0.00929543229960673 1.73733697822087e-19 yellow

DEFB125 -0.391715060974618 0.391715060974618 0.0544300252764926 0.00854985921491209 0.00854985921491209 0.725652654940988 blue

RPS27 -0.39146907516764 0.39146907516764 0.292789400540698 0.0085951517322725 0.0085951517322725 0.0537635963032217 brown

C5orf47 -0.392295593081556 0.392295593081556 0.189215615648996 0.00844378221280635 0.00844378221280635 0.218648223760233 blue

NDUFB9 -0.390716648759162 0.390716648759162 0.313316447988005 0.00873497855105977 0.00873497855105977 0.0383644486174637 brown

C14orf178 -0.388132097640421 0.388132097640421 -0.0352768089096372 0.00923029818650263 0.00923029818650263 0.82016397247163 grey

PPP6R1 -0.388664994691128 0.388664994691128 -0.00789735703139121 0.009126239638872 0.009126239638872 0.959422892166705 turquoise

GAGE1 -0.3890007000424 0.3890007000424 -0.195726373834855 0.0090612059527332 0.0090612059527332 0.202917625548103 blue

XRCC6 -0.39133333183046 0.39133333183046 0.241950918499412 0.00862023405528884 0.00862023405528884 0.113574423133908 brown

PET117 -0.394156633888315 0.394156633888315 0.438660595107402 0.00811133464185418 0.00811133464185418 0.00289609266199847 grey

RCL1 -0.393578164692849 0.393578164692849 0.201492930626576 0.00821343890824668 0.00821343890824668 0.189663592735441 brown

FBXO39 -0.393804007128115 0.393804007128115 -0.127078109248914 0.00817344487650926 0.00817344487650926 0.411068785212677 blue

TLR3 -0.389826460399256 0.389826460399256 0.408204297314735 0.00890293148280899 0.00890293148280899 0.00594635379144349 brown

ADAM2 -0.389994987386213 0.389994987386213 0.166955310252085 0.00887092371404198 0.00887092371404198 0.278720005496038 grey

PRKX -0.38682770215353 0.38682770215353 -0.0373845342972786 0.00948932118948101 0.00948932118948101 0.809612365631097 blue

GOLGA6L6 -0.387554065560708 0.387554065560708 -0.225427181758711 0.00934432210843374 0.00934432210843374 0.141215347100545 blue

SOX17 -0.390105522572893 0.390105522572893 0.141221523536521 0.0088499839592853 0.0088499839592853 0.360515084265085 turquoise

IRF5 -0.389379760289595 0.389379760289595 0.526409970803014 0.00898825302580875 0.00898825302580875 0.000242164594683362 yellow

IFITM5 -0.383434718988273 0.383434718988273 0.322583161371472 0.0101925292097705 0.0101925292097705 0.0327094041550456 grey

ASPA -0.389442181236636 0.389442181236636 0.128361580173233 0.00897628824770272 0.00897628824770272 0.406320795682147 grey

OTC -0.389216254788039 0.389216254788039 -0.13273251706427 0.00901965864515469 0.00901965864515469 0.390389755493002 blue

DGAT2L6 -0.385445952656307 0.385445952656307 0.0204180108817161 0.00977049314762178 0.00977049314762178 0.895338380396556 grey

TM4SF19 -0.386570036548855 0.386570036548855 0.316617900617511 0.0095412197330518 0.0095412197330518 0.0362643547139469 yellow

KIFC1 -0.38743877302821 0.38743877302821 -0.0219837221349246 0.00936720922951505 0.00936720922951505 0.887362386561275 grey

ZNF816-ZNF321P -0.382787124679656 0.382787124679656 0.268786046884946 0.0103317059622044 0.0103317059622044 0.07769424225675 grey

OR5V1 -0.389279213221639 0.389279213221639 -0.0976874640469197 0.00900755462943187 0.00900755462943187 0.528143484453301 blue

XKRX -0.387153695302854 0.387153695302854 -0.0783691602521291 0.00942400796726724 0.00942400796726724 0.613098393680211 grey

OR6F1 -0.381889711951341 0.381889711951341 -0.105124153516248 0.0105272637868121 0.0105272637868121 0.497058230203952 blue

TBC1D3L -0.382815676536941 0.382815676536941 -0.0119929184963358 0.0103255356223561 0.0103255356223561 0.938413083904932 blue

LGALS3BP -0.380179422848024 0.380179422848024 0.731752498209814 0.0109087525916215 0.0109087525916215 1.66964286717369e-08 yellow

IFIT1B -0.38439349745083 0.38439349745083 -0.202918367530493 0.00998942894552726 0.00998942894552726 0.18648435330125 blue

HIST1H4C -0.384026646572066 0.384026646572066 0.228953321626488 0.0100667257944809 0.0100667257944809 0.134935062801158 brown

UTP6 -0.385153805424302 0.385153805424302 -0.0548988686036177 0.00983084963351378 0.00983084963351378 0.723382719975911 blue

LPCAT2 -0.382136834690287 0.382136834690287 0.793915941408756 0.0104730986198232 0.0104730986198232 1.27972228137757e-10 yellow

HMGB1 -0.382887784820509 0.382887784820509 0.208818269515336 0.0103099663718369 0.0103099663718369 0.173729051241882 brown

ALDH8A1 -0.384290309499526 0.384290309499526 -0.0438941611932822 0.0100111193729709 0.0100111193729709 0.777241815983969 blue

CPA4 -0.380917262533967 0.380917262533967 -0.271117354900163 0.0107427460726841 0.0107427460726841 0.0750557009178216 blue

PRPS2 -0.37848107588602 0.37848107588602 0.172671494751905 0.0112992302846478 0.0112992302846478 0.262354273344769 grey

LRRC74A -0.379858271927878 0.379858271927878 -0.0578558685676193 0.0109816915582498 0.0109816915582498 0.709120920078495 blue

OR2S2 -0.38067739356751 0.38067739356751 0.0865649784715888 0.0107964751506334 0.0107964751506334 0.57634791202541 grey

CD53 -0.378794531573411 0.378794531573411 0.816829943713429 0.011226275014372 0.011226275014372 1.368818033135e-11 yellow

HIST1H4B -0.384224243863675 0.384224243863675 0.423447301177548 0.010025027785741 0.010025027785741 0.00418233022318531 brown

BTN1A1 -0.382093303340723 0.382093303340723 -0.13497280880744 0.0104826225920599 0.0104826225920599 0.382368334956724 blue

ST3GAL6 -0.378347030968997 0.378347030968997 0.520203638487065 0.0113305521688722 0.0113305521688722 0.000295235098745346 yellow

ZNF214 -0.376462534150536 0.376462534150536 0.173485245348669 0.0117788209878052 0.0117788209878052 0.260077609808307 grey

VAT1L -0.375843178722386 0.375843178722386 0.339816992149537 0.0119294188653863 0.0119294188653863 0.0240189870250229 grey

CCDC152 -0.377629585946067 0.377629585946067 0.34388150766127 0.0114994610266056 0.0114994610266056 0.0222786820696509 grey

HIST2H2AB -0.375920629797367 0.375920629797367 0.358925706451997 0.0119104969100978 0.0119104969100978 0.0167279045282558 brown

PRTG -0.38183823162352 0.38183823162352 0.125517223419185 0.0105385775792239 0.0105385775792239 0.416885582296931 grey

NDNF -0.376503603534202 0.376503603534202 0.616906772104436 0.0117688925583978 0.0117688925583978 8.21186078340445e-06 yellow

CPLX3 -0.379940670468591 0.379940670468591 0.0915811249625963 0.0109629377092741 0.0109629377092741 0.554361794885002 grey

APOC1 -0.378424264977109 0.378424264977109 0.557275915498385 0.0113124960678582 0.0113124960678582 8.51891803033145e-05 yellow

MTCP1 -0.377586130283082 0.377586130283082 -0.296958852130788 0.0115097606298779 0.0115097606298779 0.0502884030008143 blue

PLEKHD1 -0.381835337719252 0.381835337719252 -0.107659213365269 0.0105392138795975 0.0105392138795975 0.48668085510597 blue

KMT2B 0.857782152041986 -0.857782152041986 -0.195245984198416 1.02754800386911e-13 1.02754800386911e-13 0.204050377873914 blue

PRIMPOL 0.811863470052347 -0.811863470052347 0.0257164215089552 2.27954779615939e-11 2.27954779615939e-11 0.868393106825868 turquoise

SOGA1 0.794274143300627 -0.794274143300627 -0.299032428007899 1.23847752086521e-10 1.23847752086521e-10 0.0486289311447281 turquoise

SLC17A9 0.772744347728821 -0.772744347728821 -0.182477641848495 7.97331967865185e-10 7.97331967865185e-10 0.2357963388798 turquoise

TMEM259 0.77108223734913 -0.77108223734913 -0.010408241673754 9.12879525681966e-10 9.12879525681966e-10 0.946538189654748 turquoise

SNAPC4 0.772187339057089 -0.772187339057089 -0.0730125422484568 8.34430957254438e-10 8.34430957254438e-10 0.637642586800232 blue

RFX8 0.762803170848739 -0.762803170848739 -0.387589698264507 1.76217172711146e-09 1.76217172711146e-09 0.00933725826766298 turquoise

GOLGA6C 0.750600237470356 -0.750600237470356 -0.0467549452252054 4.43315451170244e-09 4.43315451170244e-09 0.763129523846255 turquoise

RBM6 0.753667344276424 -0.753667344276424 -0.119180668003462 3.53332429834751e-09 3.53332429834751e-09 0.440974443622477 turquoise

PLEKHG4 0.73966917444115 -0.73966917444115 -0.243978847876467 9.69832610552376e-09 9.69832610552376e-09 0.110487436408293 turquoise

KDM6B 0.736090891493548 -0.736090891493548 -0.00631230734849723 1.24270254228852e-08 1.24270254228852e-08 0.967562106143479 turquoise

MFAP2 0.738424940346052 -0.738424940346052 -0.210913211370359 1.05762625237776e-08 1.05762625237776e-08 0.169354678655809 blue

MAPK8IP3 0.7304188687606 -0.7304188687606 -0.213084119420669 1.82623960000938e-08 1.82623960000938e-08 0.164906256431858 blue

CARS 0.736679688678925 -0.736679688678925 -0.163886070777704 1.1933528450817e-08 1.1933528450817e-08 0.287777704415412 turquoise

TBC1D17 0.740784331599171 -0.740784331599171 -0.0924704883018689 8.96985735174341e-09 8.96985735174341e-09 0.550505352624608 turquoise

WSB1 0.727546230196695 -0.727546230196695 0.0146404373913081 2.21132278912137e-08 2.21132278912137e-08 0.92485265415256 turquoise

CDK20 0.775700723608713 -0.775700723608713 -0.205337584723643 6.24988470978077e-10 6.24988470978077e-10 0.181175810552409 turquoise

FNBP4 0.71949243295559 -0.71949243295559 0.0649277314421358 3.73398304468107e-08 3.73398304468107e-08 0.675416719857346 turquoise

ADAMTS14 0.717511037981527 -0.717511037981527 -0.276946038663199 4.23597546404064e-08 4.23597546404064e-08 0.0687669609680659 turquoise

WDR90 0.724601615661724 -0.724601615661724 0.0624279068582241 2.68383456501564e-08 2.68383456501564e-08 0.687263323365864 turquoise

CHTF18 0.718389768099674 -0.718389768099674 -0.131859424394156 4.00604159243091e-08 4.00604159243091e-08 0.393542374908889 turquoise

ING5 0.718110462651566 -0.718110462651566 -0.355695001102785 4.07783200521578e-08 4.07783200521578e-08 0.0178091542965352 blue

ADAMTS4 0.718200321133642 -0.718200321133642 -0.245001312743235 4.05460555589481e-08 4.05460555589481e-08 0.108955426404415 turquoise

NRBP2 0.702832869342735 -0.702832869342735 -0.0284055054309961 1.04448665644849e-07 1.04448665644849e-07 0.854771882086542 blue

BMP1 0.715570785256485 -0.715570785256485 -0.271909098211687 4.78798678940125e-08 4.78798678940125e-08 0.0741758353484797 turquoise

ARFGAP1 0.715810774977219 -0.715810774977219 -0.254486989527315 4.71624452963625e-08 4.71624452963625e-08 0.0955041241441213 turquoise

HOMER3 0.719542502215677 -0.719542502215677 -0.20282291437747 3.72204867669927e-08 3.72204867669927e-08 0.186696055196159 turquoise

MZF1 0.707761144048989 -0.707761144048989 -0.108103783486021 7.76171004958453e-08 7.76171004958453e-08 0.484872693783741 turquoise

DMWD 0.720455266210014 -0.720455266210014 -0.278601299096988 3.51062828556588e-08 3.51062828556588e-08 0.0670593225189727 blue

FRMD8 0.712033897224041 -0.712033897224041 -0.0292309507370812 5.97061237856186e-08 5.97061237856186e-08 0.850598855564434 turquoise

FLYWCH2 0.702734169848776 -0.702734169848776 -0.101929034386845 1.05065178018928e-07 1.05065178018928e-07 0.51029758479384 turquoise

KIAA0895L 0.695063302077242 -0.695063302077242 -0.00173650009936107 1.64817933060092e-07 1.64817933060092e-07 0.991074224762504 turquoise

ILF3 0.721989898038019 -0.721989898038019 0.10181425864216 3.18024400652539e-08 3.18024400652539e-08 0.510776455507201 turquoise

BUB1 0.706751256830805 -0.706751256830805 -0.133436366313896 8.25265807247384e-08 8.25265807247384e-08 0.387859054288455 grey

FAM160B2 0.701453902864034 -0.701453902864034 -0.258707057284688 1.13375530530678e-07 1.13375530530678e-07 0.0899485507000204 turquoise

ATG16L2 0.700970655878136 -0.700970655878136 -0.0366289923989304 1.16668486996782e-07 1.16668486996782e-07 0.813390997143527 turquoise

HSF4 0.706546530502269 -0.706546530502269 -0.223164756914933 8.35564784014672e-08 8.35564784014672e-08 0.145357320768457 blue

WDHD1 0.710847288249254 -0.710847288249254 -0.285285674101435 6.42487511758179e-08 6.42487511758179e-08 0.0605028395063901 blue

SNRNP70 0.691894343204765 -0.691894343204765 -0.0469430395765791 1.97716758963681e-07 1.97716758963681e-07 0.762204249314783 turquoise

SMG6 0.712610667425042 -0.712610667425042 -0.124857679476254 5.76079764723636e-08 5.76079764723636e-08 0.419357426858391 blue

ZNF598 0.718718953502097 -0.718718953502097 0.0388581737992365 3.92294507720169e-08 3.92294507720169e-08 0.802254738251882 blue

GPSM1 0.711660493986835 -0.711660493986835 -0.209885217796958 6.11022434750914e-08 6.11022434750914e-08 0.171491139657471 blue

CDK3 0.723173630025648 -0.723173630025648 -0.135195602599979 2.94548824388685e-08 2.94548824388685e-08 0.381575977570354 turquoise

ZNF793 0.695549773461498 -0.695549773461498 -0.0626169346218124 1.60244144575762e-07 1.60244144575762e-07 0.686364891187682 turquoise

COL27A1 0.686916840854082 -0.686916840854082 -0.129346685224568 2.6193875806921e-07 2.6193875806921e-07 0.402698062797383 turquoise

RHOT2 0.68337991170189 -0.68337991170189 -0.0111900346249246 3.1882986814204e-07 3.1882986814204e-07 0.942528985655214 turquoise

PIWIL4 0.717710872648089 -0.717710872648089 -0.185608279508587 4.18262896137468e-08 4.18262896137468e-08 0.227718221068236 turquoise

NOP2 0.697891169106829 -0.697891169106829 -0.043306048749266 1.39836576165995e-07 1.39836576165995e-07 0.780151947917049 blue

CRIPAK 0.677905091974133 -0.677905091974133 -0.345384048544864 4.29921967394791e-07 4.29921967394791e-07 0.0216626802282742 turquoise

RBM4 0.680287689841779 -0.680287689841779 0.0684941276696651 3.7776941628975e-07 3.7776941628975e-07 0.65864953021709 turquoise

FHL3 0.689579845797701 -0.689579845797701 -0.310706856406335 2.25498821816816e-07 2.25498821816816e-07 0.0400940452243069 turquoise

GLIS1 0.676567789454683 -0.676567789454683 -0.215073823061937 4.6204820213801e-07 4.6204820213801e-07 0.160904158973198 blue

ADAM12 0.675216440548402 -0.675216440548402 -0.197781771142458 4.96764690152274e-07 4.96764690152274e-07 0.19812088462646 turquoise

GSDMB 0.682331049523351 -0.682331049523351 -0.375130505052562 3.37789372226898e-07 3.37789372226898e-07 0.0121047396827029 turquoise

CMIP 0.673429089509572 -0.673429089509572 -0.151490245546322 5.4640694869658e-07 5.4640694869658e-07 0.326284883416652 turquoise

NARF 0.672805513612246 -0.672805513612246 -0.132126918074034 5.64782964870597e-07 5.64782964870597e-07 0.392574916398311 turquoise

MTHFD2 0.677790481404891 -0.677790481404891 -0.0488492512425986 4.32591766979032e-07 4.32591766979032e-07 0.752845885887222 turquoise

PLXNA3 0.679709986421535 -0.679709986421535 -0.243050133979188 3.89845045556554e-07 3.89845045556554e-07 0.111893134358664 turquoise

KIFC3 0.699565312223097 -0.699565312223097 -0.342894069218885 1.26758050879418e-07 1.26758050879418e-07 0.0226914445290743 blue

CEMIP 0.665588215621945 -0.665588215621945 -0.138470581019027 8.23563314651234e-07 8.23563314651234e-07 0.370041022359444 turquoise

NT5DC2 0.666531887608856 -0.666531887608856 -0.0828080993226155 7.84387736965398e-07 7.84387736965398e-07 0.593069658035605 turquoise

SCHIP1 0.669420384152044 -0.669420384152044 -0.26456735764212 6.74952483427384e-07 6.74952483427384e-07 0.0826534144486454 turquoise

PLXNA1 0.662806224960897 -0.662806224960897 -0.0300295892598008 9.49864590924562e-07 9.49864590924562e-07 0.846565184114917 turquoise

DAZAP1 0.675727641219975 -0.675727641219975 -0.0305092927027356 4.83356563690046e-07 4.83356563690046e-07 0.844144207805132 turquoise

MZT1 0.674862635000367 -0.674862635000367 -0.0760553638365682 5.06245847642822e-07 5.06245847642822e-07 0.623651105196569 turquoise

TAF6 0.677554743423138 -0.677554743423138 -0.0755979186303877 4.38131645157888e-07 4.38131645157888e-07 0.625746344438078 turquoise

NOL12 0.668385479897546 -0.668385479897546 0.0826814588443915 7.12419581083826e-07 7.12419581083826e-07 0.593637043507103 turquoise

MRI1 0.65901399465165 -0.65901399465165 -0.0810261695714838 1.1510609035102e-06 1.1510609035102e-06 0.601075118960706 turquoise

SS18L1 0.658534485750072 -0.658534485750072 -0.0405043923649825 1.17913893447955e-06 1.17913893447955e-06 0.794055417137813 turquoise

PIK3CB 0.678382557050581 -0.678382557050581 -0.026701735545804 4.18963455974786e-07 4.18963455974786e-07 0.863397533654497 blue

GSDMD 0.6879560135368 -0.6879560135368 -0.30011630634076 2.47112477555087e-07 2.47112477555087e-07 0.0477793080227355 blue

BBS2 0.699077308183421 -0.699077308183421 -0.0326590078510684 1.30447642261143e-07 1.30447642261143e-07 0.833312709103619 blue

LENG8 0.65703333013454 -0.65703333013454 0.0824991542853927 1.27119511876263e-06 1.27119511876263e-06 0.594454240107092 turquoise

AP5Z1 0.674137270532515 -0.674137270532515 0.121290310523447 5.26211408605993e-07 5.26211408605993e-07 0.432870384421146 turquoise

HOXA9 0.689687794136124 -0.689687794136124 -0.0980615287333415 2.2412624688991e-07 2.2412624688991e-07 0.526557424293192 blue

ZNF469 0.655804986558262 -0.655804986558262 -0.238239146425917 1.3514251011082e-06 1.3514251011082e-06 0.11939358191292 turquoise

GPR107 0.692627379161971 -0.692627379161971 0.023424723940392 1.89604694432367e-07 1.89604694432367e-07 0.880031367592095 blue

OGT 0.652953434525459 -0.652953434525459 0.0693628274431362 1.5560894443692e-06 1.5560894443692e-06 0.654589933029421 turquoise

RAB15 0.674940800429081 -0.674940800429081 0.015558705821578 5.04136855873971e-07 5.04136855873971e-07 0.920154055714883 turquoise

EGLN3 0.660124368131035 -0.660124368131035 -0.258214583179307 1.08840266356786e-06 1.08840266356786e-06 0.0905836470492054 blue

RAD18 0.665061998690045 -0.665061998690045 -0.122872193781637 8.46188827437309e-07 8.46188827437309e-07 0.426848587502001 blue

OAZ2 0.669433410138522 -0.669433410138522 0.0284079367396178 6.74492768181762e-07 6.74492768181762e-07 0.85475958483327 turquoise

TMEM200A 0.649092163766256 -0.649092163766256 -0.0863058495740404 1.87910042379974e-06 1.87910042379974e-06 0.577494372453955 blue

ZNF107 0.690226801122184 -0.690226801122184 -0.0414283461062802 2.17388222122998e-07 2.17388222122998e-07 0.789463011611768 grey

GPD1 0.667955217843602 -0.667955217843602 -0.251361835963254 7.28556917263282e-07 7.28556917263282e-07 0.0997861729528999 brown

PLEKHG2 0.67141903693532 -0.67141903693532 0.0802923330804019 6.0771497416796e-07 6.0771497416796e-07 0.604385550346646 turquoise

MON1B 0.664234322107882 -0.664234322107882 -0.315736794021477 8.82944918426034e-07 8.82944918426034e-07 0.0368153758189577 brown

RAD54L 0.647700409376691 -0.647700409376691 -0.393180065760127 2.0099909867456e-06 2.0099909867456e-06 0.00828434846733344 grey

AKAP17A 0.659775385100526 -0.659775385100526 0.0100044075958149 1.10774668747898e-06 1.10774668747898e-06 0.948609655912245 turquoise

FOSL2 0.653104864453907 -0.653104864453907 -0.0805522306979746 1.54453730490895e-06 1.54453730490895e-06 0.603212216603575 turquoise

ZNF765 0.659840319954669 -0.659840319954669 0.0431118622063693 1.10412342430853e-06 1.10412342430853e-06 0.781113487430605 turquoise

POLM 0.669987811441665 -0.669987811441665 -0.250435365155378 6.55193859570986e-07 6.55193859570986e-07 0.101083552165026 turquoise

TAF1C 0.644232133143394 -0.644232133143394 0.0808765177849413 2.37373552172949e-06 2.37373552172949e-06 0.601749576728935 turquoise

BTBD19 0.65585782377881 -0.65585782377881 -0.3799475264041 1.34787968668278e-06 1.34787968668278e-06 0.0109613785396485 turquoise

NLK 0.649028088241618 -0.649028088241618 0.0627042760193867 1.88494908280572e-06 1.88494908280572e-06 0.685949909422515 turquoise

SEC24C 0.676673958375295 -0.676673958375295 -0.166673858368292 4.59418450273677e-07 4.59418450273677e-07 0.279542729546184 blue

HOXB2 0.642672805107823 -0.642672805107823 -0.211606183406243 2.5563295703454e-06 2.5563295703454e-06 0.167925383730731 turquoise

SIN3B 0.649966968780477 -0.649966968780477 0.071936713394701 1.80091126384724e-06 1.80091126384724e-06 0.6426197156079 turquoise

FGF3 0.673325565371245 -0.673325565371245 -0.219153596884682 5.49418748441906e-07 5.49418748441906e-07 0.152920096616723 grey

TYMS 0.63855704377049 -0.63855704377049 -0.0123150635152431 3.10245574563739e-06 3.10245574563739e-06 0.936762080228355 turquoise

CCDC78 0.681604075177322 -0.681604075177322 -0.375292507468313 3.51539367231623e-07 3.51539367231623e-07 0.0120646943527801 brown

HJURP 0.641480971521485 -0.641480971521485 -0.244856646858033 2.70454894698086e-06 2.70454894698086e-06 0.109171197663259 turquoise

NPIPB3 0.639312564308888 -0.639312564308888 -0.105303720760279 2.99476215547513e-06 2.99476215547513e-06 0.496319444905523 blue

ZNF17 0.657617239272352 -0.657617239272352 0.041677875380927 1.23462439125156e-06 1.23462439125156e-06 0.78822395606346 blue

SPAG5 0.640652336735287 -0.640652336735287 -0.216709664968898 2.81223640256426e-06 2.81223640256426e-06 0.157667166712082 grey

MAST2 0.639592669273128 -0.639592669273128 -0.0804729501851521 2.95572057029197e-06 2.95572057029197e-06 0.603570031720482 turquoise

WDR41 0.638028132205137 -0.638028132205137 -0.0983255102260407 3.17996418777453e-06 3.17996418777453e-06 0.525439537354098 turquoise

COL6A3 0.633804721109258 -0.633804721109258 -0.113863545983531 3.86612288589369e-06 3.86612288589369e-06 0.461766950856208 turquoise

FKBP10 0.644114421649022 -0.644114421649022 -0.135968825055496 2.38708677702625e-06 2.38708677702625e-06 0.378833584416496 turquoise

UBE2C 0.634263954479639 -0.634263954479639 -0.133274460615784 3.78539010575066e-06 3.78539010575066e-06 0.388440333820174 turquoise

NPEPL1 0.639708127611056 -0.639708127611056 -0.0767143323947074 2.93976502390031e-06 2.93976502390031e-06 0.620637982584815 turquoise

ERN2 0.634166653617102 -0.634166653617102 -0.280144830058422 3.80236444677863e-06 3.80236444677863e-06 0.0654974224725824 turquoise

LRIG1 0.671839257026814 -0.671839257026814 -0.192400347854443 5.943934339813e-07 5.943934339813e-07 0.210851311453543 turquoise

VAV2 0.640488741799066 -0.640488741799066 -0.150261524833037 2.83395952004668e-06 2.83395952004668e-06 0.330269864064702 blue

MICALL2 0.651827095646525 -0.651827095646525 -0.0666311687468148 1.64455689638627e-06 1.64455689638627e-06 0.667388127220658 turquoise

KIAA1522 0.63287430214315 -0.63287430214315 -0.249467668471889 4.03458242766459e-06 4.03458242766459e-06 0.102452447758368 blue

ODF2 0.651121083828657 -0.651121083828657 -0.184238625935676 1.70235924196912e-06 1.70235924196912e-06 0.231228688387345 turquoise

TMEM128 0.630527589860347 -0.630527589860347 0.0320848317351642 4.49001470103566e-06 4.49001470103566e-06 0.836202841140393 turquoise

CDKN1A 0.637705696561532 -0.637705696561532 -0.0452656446059198 3.22808826264574e-06 3.22808826264574e-06 0.770467118137225 turquoise

FAM193B 0.628580866467958 -0.628580866467958 -0.171102741242307 4.90331157935398e-06 4.90331157935398e-06 0.266780577762513 turquoise

CENPT 0.629283010033362 -0.629283010033362 -0.0981620005267557 4.75036133997744e-06 4.75036133997744e-06 0.526131816728189 turquoise

H2AFY2 0.624957262965121 -0.624957262965121 -0.0482041991973938 5.76740443094e-06 5.76740443094e-06 0.756008864960815 turquoise

TSC2 0.639880936239213 -0.639880936239213 -0.0131068585167538 2.91603266442491e-06 2.91603266442491e-06 0.932705232663763 turquoise

CCNB1IP1 0.64645334230521 -0.64645334230521 0.0751000827256641 2.13438527614483e-06 2.13438527614483e-06 0.628029899684803 turquoise

TATDN2 0.620865114566645 -0.620865114566645 0.11631078809679 6.91069466992329e-06 6.91069466992329e-06 0.452132191530315 turquoise

RFC4 0.660135155422605 -0.660135155422605 0.00224643554010762 1.08780974409642e-06 1.08780974409642e-06 0.988453264567847 blue

GALNT2 0.623107978460407 -0.623107978460407 -0.154023943730883 6.2605510472024e-06 6.2605510472024e-06 0.318163141244595 turquoise

SPDYE5 0.637526771784956 -0.637526771784956 0.0181754394953627 3.25508235321772e-06 3.25508235321772e-06 0.906780003552464 turquoise

MAGED1 0.625932174992817 -0.625932174992817 -0.178822378340197 5.52208365821739e-06 5.52208365821739e-06 0.245472741513627 turquoise

ZNF814 0.630738331016105 -0.630738331016105 0.0196539114225222 4.44725667851227e-06 4.44725667851227e-06 0.899234585215862 turquoise

ULK3 0.620796643452812 -0.620796643452812 -0.0208859066805147 6.93148688018669e-06 6.93148688018669e-06 0.89295373667342 turquoise

MXD3 0.63280606111917 -0.63280606111917 -0.19975256308232 4.04720076246677e-06 4.04720076246677e-06 0.193597184252793 blue

TPCN2 0.634149524728823 -0.634149524728823 -0.142493001122557 3.80535987933837e-06 3.80535987933837e-06 0.356162819489701 turquoise

CHST1 0.620380240110737 -0.620380240110737 -0.192487333699312 7.05917914978782e-06 7.05917914978782e-06 0.210641107043075 turquoise

RHOBTB3 0.615574980880833 -0.615574980880833 0.0619018578186751 8.69799713438743e-06 8.69799713438743e-06 0.68976582584789 turquoise

SLC25A29 0.634469375152596 -0.634469375152596 0.0384401750023371 3.74978347656579e-06 3.74978347656579e-06 0.804340047425091 blue

HERC4 0.618240024157441 -0.618240024157441 0.0813554169472519 7.75035134665531e-06 7.75035134665531e-06 0.599592410823507 turquoise

SCAMP4 0.628312236847207 -0.628312236847207 0.130282500764922 4.96301949651393e-06 4.96301949651393e-06 0.399273939387249 turquoise

TMEM255B 0.619903870961096 -0.619903870961096 -0.1032218940004 7.20791503091951e-06 7.20791503091951e-06 0.504919151793221 blue

AXIN2 0.653357889176833 -0.653357889176833 -0.425719506693609 1.52541176542045e-06 1.52541176542045e-06 0.00396311722813882 turquoise

ARMC7 0.619584045376331 -0.619584045376331 -0.293896250902737 7.30938689367425e-06 7.30938689367425e-06 0.0528227473775426 brown

SBF1 0.629022619774492 -0.629022619774492 -0.0352403731874167 4.80656217122418e-06 4.80656217122418e-06 0.820346657310498 turquoise

ARHGEF1 0.614654484643398 -0.614654484643398 0.121662236330683 9.04932859048634e-06 9.04932859048634e-06 0.431450320694089 turquoise

FHOD1 0.627667080077081 -0.627667080077081 -0.304258271761817 5.10916653914594e-06 5.10916653914594e-06 0.0446426194044851 grey

MCTS2P 0.631624032744606 -0.631624032744606 -0.121407863740188 4.27162791234643e-06 4.27162791234643e-06 0.432421268608464 turquoise

CARS2 0.626402538467029 -0.626402538467029 0.00402216908582533 5.40719719617864e-06 5.40719719617864e-06 0.979327483348717 turquoise

ATG4B 0.62352412927712 -0.62352412927712 -0.0527989363692961 6.14630550527534e-06 6.14630550527534e-06 0.73356767694088 turquoise

SMC4 0.616653497678755 -0.616653497678755 0.0313693231767658 8.30234075164972e-06 8.30234075164972e-06 0.839807354637045 turquoise

MUC1 0.61953361912132 -0.61953361912132 -0.392975192105268 7.32550527204098e-06 7.32550527204098e-06 0.00832104588212645 turquoise

ANKRD36B 0.621575681208374 -0.621575681208374 0.0505286479579215 6.69828653128741e-06 6.69828653128741e-06 0.744629905335986 turquoise

WDSUB1 0.62928897849777 -0.62928897849777 0.0527627489076588 4.7490802818328e-06 4.7490802818328e-06 0.733743593578265 blue

TDP1 0.614283200308667 -0.614283200308667 -0.0749968655541308 9.19469800138561e-06 9.19469800138561e-06 0.628503783872899 blue

PPP2R3A 0.609163644855999 -0.609163644855999 -0.155326221670042 1.14308666144569e-05 1.14308666144569e-05 0.314038817420725 blue

MC1R 0.622794216647116 -0.622794216647116 -0.318749423285477 6.34797791877726e-06 6.34797791877726e-06 0.0349593250047136 brown

COL5A3 0.624089097365569 -0.624089097365569 -0.171506650509268 5.99427812648873e-06 5.99427812648873e-06 0.265636227572491 grey

SEC31B 0.615475500968525 -0.615475500968525 -0.0204750999871486 8.73535199667043e-06 8.73535199667043e-06 0.895047374959275 turquoise

LYSMD1 0.614710992474929 -0.614710992474929 0.0904342214159035 9.02739034262136e-06 9.02739034262136e-06 0.559353702615309 blue

PKLR 0.628401438184499 -0.628401438184499 -0.194445417765741 4.94311890252856e-06 4.94311890252856e-06 0.205947941344297 brown

PIK3R2 0.612832147033887 -0.612832147033887 -0.154987390972578 9.78367789276864e-06 9.78367789276864e-06 0.315108619970408 brown

GDF1 0.644918728949769 -0.644918728949769 -0.190606618779427 2.2972250182083e-06 2.2972250182083e-06 0.215218552684141 brown

XAF1 0.613491462177341 -0.613491462177341 -0.123758757740733 9.51189035596265e-06 9.51189035596265e-06 0.42349436904189 blue

PCGF3 0.638132609087944 -0.638132609087944 -0.125473289370885 3.16451351845251e-06 3.16451351845251e-06 0.417049980799439 turquoise

CRTC2 0.628901738563417 -0.628901738563417 -0.289964988510946 4.83286017108608e-06 4.83286017108608e-06 0.0562255994572206 grey

NPC1 0.606997043622115 -0.606997043622115 -0.0264127155738958 1.25197200246686e-05 1.25197200246686e-05 0.864862337463087 turquoise

CACNA1A 0.607654428500371 -0.607654428500371 -0.300344396699662 1.21796746885755e-05 1.21796746885755e-05 0.0476020544762711 turquoise

NECAB3 0.638792508547417 -0.638792508547417 -0.314404785764705 3.06851444314287e-06 3.06851444314287e-06 0.0376614096176726 blue

ANO8 0.6048074796764 -0.6048074796764 0.130413068092155 1.3716223358248e-05 1.3716223358248e-05 0.398797543200358 turquoise

PTK2 0.607852394565542 -0.607852394565542 0.0994946439675405 1.20789481596302e-05 1.20789481596302e-05 0.520502695543824 turquoise

PABPC1L 0.629201536465247 -0.629201536465247 -0.278144665633271 4.76788054540406e-06 4.76788054540406e-06 0.0675270095466812 blue

ITGB3BP 0.628434170246093 -0.628434170246093 -0.0303393551428689 4.93583493448847e-06 4.93583493448847e-06 0.845001690761304 brown

ARMC9 0.611344609165 -0.611344609165 -0.0203159800156482 1.04233190626832e-05 1.04233190626832e-05 0.895858505301855 turquoise

EGFR 0.611620764206205 -0.611620764206205 -0.04857757770211 1.03017410141343e-05 1.03017410141343e-05 0.754177538900595 grey

TIGD7 0.612478970439481 -0.612478970439481 -0.270450298371318 9.9321872606828e-06 9.9321872606828e-06 0.0758033583006023 grey

CKS2 0.617656926420071 -0.617656926420071 -0.081427566487588 7.94917253026376e-06 7.94917253026376e-06 0.599267711287841 turquoise

CCDC159 0.609220782088562 -0.609220782088562 -0.0892046267334789 1.14033673069464e-05 1.14033673069464e-05 0.564728761450626 turquoise

ABCA5 0.612647083520969 -0.612647083520969 0.0721402415176034 9.86123972615466e-06 9.86123972615466e-06 0.641676933984106 turquoise

FOSL1 0.601725942084173 -0.601725942084173 -0.144129982715872 1.55785668966873e-05 1.55785668966873e-05 0.350606675943593 turquoise

PPFIBP1 0.6031952240372 -0.6031952240372 -0.0434016128992081 1.46634286589874e-05 1.46634286589874e-05 0.779678868673688 turquoise

WDR55 0.630822348495869 -0.630822348495869 -0.116548840756008 4.43031503936243e-06 4.43031503936243e-06 0.45120086396115 turquoise

ST6GAL2 0.604948281905405 -0.604948281905405 -0.244782040054429 1.36362295180325e-05 1.36362295180325e-05 0.109282601978931 blue

ZC3H7B 0.599264916244041 -0.599264916244041 -0.0475831530214105 1.72296157139313e-05 1.72296157139313e-05 0.759057861176944 turquoise

EZH2 0.600221798728776 -0.600221798728776 -0.281510039550979 1.65694745708561e-05 1.65694745708561e-05 0.0641401809853094 turquoise

PTK7 0.629991226481789 -0.629991226481789 -0.217173496283854 4.60055591567708e-06 4.60055591567708e-06 0.156758053540324 grey

MAP1S 0.621447149652009 -0.621447149652009 -0.330925707301583 6.73625700328785e-06 6.73625700328785e-06 0.0282244379548677 brown

RAD23B 0.609935240102002 -0.609935240102002 0.0972815286723813 1.10646049096033e-05 1.10646049096033e-05 0.529867328549488 blue

NOL3 0.631125388248586 -0.631125388248586 -0.168859098022362 4.36970220364959e-06 4.36970220364959e-06 0.273196679711991 turquoise

KIF14 0.616775274408661 -0.616775274408661 -0.18597654713755 8.2587232290308e-06 8.2587232290308e-06 0.226780616553031 grey

TAMM41 0.617197201971726 -0.617197201971726 -0.0990553860341002 8.10922485818635e-06 8.10922485818635e-06 0.52235482239668 blue

PHOX2A 0.60108899469653 -0.60108899469653 0.113462796578596 1.59913415342449e-05 1.59913415342449e-05 0.463355152913735 turquoise

MLXIP 0.610726709110198 -0.610726709110198 -0.183329393191833 1.0700144805828e-05 1.0700144805828e-05 0.233579438663759 blue

EDARADD 0.614433633471759 -0.614433633471759 -0.192864183721524 9.13554178084221e-06 9.13554178084221e-06 0.209732123193965 brown

EXOC1 0.607893707754083 -0.607893707754083 -0.142617352881429 1.20580245047164e-05 1.20580245047164e-05 0.35573888466375 brown

S100A3 0.625719210916484 -0.625719210916484 -0.20392075858884 5.5748365645388e-06 5.5748365645388e-06 0.184271505178817 turquoise

H2AFX 0.617009399365681 -0.617009399365681 -0.100823513383817 8.17545720216766e-06 8.17545720216766e-06 0.514919460605868 blue

GNAI2 0.629990961847384 -0.629990961847384 0.0131818817092255 4.60061106933834e-06 4.60061106933834e-06 0.932320928958518 turquoise

UCKL1 0.599284317430666 -0.599284317430666 -0.0654112424281042 1.72159948043631e-05 1.72159948043631e-05 0.673134181955321 blue

PPAN 0.618508076609638 -0.618508076609638 -0.0664001867786058 7.66049730235692e-06 7.66049730235692e-06 0.668474661312205 turquoise

LCAT 0.607915075798913 -0.607915075798913 -0.307614983071754 1.20472154267792e-05 1.20472154267792e-05 0.042225240420302 turquoise

NCAN 0.623297457678971 -0.623297457678971 -0.514993774161454 6.2082930353017e-06 6.2082930353017e-06 0.000347650722764593 brown

EIF1AD 0.608243122328327 -0.608243122328327 -0.223589830809008 1.188238606207e-05 1.188238606207e-05 0.1445723482989 brown

DMPK 0.599732636312353 -0.599732636312353 -0.113755932337642 1.69039844592105e-05 1.69039844592105e-05 0.462193144416864 turquoise

NLRP1 0.620093268566497 -0.620093268566497 -0.0466591724292841 7.148437551765e-06 7.148437551765e-06 0.763600775219568 blue

R3HDM2 0.60426801253828 -0.60426801253828 0.147224395195411 1.40267159917463e-05 1.40267159917463e-05 0.340249479957029 turquoise

PACS1 0.597920243255477 -0.597920243255477 0.0783716669937291 1.81981200423464e-05 1.81981200423464e-05 0.613087002286171 turquoise

PLAGL1 0.608442403348467 -0.608442403348467 -0.156146132325997 1.17832704474043e-05 1.17832704474043e-05 0.311459622740824 turquoise

TMEM25 0.606637958184896 -0.606637958184896 -0.346256492444265 1.27091267614782e-05 1.27091267614782e-05 0.0213116027723702 blue

HMCN1 0.589698285087734 -0.589698285087734 0.0425446277239899 2.52901322807566e-05 2.52901322807566e-05 0.783924064341599 turquoise

CYB5R2 0.621822854409388 -0.621822854409388 -0.379869819404886 6.62582075484865e-06 6.62582075484865e-06 0.0109790617070438 grey

TYK2 0.601751839822044 -0.601751839822044 0.066102876889952 1.55619926385529e-05 1.55619926385529e-05 0.669874186034412 turquoise

PHKG1 0.616992007317188 -0.616992007317188 -0.294629886741778 8.18161597683769e-06 8.18161597683769e-06 0.0522064794226993 brown

PRC1 0.594016888212892 -0.594016888212892 -0.139499390594718 2.12994536292749e-05 2.12994536292749e-05 0.366460971938927 turquoise

FADS3 0.613582053016039 -0.613582053016039 0.0491387599474832 9.47509394273423e-06 9.47509394273423e-06 0.75142759062233 turquoise

IFT20 0.602321746304794 -0.602321746304794 0.0566207424785508 1.52013316088137e-05 1.52013316088137e-05 0.715066395058922 turquoise

EVL 0.603576084920617 -0.603576084920617 -0.304866164450841 1.44344062883321e-05 1.44344062883321e-05 0.0441966868315884 turquoise

EIF4ENIF1 0.60175047525966 -0.60175047525966 -0.217337164185268 1.5562865538834e-05 1.5562865538834e-05 0.156438179148393 brown

PHF11 0.589591529764371 -0.589591529764371 0.154093345279854 2.53969316429843e-05 2.53969316429843e-05 0.317942487281114 turquoise

RCCD1 0.607758077942856 -0.607758077942856 0.117398222685019 1.21268412562366e-05 1.21268412562366e-05 0.447886389308947 turquoise

TNFRSF12A 0.585170294843702 -0.585170294843702 -0.289001812528949 3.02002734509691e-05 3.02002734509691e-05 0.0570855768064208 turquoise

UAP1L1 0.585868310128023 -0.585868310128023 0.138520718023676 2.93904846369034e-05 2.93904846369034e-05 0.369866071832562 turquoise

DDB2 0.595391372365432 -0.595391372365432 0.0781757453510207 2.01560293264932e-05 2.01560293264932e-05 0.61397760105468 turquoise

CDK1 0.6116537643579 -0.6116537643579 -0.0751271887137251 1.02873001602428e-05 1.02873001602428e-05 0.627905476864596 turquoise

ZNF587B 0.603445388398596 -0.603445388398596 -0.108962165897106 1.4512624948263e-05 1.4512624948263e-05 0.48139141842185 blue

PDZD2 0.615611956529769 -0.615611956529769 -0.0441092827888015 8.6841502316968e-06 8.6841502316968e-06 0.776178088589637 blue

SH3PXD2A 0.585269608336807 -0.585269608336807 0.0804851616443547 3.00838243661665e-05 3.00838243661665e-05 0.603514912000937 turquoise

SRP54 0.593974295083456 -0.593974295083456 -0.0443659356927018 2.13358180569434e-05 2.13358180569434e-05 0.774909527583897 turquoise

RFC5 0.605152022275363 -0.605152022275363 0.195034371908152 1.35212371259628e-05 1.35212371259628e-05 0.20455076101378 turquoise

SBSN 0.58919266852473 -0.58919266852473 -0.353224713124789 2.57996228280268e-05 2.57996228280268e-05 0.0186751940441989 grey

CERCAM 0.591440334702456 -0.591440334702456 -0.191450312159499 2.36044897087886e-05 2.36044897087886e-05 0.213156630029993 blue

SQLE 0.587622152643282 -0.587622152643282 -0.0974367884927607 2.74427207489229e-05 2.74427207489229e-05 0.52920767730712 turquoise

ANAPC15 0.615420440100747 -0.615420440100747 -0.0966606717885718 8.7560908456628e-06 8.7560908456628e-06 0.532509176971575 grey

KIAA1109 0.591172154111304 -0.591172154111304 -0.0692617034957384 2.3857095991161e-05 2.3857095991161e-05 0.655062001123724 turquoise

SPON2 0.578880169377116 -0.578880169377116 -0.00932783938015886 3.84741290657086e-05 3.84741290657086e-05 0.952080855062881 turquoise

SMARCD1 0.585203733105468 -0.585203733105468 0.0130856858410363 3.01610197530964e-05 3.01610197530964e-05 0.932813691701012 turquoise

MEGF6 0.583154080289888 -0.583154080289888 -0.255694177740515 3.26553920157092e-05 3.26553920157092e-05 0.0938885480569552 brown

MARK3 0.584715111455477 -0.584715111455477 0.149930771118222 3.07392910537606e-05 3.07392910537606e-05 0.331347726941513 turquoise

MAP1LC3A 0.607275184202444 -0.607275184202444 -0.284375388934962 1.23747956386708e-05 1.23747956386708e-05 0.0613643392326575 grey

OXSM 0.586558326198034 -0.586558326198034 -0.0696992941718403 2.86095566320912e-05 2.86095566320912e-05 0.653020193628876 turquoise

STX1A 0.583264761850294 -0.583264761850294 -0.280365257679233 3.2516018398479e-05 3.2516018398479e-05 0.0652767498404498 turquoise

KIF11 0.588224004530222 -0.588224004530222 -0.241207523113692 2.68020539859362e-05 2.68020539859362e-05 0.114722295176901 brown

RNASEH2C 0.583877294530489 -0.583877294530489 -0.0380843695027237 3.17544893322533e-05 3.17544893322533e-05 0.806116145575099 turquoise

SOCS5 0.576097845390987 -0.576097845390987 -0.156176022203649 4.27559260658735e-05 4.27559260658735e-05 0.311365853161753 blue

BEND3 0.594149779187388 -0.594149779187388 -0.196583981366525 2.11863611233652e-05 2.11863611233652e-05 0.200906386905653 brown

PURA 0.583504115346764 -0.583504115346764 0.144568966786061 3.22164762010424e-05 3.22164762010424e-05 0.3491257596341 turquoise

USP22 0.581227822183381 -0.581227822183381 0.163389730867473 3.51701963218536e-05 3.51701963218536e-05 0.289260233131725 turquoise

BCL3 0.597051274911083 -0.597051274911083 -0.126504455545579 1.88502975461063e-05 1.88502975461063e-05 0.413201144574048 grey

INF2 0.591683624473842 -0.591683624473842 -0.0307072404636242 2.33774512831677e-05 2.33774512831677e-05 0.843145613461074 turquoise

TMUB1 0.594869648487221 -0.594869648487221 -0.0772762373422871 2.05832498159953e-05 2.05832498159953e-05 0.618073516417622 turquoise

TRIO 0.580629470659931 -0.580629470659931 -0.0160475158532942 3.5986681529768e-05 3.5986681529768e-05 0.917653996281887 turquoise

RPH3AL 0.584021273132779 -0.584021273132779 -0.229671832289756 3.15778736925076e-05 3.15778736925076e-05 0.133681270924669 grey

CALML3 0.598514396411959 -0.598514396411959 0.0872633046430836 1.7764182537131e-05 1.7764182537131e-05 0.573263481101353 blue

PRR14 0.581411699318781 -0.581411699318781 0.0902190052629901 3.49227098248109e-05 3.49227098248109e-05 0.560292769971886 turquoise

TUBE1 0.581303935806112 -0.581303935806112 0.0136711524373797 3.50675585077446e-05 3.50675585077446e-05 0.929815033391041 grey

CANT1 0.596470251369873 -0.596470251369873 0.147719273924144 1.92982520612291e-05 1.92982520612291e-05 0.338610800429455 turquoise

NKPD1 0.592512970636771 -0.592512970636771 -0.419618213598029 2.26184568663915e-05 2.26184568663915e-05 0.00457577254801199 yellow

GTPBP2 0.607007639916585 -0.607007639916585 -0.0916094016035964 1.2514170403611e-05 1.2514170403611e-05 0.554238986411247 turquoise

GALT 0.596318305341444 -0.596318305341444 -0.121817235518839 1.94169981388192e-05 1.94169981388192e-05 0.430859281817636 brown

YTHDF1 0.585922262558692 -0.585922262558692 0.0512109623476012 2.93287278650919e-05 2.93287278650919e-05 0.741299798555168 blue

PCDHB10 0.572677527079003 -0.572677527079003 0.112189935042086 4.86144407720851e-05 4.86144407720851e-05 0.468419005884765 turquoise

ELP5 0.580414783953985 -0.580414783953985 0.161293233233032 3.62838335472864e-05 3.62838335472864e-05 0.295576918758306 turquoise

ATG2B 0.578971222303016 -0.578971222303016 0.147340606843658 3.83408743523554e-05 3.83408743523554e-05 0.339864231930222 blue

FCF1 0.570156327790046 -0.570156327790046 0.131862710807092 5.33923168319948e-05 5.33923168319948e-05 0.393530480329437 turquoise

ANKRD36 0.588341733606014 -0.588341733606014 -0.0572490665433175 2.66783454461108e-05 2.66783454461108e-05 0.712039749691256 turquoise

NUDT17 0.573746785313887 -0.573746785313887 0.0259298813278111 4.67086691994796e-05 4.67086691994796e-05 0.867310422173683 turquoise

DTYMK 0.569620917764807 -0.569620917764807 0.107535369574403 5.44605526250916e-05 5.44605526250916e-05 0.487185178821854 turquoise

ARFGAP3 0.574420812488163 -0.574420812488163 -0.015446261013794 4.55426488591077e-05 4.55426488591077e-05 0.920729272508981 turquoise

LIG1 0.593948525653048 -0.593948525653048 -0.0448937234041547 2.13578466606265e-05 2.13578466606265e-05 0.772302648089897 blue

PSMA1 0.574033385648863 -0.574033385648863 0.180007973339946 4.62095791613583e-05 4.62095791613583e-05 0.242305214320477 turquoise

POSTN 0.566583486607536 -0.566583486607536 -0.0733581653549836 6.08987171202449e-05 6.08987171202449e-05 0.636046946230283 turquoise

SKOR1 0.576854946282279 -0.576854946282279 -0.223057609571353 4.15496503059093e-05 4.15496503059093e-05 0.145555682285098 blue

MINK1 0.577810334383963 -0.577810334383963 0.153878208115333 4.00718438645764e-05 4.00718438645764e-05 0.318626804885981 turquoise

HGSNAT 0.577407156931406 -0.577407156931406 -0.0104282742527425 4.06895241457511e-05 4.06895241457511e-05 0.946435441528748 blue

RUVBL2 0.586433907142219 -0.586433907142219 0.0289977983995543 2.87489490677577e-05 2.87489490677577e-05 0.851777150230157 turquoise

C2CD2 0.586952060120384 -0.586952060120384 -0.434736938301644 2.81725051473787e-05 2.81725051473787e-05 0.00318912045528255 grey

CCDC18 0.570874154479712 -0.570874154479712 0.051226660734423 5.19901254519574e-05 5.19901254519574e-05 0.741223235601987 blue

FBRS 0.599140539653685 -0.599140539653685 -0.140713067039013 1.73171714177012e-05 1.73171714177012e-05 0.362264493883122 blue

PRRC2C 0.570969744876318 -0.570969744876318 0.0106785838251449 5.18059581913227e-05 5.18059581913227e-05 0.945151663809204 turquoise

PALLD 0.583121039282851 -0.583121039282851 0.122219314946659 3.26971040665965e-05 3.26971040665965e-05 0.429328195047742 turquoise

UHRF1 0.581080817284012 -0.581080817284012 -0.221375261118073 3.53692077884914e-05 3.53692077884914e-05 0.148696448005919 turquoise

SLC38A10 0.587189850563753 -0.587189850563753 0.0406425860145239 2.79115229095405e-05 2.79115229095405e-05 0.793368099681329 turquoise

STK19 0.590368030524385 -0.590368030524385 -0.159901407068951 2.46294426171778e-05 2.46294426171778e-05 0.299819236233701 turquoise

NRM 0.596922106047086 -0.596922106047086 -0.0739482522103029 1.89490508862046e-05 1.89490508862046e-05 0.633326449789788 turquoise

TXN2 0.573830249501687 -0.573830249501687 0.0283823946624435 4.65628178227648e-05 4.65628178227648e-05 0.854888775144864 brown

NCAPG2 0.570418569833455 -0.570418569833455 -0.142148390674634 5.28761056256322e-05 5.28761056256322e-05 0.357339254124342 blue

KCTD10 0.572541525352275 -0.572541525352275 -0.0415164888918383 4.88618634113657e-05 4.88618634113657e-05 0.789025273486857 turquoise

PIGBOS1 0.587090102666574 -0.587090102666574 0.0193385478612913 2.80207285557761e-05 2.80207285557761e-05 0.900843337382204 turquoise

ITPR3 0.573513910830426 -0.573513910830426 -0.1378252777258 4.71178195720999e-05 4.71178195720999e-05 0.37229719592079 turquoise

COL5A2 0.562905660513534 -0.562905660513534 -0.0430287608848884 6.96196996969168e-05 6.96196996969168e-05 0.781525072916772 turquoise

RRAD 0.571457446151404 -0.571457446151404 -0.284893118860905 5.08755727520462e-05 5.08755727520462e-05 0.0608731640573094 turquoise

KLHL41 0.594076832653988 -0.594076832653988 -0.466569344987571 2.12483715420105e-05 2.12483715420105e-05 0.00141088708745557 grey

SCFD1 0.573204558892831 -0.573204558892831 0.0547479555387417 4.76663988324543e-05 4.76663988324543e-05 0.724113120390733 blue

ECHDC3 0.587553739148704 -0.587553739148704 -0.367520061158007 2.75164270476744e-05 2.75164270476744e-05 0.0141183074630308 brown

C4orf47 0.564646435520947 -0.564646435520947 0.000425527297522918 6.53592296482225e-05 6.53592296482225e-05 0.997812707994859 turquoise

IGDCC4 0.565233452427867 -0.565233452427867 -0.0385112009501129 6.39770185448695e-05 6.39770185448695e-05 0.803985618655637 turquoise

CTPS1 0.573180805035009 -0.573180805035009 -0.0587550460092677 4.77087621070775e-05 4.77087621070775e-05 0.704803278958745 turquoise

GRIN3B 0.565832856925415 -0.565832856925415 0.10531129473904 6.25931829045774e-05 6.25931829045774e-05 0.49628829604194 turquoise

CHKB 0.587616032639723 -0.587616032639723 0.109542895333723 2.74493068561778e-05 2.74493068561778e-05 0.479043656368586 turquoise

KIAA0232 0.582488388340613 -0.582488388340613 0.231567599194788 3.35052442754525e-05 3.35052442754525e-05 0.13041489358536 turquoise

SMPD4 0.571048298413891 -0.571048298413891 -0.0034509571047639 5.16550605365834e-05 5.16550605365834e-05 0.982262804771705 turquoise

C9orf50 0.575249620490883 -0.575249620490883 -0.169678340768774 4.41453315625859e-05 4.41453315625859e-05 0.270842224487208 brown

DNHD1 0.577761903189981 -0.577761903189981 -0.166239780137002 4.01455863643548e-05 4.01455863643548e-05 0.280814718290945 grey

PTPN1 0.563498775095711 -0.563498775095711 -0.0324398851142072 6.81404672680706e-05 6.81404672680706e-05 0.834415415738667 turquoise

DZIP1L 0.564095045575177 -0.564095045575177 -0.126170148159903 6.66822869261414e-05 6.66822869261414e-05 0.414446722832864 turquoise

ARID2 0.559230235854986 -0.559230235854986 -0.166169909122483 7.9457641466526e-05 7.9457641466526e-05 0.281019816130964 blue

ARHGAP1 0.558841327683484 -0.558841327683484 0.0805046004936483 8.05694197564467e-05 8.05694197564467e-05 0.603427174003459 turquoise

APLN 0.564799647398612 -0.564799647398612 -0.246607299884939 6.4995870715023e-05 6.4995870715023e-05 0.106581854906286 turquoise

IFRD1 0.577140391327044 -0.577140391327044 -0.0114481419348137 4.11029870740916e-05 4.11029870740916e-05 0.941205658579999 turquoise

SPECC1 0.565378036418571 -0.565378036418571 -0.177040080648115 6.36406913002067e-05 6.36406913002067e-05 0.250286922442321 turquoise

RUNX1 0.56035068455073 -0.56035068455073 -0.0718400466872025 7.63322266802492e-05 7.63322266802492e-05 0.643067689027011 turquoise

NXPH4 0.557390370287105 -0.557390370287105 -0.381156662402514 8.48434123630368e-05 8.48434123630368e-05 0.0106893506060979 turquoise

YTHDC2 0.56324792172008 -0.56324792172008 0.311056259835252 6.87625667444208e-05 6.87625667444208e-05 0.0398588362701745 turquoise

SH3BGRL2 0.558708254923925 -0.558708254923925 -0.251092418581949 8.09530767760332e-05 8.09530767760332e-05 0.100162123423769 brown

CDK11A 0.56739020967388 -0.56739020967388 0.0834628613643758 5.91243441261613e-05 5.91243441261613e-05 0.590139965033276 blue

CHD3 0.562185089012868 -0.562185089012868 0.0408311747679805 7.14561495624865e-05 7.14561495624865e-05 0.792430387667409 blue

CELSR1 0.572808632039094 -0.572808632039094 -0.322052012432205 4.83770099550216e-05 4.83770099550216e-05 0.0330137986157217 grey

EGFL8 0.564831479631588 -0.564831479631588 -0.165847324991618 6.49206084591798e-05 6.49206084591798e-05 0.281967992957039 blue

ENOSF1 0.559873883247601 -0.559873883247601 -0.0184842561410001 7.76482847126023e-05 7.76482847126023e-05 0.905203257714312 turquoise

KIF5C 0.56175314672928 -0.56175314672928 -0.295657788981922 7.25780448308416e-05 7.25780448308416e-05 0.0513527820086205 turquoise

PCNT 0.56081752426502 -0.56081752426502 0.0157295962393222 7.50633766935465e-05 7.50633766935465e-05 0.919279934793386 turquoise

HNRNPH3 0.556791641218986 -0.556791641218986 0.0845928036194306 8.66664103167087e-05 8.66664103167087e-05 0.585099279053557 turquoise

SPSB3 0.559698804423468 -0.559698804423468 0.0936884830915092 7.81366969167292e-05 7.81366969167292e-05 0.545244647491053 turquoise

MICAL1 0.557853187746768 -0.557853187746768 -0.0227356929336634 8.34582260012812e-05 8.34582260012812e-05 0.883535577569496 brown

NPIPA1 0.55686042835149 -0.55686042835149 -0.177809474780968 8.64551715369302e-05 8.64551715369302e-05 0.248200968447586 turquoise

RAB34 0.579903958979505 -0.579903958979505 -0.028604109127086 3.69999180660864e-05 3.69999180660864e-05 0.853767481863179 turquoise

FAM184B 0.557294862140063 -0.557294862140063 -0.0401250965333984 8.51318540709528e-05 8.51318540709528e-05 0.795942666822153 turquoise

CDH23 0.569390716696781 -0.569390716696781 -0.207733461163305 5.49258091973217e-05 5.49258091973217e-05 0.176025904367821 turquoise

PXMP2 0.562372186187507 -0.562372186187507 0.0426911247890921 7.09751192553848e-05 7.09751192553848e-05 0.783197927631775 turquoise

DNAJC4 0.563026299582979 -0.563026299582979 -0.0348777070901931 6.93164757459191e-05 6.93164757459191e-05 0.822165537210173 blue

SYN1 0.553088902556789 -0.553088902556789 -0.260158888724219 9.87548774077649e-05 9.87548774077649e-05 0.0880963521934988 blue

TBL3 0.571299288757324 -0.571299288757324 -0.00709158630155552 5.11756043240573e-05 5.11756043240573e-05 0.963560058747679 blue

ELN 0.571698498272653 -0.571698498272653 -0.120482563066679 5.04213734945924e-05 5.04213734945924e-05 0.435963435884647 blue

NEUROG3 0.561804195125602 -0.561804195125602 -0.409289888917769 7.24446245054873e-05 7.24446245054873e-05 0.00580221543709726 brown

PRRC2A 0.566961869981519 -0.566961869981519 0.0105543372697425 6.00605105668487e-05 6.00605105668487e-05 0.945788877613423 turquoise

HSBP1L1 0.559015174008318 -0.559015174008318 -0.221658040171993 8.00707037789873e-05 8.00707037789873e-05 0.148165072481056 blue

ETFDH 0.565382463945048 -0.565382463945048 -0.160217396118948 6.36304175929857e-05 6.36304175929857e-05 0.298852678217968 brown

C12orf75 0.548050739982633 -0.548050739982633 -0.0518216162408123 0.000117666661878017 0.000117666661878017 0.738323391109541 turquoise

ZNF146 0.554614927658514 -0.554614927658514 0.0368507801031621 9.35983990078638e-05 9.35983990078638e-05 0.812281349755489 blue

OSTC 0.554492998580225 -0.554492998580225 0.0319023968248808 9.40012138231243e-05 9.40012138231243e-05 0.837121580736019 turquoise

ABCB9 0.575570918352645 -0.575570918352645 -0.404671047615915 4.36142602431084e-05 4.36142602431084e-05 0.00643721776232666 yellow

SPDYE1 0.552755351787871 -0.552755351787871 -0.158203206948761 9.99157931939759e-05 9.99157931939759e-05 0.305048121190961 grey

IFT122 0.558225140314197 -0.558225140314197 0.21783515136921 8.235994641613e-05 8.235994641613e-05 0.155467841804157 turquoise

HMGXB4 0.565111457993068 -0.565111457993068 -0.324030738019516 6.42620580288823e-05 6.42620580288823e-05 0.0318915545579067 brown

TAF13 0.547762882824156 -0.547762882824156 -0.146877899417069 0.000118840514875088 0.000118840514875088 0.341399732647914 turquoise

MEN1 0.571489078636215 -0.571489078636215 -0.139720001927441 5.08157575455039e-05 5.08157575455039e-05 0.365696008749633 blue

PTTG1 0.554192703466409 -0.554192703466409 -0.113159625377591 9.5000025424133e-05 9.5000025424133e-05 0.464558591054555 turquoise

NKTR 0.558398993225983 -0.558398993225983 0.0162379423819389 8.18511292976954e-05 8.18511292976954e-05 0.916680255054321 turquoise

TM2D3 0.548183538725979 -0.548183538725979 0.164153829279512 0.000117128679719579 0.000117128679719579 0.286979985518756 turquoise

EPS15L1 0.564444354983961 -0.564444354983961 -0.0830340787391783 6.58413175155161e-05 6.58413175155161e-05 0.592057801179969 blue

TCF3 0.551680611037726 -0.551680611037726 -0.038213723114728 0.000103741305738225 0.000103741305738225 0.805470330469868 turquoise

RILPL1 0.555362032341938 -0.555362032341938 0.0460544792555156 9.1164294573279e-05 9.1164294573279e-05 0.766578119207909 turquoise

NID2 0.564845174260856 -0.564845174260856 0.149081246245574 6.48882541303352e-05 6.48882541303352e-05 0.334126197519077 turquoise

NEK8 0.554471736512661 -0.554471736512661 -0.418318493390115 9.40716181434007e-05 9.40716181434007e-05 0.00471647128302007 yellow

DLG1 0.547899767108487 -0.547899767108487 -0.106051361018637 0.000118280994596943 0.000118280994596943 0.493249540744072 turquoise

PDLIM7 0.547992109375135 -0.547992109375135 -0.132835842565056 0.000117904894171414 0.000117904894171414 0.390017642596926 turquoise

TOLLIP 0.555470002217523 -0.555470002217523 0.0674915355504838 9.08173259207579e-05 9.08173259207579e-05 0.66334690656654 grey

SYNPO 0.561537743050209 -0.561537743050209 -0.233425548645816 7.3143493313633e-05 7.3143493313633e-05 0.127271941767968 turquoise

TRPC5OS 0.547317565079657 -0.547317565079657 -0.292399183969444 0.000120677439947428 0.000120677439947428 0.0540984859729733 turquoise

VIPAS39 0.561943935108819 -0.561943935108819 -0.0686335995724668 7.20805415034324e-05 7.20805415034324e-05 0.657997094236121 turquoise

ANKRD10 0.550321361277116 -0.550321361277116 -0.00247454970818929 0.000108770034872544 0.000108770034872544 0.987280843295605 turquoise

POMZP3 0.56651375313612 -0.56651375313612 -0.161997631365468 6.10543518824241e-05 6.10543518824241e-05 0.293444735411642 brown

VEGFA 0.541777231472971 -0.541777231472971 -0.360687095591653 0.000145789276787979 0.000145789276787979 0.0161621665282924 turquoise

FBXL17 0.553833267059168 -0.553833267059168 0.0926545666709616 9.62082320547042e-05 9.62082320547042e-05 0.549708748013205 blue

ARHGEF16 0.55687080173595 -0.55687080173595 -0.128682267244856 8.6423356539974e-05 8.6423356539974e-05 0.405139412945046 blue

ESRRA 0.563234770223498 -0.563234770223498 0.18104812186825 6.87953238926129e-05 6.87953238926129e-05 0.239549186982874 turquoise

RERE 0.565607224552569 -0.565607224552569 0.036279558320622 6.31108614512347e-05 6.31108614512347e-05 0.815140016271001 turquoise

GOLGA3 0.555773435971929 -0.555773435971929 -0.270729942811507 8.98486427701074e-05 8.98486427701074e-05 0.0754892145062456 turquoise

ZC3H7A 0.569197428909273 -0.569197428909273 0.0322458511284402 5.53192583593561e-05 5.53192583593561e-05 0.835392128621848 turquoise

UCN2 0.552155554522976 -0.552155554522976 -0.202670899018034 0.000102034635162245 0.000102034635162245 0.187033557372773 blue

SRPK1 0.559193286156805 -0.559193286156805 -0.00382325154815441 7.95626664901219e-05 7.95626664901219e-05 0.980349644224864 turquoise

AMPD3 0.545776709031324 -0.545776709031324 0.0963067349320816 0.000127234140307291 0.000127234140307291 0.534018106741934 turquoise

NUP107 0.543541520619681 -0.543541520619681 0.166251534170803 0.00013732102926557 0.00013732102926557 0.280780225385955 turquoise

FIBIN 0.541072757140396 -0.541072757140396 -0.16005045072388 0.000149300901350815 0.000149300901350815 0.299363086400986 turquoise

H2AFZ 0.543862601001484 -0.543862601001484 0.112745154649007 0.000135828801748307 0.000135828801748307 0.466206540921184 turquoise

UPF1 0.541827189421501 -0.541827189421501 0.13953620087592 0.000145543116737324 0.000145543116737324 0.366333266586859 turquoise

LYPLAL1 0.552328270696855 -0.552328270696855 0.197010174690198 0.00010142034967926 0.00010142034967926 0.199912120930255 blue

CAMK2N1 0.538914509247276 -0.538914509247276 -0.00766319508289965 0.000160541934566047 0.000160541934566047 0.960625074529072 turquoise

KRBOX1 0.547975543085103 -0.547975543085103 0.032777855511178 0.00011797228689361 0.00011797228689361 0.832714755755594 blue

SCAI 0.547028116135027 -0.547028116135027 -0.00991482065630892 0.000121885195805026 0.000121885195805026 0.949069237825731 blue

NUMBL 0.562116626375646 -0.562116626375646 -0.0663311547212306 7.16329091866337e-05 7.16329091866337e-05 0.668799516667654 blue

HSPB6 0.544335177524087 -0.544335177524087 0.0824413933772851 0.0001336592467495 0.0001336592467495 0.594713261909502 turquoise

CTHRC1 0.540202884064297 -0.540202884064297 -0.116425360196612 0.000153742741629989 0.000153742741629989 0.45168382326451 turquoise

PLEKHO1 0.552029674191828 -0.552029674191828 -0.0291474993680195 0.00010248447210589 0.00010248447210589 0.851020561485471 brown

TNR 0.555448504820146 -0.555448504820146 -0.289242241075753 9.08863133598567e-05 9.08863133598567e-05 0.0568699276595758 brown

MDM4 0.552791631641902 -0.552791631641902 0.0785698396750846 9.97889233714124e-05 9.97889233714124e-05 0.612186730725501 turquoise

ERAL1 0.548356129376983 -0.548356129376983 -0.221541121493147 0.000116432834718502 0.000116432834718502 0.148384606838184 turquoise

ANAPC7 0.561248817268138 -0.561248817268138 -0.0430703539770591 7.39082430903972e-05 7.39082430903972e-05 0.78131906263924 blue

CD70 0.552541442629487 -0.552541442629487 -0.0932985008852668 0.000100666814629799 0.000100666814629799 0.546926419664054 brown

METTL3 0.536158614747385 -0.536158614747385 0.185673872956866 0.000176009116875588 0.000176009116875588 0.22755102646247 turquoise

ARHGAP11B 0.541954259128089 -0.541954259128089 0.00905756814611122 0.000144918698238873 0.000144918698238873 0.953467756497446 turquoise

MSH2 0.53786408898654 -0.53786408898654 0.181367981903569 0.000166285765374485 0.000166285765374485 0.238705970462276 turquoise

BCKDK 0.544473131374533 -0.544473131374533 -0.118244370881865 0.000133031870153595 0.000133031870153595 0.444597834921 blue

CDK10 0.544952808343007 -0.544952808343007 -0.00449399839547358 0.000130871173874191 0.000130871173874191 0.976903067405956 turquoise

SH2B1 0.538719701235017 -0.538719701235017 0.0477971016988939 0.000161593390717471 0.000161593390717471 0.758007080194986 turquoise

CDCA5 0.560835499095573 -0.560835499095573 -0.293932784335786 7.5014908186618e-05 7.5014908186618e-05 0.0527919206297959 grey

VLDLR 0.556664178271235 -0.556664178271235 -0.407475048643061 8.70590788354205e-05 8.70590788354205e-05 0.00604491560627815 turquoise

ZRANB2 0.535957352137349 -0.535957352137349 0.202540612381438 0.000177189899267429 0.000177189899267429 0.18732316316076 turquoise

VPS37B 0.556138984217393 -0.556138984217393 -0.0501697956742493 8.86941526305895e-05 8.86941526305895e-05 0.746383173810807 turquoise

TNFRSF10D 0.535520751485966 -0.535520751485966 -0.333346864075258 0.000179776078682434 0.000179776078682434 0.0270228637164328 turquoise

OBSL1 0.556513045665956 -0.556513045665956 -0.155017573481657 8.75267625533917e-05 8.75267625533917e-05 0.315013230108581 blue

MTAP 0.55702802734857 -0.55702802734857 0.0200243372448515 8.59424508412186e-05 8.59424508412186e-05 0.897345456016842 blue

MGAT4B 0.537950255915094 -0.537950255915094 0.103064894288572 0.000165807677656998 0.000165807677656998 0.505570747642416 turquoise

L3HYPDH 0.542947873481458 -0.542947873481458 0.16446472938773 0.000140119246772139 0.000140119246772139 0.286055543848195 turquoise

IKBIP 0.540064189640289 -0.540064189640289 0.0017490436576058 0.000154461928063527 0.000154461928063527 0.991009752303614 turquoise

ARPC4-TTLL3 0.556644271450509 -0.556644271450509 0.0319045523204485 8.712055069551e-05 8.712055069551e-05 0.83711072443885 turquoise

SHARPIN 0.547240045110616 -0.547240045110616 0.132961291961547 0.000120999829993627 0.000120999829993627 0.389566132917754 turquoise

CYP27C1 0.542464179788161 -0.542464179788161 -0.286840467572505 0.000142437318860441 0.000142437318860441 0.0590537359812695 turquoise

PPP1R37 0.534585582493139 -0.534585582493139 -0.357616620517753 0.000185430874286007 0.000185430874286007 0.017159140791301 grey

LPIN3 0.535205099664581 -0.535205099664581 -0.190100978846053 0.000181667062444567 0.000181667062444567 0.216460914824441 turquoise

LOXL2 0.531909659697281 -0.531909659697281 -0.200844358702395 0.000202512623333969 0.000202512623333969 0.191122810144301 turquoise

TFPI2 0.54513166856962 -0.54513166856962 -0.200827278849775 0.000130073688291681 0.000130073688291681 0.191161345277597 grey

MRPS27 0.532478911825875 -0.532478911825875 -0.20541785028458 0.000198763960944775 0.000198763960944775 0.181001554819604 blue

ENO2 0.535249492177614 -0.535249492177614 -0.243426563747037 0.000181400035198803 0.000181400035198803 0.111321742227025 turquoise

DYNLT1 0.533831979592886 -0.533831979592886 -0.0663026845109407 0.000190104412539043 0.000190104412539043 0.668933510967464 blue

PTPN23 0.542328459711144 -0.542328459711144 -0.000587256913639973 0.000143093968968661 0.000143093968968661 0.996981390014235 turquoise

TNS3 0.533514615062178 -0.533514615062178 0.0859136924130368 0.000192104285959944 0.000192104285959944 0.579231353302209 turquoise

CHPF 0.540264036592952 -0.540264036592952 -0.228120809663917 0.000153426606580578 0.000153426606580578 0.136398713691393 turquoise

MDGA1 0.535548451483781 -0.535548451483781 -0.181060137463441 0.000179610989328176 0.000179610989328176 0.239517474872225 turquoise

PSD3 0.531943553272336 -0.531943553272336 0.0742853935073811 0.000202287647665712 0.000202287647665712 0.631774255830717 turquoise

DDHD2 0.542406587564243 -0.542406587564243 -0.081861786286554 0.000142715630910928 0.000142715630910928 0.597315185922465 turquoise

MYBL1 0.552759724818292 -0.552759724818292 -0.288568689635772 9.99004930374175e-05 9.99004930374175e-05 0.0574757142205596 turquoise

TAF4B 0.530572059305991 -0.530572059305991 -0.029533578631993 0.000211574049883384 0.000211574049883384 0.849069928254324 blue

MED29 0.543210576105665 -0.543210576105665 -0.195194348403915 0.000138874652168534 0.000138874652168534 0.204172397777316 grey

RGS3 0.532954194476514 -0.532954194476514 -0.00568194031499683 0.000195682334976056 0.000195682334976056 0.970799985568146 turquoise

IER3IP1 0.534143559898988 -0.534143559898988 0.0615516608847681 0.000188159341396291 0.000188159341396291 0.691433587577624 turquoise

MBD1 0.534449774721798 -0.534449774721798 -0.108293896446994 0.000186265336172431 0.000186265336172431 0.484100535990681 turquoise

SH3BGRL3 0.544098793154177 -0.544098793154177 -0.00987456791361163 0.000134740503213571 0.000134740503213571 0.949275740132389 turquoise

ATP8B3 0.538544541322709 -0.538544541322709 -0.189917997660972 0.000162544122539453 0.000162544122539453 0.216911724704471 blue

TESK1 0.535409753735124 -0.535409753735124 0.00349452645844598 0.000180438994189056 0.000180438994189056 0.982038903670869 blue

KATNAL1 0.535349941158802 -0.535349941158802 0.036152420862007 0.000180797131861711 0.000180797131861711 0.81577659702912 turquoise

TTC3 0.535229001244066 -0.535229001244066 0.140153958966019 0.000181523246866835 0.000181523246866835 0.364194081441944 turquoise

ARNTL 0.543269813751398 -0.543269813751398 -0.00254589693016004 0.000138595394046016 0.000138595394046016 0.986914150652595 turquoise

CEP76 0.545590155653472 -0.545590155653472 -0.0293441183064275 0.000128049531570784 0.000128049531570784 0.850027050180021 brown

PNMA3 0.530458913472433 -0.530458913472433 -0.25576095952386 0.000212357116303903 0.000212357116303903 0.0937997946335347 brown

ZYX 0.544541848728335 -0.544541848728335 0.220858736941328 0.000132720360480263 0.000132720360480263 0.1496706763565 turquoise

RBM4B 0.54553950906187 -0.54553950906187 -0.235543947668739 0.00012827171585363 0.00012827171585363 0.123758090909454 blue

TXNDC15 0.540863318229009 -0.540863318229009 0.0441545158240984 0.000150359591914126 0.000150359591914126 0.775954472912507 blue

SOX11 0.526099290101972 -0.526099290101972 -0.294862685601815 0.000244600917904144 0.000244600917904144 0.0520121395375518 turquoise

HPS4 0.534352978389761 -0.534352978389761 0.121482260894847 0.000186862168366448 0.000186862168366448 0.432137166321741 turquoise

ETV6 0.539053425107327 -0.539053425107327 0.183126426550898 0.000159795942040618 0.000159795942040618 0.234106414163653 turquoise

SUN1 0.530972372048031 -0.530972372048031 0.0262232425730622 0.00020882446365806 0.00020882446365806 0.865822863027612 turquoise

MCEE 0.542694100195337 -0.542694100195337 0.111030276902146 0.00014133113683981 0.00014133113683981 0.473058079363201 turquoise

LGR4 0.532610057969345 -0.532610057969345 0.0553648001418741 0.000197909281647675 0.000197909281647675 0.721129203690618 turquoise

ASAP3 0.533392598078661 -0.533392598078661 -0.134568901445527 0.000192878230636777 0.000192878230636777 0.383807292101779 brown

IL11 0.522487368779628 -0.522487368779628 -0.332165906759315 0.000274595892923932 0.000274595892923932 0.0276035241950428 turquoise

ZFYVE27 0.527086734087476 -0.527086734087476 0.15851766821295 0.000236933232577658 0.000236933232577658 0.304075498980418 turquoise

GPER1 0.530662167672739 -0.530662167672739 -0.25941222647242 0.000210952291830335 0.000210952291830335 0.0890451821986814 turquoise

ZBTB21 0.528305252373892 -0.528305252373892 -0.259374493132247 0.000227770950470971 0.000227770950470971 0.0890933420493315 turquoise

UBE2O 0.534574140876754 -0.534574140876754 -0.114580902171996 0.000185501045753646 0.000185501045753646 0.458931341171652 turquoise

TUBB3 0.530899760512979 -0.530899760512979 -0.168479859849075 0.000209320790167947 0.000209320790167947 0.274291141165367 turquoise

KCNRG 0.52770430339305 -0.52770430339305 0.0330246757809563 0.000232248792020814 0.000232248792020814 0.831473237428201 turquoise

IFT172 0.533489421680498 -0.533489421680498 -0.0990565966162866 0.000192263855046696 0.000192263855046696 0.522349713506864 turquoise

BRAT1 0.530040225826737 -0.530040225826737 0.00364413965346739 0.000215277645226013 0.000215277645226013 0.981270058155112 blue

ZNF587 0.532407897263836 -0.532407897263836 -0.0452304973505799 0.000199228155442522 0.000199228155442522 0.770640526351115 turquoise

P3H1 0.536716880277465 -0.536716880277465 -0.0938262568310537 0.00017277104948859 0.00017277104948859 0.544651099411201 turquoise

C11orf68 0.540153564990947 -0.540153564990947 -0.0244090544672987 0.000153998132323711 0.000153998132323711 0.875029292249413 turquoise

PXYLP1 0.528461673596969 -0.528461673596969 -0.430552203901017 0.000226618285807361 0.000226618285807361 0.00353003229772006 blue

TJAP1 0.52827753310431 -0.52827753310431 -0.206662105579734 0.000227975765121543 0.000227975765121543 0.178315610856559 brown

SMG9 0.532441195469306 -0.532441195469306 -0.114021528021631 0.000199010376048761 0.000199010376048761 0.461141661423116 turquoise

TRMU 0.526128648603849 -0.526128648603849 -0.126604150517825 0.00024436974802113 0.00024436974802113 0.412830110388045 blue

EML3 0.526261400993328 -0.526261400993328 -0.0225914293545866 0.000243326913366999 0.000243326913366999 0.884269540307008 turquoise

COPA 0.52222087419839 -0.52222087419839 -0.0133675973138728 0.000276935288287862 0.000276935288287862 0.931369672791696 turquoise

GK5 0.527150393216364 -0.527150393216364 -0.0593366258591776 0.00023644644324756 0.00023644644324756 0.702015530965362 grey

FADD 0.52621126828734 -0.52621126828734 -0.0394416233553566 0.00024372025672031 0.00024372025672031 0.799346300698891 turquoise

DVL2 0.521798893938204 -0.521798893938204 -0.100423910408084 0.000280676412469668 0.000280676412469668 0.516595230845131 blue

SYT7 0.523488451532332 -0.523488451532332 0.0987065274461686 0.000265966505377969 0.000265966505377969 0.523828103188192 grey

HOXA3 0.523678586781891 -0.523678586781891 0.183241873276107 0.000264355474575646 0.000264355474575646 0.233806572844672 turquoise

TMEM246 0.523926998743964 -0.523926998743964 -0.208702015523124 0.000262263938414313 0.000262263938414313 0.173974156988058 turquoise

PORCN 0.525361913873401 -0.525361913873401 -0.251556241678658 0.000250472235158054 0.000250472235158054 0.0995155696225279 blue

IFFO2 0.539925456953179 -0.539925456953179 -0.0106134442232367 0.000155184362686089 0.000155184362686089 0.945485736109753 turquoise

CDAN1 0.531467189333532 -0.531467189333532 -0.090659644297972 0.000205470453744956 0.000205470453744956 0.558370889622578 turquoise

FAM156A 0.524245080898728 -0.524245080898728 -0.0854321324410556 0.000259607626259842 0.000259607626259842 0.581367553220886 turquoise

LIF 0.522788005499196 -0.522788005499196 -0.34887384149106 0.000271978209260366 0.000271978209260366 0.0202868864919106 turquoise

AHI1 0.521697787752917 -0.521697787752917 0.231176116310932 0.000281579529785564 0.000281579529785564 0.131084474220489 turquoise

PKD1 0.526702542537365 -0.526702542537365 -0.205422310279562 0.000239890349423044 0.000239890349423044 0.180991875732861 turquoise

THEM5 0.538312912954286 -0.538312912954286 -0.25064903664372 0.000163809136896059 0.000163809136896059 0.100783195549076 brown

SAP130 0.537420293655338 -0.537420293655338 0.0536650826321143 0.000168767956210285 0.000168767956210285 0.729361157183684 turquoise

PLAU 0.51558387868262 -0.51558387868262 0.100064465724909 0.000341319578379017 0.000341319578379017 0.518104917255803 turquoise

EVC 0.520603257404531 -0.520603257404531 -0.0742559789351376 0.000291525508487945 0.000291525508487945 0.63190961797605 turquoise

NPEPPS 0.523693573120428 -0.523693573120428 0.197792877398931 0.000264228869568972 0.000264228869568972 0.19809518468002 turquoise

RAB2B 0.525779196044763 -0.525779196044763 -0.0360346606988105 0.00024713420153193 0.00024713420153193 0.816366329972739 turquoise

WNK3 0.528052228812981 -0.528052228812981 -0.374894938754136 0.00022964670019851 0.00022964670019851 0.0121631717345587 brown

AGO2 0.536226015042815 -0.536226015042815 -0.0120401094706417 0.0001756152836953 0.0001756152836953 0.938171212513351 turquoise

ABTB2 0.532927533762352 -0.532927533762352 -0.131975622586473 0.000195854045579026 0.000195854045579026 0.393121943948324 turquoise

PILRA 0.526758509939392 -0.526758509939392 0.249730522026224 0.000239457504129527 0.000239457504129527 0.102079219239307 turquoise

DLGAP4 0.517382983602961 -0.517382983602961 -0.0758268901343451 0.000322653291325293 0.000322653291325293 0.62469721929091 turquoise

SLC25A37 0.51539870429129 -0.51539870429129 -0.18556949099208 0.000343295009412421 0.000343295009412421 0.227817130853973 turquoise

STYX 0.52597723051908 -0.52597723051908 -0.149718080725258 0.000245564139232849 0.000245564139232849 0.332042000786659 turquoise

AMPH 0.516755591006869 -0.516755591006869 -0.111726171907079 0.000329055489310908 0.000329055489310908 0.470271316008323 brown

SCP2 0.521525220003019 -0.521525220003019 0.0425120409617937 0.000283127027810721 0.000283127027810721 0.7840856106495 blue

KAT6A 0.516450962065945 -0.516450962065945 -0.00741253093037273 0.000332205166511416 0.000332205166511416 0.961912074414554 brown

AXDND1 0.511991168442064 -0.511991168442064 -0.245057052387541 0.000381534085412695 0.000381534085412695 0.108872376721544 grey

AKR7A2 0.517514993943968 -0.517514993943968 0.0321547197148493 0.00032132058440764 0.00032132058440764 0.835850943250172 turquoise

SUCO 0.519470298154681 -0.519470298154681 0.108295973303369 0.00030215351823884 0.00030215351823884 0.484092104235887 turquoise

INPP1 0.524489525218227 -0.524489525218227 -0.0493061022896563 0.000257582802851362 0.000257582802851362 0.750608153629 turquoise

CATSPER1 0.534978559496268 -0.534978559496268 -0.414014452562111 0.000183035284547418 0.000183035284547418 0.00520968585327487 grey

FBXW5 0.521209852715377 -0.521209852715377 0.209338051231064 0.000285974921288506 0.000285974921288506 0.172636202890707 turquoise

FUT4 0.513119429308733 -0.513119429308733 -0.228375120470648 0.000368468244616585 0.000368468244616585 0.135950359261336 turquoise

RAB32 0.513742610490487 -0.513742610490487 0.0225369252977728 0.00036142553156363 0.00036142553156363 0.884546862874829 blue

EHMT1 0.52172293879825 -0.52172293879825 -0.087172677444921 0.000281354626760005 0.000281354626760005 0.573663345235372 blue

KIAA0319L 0.518315824859636 -0.518315824859636 0.0371395709534748 0.000313341642728537 0.000313341642728537 0.810837017161474 turquoise

MGRN1 0.51512989549805 -0.51512989549805 0.125149439484436 0.000346180981916122 0.000346180981916122 0.418262943255607 turquoise

HP1BP3 0.520912677148537 -0.520912677148537 0.188985457976674 0.000288682165989869 0.000288682165989869 0.219219330255278 turquoise

DTX3 0.515837919173813 -0.515837919173813 -0.247698232551282 0.000338626152692384 0.000338626152692384 0.104992175939608 blue

CCDC66 0.516078947573748 -0.516078947573748 -0.149548542368734 0.000336088408513315 0.000336088408513315 0.332596064107534 grey

RBM3 0.516621722048011 -0.516621722048011 0.186646277904971 0.000330436282118567 0.000330436282118567 0.225082298984287 turquoise

FXR2 0.522205969097782 -0.522205969097782 0.00976125403727194 0.000277066660838112 0.000277066660838112 0.949857074055685 turquoise

PCTP 0.524714426761041 -0.524714426761041 0.00211422865392783 0.000255732469448905 0.000255732469448905 0.989132769536926 turquoise

SAFB2 0.519627011729856 -0.519627011729856 0.0849733749882682 0.0003006628970814 0.0003006628970814 0.583405897793618 turquoise

ABHD2 0.518215480960867 -0.518215480960867 -0.033739057310318 0.000314331513617612 0.000314331513617612 0.827882156288167 turquoise

AGO3 0.517543433755399 -0.517543433755399 0.198235823186074 0.000321034121609404 0.000321034121609404 0.197072120515396 turquoise

NCAPD2 0.512907176562437 -0.512907176562437 -0.0857900629551355 0.00037089502583899 0.00037089502583899 0.579779434603921 turquoise

WDR73 0.523170227333294 -0.523170227333294 0.0116974140290339 0.000268682688077389 0.000268682688077389 0.93992777847109 turquoise

UBR1 0.511653573650676 -0.511653573650676 0.250545376683671 0.000385523873432406 0.000385523873432406 0.100928823973807 turquoise

MAP3K10 0.529424013310196 -0.529424013310196 -0.25695124767689 0.000219642097182639 0.000219642097182639 0.092228747385918 grey

B3GNTL1 0.516557912977785 -0.516557912977785 -0.0483743050669633 0.00033109627755097 0.00033109627755097 0.755174374675708 blue

BAG4 0.515120799814753 -0.515120799814753 0.22223183243117 0.000346279015775003 0.000346279015775003 0.147091146910722 turquoise

TNRC18 0.52793958834027 -0.52793958834027 -0.221130961593135 0.000230486225123042 0.000230486225123042 0.149156643841357 grey

SREBF1 0.524111870110742 -0.524111870110742 0.0720520859659472 0.000260717101629834 0.000260717101629834 0.642085219310775 turquoise

GTF2IRD2 0.512019879604074 -0.512019879604074 -0.0868571329592852 0.000381196490169333 0.000381196490169333 0.575056579477293 brown

SRSF2 0.5207684269367 -0.5207684269367 0.121871893311436 0.000290004592042244 0.000290004592042244 0.430650970152829 turquoise

TMEM219 0.514589568161191 -0.514589568161191 -0.272508292963055 0.000352048242308016 0.000352048242308016 0.0735153656786817 blue

MITD1 0.511065824731694 -0.511065824731694 0.223847436162061 0.000392559751820617 0.000392559751820617 0.144098161462379 turquoise

BAG3 0.509457841545669 -0.509457841545669 0.0238336472163806 0.000412403177739098 0.000412403177739098 0.877952769342598 turquoise

ZNF254 0.526553566788215 -0.526553566788215 -0.0105365208211857 0.000241045958220268 0.000241045958220268 0.945880254219868 brown

RCN3 0.522231530990027 -0.522231530990027 -0.12427535693338 0.000276841394519972 0.000276841394519972 0.421546747648691 turquoise

UMPS 0.52191922892067 -0.52191922892067 0.168243208977717 0.000279604944387288 0.000279604944387288 0.274975563784656 blue

NPR2 0.523104009152654 -0.523104009152654 -0.119433165015347 0.000269251027239894 0.000269251027239894 0.440000101345964 turquoise

SF3A3 0.522988759668726 -0.522988759668726 -0.168542651804523 0.000270242782555014 0.000270242782555014 0.274109727763592 brown

SETD1A 0.514253171379169 -0.514253171379169 0.0723400657356905 0.000355746175048487 0.000355746175048487 0.640751850889624 turquoise

TPX2 0.51592053627917 -0.51592053627917 -0.259187599320663 0.000337754353806794 0.000337754353806794 0.0893321766920294 turquoise

BRMS1L 0.516160491211567 -0.516160491211567 -0.153971584451165 0.000335233738560645 0.000335233738560645 0.318329675313023 turquoise

REC8 0.507829151777489 -0.507829151777489 -0.0270195204473022 0.000433417561732465 0.000433417561732465 0.861787466540995 blue

TGFB2 0.512311989664912 -0.512311989664912 -0.0790482885054009 0.000377777019308188 0.000377777019308188 0.61001552942715 blue

RCOR3 0.510063988335772 -0.510063988335772 0.224720531662994 0.00040481942208844 0.00040481942208844 0.142499558063605 turquoise

ATXN7L3 0.526380935988055 -0.526380935988055 -0.218915170000196 0.000242391349997002 0.000242391349997002 0.153378547176373 brown

C16orf58 0.508338879256101 -0.508338879256101 -0.0118855578461675 0.000426739499782001 0.000426739499782001 0.938963367755063 turquoise

DBN1 0.50806154808187 -0.50806154808187 0.175001194884121 0.000430361313814233 0.000430361313814233 0.255871611206226 turquoise

LIMA1 0.508618162803521 -0.508618162803521 0.165674859287348 0.000423119914782956 0.000423119914782956 0.28247578110462 turquoise

SERPINB9 0.509059101673085 -0.509059101673085 0.254016711843509 0.000417461425652855 0.000417461425652855 0.096139269620208 turquoise

ARMC6 0.50789507277374 -0.50789507277374 -0.228472069949333 0.000432548656774729 0.000432548656774729 0.135779724657652 brown

NOC3L 0.520792350601692 -0.520792350601692 0.168718379431738 0.000289784892423592 0.000289784892423592 0.273602449849265 blue

PPIE 0.510771962750839 -0.510771962750839 0.0986962485628398 0.000396120695251821 0.000396120695251821 0.523871543537566 turquoise

SLC35C2 0.513172386434437 -0.513172386434437 0.0785409637619087 0.000367864995141797 0.000367864995141797 0.612317874997071 turquoise

IBTK 0.504986785222215 -0.504986785222215 -0.00557671483225056 0.000472409070137327 0.000472409070137327 0.971340522695189 turquoise

DSEL 0.504330406048503 -0.504330406048503 0.127410376807456 0.000481849498547788 0.000481849498547788 0.409836580858663 turquoise

HOXD10 0.506351728444361 -0.506351728444361 -0.146429594951415 0.000453307882756507 0.000453307882756507 0.342891511729838 brown

GLT8D1 0.511117586459599 -0.511117586459599 -0.0076399536511898 0.000391935509510105 0.000391935509510105 0.960744400285975 turquoise

FGF19 0.523637829536827 -0.523637829536827 -0.365491407637487 0.000264700070026432 0.000264700070026432 0.0147007961867205 blue

TEX30 0.512403131831635 -0.512403131831635 0.094739105878444 0.000376715766500221 0.000376715766500221 0.540726255480382 turquoise

KNTC1 0.508575752225608 -0.508575752225608 0.0704985883820995 0.000423667782168688 0.000423667782168688 0.649297136629762 blue

KRIT1 0.50961467188496 -0.50961467188496 0.108418952658048 0.000410428849386314 0.000410428849386314 0.483592961627381 blue

ZNF653 0.51616496284509 -0.51616496284509 -0.107270282905658 0.000335186927463617 0.000335186927463617 0.488265593367484 blue

MAP2K7 0.503171433577433 -0.503171433577433 0.221191651657608 0.000498933159111248 0.000498933159111248 0.149042222099592 turquoise

SLC2A5 0.503075030304892 -0.503075030304892 -0.0742621527052365 0.00050037839028289 0.00050037839028289 0.631881206079809 grey

SOBP 0.518484101054706 -0.518484101054706 0.214446759376906 0.000311687942317574 0.000311687942317574 0.162157728918687 blue

RFX1 0.504968706153451 -0.504968706153451 0.119014519374803 0.000472666851762282 0.000472666851762282 0.441616231901303 turquoise

CRELD2 0.517062915654396 -0.517062915654396 -0.333449368549909 0.000325905240575521 0.000325905240575521 0.0269729465186475 brown

SLC38A6 0.510123829353031 -0.510123829353031 -0.0280125354014196 0.000404077574352762 0.000404077574352762 0.856759919931895 turquoise

MAP1LC3C 0.513512368309386 -0.513512368309386 -0.0506444645052178 0.000364013303278977 0.000364013303278977 0.744064324360752 grey

NSUN2 0.504739360583534 -0.504739360583534 0.15120476111099 0.000475947980571367 0.000475947980571367 0.32720806818122 turquoise

BRWD3 0.506807769736556 -0.506807769736556 0.0710702104870374 0.000447082466548354 0.000447082466548354 0.646639722486631 turquoise

ITGB1 0.501414879502614 -0.501414879502614 0.112677581395636 0.000525863554189914 0.000525863554189914 0.466475510733114 turquoise

MCM3AP 0.515832045440268 -0.515832045440268 0.0450201590926998 0.000338688211118905 0.000338688211118905 0.771678516039704 blue

NUF2 0.519787462630409 -0.519787462630409 -0.243802861593071 0.000299143603010335 0.000299143603010335 0.110752771098251 grey

LCE1D 0.507571426554458 -0.507571426554458 -0.341643243210423 0.000436829723046593 0.000436829723046593 0.0232234813618233 brown

GATB 0.507189530879407 -0.507189530879407 0.116936811795167 0.000441930266189841 0.000441930266189841 0.449685259010545 blue

CLK1 0.510031012490192 -0.510031012490192 0.0861514998790442 0.000405228746406472 0.000405228746406472 0.578177751099434 blue

TAC4 0.507012432397645 -0.507012432397645 -0.00129557858180394 0.000444313689557449 0.000444313689557449 0.99334054383619 grey

NAPB 0.516168617369276 -0.516168617369276 0.25913830171139 0.000335148674614115 0.000335148674614115 0.0893952576566399 turquoise

LZTS1 0.509058629254557 -0.509058629254557 -0.0284936663629442 0.000417467451510204 0.000417467451510204 0.854325996936294 blue

KIF1B 0.505125022372321 -0.505125022372321 0.0357955089899849 0.000470442178390662 0.000470442178390662 0.8175642896783 turquoise

TMEM263 0.504275674422036 -0.504275674422036 0.0466285730498348 0.000482644284209608 0.000482644284209608 0.763751357695185 turquoise

CNTLN 0.512661970781298 -0.512661970781298 0.204156308993001 0.000373716484342 0.000373716484342 0.183754241080212 turquoise

D2HGDH 0.516163427894995 -0.516163427894995 -0.180231050950201 0.000335202995356492 0.000335202995356492 0.241712334461791 turquoise

NHLRC3 0.508964423189868 -0.508964423189868 0.216384308114806 0.000418670646217945 0.000418670646217945 0.158307161957588 turquoise

ABCB6 0.501559746093846 -0.501559746093846 -0.0274975664294934 0.000523594180610224 0.000523594180610224 0.859366475867419 turquoise

PRPF38A 0.500065841897566 -0.500065841897566 -0.095347496767518 0.000547424357778239 0.000547424357778239 0.538118035433364 turquoise

GKAP1 0.502572518356048 -0.502572518356048 -0.217172725824717 0.000507972858298791 0.000507972858298791 0.156759560463664 brown

ABHD12 0.495812023803346 -0.495812023803346 -0.0113697686053973 0.000620702185256951 0.000620702185256951 0.941607465572909 blue

CAPN15 0.504275939160943 -0.504275939160943 -0.15300220228472 0.000482640436969034 0.000482640436969034 0.321422831499864 blue

EMX1 0.5074530191614 -0.5074530191614 -0.248895233081507 0.000438405463990085 0.000438405463990085 0.103268874323724 blue

CCNO 0.503076802676122 -0.503076802676122 -0.0810982521617767 0.000500351785863273 0.000500351785863273 0.600750371171672 turquoise

PHF21B 0.51405798751113 -0.51405798751113 -0.290387917185557 0.000357907791695844 0.000357907791695844 0.0558512841235722 brown

GPR68 0.498403783722857 -0.498403783722857 -0.00165350227429338 0.000575076753967716 0.000575076753967716 0.991500825514526 turquoise

ASIC3 0.513804284387672 -0.513804284387672 -0.18835800378155 0.000360735176678798 0.000360735176678798 0.220781516703504 blue

NCSTN 0.498145479629648 -0.498145479629648 -0.148189490452657 0.000579484957404227 0.000579484957404227 0.337058315906754 brown

TMEM8A 0.509897188917913 -0.509897188917913 0.139875170723336 0.000406893703632959 0.000406893703632959 0.365158541450298 turquoise

CCNL2 0.500809161305918 -0.500809161305918 -0.0202181512001858 0.000535448231640435 0.000535448231640435 0.896357249844214 turquoise

MAP4K5 0.497739717581998 -0.497739717581998 0.144712480247914 0.00058647088405764 0.00058647088405764 0.348642447435727 turquoise

TUBG1 0.502864264647769 -0.502864264647769 0.0527313389264336 0.000503551189886042 0.000503551189886042 0.733896296567209 blue

OSBPL10 0.496462620419946 -0.496462620419946 0.0788296268775577 0.000608954041617894 0.000608954041617894 0.611007407246025 turquoise

GPN1 0.512103589140401 -0.512103589140401 0.0184816103354054 0.000380213740496934 0.000380213740496934 0.905216765060309 blue

ABCC5 0.494886876529685 -0.494886876529685 0.398722851849045 0.000637757863059338 0.000637757863059338 0.00734308534565351 turquoise

CD99 0.500779397861874 -0.500779397861874 0.0439312772206515 0.000535923213953337 0.000535923213953337 0.777058256873075 turquoise

ZNF426 0.494566627929816 -0.494566627929816 0.0797725350642647 0.000643758957470213 0.000643758957470213 0.606735186497921 blue

DCHS1 0.491147192898902 -0.491147192898902 -0.167316836917246 0.000711061770892349 0.000711061770892349 0.277665543101458 turquoise

ACAD8 0.502541144820069 -0.502541144820069 -0.368341025127098 0.000508450421562053 0.000508450421562053 0.0138882248615716 grey

RAB22A 0.496706154653996 -0.496706154653996 0.0396533162933074 0.00060460795530946 0.00060460795530946 0.798291693290327 turquoise

TK2 0.490347565257308 -0.490347565257308 0.258133976876975 0.000727681385483922 0.000727681385483922 0.0906879274440013 turquoise

KDM4B 0.508940545905906 -0.508940545905906 -0.0718726597836673 0.000418976100659906 0.000418976100659906 0.642916539235138 turquoise

DMTF1 0.495155802477233 -0.495155802477233 0.0514454769317594 0.000632757267719968 0.000632757267719968 0.740156299624662 blue

PAGR1 0.492995846906373 -0.492995846906373 -0.175430592906622 0.000673932537943821 0.000673932537943821 0.254688573121202 turquoise

UBAP1L 0.500341347529135 -0.500341347529135 -0.173359028534064 0.000542957750180361 0.000542957750180361 0.260429865504395 blue

NXT1 0.509018889605334 -0.509018889605334 -0.0557744358595707 0.000417974624317424 0.000417974624317424 0.719149891946338 turquoise

EIF4G2 0.492000880509666 -0.492000880509666 0.149485587758325 0.000693694272151719 0.000693694272151719 0.33280195064065 turquoise

ARHGEF11 0.508646569847028 -0.508646569847028 0.250269682146799 0.000422753303985852 0.000422753303985852 0.101316924319406 turquoise

HOXD9 0.506369742297493 -0.506369742297493 -0.214691125292645 0.000453060505278735 0.000453060505278735 0.16166837240959 blue

DRD4 0.499224126579648 -0.499224126579648 -0.275555320416177 0.000561275557517007 0.000561275557517007 0.0702281465096683 brown

ZNF532 0.502055367092773 -0.502055367092773 0.0451801030893076 0.000515896484065125 0.000515896484065125 0.770889178950364 turquoise

SYNDIG1 0.492979570793171 -0.492979570793171 -0.289210837515218 0.000674251710081166 0.000674251710081166 0.0568980575194514 turquoise

BIRC5 0.498039737229726 -0.498039737229726 -0.216181977977995 0.000581298276384345 0.000581298276384345 0.158706111510884 turquoise

PBK 0.492614682160859 -0.492614682160859 -0.203338345241667 0.000681442747316547 0.000681442747316547 0.185554931395362 grey

NPHP3 0.489652651773799 -0.489652651773799 0.0233389304142237 0.000742405979876123 0.000742405979876123 0.880467566342813 blue

SYVN1 0.488251143218143 -0.488251143218143 -0.177941204816081 0.000772916176733906 0.000772916176733906 0.247845005519235 grey

WIZ 0.490029800200942 -0.490029800200942 0.119609741219545 0.000734381800770091 0.000734381800770091 0.43931943228474 turquoise

DLG5 0.492044645030176 -0.492044645030176 0.0778583921145791 0.000692814244998769 0.000692814244998769 0.615421354740002 turquoise

ZBTB49 0.489009647163632 -0.489009647163632 -0.0152163378970956 0.000756267507025766 0.000756267507025766 0.921905579334647 brown

CDH3 0.496171935561332 -0.496171935561332 -0.220940560410459 0.000614178221045302 0.000614178221045302 0.149516035182864 brown

C1orf174 0.49796898215796 -0.49796898215796 0.0554724721426413 0.000582514456646353 0.000582514456646353 0.720608770161515 turquoise

DOCK6 0.498861297589062 -0.498861297589062 0.180388459068824 0.000567342605726565 0.000567342605726565 0.241294578880784 turquoise

INPP5E 0.496238487590404 -0.496238487590404 -0.085414924875807 0.000612978617092136 0.000612978617092136 0.581443951646318 blue

METRN 0.504958850239517 -0.504958850239517 0.0243499964827816 0.00047280743620504 0.00047280743620504 0.875329273694275 turquoise

PGAP1 0.503696453364063 -0.503696453364063 0.0858907643406002 0.000491127917900275 0.000491127917900275 0.579332981658338 turquoise

MDK 0.487400144061015 -0.487400144061015 -0.0088095135976891 0.000791984559993794 0.000791984559993794 0.954740772264027 turquoise

DUSP8 0.505760733872196 -0.505760733872196 -0.0225288003463415 0.000461491426928749 0.000461491426928749 0.88458820467964 blue

PIM1 0.486280320083209 -0.486280320083209 0.0526343555184955 0.000817716975911562 0.000817716975911562 0.734367855795773 turquoise

KCNMA1 0.493757017058075 -0.493757017058075 0.209729020366311 0.000659156381392702 0.000659156381392702 0.171817455931267 turquoise

ARL4C 0.486248308748813 -0.486248308748813 -0.0356045824183083 0.000818463423542193 0.000818463423542193 0.818520974856477 turquoise

WDR34 0.496940033501313 -0.496940033501313 0.069328916315863 0.000600460373891013 0.000600460373891013 0.654748222562086 turquoise

APBA1 0.493916137268993 -0.493916137268993 -0.120977521437883 0.000656104396137478 0.000656104396137478 0.434066671308546 turquoise

PSMC6 0.503020382807898 -0.503020382807898 0.165815384817136 0.000501199307687431 0.000501199307687431 0.282061988877919 turquoise

ANKRD52 0.501108602765156 -0.501108602765156 0.0179562718648242 0.000530690550623133 0.000530690550623133 0.907899237621561 turquoise

TIAL1 0.496916685528598 -0.496916685528598 0.0639857249531972 0.000600873275034369 0.000600873275034369 0.679871959197004 turquoise

CRYZ 0.493078145693358 -0.493078145693358 0.194812108683135 0.000672320741833127 0.000672320741833127 0.205077255044876 turquoise

NDN 0.490129151365407 -0.490129151365407 0.0402759492298315 0.000732280968217161 0.000732280968217161 0.795191935628781 turquoise

SFT2D3 0.487359539993111 -0.487359539993111 -0.259963313627311 0.000792904799462842 0.000792904799462842 0.0883441187849326 grey

GCGR 0.485398184323419 -0.485398184323419 -0.297191181784305 0.000838510980004929 0.000838510980004929 0.0501002261940056 blue

ZNF518B 0.486016761632975 -0.486016761632975 -0.0315617568948357 0.000823880850423155 0.000823880850423155 0.838837610813123 turquoise

TMEM129 0.483531178369872 -0.483531178369872 0.0972038278989203 0.000884085966406562 0.000884085966406562 0.530197606379835 turquoise

TRAF5 0.494518544845053 -0.494518544845053 0.087235905472757 0.000644664334598668 0.000644664334598668 0.573384357924847 grey

PFKFB3 0.48618347629545 -0.48618347629545 -0.200279861986457 0.00081997706743354 0.00081997706743354 0.192399333145253 turquoise

TMEM38B 0.490940475118198 -0.490940475118198 -0.138876402648957 0.000715325346570481 0.000715325346570481 0.36862635120381 blue

C1orf53 0.482623697049572 -0.482623697049572 -0.0485564212204751 0.000907026953566867 0.000907026953566867 0.754281270538784 blue

PGRMC2 0.494824575759121 -0.494824575759121 0.0876492187437741 0.000638921366305166 0.000638921366305166 0.571562183891913 turquoise

PIGZ 0.496568779952593 -0.496568779952593 0.127013884089463 0.000607056098533462 0.000607056098533462 0.411307206532717 turquoise

TNFSF11 0.48513308009873 -0.48513308009873 -0.298185469488537 0.000844851767356358 0.000844851767356358 0.0493013127035723 grey

APEX1 0.501982903971721 -0.501982903971721 0.0625561000839325 0.000517015559511035 0.000517015559511035 0.686653985807206 turquoise

PCDHA6 0.494747553565528 -0.494747553565528 -0.196587822283205 0.000640362429561749 0.000640362429561749 0.200897410936484 brown

TNNT2 0.494178251953808 -0.494178251953808 -0.223627502467056 0.000651104529131077 0.000651104529131077 0.144502932381929 brown

LUC7L3 0.48741750838802 -0.48741750838802 0.306886494139764 0.000791591311212162 0.000791591311212162 0.0427405917778239 turquoise

ST6GALNAC4 0.488885457140361 -0.488885457140361 -0.201698488690171 0.00075897124245383 0.00075897124245383 0.189202764562042 brown

PIGG 0.500301681694172 -0.500301681694172 -0.115398804599142 0.000543598806571229 0.000543598806571229 0.455709794529533 grey

ZNF766 0.485965543954498 -0.485965543954498 0.0951862012405344 0.000825083491795594 0.000825083491795594 0.538808928940614 turquoise

STRA6 0.484875044669697 -0.484875044669697 -0.263501613550225 0.000851064587445715 0.000851064587445715 0.0839445212808423 brown

CDK6 0.486127467818685 -0.486127467818685 -0.116684811767896 0.000821286711488376 0.000821286711488376 0.450669377750053 turquoise

GOLM1 0.484304507948279 -0.484304507948279 -0.0419555540064706 0.000864946734419298 0.000864946734419298 0.786845729884797 turquoise

ALG13 0.4887229793228 -0.4887229793228 0.166662527012979 0.000762521593184539 0.000762521593184539 0.279575885998031 turquoise

SLC35D1 0.485578677626021 -0.485578677626021 -0.358320727106546 0.000834218288743991 0.000834218288743991 0.0169260422435462 brown

PLEKHN1 0.496122192388898 -0.496122192388898 -0.265959253395133 0.000615076218149984 0.000615076218149984 0.0809906058295882 brown

ZFP1 0.482396382389641 -0.482396382389641 -0.142445031724176 0.000912856023380052 0.000912856023380052 0.35632643676838 blue

EGLN2 0.489353127385884 -0.489353127385884 0.147588133576873 0.000748834422264563 0.000748834422264563 0.339044565932896 turquoise

ATP1A3 0.484452504133942 -0.484452504133942 -0.0490347825288236 0.000861326439035791 0.000861326439035791 0.751936880314954 blue

DENND4B 0.495833351646545 -0.495833351646545 0.161175622308837 0.000620313860477137 0.000620313860477137 0.295933893521456 turquoise

TMEM158 0.492638377747202 -0.492638377747202 -0.226177881136358 0.000680973690388284 0.000680973690388284 0.139860495948966 grey

CCDC81 0.495560188232766 -0.495560188232766 -0.225911389565041 0.000625303961325817 0.000625303961325817 0.140340347965524 brown

NEK9 0.481218004921581 -0.481218004921581 0.0678554423859162 0.000943612713205322 0.000943612713205322 0.661640431868386 blue

LOX 0.47939296863593 -0.47939296863593 -0.201535099754237 0.000993074927541804 0.000993074927541804 0.189568991402092 turquoise

TCF25 0.485371563813182 -0.485371563813182 0.101424486628074 0.000839145765701381 0.000839145765701381 0.512404361450096 turquoise

ZNF106 0.488115852576479 -0.488115852576479 0.160216886364357 0.000775919922608635 0.000775919922608635 0.29885423585648 turquoise

ACSF3 0.49678975415922 -0.49678975415922 -0.39428098938732 0.000603122470923573 0.000603122470923573 0.00808952847357416 grey

FAM180B 0.493483350372043 -0.493483350372043 -0.131248542222552 0.000664435164466915 0.000664435164466915 0.39575699548259 grey

DPP4 0.478854752796413 -0.478854752796413 -0.0232015562437673 0.00100809663417966 0.00100809663417966 0.881166088544563 turquoise

C14orf132 0.490284701463917 -0.490284701463917 0.046048093798466 0.000729002578546021 0.000729002578546021 0.766609577268474 turquoise

DCAF17 0.487054753040356 -0.487054753040356 0.125540477700788 0.000799842982383323 0.000799842982383323 0.416798581117159 blue

NANOGNB 0.495888484130194 -0.495888484130194 -0.0945321331686689 0.000619311049467311 0.000619311049467311 0.541614950063554 brown

MAPK7 0.487747384688828 -0.487747384688828 -0.0364530248535349 0.000784153708532781 0.000784153708532781 0.81427165431494 turquoise

TAF9 0.481736753724542 -0.481736753724542 0.133928844713669 0.000929960776513994 0.000929960776513994 0.38609408419768 turquoise

HOXD11 0.479819442626545 -0.479819442626545 -0.174153372934402 0.000981314063017306 0.000981314063017306 0.258218242770514 turquoise

PCDHGA3 0.487850124953862 -0.487850124953862 -0.0734463382048323 0.000781850055480698 0.000781850055480698 0.635640138134111 turquoise

AP1G2 0.483585061777616 -0.483585061777616 -0.0232980166563452 0.000882740244525521 0.000882740244525521 0.880675595872208 turquoise

DDX18 0.482291639847779 -0.482291639847779 0.154017429731934 0.000915553191396157 0.000915553191396157 0.31818385669094 turquoise

SFPQ 0.482226608518319 -0.482226608518319 0.325381293303337 0.000917231354880767 0.000917231354880767 0.0311438239577931 blue

EGR4 0.491651881636316 -0.491651881636316 -0.427365858270575 0.000700747909884184 0.000700747909884184 0.00381062851374676 grey

ZBED4 0.489546101930019 -0.489546101930019 -0.0104950908734865 0.00074468709055989 0.00074468709055989 0.946092741868515 blue

SSC5D 0.483844036875314 -0.483844036875314 -0.0534102177485702 0.00087629790840655 0.00087629790840655 0.730598123527887 turquoise

TPM2 0.478095913183709 -0.478095913183709 0.0312290108099859 0.00102962019729203 0.00102962019729203 0.840514587779215 turquoise

ANKS6 0.479484283567361 -0.479484283567361 -0.000873408679808723 0.000990546214149008 0.000990546214149008 0.99551052911457 turquoise

ABCB4 0.475336079128072 -0.475336079128072 -0.0660984872422039 0.00111139588302502 0.00111139588302502 0.669894857683508 turquoise

SAP30 0.479916981865003 -0.479916981865003 -0.0289372573874742 0.000978641745382316 0.000978641745382316 0.852083161904927 turquoise

FAM76B 0.477401056757278 -0.477401056757278 0.00331343438219759 0.00104968727836236 0.00104968727836236 0.982969537090234 turquoise

DCDC2B 0.485153310311247 -0.485153310311247 -0.208100385969689 0.000844366393869361 0.000844366393869361 0.175246589400168 brown

TUSC1 0.492276222131442 -0.492276222131442 -0.291215552795013 0.00068817425404309 0.00068817425404309 0.0551245685099667 grey

PERP 0.476981176407887 -0.476981176407887 -0.0782383141803868 0.00106198147098777 0.00106198147098777 0.613693122908848 turquoise

NOL4 0.475791149668181 -0.475791149668181 -0.312393125833183 0.00109752591034708 0.00109752591034708 0.0389692873648161 brown

CPT2 0.479875535953518 -0.479875535953518 -0.0819840674384884 0.000979776459807133 0.000979776459807133 0.596765836468269 blue

SLITRK5 0.481561336166699 -0.481561336166699 -0.147970414934298 0.000934557392237529 0.000934557392237529 0.337781074295193 grey

SLC35F5 0.484307328041769 -0.484307328041769 -0.017724350931902 0.000864877622221782 0.000864877622221782 0.909083791499942 brown

MAP1A 0.481534667191424 -0.481534667191424 0.0571122907562567 0.000935257994921023 0.000935257994921023 0.712698230163547 blue

ATXN3 0.488361635764843 -0.488361635764843 0.00493516499287635 0.000770470721149781 0.000770470721149781 0.974636392270114 blue

BCL6 0.48485204367329 -0.48485204367329 0.0962220507360611 0.000851620367719301 0.000851620367719301 0.534379446934186 turquoise

PIDD1 0.486733479727165 -0.486733479727165 -0.222017560578821 0.000807215169099644 0.000807215169099644 0.14749151098316 grey

UBAP2L 0.477702682390824 -0.477702682390824 0.178875674191484 0.00104093406958236 0.00104093406958236 0.245329754269813 turquoise

LIMD1 0.477239060151736 -0.477239060151736 0.181170698150216 0.00105441546485818 0.00105441546485818 0.239225811927998 turquoise

SLC35B2 0.486690929744041 -0.486690929744041 -0.0364261294504985 0.000808196094920909 0.000808196094920909 0.814406276538887 turquoise

MARVELD1 0.476722892604773 -0.476722892604773 -0.0416319229832676 0.00106960767115234 0.00106960767115234 0.788452097302647 turquoise

SPC25 0.477012121640461 -0.477012121640461 -0.135219754025034 0.00106107102695407 0.00106107102695407 0.381490142350548 brown

LRRC32 0.481800493290195 -0.481800493290195 -0.0313968065232806 0.000928295558998475 0.000928295558998475 0.839668841630053 turquoise

ATP11A 0.486084310310103 -0.486084310310103 0.160248670537638 0.000822297136689605 0.000822297136689605 0.298757124106783 blue

MMS19 0.478380780631076 -0.478380780631076 -0.106568522426501 0.00102149271744607 0.00102149271744607 0.491131761438827 grey

GNPTG 0.475882056637284 -0.475882056637284 -0.212922982820204 0.00109477373264234 0.00109477373264234 0.165233499893095 brown

CTDSPL 0.474039676263396 -0.474039676263396 0.0903357311956153 0.00115176844366615 0.00115176844366615 0.559783360359902 turquoise

TRAPPC4 0.474252247958483 -0.474252247958483 0.12895364765535 0.00114506034056831 0.00114506034056831 0.404141219233006 turquoise

ZFP3 0.470752099125539 -0.470752099125539 0.0544525909649726 0.00126006409592013 0.00126006409592013 0.725543348312133 blue

LRRC75B 0.476441369066994 -0.476441369066994 -0.293868009858547 0.0010779756081367 0.0010779756081367 0.0528465869773305 turquoise

IL5 0.48190792165086 -0.48190792165086 -0.104672324228477 0.000925494978904155 0.000925494978904155 0.498919665805945 brown

FOXK2 0.481236161596375 -0.481236161596375 -0.222792827426567 0.000943131879427385 0.000943131879427385 0.14604672913579 grey

ADAMTS2 0.474298218778899 -0.474298218778899 -0.107322172576356 0.00114361422742685 0.00114361422742685 0.488054008509129 turquoise

PYROXD1 0.472645631829855 -0.472645631829855 0.162556207027613 0.00119663396567971 0.00119663396567971 0.291761039042342 turquoise

RP2 0.469453700154234 -0.469453700154234 0.149540423656562 0.00130526934760288 0.00130526934760288 0.332622611061401 turquoise

NPHP4 0.479518707257515 -0.479518707257515 0.118185079575595 0.000989594437180358 0.000989594437180358 0.444827837054125 turquoise

SPTLC2 0.467569193709982 -0.467569193709982 -0.217766564409948 0.00137344580406149 0.00137344580406149 0.15560122270189 grey

GMEB2 0.476869755586293 -0.476869755586293 -0.137755178117601 0.0010652653674382 0.0010652653674382 0.372542778896761 turquoise

PLK3 0.473379941448226 -0.473379941448226 0.0400848884729645 0.00117281099167835 0.00117281099167835 0.79614279639335 turquoise

RBBP7 0.474537696769597 -0.474537696769597 -0.0423259246866246 0.00113610718958869 0.00113610718958869 0.785008439120905 brown

SH3GLB2 0.480934005676792 -0.480934005676792 0.21669752735932 0.000951162241497341 0.000951162241497341 0.157691008098977 turquoise

C2CD5 0.480677282565514 -0.480677282565514 0.19546834241131 0.000958033020077283 0.000958033020077283 0.203525510214067 blue

AFAP1 0.468838206634588 -0.468838206634588 -0.00102106694685372 0.00132719781493223 0.00132719781493223 0.994751550004944 turquoise

FAM181B 0.48372340937252 -0.48372340937252 -0.462419674366206 0.0008792934288273 0.0008792934288273 0.0015761507001481 grey

CCDC17 0.469657186138842 -0.469657186138842 -0.290973626239067 0.00129809083940381 0.00129809083940381 0.0553362050080791 blue

CPT1C 0.479061725462734 -0.479061725462734 -0.334540964845297 0.00100229617794655 0.00100229617794655 0.0264461070314004 turquoise

TXLNA 0.472374111346592 -0.472374111346592 0.0461866982758222 0.00120555128203172 0.00120555128203172 0.765926823406737 turquoise

RABGGTA 0.476044731182937 -0.476044731182937 0.0617824561728303 0.00108986415703107 0.00108986415703107 0.690334296061067 blue

CEP95 0.472782680170632 -0.472782680170632 -0.0233276692795835 0.00119215533461614 0.00119215533461614 0.880524823760432 blue

KLHL5 0.478400587123496 -0.478400587123496 0.0198811386153286 0.00102092975610499 0.00102092975610499 0.89807568668472 grey

PVALB 0.47696756744723 -0.47696756744723 -0.208476160024881 0.00106238208248629 0.00106238208248629 0.17445105401838 brown

GPBP1 0.474338925947436 -0.474338925947436 0.293871871009627 0.00114233505179433 0.00114233505179433 0.0528433270860408 turquoise

GPATCH8 0.465465217178136 -0.465465217178136 0.0580795105667291 0.00145328415409234 0.00145328415409234 0.708046195294978 turquoise

FBXO31 0.474383172648259 -0.474383172648259 0.119158653646062 0.00114094609560448 0.00114094609560448 0.441059449655072 turquoise

NEDD1 0.470678049687186 -0.470678049687186 0.241990312691339 0.00126260426756196 0.00126260426756196 0.113513838944886 turquoise

TUBA1A 0.465215441701532 -0.465215441701532 0.105318358197961 0.00146303065636211 0.00146303065636211 0.496259247655848 turquoise

SYNC 0.470973811214037 -0.470973811214037 0.0965466418717632 0.00125248570091522 0.00125248570091522 0.532995090540702 turquoise

CSTF3 0.470341250752898 -0.470341250752898 -0.0128738285366465 0.00127421528215727 0.00127421528215727 0.933899016693054 turquoise

RASL12 0.463308155742328 -0.463308155742328 -0.292051196644089 0.00153938861546922 0.00153938861546922 0.0543985470227829 grey

SLC25A25 0.474530138988279 -0.474530138988279 0.102863759365996 0.00113634343495029 0.00113634343495029 0.506406140854549 turquoise

GPR17 0.468124292995352 -0.468124292995352 -0.390183095498865 0.00135304242746993 0.00135304242746993 0.00883531398315739 brown

POLR1C 0.47656076772644 -0.47656076772644 -0.123362695455695 0.00107441953441687 0.00107441953441687 0.424990986870423 blue

RCN2 0.46614451036444 -0.46614451036444 0.109975197783648 0.00142706836427642 0.00142706836427642 0.477299872121841 turquoise

SUPT20H 0.466195321542434 -0.466195321542434 0.219333432295877 0.00142512440962649 0.00142512440962649 0.152574971798362 turquoise

CCDC86 0.46619884193864 -0.46619884193864 -0.103122636927079 0.0014249898120319 0.0014249898120319 0.505331048795108 blue

ERCC1 0.470466550329783 -0.470466550329783 -0.101115673149162 0.00126988458225502 0.00126988458225502 0.513695987747433 turquoise

KLHL17 0.465542309880871 -0.465542309880871 -0.257315801358132 0.00145028758026923 0.00145028758026923 0.0917516743381129 brown

NPRL3 0.463459877190429 -0.463459877190429 0.00978787876173883 0.00153318756488352 0.00153318756488352 0.949720478969057 turquoise

FBXW8 0.464086419347881 -0.464086419347881 -0.231711684748655 0.00150781407262448 0.00150781407262448 0.130169098891044 grey

RABEP2 0.478337217636775 -0.478337217636775 -0.123081628438113 0.00102273188542544 0.00102273188542544 0.426054870871339 blue

RHBDL1 0.463477356096876 -0.463477356096876 -0.203366844702186 0.00153247460514925 0.00153247460514925 0.185491981154338 brown

ZBTB20 0.469470812479042 -0.469470812479042 0.0799580934482917 0.00130466430868043 0.00130466430868043 0.605895957836535 blue

AGMO 0.471636829995172 -0.471636829995172 0.083297297784443 0.00123006420257473 0.00123006420257473 0.590880162170375 grey

ACTG1 0.461490566592008 -0.461490566592008 0.141603330700063 0.00161542461741384 0.00161542461741384 0.359204793107687 turquoise

LCE1A 0.468172191279498 -0.468172191279498 -0.292707623522365 0.00135129455392045 0.00135129455392045 0.0538336400674986 brown

TDRD12 0.460231937731459 -0.460231937731459 -0.287460535819347 0.00167001062441174 0.00167001062441174 0.0584836194148374 brown

GRAMD1A 0.467745642800324 -0.467745642800324 -0.0324711464517727 0.0013669307566975 0.0013669307566975 0.834258077883826 brown

PCDHGA2 0.476793911187487 -0.476793911187487 -0.227415880252685 0.00106750589630364 0.00106750589630364 0.137647265435553 turquoise

CASP8 0.464921756765897 -0.464921756765897 0.021232843930262 0.00147456474632819 0.00147456474632819 0.891186162337245 turquoise

ZNF100 0.467867915202283 -0.467867915202283 0.155609674144737 0.00136243220215888 0.00136243220215888 0.31314563347774 blue

CREBBP 0.469450693807968 -0.469450693807968 0.0860509418298939 0.00130537566847374 0.00130537566847374 0.578623165936289 turquoise

CACNB4 0.461510720226272 -0.461510720226272 -0.0966978010404963 0.00161456358949563 0.00161456358949563 0.532351005431855 grey

SART1 0.461857177454355 -0.461857177454355 -0.255821641225766 0.00159982531417483 0.00159982531417483 0.0937192045001524 brown

KRT7 0.473433806894322 -0.473433806894322 -0.38741732699608 0.0011710801884483 0.0011710801884483 0.00937147186657002 brown

CABP4 0.465016290074481 -0.465016290074481 -0.297894263493925 0.00147084329644426 0.00147084329644426 0.0495342236699749 brown

SH3YL1 0.46813472529261 -0.46813472529261 0.231132994392478 0.00135266156770976 0.00135266156770976 0.131158385441692 turquoise

ESYT1 0.467524359404671 -0.467524359404671 -0.104243505227502 0.00137510561489775 0.00137510561489775 0.500689600499113 blue

TLE3 0.460391298642995 -0.460391298642995 0.282180958881611 0.00166301004363768 0.00166301004363768 0.0634814283505474 turquoise

CEP192 0.459231682635626 -0.459231682635626 0.0526929839980759 0.00171455044544548 0.00171455044544548 0.734082776918335 turquoise

CTBP1 0.469709082488311 -0.469709082488311 0.0437713590658129 0.00129626568466645 0.00129626568466645 0.777849225230441 blue

ARF4 0.45811620946797 -0.45811620946797 0.0671148151849698 0.00176545920566468 0.00176545920566468 0.665115242908564 turquoise

NRGN 0.460257311230463 -0.460257311230463 -0.0365145545019995 0.0016688942443312 0.0016688942443312 0.813963693759585 turquoise

IFT81 0.466368072814779 -0.466368072814779 0.180648280363683 0.00141853279846387 0.00141853279846387 0.240606094543518 turquoise

IL36A 0.464494405216077 -0.464494405216077 -0.301159729160348 0.00149149260557638 0.00149149260557638 0.0469727940642162 brown

E4F1 0.463624834886708 -0.463624834886708 -0.177255305366004 0.00152647068848224 0.00152647068848224 0.249702228010509 turquoise

RARS2 0.463242037214837 -0.463242037214837 0.0750538331013493 0.00154209791882646 0.00154209791882646 0.628242219792478 turquoise

GOLT1B 0.458448581518953 -0.458448581518953 0.0364189289528917 0.00175015196433093 0.00175015196433093 0.814442318799152 turquoise

GJA1 0.45664053524573 -0.45664053524573 0.165768655537689 0.00183486197466279 0.00183486197466279 0.282199544172664 turquoise

SIRT3 0.465010238394466 -0.465010238394466 0.0735600673019971 0.00147108128016292 0.00147108128016292 0.635115576183631 grey

IER5L 0.464859201423083 -0.464859201423083 0.0145662652962239 0.00147703192725249 0.00147703192725249 0.925232290577636 turquoise

ZCCHC9 0.46321165542005 -0.46321165542005 0.223646477006005 0.00154334427522598 0.00154334427522598 0.144467978151762 turquoise

MAGI3 0.467075506938414 -0.467075506938414 0.140405320944781 0.00139182107191104 0.00139182107191104 0.36332581887759 blue

LRRC17 0.455667195583067 -0.455667195583067 -0.0694389266681243 0.00188195085188648 0.00188195085188648 0.654234773402026 turquoise

FGFR1OP 0.468432110825481 -0.468432110825481 0.0995744479133034 0.00134184467645062 0.00134184467645062 0.520166553432649 turquoise

OVCA2 0.468135627441088 -0.468135627441088 0.0990343355805183 0.00135262863676931 0.00135262863676931 0.522443663318677 turquoise

LPCAT1 0.459081381001362 -0.459081381001362 0.0343965264954967 0.00172133331101158 0.00172133331101158 0.824580224142392 turquoise

HECTD3 0.457472492565549 -0.457472492565549 0.190793545852113 0.00179544283776983 0.00179544283776983 0.214760527162241 turquoise

GMPPA 0.468659900011725 -0.468659900011725 -0.20528099685719 0.00133361129808554 0.00133361129808554 0.181298734354262 turquoise

CSNK1G2 0.459252635173225 -0.459252635173225 0.0091213372659746 0.00171360677495564 0.00171360677495564 0.953140511781432 blue

WWC3 0.456061710362955 -0.456061710362955 -0.00778129094580202 0.00186273740752498 0.00186273740752498 0.960018761980286 turquoise

CLEC18B 0.455604027996556 -0.455604027996556 -0.273713277060916 0.00188504343761168 0.00188504343761168 0.0722011944005519 turquoise

OSBPL7 0.45682916343033 -0.45682916343033 0.0456832683348583 0.0018258580079986 0.0018258580079986 0.768407510285803 blue

HAPLN3 0.466773280262567 -0.466773280262567 -0.0989537193038567 0.00140317756585148 0.00140317756585148 0.522783963858969 turquoise

XPO6 0.46843801953739 -0.46843801953739 0.0595411082893544 0.00134163053827229 0.00134163053827229 0.701036279038458 turquoise

TRUB1 0.459646159654789 -0.459646159654789 -0.0759482252647917 0.00169596830660063 0.00169596830660063 0.624141570374399 turquoise

DHDDS 0.464662183645601 -0.464662183645601 0.0684835339725977 0.00148482626392692 0.00148482626392692 0.658699096743117 turquoise

ICMT 0.455639937096144 -0.455639937096144 0.0223324246780182 0.00188328483345592 0.00188328483345592 0.885587505539699 blue

KMT2D 0.458194415645061 -0.458194415645061 0.0681445577868685 0.00176184682877639 0.00176184682877639 0.660285884046873 turquoise

TMEM236 0.463445402209622 -0.463445402209622 0.0374796678460656 0.00153377821808365 0.00153377821808365 0.809136882973372 grey

ZSCAN5A 0.465933006673875 -0.465933006673875 -0.241285574278135 0.00143518548368943 0.00143518548368943 0.114601365211884 brown

PRKRIP1 0.466127888180758 -0.466127888180758 -0.154440368821409 0.00142770481349637 0.00142770481349637 0.316840617390946 blue

CEP120 0.457735590000493 -0.457735590000493 -0.162117472593158 0.00178313399488212 0.00178313399488212 0.293082973627185 brown

PLOD2 0.452217611761899 -0.452217611761899 -0.131471357480658 0.00205756351348345 0.00205756351348345 0.394948387954033 turquoise

MSL3 0.461127812560647 -0.461127812560647 0.203876679371066 0.00163099240441285 0.00163099240441285 0.184368417555924 turquoise

LUC7L 0.458885219008702 -0.458885219008702 0.105806922236947 0.00173022153292526 0.00173022153292526 0.494252157093541 turquoise

C12orf49 0.465861293944491 -0.465861293944491 -0.289698615018032 0.00143794697751376 0.00143794697751376 0.056462386007188 grey

CACNB3 0.461118068121521 -0.461118068121521 0.00957206927538038 0.00163141242039581 0.00163141242039581 0.950827705387697 blue

NUP62 0.454693263134558 -0.454693263134558 0.148219026399015 0.00193013557048172 0.00193013557048172 0.336960946368438 blue

UBE2G2 0.454518809104415 -0.454518809104415 -0.127308902505667 0.0019388808837018 0.0019388808837018 0.410212670610293 brown

CCDC14 0.463723541969505 -0.463723541969505 0.320703605129341 0.00152246395438007 0.00152246395438007 0.0337970609439556 turquoise

NIN 0.457228879944269 -0.457228879944269 -0.0997811653960192 0.00180690711552543 0.00180690711552543 0.519296340813895 turquoise

MXRA5 0.450417436459283 -0.450417436459283 -0.0479591707369096 0.00215483620378176 0.00215483620378176 0.757211385903277 turquoise

GLIS3 0.456916837994943 -0.456916837994943 -0.329360114499465 0.00182168628664229 0.00182168628664229 0.0290247791773417 grey

BAG2 0.453206325731959 -0.453206325731959 -0.0558503059332382 0.00200580668363078 0.00200580668363078 0.718783495366237 turquoise

PDPN 0.450637172302685 -0.450637172302685 0.11117955706342 0.00214274911693881 0.00214274911693881 0.472459539033078 turquoise

FCRL1 0.461206428116262 -0.461206428116262 -0.298090786717608 0.00162760733173817 0.00162760733173817 0.0493769440737813 brown

ATRX 0.46010957122692 -0.46010957122692 0.322064525743298 0.00167540378858345 0.00167540378858345 0.0330066006230229 turquoise

USP44 0.454698781464818 -0.454698781464818 -0.216109089049964 0.00192985950895062 0.00192985950895062 0.158850011632388 brown

ATXN2L 0.462346411389647 -0.462346411389647 -0.0469072019437413 0.00157921651899788 0.00157921651899788 0.762380516735264 blue

PITPNM3 0.451001165732853 -0.451001165732853 -0.222097854162951 0.00212285853198272 0.00212285853198272 0.147341389567665 turquoise

FADS2 0.452636061433013 -0.452636061433013 -0.0720814012386768 0.00203551634127312 0.00203551634127312 0.64194943641094 turquoise

OR2A42 0.453244509553478 -0.453244509553478 -0.152186648958158 0.00200383114592154 0.00200383114592154 0.324039741635269 brown

SFSWAP 0.460059806730768 -0.460059806730768 0.0673506969166809 0.00167760151334804 0.00167760151334804 0.664007796302524 turquoise

TRPC4AP 0.461145110664185 -0.461145110664185 0.104191791078403 0.00163024703786462 0.00163024703786462 0.500903265291832 turquoise

CD44 0.4548101513716 -0.4548101513716 0.0720315782264546 0.00192429554713703 0.00192429554713703 0.64218021420806 blue

NKAIN4 0.451998540434297 -0.451998540434297 -0.333425692406155 0.00206918984173862 0.00206918984173862 0.0269844694100588 grey

TOP3B 0.452351934283446 -0.452351934283446 -0.155390618477315 0.00205046347308985 0.00205046347308985 0.313835755721512 brown

PAIP1 0.455273588136434 -0.455273588136434 0.0644317214288739 0.00190129465750049 0.00190129465750049 0.67776125531513 turquoise

RCC1 0.457104640584988 -0.457104640584988 -0.161528655196831 0.00181277866844182 0.00181277866844182 0.29486319700887 turquoise

FAM83G 0.462044162789456 -0.462044162789456 -0.434306443615266 0.00159192063985251 0.00159192063985251 0.00322280342380288 blue

PPP1R14B 0.456490693337324 -0.456490693337324 0.034670411336477 0.00184204250804892 0.00184204250804892 0.823205602497498 turquoise

AGA 0.44922225623569 -0.44922225623569 -0.0262068743214657 0.00222164038960986 0.00222164038960986 0.865905850188411 turquoise

PIAS3 0.45424642806452 -0.45424642806452 -0.0188312682364084 0.00195260532869305 0.00195260532869305 0.903431924763018 turquoise

VENTX 0.456076904553358 -0.456076904553358 0.0258136897444733 0.0018620009127139 0.0018620009127139 0.867899725217422 blue

MRPL4 0.456081204295568 -0.456081204295568 -0.00649450283014883 0.00186179254182765 0.00186179254182765 0.966626353025122 blue

DUT 0.450167547442275 -0.450167547442275 0.174953589534679 0.00216865508462805 0.00216865508462805 0.256002995309416 turquoise

SNAPC1 0.458109213498189 -0.458109213498189 -0.0242646114888982 0.0017657826719207 0.0017657826719207 0.875763012100821 turquoise

INSIG1 0.44659412417716 -0.44659412417716 0.000897469273349542 0.0023750056592766 0.0023750056592766 0.995386854834497 turquoise

MIIP 0.449667626607504 -0.449667626607504 -0.0854790057986925 0.00219653584529333 0.00219653584529333 0.581159467087366 brown

FAM114A1 0.44760668134921 -0.44760668134921 0.0541435206918482 0.00231484590461123 0.00231484590461123 0.72704093092541 turquoise

BNC2 0.454104988043899 -0.454104988043899 -0.0305079334642585 0.00195976589299018 0.00195976589299018 0.844151065641947 turquoise

PXDN 0.444827225444363 -0.444827225444363 -0.0778758411470312 0.00248329191061952 0.00248329191061952 0.615341935476443 turquoise

MKRN3 0.449496346481598 -0.449496346481598 -0.197235618483044 0.00220616072941573 0.00220616072941573 0.199387586052917 grey

TTC14 0.456621608843271 -0.456621608843271 0.0529062159892947 0.00183576757241658 0.00183576757241658 0.733046242139735 turquoise

SEC22A 0.455240389763171 -0.455240389763171 0.0468887368504715 0.00190293420457868 0.00190293420457868 0.762471341946179 turquoise

APCDD1L 0.449090468813278 -0.449090468813278 -0.362054220973534 0.00222911736019657 0.00222911736019657 0.0157343075781392 turquoise

SRRM2 0.449060107177713 -0.449060107177713 0.142290750988702 0.00223084307532051 0.00223084307532051 0.356852977484637 turquoise

VPS13C 0.449480015982343 -0.449480015982343 0.426788629241507 0.00220708034398512 0.00220708034398512 0.00386349753905627 turquoise

AIP 0.45224895739555 -0.45224895739555 -0.122912921452504 0.00205590470045662 0.00205590470045662 0.426694172522584 brown

C1QTNF6 0.444910744581801 -0.444910744581801 0.140550987754289 0.00247807702087001 0.00247807702087001 0.362823223754066 turquoise

RIN1 0.45287639788514 -0.45287639788514 -0.241601869927121 0.00202294818912257 0.00202294818912257 0.114112296298903 grey

RBM5 0.452856319102722 -0.452856319102722 0.154752926078218 0.00202399555633572 0.00202399555633572 0.315850253942273 turquoise

ZNF862 0.444679573247521 -0.444679573247521 -0.057294729301061 0.00249253490330864 0.00249253490330864 0.711819961252677 turquoise

TMEM41B 0.454282042955631 -0.454282042955631 -0.0650176055442267 0.00195080593460491 0.00195080593460491 0.674992227590194 turquoise

AUTS2 0.446672078829513 -0.446672078829513 0.0031578273245523 0.00237032557738908 0.00237032557738908 0.983769220713326 turquoise

CDKN3 0.446917241123911 -0.446917241123911 -0.0728401824402885 0.0023556599982337 0.0023556599982337 0.638438927306493 turquoise

ARHGEF17 0.453432830192699 -0.453432830192699 0.0472115582610881 0.00199411303774189 0.00199411303774189 0.760883918773207 grey

RBM25 0.45623097205008 -0.45623097205008 0.185110388058983 0.00185454747499904 0.00185454747499904 0.228990073698025 turquoise

BARHL2 0.447535725251298 -0.447535725251298 -0.292431101564518 0.00231901742635726 0.00231901742635726 0.0540710309768029 brown

RBPMS2 0.44768574602003 -0.44768574602003 -0.197753218602372 0.00231020547848425 0.00231020547848425 0.198186966169027 grey

STAT2 0.446418758180525 -0.446418758180525 0.130548178690868 0.0023855637247144 0.0023855637247144 0.398304917547351 turquoise

SLC2A1 0.444098058929898 -0.444098058929898 -0.197739811099811 0.00252923310182966 0.00252923310182966 0.198218001638457 turquoise

CLASRP 0.4489815386384 -0.4489815386384 0.167606293861494 0.00223531428300037 0.00223531428300037 0.276823175757483 blue

FANCC 0.446046723425538 -0.446046723425538 -0.0520647034285124 0.0024080995630507 0.0024080995630507 0.737139600488122 turquoise

IDNK 0.448468109072896 -0.448468109072896 0.149112877688283 0.00226472797619547 0.00226472797619547 0.334022484419471 turquoise

KHNYN 0.444606715823988 -0.444606715823988 0.0523305264230981 0.00249710692286498 0.00249710692286498 0.735845778373844 turquoise

ABHD1 0.454261362741308 -0.454261362741308 0.149917322529294 0.00195185059576119 0.00195185059576119 0.33139159963755 turquoise

SRGAP2C 0.442789580407581 -0.442789580407581 0.330856112804177 0.00261355437812591 0.00261355437812591 0.0282596217397254 turquoise

UGGT1 0.444084418148936 -0.444084418148936 0.122098435835603 0.00253009962890996 0.00253009962890996 0.42978817250033 turquoise

SPICE1 0.447557656009154 -0.447557656009154 -0.158662832432789 0.00231772740555507 0.00231772740555507 0.303627179469604 grey

F2R 0.447484706285278 -0.447484706285278 -0.0818171215993376 0.00232202093806281 0.00232202093806281 0.597515897773066 brown

PIP5K1C 0.445129360602009 -0.445129360602009 0.153689309102194 0.00246447242905717 0.00246447242905717 0.319228428821311 turquoise

BIRC2 0.447153982387811 -0.447153982387811 0.0838043863726345 0.00234157418575654 0.00234157418575654 0.5886143861616 turquoise

MRAS 0.449756756434221 -0.449756756434221 0.160957277428571 0.00219154198214768 0.00219154198214768 0.296597354907333 turquoise

ADAMTS12 0.440754923190747 -0.440754923190747 -0.232137548281583 0.00274959335820754 0.00274959335820754 0.129444646125751 turquoise

RANBP3 0.449409386811485 -0.449409386811485 -0.0764540791395279 0.00221106156204106 0.00221106156204106 0.62182725692693 turquoise

PCNA 0.442074320732713 -0.442074320732713 -0.0770137566075268 0.00266068592148054 0.00266068592148054 0.619270891044174 grey

NDC1 0.451484326963791 -0.451484326963791 0.0319189663172666 0.00209670800094415 0.00209670800094415 0.837038128151094 brown

CDKN2B 0.446905247951805 -0.446905247951805 -0.18713725648851 0.00235637556201365 0.00235637556201365 0.223842840467051 brown

MAZ 0.442366502024951 -0.442366502024951 0.0862372834081207 0.00264134318075399 0.00264134318075399 0.57779790184885 turquoise

ZBTB1 0.445308156430684 -0.445308156430684 -0.151655653128489 0.00245339485531019 0.00245339485531019 0.325750744150509 turquoise

SLC36A4 0.444276398680972 -0.444276398680972 0.195236760657925 0.00251792815058101 0.00251792815058101 0.204072170155411 blue

CIRBP 0.448429725751794 -0.448429725751794 0.0401107273058793 0.00226694056607967 0.00226694056607967 0.796014186005425 turquoise

SFXN3 0.439347263342102 -0.439347263342102 -0.0891361963936651 0.00284732115002106 0.00284732115002106 0.565028600270663 blue

ERF 0.440048118657393 -0.440048118657393 -0.011667315414481 0.00279828840403492 0.00279828840403492 0.940082069565159 brown

SLC25A32 0.443595980194394 -0.443595980194394 -0.141938366344562 0.00256130041530263 0.00256130041530263 0.358057392401553 blue

FBXO11 0.444978547730364 -0.444978547730364 0.0909149582644539 0.00247385053048332 0.00247385053048332 0.557258733577708 turquoise

ALG3 0.44867415996896 -0.44867415996896 -0.151808780328915 0.00225288280987112 0.00225288280987112 0.32525674981179 blue

GOLIM4 0.437141467302618 -0.437141467302618 0.1711742800584 0.00300660898255702 0.00300660898255702 0.266577657182846 turquoise

CA9 0.439094284788566 -0.439094284788566 -0.379915806825084 0.00286520457725087 0.00286520457725087 0.0109685937659369 turquoise

UNK 0.43746633391988 -0.43746633391988 0.0364350879732135 0.00298266833564912 0.00298266833564912 0.814361434971158 turquoise

TCEAL7 0.442204725798887 -0.442204725798887 -0.110940944090012 0.00265203759825634 0.00265203759825634 0.473416452424277 brown

SHC1 0.437665317514675 -0.437665317514675 0.10258953700803 0.00296808737142986 0.00296808737142986 0.507546221265333 turquoise

HOXC4 0.442195096490506 -0.442195096490506 -0.0224999702355389 0.00265267535613455 0.00265267535613455 0.884734902007492 turquoise

DDX39B 0.439217802591636 -0.439217802591636 0.0203304899745986 0.00285646061779248 0.00285646061779248 0.895784534940482 blue

DCDC1 0.442541125756834 -0.442541125756834 -0.256352174213645 0.00262984209954128 0.00262984209954128 0.0930168919320557 brown

ANGPTL6 0.438811438473399 -0.438811438473399 -0.035651089346574 0.00288531630328569 0.00288531630328569 0.818287916250246 turquoise

GLS 0.441814132046013 -0.441814132046013 0.0119256285187656 0.0026780155852508 0.0026780155852508 0.938757979671291 turquoise

S100A2 0.436107638239118 -0.436107638239118 -0.348446357027212 0.00308392335023754 0.00308392335023754 0.020451363308653 turquoise

ARHGAP28 0.439895208733716 -0.439895208733716 0.136515795466401 0.00280892235530079 0.00280892235530079 0.376900720348567 blue

MTERF1 0.436880645587061 -0.436880645587061 -0.121901874654422 0.00302595197256535 0.00302595197256535 0.430536729245778 grey

MED6 0.44669928811554 -0.44669928811554 0.118725067214763 0.00236869395807845 0.00236869395807845 0.44273553939598 blue

PMS2 0.445374383699648 -0.445374383699648 0.0952291453222273 0.00244930279642944 0.00244930279642944 0.538624940339151 turquoise

FBXL5 0.443694363894548 -0.443694363894548 0.297094951584834 0.0025549886685146 0.0025549886685146 0.0501780993021667 turquoise

GALNT7 0.435984884903336 -0.435984884903336 0.0116831160433689 0.00309321839503254 0.00309321839503254 0.940001072333232 turquoise

CXCR5 0.436757416108619 -0.436757416108619 0.0983859916904923 0.00303512886059086 0.00303512886059086 0.525183580321203 turquoise

ETNPPL 0.432941084928289 -0.432941084928289 -0.151972494098591 0.0033317074326688 0.0033317074326688 0.324729122594886 blue

TOR4A 0.434437378477582 -0.434437378477582 -0.236995486876269 0.0032125258622835 0.0032125258622835 0.121392849370412 blue

PTGER2 0.443122239997692 -0.443122239997692 -0.259403234721706 0.00259188565205971 0.00259188565205971 0.0890566567234192 turquoise

PLXDC1 0.441462312833089 -0.441462312833089 0.0958852156590904 0.00270160622511297 0.00270160622511297 0.535817869585754 blue

MIER2 0.440054090488281 -0.440054090488281 0.139361611861202 0.0027978738193035 0.0027978738193035 0.366939203079048 turquoise

AEBP2 0.434346960275358 -0.434346960275358 0.0439049155723894 0.00321962004177025 0.00321962004177025 0.777188628447564 turquoise

VPS4A 0.439144398400427 -0.439144398400427 -0.12453260054207 0.00286165414632493 0.00286165414632493 0.42057880973476 brown

LTBP1 0.435126048712631 -0.435126048712631 -0.0986318221812283 0.00315894184835228 0.00315894184835228 0.524143861177651 turquoise

MAPK1IP1L 0.438065904739522 -0.438065904739522 -0.0623645408343188 0.00293892336824605 0.00293892336824605 0.687564591808269 turquoise

SENP5 0.435665647006641 -0.435665647006641 0.086427794116401 0.00311750682866394 0.00311750682866394 0.576954725979239 turquoise

USP16 0.442106791888064 -0.442106791888064 0.286950613647113 0.00265853015097734 0.00265853015097734 0.0589521396846898 turquoise

RPL28 0.440393765803865 -0.440393765803865 0.023357222935868 0.00277438118378413 0.00277438118378413 0.880374559010367 turquoise

ACAA2 0.433307882878171 -0.433307882878171 0.370076835733164 0.00330213820333459 0.00330213820333459 0.0134122324746922 turquoise

TBC1D21 0.443577678581457 -0.443577678581457 -0.417745228801388 0.00256247605836149 0.00256247605836149 0.00477971843413501 yellow

RRM2B 0.438246969012028 -0.438246969012028 0.0698098302407487 0.00292582420224693 0.00292582420224693 0.65250482521893 blue

ANAPC5 0.437588003722475 -0.437588003722475 0.117055516151266 0.00297374525127944 0.00297374525127944 0.449222098040439 turquoise

MLLT1 0.439692097293506 -0.439692097293506 0.0846550381644704 0.00282310248308796 0.00282310248308796 0.584822211205612 turquoise

SH3PXD2B 0.436854041969994 -0.436854041969994 0.112571852920172 0.00302793107515266 0.00302793107515266 0.466896520775671 turquoise

CRLF3 0.438478160561745 -0.438478160561745 0.0781436511626331 0.00290917319217446 0.00290917319217446 0.614123543614159 turquoise

ZNF707 0.438175264609539 -0.438175264609539 0.105114470054936 0.00293100553364278 0.00293100553364278 0.497098086466403 turquoise

IL9R 0.442610303812989 -0.442610303812989 -0.213121727881057 0.00262529809975917 0.00262529809975917 0.164829947206874 brown

TMEM200B 0.440279609680678 -0.440279609680678 -0.126524074064309 0.00278225701499978 0.00278225701499978 0.413128115410482 turquoise

CLIP1 0.436829191566848 -0.436829191566848 0.207407857736695 0.00302978077961943 0.00302978077961943 0.176719539182586 turquoise

SIGMAR1 0.437197803530169 -0.437197803530169 -0.108976191767614 0.00300244528960124 0.00300244528960124 0.481334643865791 blue

CPSF1 0.434290258252394 -0.434290258252394 -0.232006047429456 0.00322407587514387 0.00322407587514387 0.129668024500774 brown

ZNF630 0.434882573909016 -0.434882573909016 -0.157527692878762 0.00317779574402906 0.00317779574402906 0.307144191580225 brown

WDR75 0.442566893531225 -0.442566893531225 0.128492580736181 0.00262814871986426 0.00262814871986426 0.405837961976283 turquoise

ZNF655 0.433940807395594 -0.433940807395594 0.267553473547149 0.00325165656244852 0.00325165656244852 0.0791183728571729 turquoise

SLC24A5 0.435792104598464 -0.435792104598464 -0.255669433002887 0.00310786565034433 0.00310786565034433 0.0939214504475534 brown

NEUROG1 0.434617382765201 -0.434617382765201 -0.132970085754874 0.00319844363339991 0.00319844363339991 0.389534494339689 brown

FAM149B1 0.439199152379922 -0.439199152379922 0.112860397215681 0.00285777938032228 0.00285777938032228 0.465748018819187 turquoise

SPATS1 0.429398989701148 -0.429398989701148 -0.140839351502288 0.00362942175894827 0.00362942175894827 0.361829518414726 blue

VOPP1 0.432461980672815 -0.432461980672815 0.0609066555591189 0.00337067992597598 0.00337067992597598 0.694509105251556 turquoise

LASP1 0.435107765913624 -0.435107765913624 0.0869965578301549 0.00316035418976988 0.00316035418976988 0.574440782014293 turquoise

S100PBP 0.432660618322105 -0.432660618322105 0.122732616768406 0.00335447358245981 0.00335447358245981 0.427378018162928 turquoise

MAPK15 0.43439737891557 -0.43439737891557 -0.1783132551154 0.00321566251910305 0.00321566251910305 0.246841505782536 brown

MRPS25 0.434376152537734 -0.434376152537734 0.225681474190163 0.00321732812558142 0.00321732812558142 0.140755319540212 turquoise

CC2D1A 0.437847004307831 -0.437847004307831 -0.062690341980778 0.00295482862969386 0.00295482862969386 0.686016107559714 brown

AANAT 0.436832813383112 -0.436832813383112 -0.199142236239143 0.00302951113307431 0.00302951113307431 0.194990225875151 brown

SRRT 0.431016722771313 -0.431016722771313 0.0314151560480778 0.00349067693517507 0.00349067693517507 0.839576364731645 turquoise

WWP2 0.431727542247369 -0.431727542247369 -0.101403394047607 0.00343119890335391 0.00343119890335391 0.51249253016164 turquoise

CD1B 0.432446506164308 -0.432446506164308 -0.153429884105713 0.00337194533088195 0.00337194533088195 0.320055837590985 blue

RHOXF1 0.438311260316169 -0.438311260316169 -0.115263615246097 0.00292118538733159 0.00292118538733159 0.456241429540396 brown

ALPK3 0.430153711121898 -0.430153711121898 -0.380953457458179 0.00356410312687327 0.00356410312687327 0.010734658568528 turquoise

CGB7 0.42834551272587 -0.42834551272587 -0.223602783522951 0.00372234955359221 0.00372234955359221 0.144548478121943 brown

UBE2E3 0.435559736702368 -0.435559736702368 0.0145276389319495 0.00312560167705928 0.00312560167705928 0.925429999125646 blue

ATP6V1A 0.427852555414004 -0.427852555414004 0.344675513246758 0.00376654463189983 0.00376654463189983 0.0219513571978122 turquoise

FOXL1 0.42893084658105 -0.42893084658105 -0.164223870349813 0.00367046295776525 0.00367046295776525 0.286771553440319 brown

SLTM 0.430008214636578 -0.430008214636578 0.0503041563892109 0.00357661459572462 0.00357661459572462 0.745726569992483 grey

CDK19 0.435645506827316 -0.435645506827316 0.147005202997797 0.00311904474673626 0.00311904474673626 0.340976847934732 turquoise

CA12 0.424947319187337 -0.424947319187337 -0.266163688382419 0.00403645957187766 0.00403645957187766 0.080748600604954 turquoise

PTPRM 0.428232432911638 -0.428232432911638 0.287378668946063 0.00373244715167188 0.00373244715167188 0.0585586378245827 turquoise

TEP1 0.427095791846432 -0.427095791846432 0.00431580199261382 0.00383528482057148 0.00383528482057148 0.977818676798938 grey

SNAPC3 0.43651086548003 -0.43651086548003 0.00596080448942153 0.00305356290213489 0.00305356290213489 0.969367539839147 blue

C9orf92 0.424859422103793 -0.424859422103793 -0.382752277068812 0.0040448828104277 0.0040448828104277 0.0103392411609589 grey

NOXO1 0.430844237520514 -0.430844237520514 -0.423494178686492 0.00350524524675221 0.00350524524675221 0.00417770276647996 yellow

KPNA2 0.432437519139597 -0.432437519139597 0.0803639341694905 0.0033726804227232 0.0033726804227232 0.604062201560722 turquoise

CDK11B 0.428061812444942 -0.428061812444942 0.196448729220298 0.00374772831373734 0.00374772831373734 0.201222642082694 turquoise

SGK2 0.422769660068501 -0.422769660068501 -0.199411390441322 0.00424972492964727 0.00424972492964727 0.19437502365204 brown

SLC39A4 0.425358519267975 -0.425358519267975 -0.142266646278385 0.00399725740271152 0.00399725740271152 0.356935286472205 brown

KAT5 0.430326101568851 -0.430326101568851 0.14719155185569 0.00354932866587462 0.00354932866587462 0.340358406303133 turquoise

LRRC59 0.426451631148427 -0.426451631148427 0.0581207630859644 0.00389465943312493 0.00389465943312493 0.707848015020268 blue

IL3RA 0.432112397031377 -0.432112397031377 0.108938395422941 0.00339936842653638 0.00339936842653638 0.481487645648116 turquoise

LAX1 0.422810173847063 -0.422810173847063 -0.288781601566917 0.00424566913105364 0.00424566913105364 0.0572836667137291 brown

AARS 0.4255715256946 -0.4255715256946 0.111522036037956 0.00397708146894872 0.00397708146894872 0.471087886356974 turquoise

ANKRD9 0.426835082951542 -0.426835082951542 0.173720635173511 0.00385921916801622 0.00385921916801622 0.259421514998725 turquoise

PTBP2 0.424347735106459 -0.424347735106459 0.390669853710291 0.00409422400916038 0.00409422400916038 0.00874373900260837 turquoise

LOXL1 0.423320508457341 -0.423320508457341 -0.0406643205549973 0.00419486886555497 0.00419486886555497 0.793260015222348 turquoise

ZC3H4 0.431560491366256 -0.431560491366256 0.209465981363495 0.00344509645841051 0.00344509645841051 0.17236798944978 blue

DBR1 0.433383521208881 -0.433383521208881 -0.140903412118939 0.00329606937918915 0.00329606937918915 0.361608988036847 grey

PDGFRB 0.42338039247299 -0.42338039247299 0.21384210098072 0.00418894279513898 0.00418894279513898 0.163373220434366 turquoise

CCDC144A 0.430450102414108 -0.430450102414108 0.0620020907920284 0.00353873458121916 0.00353873458121916 0.689288747749081 turquoise

SLC5A1 0.42293757827072 -0.42293757827072 -0.144705619317118 0.00423293681944011 0.00423293681944011 0.34866554375448 grey

HMGXB3 0.429243545018095 -0.429243545018095 0.216550524572583 0.00364300447983136 0.00364300447983136 0.157979968395544 turquoise

SAR1B 0.423781143373101 -0.423781143373101 -0.156568119601912 0.00414947281565574 0.00414947281565574 0.310137439385447 brown

N4BP2L2 0.432610131192285 -0.432610131192285 0.246878104647254 0.00335858620700584 0.00335858620700584 0.10618554046428 turquoise

CMPK1 0.432187307950483 -0.432187307950483 -0.0118044002025421 0.00339320291464893 0.00339320291464893 0.939379365084263 turquoise

LRRC40 0.42313792835643 -0.42313792835643 0.0354350740008316 0.00421298208186819 0.00421298208186819 0.819370557382512 turquoise

CITED4 0.429356933496059 -0.429356933496059 -0.275289346716321 0.00363309223513625 0.00363309223513625 0.0705103730819593 brown

STK17A 0.426550468344239 -0.426550468344239 0.224929904007776 0.00388549733788919 0.00388549733788919 0.14211816365477 turquoise

SEC61G 0.422497415491356 -0.422497415491356 0.0620048241534918 0.00427706700621688 0.00427706700621688 0.689275739456518 turquoise

TBC1D2 0.419414890794884 -0.419414890794884 0.301206395267851 0.0045975382281143 0.0045975382281143 0.0469369829421435 turquoise

UBD 0.420151876889728 -0.420151876889728 -0.0511430816069385 0.00451907113169103 0.00451907113169103 0.741630889638751 brown

B3GNT8 0.423701153793617 -0.423701153793617 -0.186333781046536 0.00415732492333436 0.00415732492333436 0.225873642430197 brown

MAPRE3 0.426361513613361 -0.426361513613361 0.0497135642049915 0.00390302969750796 0.00390302969750796 0.748614041464426 turquoise

FAM71D 0.430371024429488 -0.430371024429488 -0.120065629922726 0.00354548744829849 0.00354548744829849 0.43756476171887 brown

NDRG1 0.420642592878328 -0.420642592878328 -0.067464475217471 0.00446747502573275 0.00446747502573275 0.663473868459534 turquoise

ZNF23 0.425690856629849 -0.425690856629849 -0.169909453394849 0.00396581744086377 0.00396581744086377 0.270180451555362 grey

FOXA3 0.428218604450533 -0.428218604450533 -0.192366332623479 0.00373368362530773 0.00373368362530773 0.210933550234106 brown

MYO18A 0.427119861770728 -0.427119861770728 0.141234787752961 0.00383308164994758 0.00383308164994758 0.360469515530955 turquoise

ASL 0.421900579574185 -0.421900579574185 0.19850513945936 0.00433754679579517 0.00433754679579517 0.196451910797333 turquoise

APPL1 0.423437356520808 -0.423437356520808 0.112133339182832 0.00418331247374462 0.00418331247374462 0.468644845883915 turquoise

GSDMA 0.418936151057988 -0.418936151057988 -0.107451296131735 0.0046491443509674 0.0046491443509674 0.487527702408147 brown

PDK1 0.422822372799261 -0.422822372799261 -0.207406311294802 0.00424444856741277 0.00424444856741277 0.176722838253026 turquoise

NOL9 0.421066987784598 -0.421066987784598 0.12225671545 0.00442326767893174 0.00442326767893174 0.429185931972523 turquoise

WBP1 0.425469943378949 -0.425469943378949 0.149383202905734 0.00398669219020506 0.00398669219020506 0.333136959027573 turquoise

ATF6B 0.426546496632004 -0.426546496632004 0.133793532363149 0.00388586514700505 0.00388586514700505 0.386578552190078 brown

PTCD1 0.424321085620261 -0.424321085620261 0.127902781729707 0.00409680813373172 0.00409680813373172 0.408014409796155 turquoise

PCDHGC3 0.420305873867647 -0.420305873867647 0.226964364245126 0.00450282347966578 0.00450282347966578 0.138451427330174 turquoise

PUM1 0.426136226919789 -0.426136226919789 0.331538662939689 0.0039240235954533 0.0039240235954533 0.0279161237779344 turquoise

SPACA3 0.422477612940082 -0.422477612940082 -0.340048440342419 0.00427906179922413 0.00427906179922413 0.0239169264984764 brown

GSKIP 0.418027893750271 -0.418027893750271 0.180203314928847 0.0047484407526163 0.0047484407526163 0.241785995683219 turquoise

SAP25 0.422429706104533 -0.422429706104533 -0.19227416333653 0.0042838910152118 0.0042838910152118 0.211156500703117 brown

KLK12 0.426413412638016 -0.426413412638016 -0.462644271884166 0.00389820731079801 0.00389820731079801 0.00156678489082264 grey

RNF4 0.421488623357296 -0.421488623357296 0.278879724822231 0.00437972646994042 0.00437972646994042 0.0667754204024709 turquoise

ANKRD65 0.423484748661651 -0.423484748661651 -0.365415701487622 0.00417863328028053 0.00417863328028053 0.0147229229560255 blue

U2AF2 0.425017727585993 -0.425017727585993 0.210812678084433 0.00402972335573946 0.00402972335573946 0.169562762453391 turquoise

GIPC1 0.419710675379175 -0.419710675379175 -0.139892877987637 0.00456590430417808 0.00456590430417808 0.365097237994228 brown

XAB2 0.417931218982661 -0.417931218982661 -0.064998580723256 0.0047591179492616 0.0047591179492616 0.675082077084691 brown

SMC5 0.418045684800513 -0.418045684800513 0.129801203314899 0.00474647810630499 0.00474647810630499 0.401032879818253 turquoise

CST6 0.421367311123466 -0.421367311123466 -0.233545311678337 0.00439221554900072 0.00439221554900072 0.127071313821524 turquoise

CDX2 0.417957015204047 -0.417957015204047 -0.15149543651624 0.00475626685269286 0.00475626685269286 0.326268112243643 brown

TCF7L1 0.423946872533134 -0.423946872533134 0.0641254514247831 0.00413324540015195 0.00413324540015195 0.679210434603136 turquoise

COL3A1 0.414871224915971 -0.414871224915971 -0.00802520655056376 0.00510807416187164 0.00510807416187164 0.958766552873223 turquoise

SYPL1 0.421682977781799 -0.421682977781799 0.284998891922497 0.004359782344755 0.004359782344755 0.0607732033603555 turquoise

NF2 0.418839422285134 -0.418839422285134 0.162675847618507 0.00465963244744012 0.00465963244744012 0.291401225575088 turquoise

SLC39A10 0.423074711173776 -0.423074711173776 -0.0510744093160631 0.00421926957610994 0.00421926957610994 0.741965888450905 blue

SLC35G1 0.416651417586293 -0.416651417586293 -0.131605993339559 0.00490245049676448 0.00490245049676448 0.394460254796761 brown

TTBK1 0.425867439672248 -0.425867439672248 -0.267539695874374 0.00394920041007157 0.00394920041007157 0.0791344065842528 brown

TMEM126A 0.418874465770076 -0.418874465770076 0.244901687716931 0.00465583037473458 0.00465583037473458 0.109103983692227 turquoise

C6orf223 0.420983727881923 -0.420983727881923 -0.250526676753116 0.0044319102552735 0.0044319102552735 0.100955112076266 grey

UACA 0.41759559759201 -0.41759559759201 0.202754365739328 0.00479634814989461 0.00479634814989461 0.18684819211742 grey

JKAMP 0.424560579432386 -0.424560579432386 0.241205356108116 0.00407363619676189 0.00407363619676189 0.114725654045594 turquoise

C6orf62 0.422219785969687 -0.422219785969687 0.245969100200689 0.00430510805756761 0.00430510805756761 0.107520308623652 turquoise

LRP6 0.420539330076604 -0.420539330076604 0.230582987165486 0.00447828961657798 0.00447828961657798 0.132103834561222 turquoise

GBE1 0.418726392892855 -0.418726392892855 -0.0992452910205339 0.0046719141292835 0.0046719141292835 0.521553689491337 turquoise

TIPRL 0.418913708246781 -0.418913708246781 0.150554332127539 0.0046515759411111 0.0046515759411111 0.329317488839307 turquoise

FAM50A 0.421211379141251 -0.421211379141251 0.119417827464187 0.00440831439672569 0.00440831439672569 0.440059252321009 turquoise

PBRM1 0.421969575146905 -0.421969575146905 0.237592713282422 0.00433051722492261 0.00433051722492261 0.120429641169568 blue

C6orf120 0.422287620970985 -0.422287620970985 0.0642618445426459 0.00429824182352241 0.00429824182352241 0.67856492109856 turquoise

PPIL3 0.418050450614768 -0.418050450614768 0.345255305301881 0.00474595247851804 0.00474595247851804 0.0217148957970778 turquoise

OSGEPL1 0.415301588310226 -0.415301588310226 0.193610135624796 0.00505768817372586 0.00505768817372586 0.207940923491538 turquoise

IL1RAPL1 0.421133999201513 -0.421133999201513 -0.616356993586792 0.00441632242568738 0.00441632242568738 8.40942948122566e-06 yellow

WWP1 0.421114147777671 -0.421114147777671 0.329908328270766 0.00441837889028821 0.00441837889028821 0.0287424140265066 turquoise

FLNC 0.421428397315253 -0.421428397315253 -0.226973442272113 0.00438592285160677 0.00438592285160677 0.138435224840773 grey

POFUT1 0.423793731856384 -0.423793731856384 -0.335975864148601 0.00414823825948194 0.00414823825948194 0.0257666357095643 blue

PKN3 0.414262021023729 -0.414262021023729 -0.150476371457022 0.00518014548248221 0.00518014548248221 0.329570893433906 turquoise

C3orf18 0.417424548348384 -0.417424548348384 -0.0164721267012285 0.00481541999310248 0.00481541999310248 0.915482924783582 turquoise

COL1A1 0.41169995542722 -0.41169995542722 0.153557255396366 0.00549302623406553 0.00549302623406553 0.319649431508486 turquoise

CLIP3 0.412723498195595 -0.412723498195595 0.185492977772326 0.00536611307114899 0.00536611307114899 0.228012324211843 turquoise

SLC23A3 0.415598388405768 -0.415598388405768 -0.0597747949582008 0.00502319219364317 0.00502319219364317 0.699917756020998 grey

CDC16 0.413891632660255 -0.413891632660255 0.162210771973589 0.00522439531387166 0.00522439531387166 0.292801532877364 turquoise

PJA1 0.421848199890017 -0.421848199890017 0.232313625220684 0.00434289012050464 0.00434289012050464 0.129145998189262 turquoise

CLSTN1 0.417775832540465 -0.417775832540465 -0.0124435435452725 0.00477632339453274 0.00477632339453274 0.936103688611857 turquoise

HHIPL2 0.412225464036836 -0.412225464036836 -0.161659024317779 0.00542754403988668 0.00542754403988668 0.294468440028017 grey

TM9SF2 0.411530559973573 -0.411530559973573 0.154049966368771 0.00551428003980508 0.00551428003980508 0.318080394038003 grey

SRSF11 0.418852315548255 -0.418852315548255 0.162187740857923 0.00465823326708781 0.00465823326708781 0.292870990759246 turquoise

METTL17 0.416133908826104 -0.416133908826104 -0.0320151226531253 0.00496146889710709 0.00496146889710709 0.8365538697263 brown

GYS1 0.414969182733016 -0.414969182733016 -0.0952773089350812 0.00509656721762241 0.00509656721762241 0.538418625434226 blue

CEP131 0.418553211645805 -0.418553211645805 -0.145619969642841 0.0046907866374462 0.0046907866374462 0.345595777640739 blue

DOCK7 0.416615547116964 -0.416615547116964 0.167578596283661 0.0049065214579197 0.0049065214579197 0.27690370756459 turquoise

UGGT2 0.413467152523744 -0.413467152523744 -0.0695155097721866 0.00527551168697363 0.00527551168697363 0.653877431628942 turquoise

TSC1 0.419445374346593 -0.419445374346593 -0.019929527524794 0.00459426922243473 0.00459426922243473 0.897828921735274 grey

SCRIB 0.415932714889794 -0.415932714889794 0.0149054313926829 0.00498458027714931 0.00498458027714931 0.923496465690437 brown

PTS 0.413699004645338 -0.413699004645338 0.126449664560389 0.00524753811739052 0.00524753811739052 0.413405140881842 turquoise

SACS 0.419970266743246 -0.419970266743246 -0.00193797445084834 0.00453829788070625 0.00453829788070625 0.990038676646333 turquoise

HIST1H2BE 0.420048957815314 -0.420048957815314 -0.227915617771932 0.00452995824712792 0.00452995824712792 0.136761271563063 brown

ISOC2 0.418078026001174 -0.418078026001174 0.0270341809112698 0.00474291215004428 0.00474291215004428 0.861713202146038 turquoise

TMEM50B 0.412454852721876 -0.412454852721876 0.217812748532876 0.00539917408780466 0.00539917408780466 0.155511399346043 blue

LLGL1 0.415277519541439 -0.415277519541439 -0.031550903330462 0.0050604946205778 0.0050604946205778 0.838892299628417 turquoise

CAPZB 0.418096096018345 -0.418096096018345 0.00271827465008689 0.00474092075877174 0.00474092075877174 0.986028217813666 turquoise

DLG3 0.413061106894102 -0.413061106894102 -0.0927732274952374 0.00532481489819635 0.00532481489819635 0.549195530199189 turquoise

USP35 0.413359218387012 -0.413359218387012 -0.173570069049382 0.00528857843320165 0.00528857843320165 0.259841055879685 brown

KDM5C 0.418210251205608 -0.418210251205608 0.0628132754500753 0.00472835722484553 0.00472835722484553 0.685432152711578 turquoise

GMFB 0.41192414509649 -0.41192414509649 0.272244737808285 0.0054650070230497 0.0054650070230497 0.0738052992725765 turquoise

AGPAT5 0.411682799046868 -0.411682799046868 0.138641275894877 0.00549517557370773 0.00549517557370773 0.369445594084618 blue

DNAJB7 0.414312664552696 -0.414312664552696 -0.0372683552631609 0.00517412059253961 0.00517412059253961 0.810193126632902 grey

EML1 0.416162090746622 -0.416162090746622 0.0572768418284108 0.00495823907376763 0.00495823907376763 0.711906056232142 turquoise

SEC16A 0.416354048456299 -0.416354048456299 0.0291914918763279 0.0049362882210209 0.0049362882210209 0.850798248428532 turquoise

DNAJC1 0.413793697244132 -0.413793697244132 0.0530170268648022 0.00523615038973944 0.00523615038973944 0.732507768226358 turquoise

INTS8 0.408588364568421 -0.408588364568421 0.206290262880328 0.00589500823476343 0.00589500823476343 0.179115285961857 turquoise

BIVM 0.409608714113566 -0.409608714113566 -0.181470754308796 0.00576046452065595 0.00576046452065595 0.238435470374569 grey

TANC1 0.411927483724992 -0.411927483724992 0.353020959794091 0.00546459070249984 0.00546459070249984 0.0187481844198843 turquoise

MMP2 0.407792612501335 -0.407792612501335 0.160682697269676 0.00600182224392463 0.00600182224392463 0.297433052577413 turquoise

GAL3ST2 0.408195505223021 -0.408195505223021 -0.186118005987507 0.00594753372874569 0.00594753372874569 0.226421170583775 brown

MATN2 0.410255000810867 -0.410255000810867 0.039543356347574 0.00567663184316756 0.00567663184316756 0.798839445279892 turquoise

CCNB2 0.406770299087371 -0.406770299087371 -0.105515364266228 0.00614151036300618 0.00614151036300618 0.495449414951107 turquoise

ZNF700 0.409630883132802 -0.409630883132802 -0.250120082273854 0.00575757116472147 0.00575757116472147 0.101527997696194 grey

PLOD1 0.406588308383687 -0.406588308383687 -0.0287660484491296 0.00616667097301941 0.00616667097301941 0.852948673673495 turquoise

KDELR1 0.409377094565047 -0.409377094565047 0.0809190518297032 0.00579076961740064 0.00579076961740064 0.601557848818137 turquoise

CD276 0.409105154252874 -0.409105154252874 0.0164068998274533 0.00582652695319618 0.00582652695319618 0.915816396250946 turquoise

SRRM1 0.411805373511454 -0.411805373511454 0.268549258936358 0.005479835567411 0.005479835567411 0.0779662570598331 turquoise

KLHL42 0.41067538190393 -0.41067538190393 -0.164524920662023 0.00562267325463837 0.00562267325463837 0.285876793233549 brown

UBASH3B 0.406736176030776 -0.406736176030776 -0.146111424088161 0.00614622115361001 0.00614622115361001 0.343952689920528 turquoise

ADHFE1 0.413978952381506 -0.413978952381506 0.23914310385851 0.00521393380492627 0.00521393380492627 0.117956084642801 turquoise

ANAPC4 0.407606184269427 -0.407606184269427 0.260423713253938 0.00602708808261613 0.00602708808261613 0.0877617171157983 turquoise

ACSF2 0.40780701140427 -0.40780701140427 0.12960499854764 0.00599987465118835 0.00599987465118835 0.4017512107192 turquoise

PPL 0.405533447088483 -0.405533447088483 -0.157930731848731 0.00631427650758864 0.00631427650758864 0.305892487973432 brown

UBA7 0.411106220963418 -0.411106220963418 0.217818548810825 0.00556783543984559 0.00556783543984559 0.155500121085119 turquoise

PTPRH 0.406506826816566 -0.406506826816566 -0.169837143182824 0.00617796492769157 0.00617796492769157 0.270387391220335 brown

ACBD5 0.41079865810849 -0.41079865810849 0.222033239948356 0.00560693474427876 0.00560693474427876 0.147462187100233 turquoise

SLC12A2 0.411979232396593 -0.411979232396593 0.358092066825853 0.00545814126877764 0.00545814126877764 0.0170014461609196 turquoise

PPP2CB 0.404591298045266 -0.404591298045266 0.247282331376897 0.00644869073970473 0.00644869073970473 0.105596063228217 turquoise

ZNF212 0.406803304124838 -0.406803304124838 0.002521335691495 0.0061369568990297 0.0061369568990297 0.987040384079583 grey

C18orf21 0.407806179025262 -0.407806179025262 -0.169310932303083 0.00599998722366479 0.00599998722366479 0.271896471925288 brown

SOX8 0.405563705670698 -0.405563705670698 -0.0908376889467949 0.00631000015933034 0.00631000015933034 0.557595211538146 brown

CAMK2D 0.409630809974925 -0.409630809974925 0.310610802247976 0.00575758071073413 0.00575758071073413 0.0401589047181894 turquoise

RTBDN 0.408522756076418 -0.408522756076418 -0.334295574779793 0.00590375200338677 0.00590375200338677 0.0265637875193572 brown

PCBP1 0.405174657129702 -0.405174657129702 0.0523707520945282 0.00636517502672672 0.00636517502672672 0.735650053496659 blue

MRPL57 0.403337488614114 -0.403337488614114 -0.276145847130986 0.00663141073815984 0.00663141073815984 0.0696047275193327 brown

ZHX3 0.409420485004384 -0.409420485004384 0.0431176472066097 0.00578508189412076 0.00578508189412076 0.781084837592255 blue

KIAA0355 0.40459200516329 -0.40459200516329 0.244826313533996 0.00644858893406859 0.00644858893406859 0.109216481521975 turquoise

ALKBH2 0.411495429200879 -0.411495429200879 -0.295904089182957 0.00551869678898444 0.00551869678898444 0.0511499053803226 brown

HIST1H3F 0.404716351010566 -0.404716351010566 0.139182710658717 0.00643070818346958 0.00643070818346958 0.367560729818796 turquoise

TGFB3 0.411034627928529 -0.411034627928529 -0.199405411801008 0.00557691556320803 0.00557691556320803 0.194388674027269 turquoise

INO80D 0.410444283503447 -0.410444283503447 -0.0159027022015198 0.00565228076689872 0.00565228076689872 0.918394576994399 brown

PCMT1 0.405718702441347 -0.405718702441347 0.264603696021812 0.00628813432164167 0.00628813432164167 0.0826096669709631 turquoise

SLC35E2B 0.405792081585032 -0.405792081585032 0.160182949165165 0.00627780545455131 0.00627780545455131 0.298957948266338 turquoise

ANTXR1 0.402420377225732 -0.402420377225732 0.0841983438675631 0.00676788737793682 0.00676788737793682 0.586856777191375 turquoise

MEOX2 0.407390985295612 -0.407390985295612 0.248258041591498 0.00605636784189101 0.00605636784189101 0.104183510265958 turquoise

BCS1L 0.410312810250154 -0.410312810250154 -0.02899455962257 0.00566918505075351 0.00566918505075351 0.851793520465719 turquoise

WDR26 0.408620411314588 -0.408620411314588 0.283239204171901 0.00589074139329377 0.00589074139329377 0.062453334088007 turquoise

COL13A1 0.401282582536044 -0.401282582536044 -0.0786572392503911 0.00694057911901843 0.00694057911901843 0.611789865490898 turquoise

MXD4 0.409005763683221 -0.409005763683221 0.153139532244329 0.00583964355517944 0.00583964355517944 0.320983485588054 turquoise

TRIM35 0.404564220675117 -0.404564220675117 0.0990646030039927 0.00645259018786571 0.00645259018786571 0.522315925632936 blue

C1QTNF8 0.400274251917711 -0.400274251917711 -0.00483630445683569 0.00709679532976897 0.00709679532976897 0.975144312464511 grey

CRISPLD2 0.400826502199239 -0.400826502199239 -0.104754885373248 0.00701086509866097 0.00701086509866097 0.498579265989552 turquoise

MOSPD3 0.408329028513986 -0.408329028513986 -0.183329207147228 0.00592963619763101 0.00592963619763101 0.233579921332083 brown

DAPK3 0.4006385155242 -0.4006385155242 0.0970169595310305 0.00704001423296008 0.00704001423296008 0.530992328502107 turquoise

C11orf49 0.410198522686467 -0.410198522686467 0.324320405683665 0.00568391534825556 0.00568391534825556 0.0317299441726767 turquoise

RABGGTB 0.405558501177546 -0.405558501177546 0.129169337705639 0.00631073551475176 0.00631073551475176 0.403348878954848 turquoise

B4GALT1 0.404815722829449 -0.404815722829449 0.101994114386777 0.00641644957311799 0.00641644957311799 0.51002615668553 turquoise

C10orf88 0.401785589790077 -0.401785589790077 -0.0130607457570695 0.00686376922317254 0.00686376922317254 0.932941451185892 turquoise

MRPL3 0.402280205349455 -0.402280205349455 0.242070837841977 0.00678895935491349 0.00678895935491349 0.113390075923367 turquoise

NFATC4 0.399809942383761 -0.399809942383761 -0.12285590841642 0.00716974546173335 0.00716974546173335 0.426910340655427 turquoise

PEX1 0.407555492659993 -0.407555492659993 -0.119949366335307 0.00603397404782767 0.00603397404782767 0.438011879165375 brown

BRD2 0.408401217960983 -0.408401217960983 0.310094896674221 0.0059199793763113 0.0059199793763113 0.0405087329109646 turquoise

MAFB 0.398761971333351 -0.398761971333351 0.0564884271750674 0.00733678536415203 0.00733678536415203 0.715704312205074 turquoise

NCOA5 0.407443578618575 -0.407443578618575 0.0617360860895751 0.00604920067173715 0.00604920067173715 0.690555109046638 turquoise

MTCH2 0.404682670332082 -0.404682670332082 0.0213698741836902 0.00643554716668711 0.00643554716668711 0.890488164280496 turquoise

TIA1 0.40505641153865 -0.40505641153865 0.271097377119006 0.00638202728738489 0.00638202728738489 0.0750780080146831 turquoise

NAV2 0.406263825667498 -0.406263825667498 -0.150847375629386 0.00621175332655525 0.00621175332655525 0.328366064168123 turquoise

DNMT1 0.397564166849036 -0.397564166849036 0.320146155293381 0.00753181769621403 0.00753181769621403 0.034125314834413 turquoise

MAGOH 0.404436110263211 -0.404436110263211 0.220361741902664 0.00647106726111195 0.00647106726111195 0.150612492626981 turquoise

C4orf46 0.403386818900614 -0.403386818900614 -0.0955503798620949 0.00662413788724262 0.00662413788724262 0.53724961459639 brown

FAM162A 0.399317149075904 -0.399317149075904 -0.101454695444634 0.00724787935036494 0.00724787935036494 0.51227809940306 turquoise

NVL 0.405869241337085 -0.405869241337085 0.057899028289059 0.00626696030165184 0.00626696030165184 0.708913469968379 turquoise

TTYH3 0.402083972855263 -0.402083972855263 0.176659541122231 0.00681855431485649 0.00681855431485649 0.251322975854997 turquoise

DGCR8 0.400486115253321 -0.400486115253321 0.0124020217254611 0.00706372225404196 0.00706372225404196 0.936316461204158 turquoise

RBFOX2 0.400878580428279 -0.400878580428279 0.245390457269443 0.00700280836578918 0.00700280836578918 0.108376624866269 turquoise

NPIPB15 0.404927625603004 -0.404927625603004 -0.135005965682067 0.00640042574281317 0.00640042574281317 0.38225035229731 turquoise

SHKBP1 0.402207522214499 -0.402207522214499 -0.230744231311267 0.00679990812583438 0.00679990812583438 0.131826134144958 brown

C16orf46 0.404748940490423 -0.404748940490423 -0.229779798264433 0.00642602897795738 0.00642602897795738 0.133493624865956 grey

IL21 0.398984634185903 -0.398984634185903 -0.202700437581626 0.00730101561789168 0.00730101561789168 0.186967942337789 brown

PUSL1 0.402332924043467 -0.402332924043467 -0.0279732314868794 0.00678102752154319 0.00678102752154319 0.856958807082522 blue

COTL1 0.396414477912886 -0.396414477912886 -0.225455231152976 0.00772321196786055 0.00772321196786055 0.141164549789461 brown

SRSF5 0.398999428859105 -0.398999428859105 0.252183508415004 0.00729864427333608 0.00729864427333608 0.0986462905352428 turquoise

POGZ 0.400490633667283 -0.400490633667283 0.202128312860129 0.00706301835847932 0.00706301835847932 0.188241739524096 turquoise

LTBR 0.402129223311882 -0.402129223311882 -0.0214798491763701 0.00681171994122563 0.00681171994122563 0.889928038135786 blue

CHIC1 0.398421425483742 -0.398421425483742 -0.237167923849586 0.00739178544994107 0.00739178544994107 0.121114148350275 brown

SH2B3 0.404401434763395 -0.404401434763395 0.134551016139573 0.00647607630837428 0.00647607630837428 0.383871083919732 turquoise

FOXI3 0.398900283496265 -0.398900283496265 -0.163427760381029 0.00731454838406915 0.00731454838406915 0.289146466860906 brown

CDKN2D 0.396999230316507 -0.396999230316507 -0.047858764944776 0.00762534710681326 0.00762534710681326 0.757704309098363 turquoise

RBSN 0.400511719093442 -0.400511719093442 0.0938574432658879 0.00705973439352585 0.00705973439352585 0.544516786946948 turquoise

USP53 0.404251467691155 -0.404251467691155 0.0869238634510043 0.00649777850694985 0.00649777850694985 0.574761813557196 turquoise

PITRM1 0.401780717231091 -0.401780717231091 0.0827111423831354 0.00686450972558816 0.00686450972558816 0.593504031301363 turquoise

PDK2 0.40450030329535 -0.40450030329535 -0.0918628985955204 0.00646180312212024 0.00646180312212024 0.553138595369696 grey

FAP 0.394991381607794 -0.394991381607794 0.0278615815370537 0.00796592564816959 0.00796592564816959 0.857523830081818 turquoise

PHLDA2 0.396560495555537 -0.396560495555537 -0.21695793614167 0.00769867325017976 0.00769867325017976 0.157180075596265 turquoise

IL17RD 0.396520768400011 -0.396520768400011 -0.101738251368383 0.00770534284626579 0.00770534284626579 0.51109369980102 turquoise

SMYD3 0.403171016183146 -0.403171016183146 -0.115459075497283 0.00665600514408261 0.00665600514408261 0.455472886258371 turquoise

OR2T5 0.404581798734088 -0.404581798734088 -0.179701286370368 0.00645005851551262 0.00645005851551262 0.243121913239889 grey

ZNF185 0.39657438428481 -0.39657438428481 -0.330616096924972 0.00769634271623926 0.00769634271623926 0.0283812422293006 brown

WEE1 0.394970346867139 -0.394970346867139 -0.0315959404197593 0.00796956198621755 0.00796956198621755 0.83866537216375 turquoise

RNF144A 0.396874125984386 -0.396874125984386 -0.188447605914452 0.00764619417636161 0.00764619417636161 0.220557962764307 turquoise

JADE3 0.396542495913986 -0.396542495913986 -0.157605602817784 0.00770169450329971 0.00770169450329971 0.30690197499273 brown

DIDO1 0.403532410968202 -0.403532410968202 0.162295040723921 0.00660271326847002 0.00660271326847002 0.292547483677032 turquoise

TTC9 0.398952927842366 -0.398952927842366 -0.294404531475362 0.00730609987036336 0.00730609987036336 0.0523951624052221 grey

CCDC106 0.395270349036858 -0.395270349036858 -0.159007583319655 0.00791783493874339 0.00791783493874339 0.30256416147552 brown

TNPO2 0.39750445722093 -0.39750445722093 0.137780817994876 0.00754165599917873 0.00754165599917873 0.372452942392088 turquoise

ADTRP 0.399521967978249 -0.399521967978249 -0.355614754209627 0.00721531573561684 0.00721531573561684 0.0178367449407842 grey

GALNT10 0.396805966819463 -0.396805966819463 0.154740939465035 0.0076575727462504 0.0076575727462504 0.315888198352771 turquoise

RHOBTB1 0.401579193552319 -0.401579193552319 0.327698018769858 0.00689519672860551 0.00689519672860551 0.0298949600436474 turquoise

PI4KA 0.402690387330881 -0.402690387330881 0.126149435769938 0.00672745644853925 0.00672745644853925 0.414523964369846 turquoise

GAS6 0.395259934823654 -0.395259934823654 0.163230110268041 0.00791962571477958 0.00791962571477958 0.289738059189162 turquoise

BMP15 0.392948334107702 -0.392948334107702 -0.160329020447375 0.00832586711776753 0.00832586711776753 0.298511717615368 brown

ARMCX2 0.394417423041537 -0.394417423041537 0.19023567176241 0.00806566254358416 0.00806566254358416 0.216129488066055 turquoise

CRLS1 0.395146023722725 -0.395146023722725 0.0790584726387913 0.00793923612791001 0.00793923612791001 0.609969349664346 turquoise

SLIT2 0.39397524857763 -0.39397524857763 -0.0435893274103995 0.00814323207767152 0.00814323207767152 0.77874983881475 grey

EVI5 0.395961896608802 -0.395961896608802 0.314751264820936 0.00779969917972872 0.00779969917972872 0.0374398222488429 turquoise

RBP3 0.396935954977 -0.396935954977 -0.345203757643156 0.00763588500592998 0.00763588500592998 0.0217358320205183 brown

RBBP8NL 0.393927592078512 -0.393927592078512 -0.213374381866944 0.0081516306127665 0.0081516306127665 0.164317965270737 brown

FGFR2 0.392799733564798 -0.392799733564798 -0.0175980024433313 0.00835258569760696 0.00835258569760696 0.909729208207495 turquoise

DYNC1H1 0.399534241028006 -0.399534241028006 0.321607336563938 0.00721336849978442 0.00721336849978442 0.0332704298816076 turquoise

GYPE 0.396014315718414 -0.396014315718414 -0.183560565119536 0.00779080688820331 0.00779080688820331 0.23298021980599 brown

PHPT1 0.397370507591823 -0.397370507591823 -0.106945134356525 0.00756376715722015 0.00756376715722015 0.489592500740849 blue

LAMC1 0.394841057482646 -0.394841057482646 0.0202552020378122 0.00799194409579218 0.00799194409579218 0.89616835500468 brown

BTBD6 0.397728568807902 -0.397728568807902 0.0146936902419568 0.00750478659935438 0.00750478659935438 0.924580099255403 turquoise

DDIT4L 0.395694016130814 -0.395694016130814 -0.165839585579487 0.00784527890077151 0.00784527890077151 0.281990767194829 turquoise

SRSF6 0.394775320453159 -0.394775320453159 0.102974003891037 0.0080033450339249 0.0080033450339249 0.505948164936786 turquoise

AVPR1A 0.390959108061742 -0.390959108061742 -0.14801794299187 0.00868970891339952 0.00868970891339952 0.337624191655836 brown

TPGS2 0.393191619053426 -0.393191619053426 0.0646318774091822 0.00828228317844136 0.00828228317844136 0.676814796382322 turquoise

ARGLU1 0.394648488359932 -0.394648488359932 0.216804488091679 0.00802538149826264 0.00802538149826264 0.157481000297743 turquoise

SLC35B3 0.39963641623003 -0.39963641623003 0.223560165399544 0.00719717506531037 0.00719717506531037 0.144627028780793 turquoise

IAH1 0.394916616743606 -0.394916616743606 -0.0523031440004158 0.00797885697423386 0.00797885697423386 0.735979021684406 brown

KRTAP4-12 0.393837594949357 -0.393837594949357 -0.282822691058618 0.00816751123746598 0.00816751123746598 0.06285638480492 brown

CCDC60 0.39321041636523 -0.39321041636523 -0.153484785154472 0.008278923883415 0.008278923883415 0.319880623822839 brown

GPS1 0.398743642960278 -0.398743642960278 0.0514012222624495 0.0073397364676404 0.0073397364676404 0.740372044214589 turquoise

ITPKA 0.39563709840141 -0.39563709840141 -0.103050881455816 0.00785499297906959 0.00785499297906959 0.505628925830829 blue

LIMK1 0.400086451453143 -0.400086451453143 0.0970434157614402 0.00712622396043001 0.00712622396043001 0.53087977888371 turquoise

ACADL 0.393596012236576 -0.393596012236576 0.028125285149611 0.00821027219024076 0.00821027219024076 0.856189428085597 blue

DCANP1 0.391329084292987 -0.391329084292987 -0.213507526916111 0.00862101992095293 0.00862101992095293 0.164048623030906 brown

KIAA1324L 0.399363303014758 -0.399363303014758 0.14842084359335 0.00724053038860872 0.00724053038860872 0.336296093188811 turquoise

CSRNP2 0.394094379652995 -0.394094379652995 0.120243319445702 0.00812227016687111 0.00812227016687111 0.436881906503306 turquoise

ARSJ 0.391320106382111 -0.391320106382111 -0.0569928793013061 0.00862268118806623 0.00862268118806623 0.713273282249471 turquoise

BNIP3 0.390512106164958 -0.390512106164958 -0.192323765184809 0.00877332650597181 0.00877332650597181 0.211036497219204 turquoise

ALX1 0.393619791684352 -0.393619791684352 -0.121283438216167 0.00820605460078837 0.00820605460078837 0.432896648336808 brown

CPXM1 0.396465013383621 -0.396465013383621 -0.108787556081132 0.00771471169481391 0.00771471169481391 0.482098505942716 grey

ECHDC2 0.397056598591906 -0.397056598591906 0.344332107445838 0.0076158038305418 0.0076158038305418 0.0220924263537851 turquoise

RAC3 0.390135902574371 -0.390135902574371 -0.226749822444978 0.00884423623893323 0.00884423623893323 0.138834752115483 blue

CAMK2G 0.394115177395516 -0.394115177395516 0.247654880901311 0.00811861543999074 0.00811861543999074 0.105054998799147 turquoise

YY1 0.394989517100141 -0.394989517100141 0.316455693592418 0.00796624791330947 0.00796624791330947 0.0363652828804756 turquoise

TBCD 0.397416003611931 -0.397416003611931 0.111566752572681 0.00755625082617758 0.00755625082617758 0.470908949873488 turquoise

AIPL1 0.38851154273679 -0.38851154273679 -0.178567711333828 0.00915610017415861 0.00915610017415861 0.246156764311919 grey

NXF1 0.397394517327773 -0.397394517327773 0.261385119404345 0.0075597997393985 0.0075597997393985 0.0865551721910282 turquoise

PLEKHA4 0.388488745381963 -0.388488745381963 0.14193480238328 0.00916054351972146 0.00916054351972146 0.358069586235945 turquoise

C5orf15 0.390379258110821 -0.390379258110821 0.0916134113495373 0.00879831061956417 0.00879831061956417 0.554221572694876 turquoise

PPARD 0.396333024747762 -0.396333024747762 0.119811013193435 0.00773692974456863 0.00773692974456863 0.438544276726843 turquoise

POC1B 0.396924781399247 -0.396924781399247 -0.193404142354237 0.00763774716376146 0.00763774716376146 0.208434487954835 brown

ITGA11 0.390371469708239 -0.390371469708239 -0.17414912159713 0.0087997772467196 0.0087997772467196 0.258230045885682 turquoise

POM121 0.395976369704222 -0.395976369704222 -0.0971125094771825 0.00779724311251993 0.00779724311251993 0.530585896675118 turquoise

KRTAP2-3 0.389536038790197 -0.389536038790197 -0.206138624642943 0.00895832353040442 0.00895832353040442 0.179442131785662 brown

LSM8 0.391362452602211 -0.391362452602211 0.360588362907972 0.00861484788543923 0.00861484788543923 0.0161934439629157 turquoise

SPATA21 0.393161613372125 -0.393161613372125 0.124892712524402 0.00828764797240232 0.00828764797240232 0.419225921191527 turquoise

ACE 0.392519778066592 -0.392519778066592 -0.355593160346858 0.00840312287409293 0.00840312287409293 0.017844175543758 brown

TBC1D23 0.391320200926491 -0.391320200926491 0.140430862726494 0.00862266369219584 0.00862266369219584 0.363237661564246 turquoise

ANKRA2 0.39568950716531 -0.39568950716531 0.0480585542337757 0.00784604806198361 0.00784604806198361 0.756723575178188 grey

TSPAN10 0.391739708964197 -0.391739708964197 -0.129364979305956 0.0085453322188225 0.0085453322188225 0.402630963132097 brown

GFY 0.392538657093216 -0.392538657093216 -0.216140817743017 0.00839970659236661 0.00839970659236661 0.158787359935843 brown

GSS 0.395294457436867 -0.395294457436867 0.204020305129516 0.00791369071946909 0.00791369071946909 0.184052776317658 turquoise

PTGS2 0.390835556831623 -0.390835556831623 -0.163973903469183 0.00871275188733461 0.00871275188733461 0.287515870353772 grey

SUSD5 0.386839036612636 -0.386839036612636 -0.130831707941557 0.0094870438039261 0.0094870438039261 0.397272293154071 turquoise

NREP 0.38851075740486 -0.38851075740486 0.140452768579684 0.00915625320938109 0.00915625320938109 0.363162063914987 turquoise

ABCG4 0.388298373591474 -0.388298373591474 -0.133755103673088 0.00919772061431614 0.00919772061431614 0.386716206198306 brown

HMMR 0.393861579558497 -0.393861579558497 -0.136426584144975 0.00816327637964919 0.00816327637964919 0.377215571350792 grey

PTGES3 0.388038715592519 -0.388038715592519 0.0933379603199588 0.00924863750877349 0.00924863750877349 0.546756141017617 turquoise

FUS 0.386078329790022 -0.386078329790022 0.0135903210447699 0.00964093539545969 0.00964093539545969 0.930228981553396 turquoise

DPP9 0.387948455385346 -0.387948455385346 0.122487399279157 0.00926639349881848 0.00926639349881848 0.428309046727045 turquoise

VWA2 0.393573898904848 -0.393573898904848 -0.307088525868094 0.00821419594969387 0.00821419594969387 0.042597158631805 brown

ASH1L 0.386591776366793 -0.386591776366793 0.177744307946755 0.00953683153845111 0.00953683153845111 0.248377190655437 turquoise

HEMK1 0.386705466606661 -0.386705466606661 -0.0846773959757755 0.00951391133287803 0.00951391133287803 0.584722688709936 grey

CCDC93 0.391334996670896 -0.391334996670896 0.260753521362999 0.00861992604871932 0.00861992604871932 0.0873463504813921 turquoise

SEMA4C 0.387994088418722 -0.387994088418722 0.105491189272463 0.0092574129078052 0.0092574129078052 0.49554875445764 turquoise

RASGRF2 0.389098440535593 -0.389098440535593 -0.245975465385909 0.00904234651160527 0.00904234651160527 0.1075109177771 brown

TGM7 0.384745887787561 -0.384745887787561 -0.154103110056763 0.00991565917085757 0.00991565917085757 0.317911449092366 brown

EP400 0.385988405188221 -0.385988405188221 -0.158106188814967 0.00965926814139838 0.00965926814139838 0.305348597404362 grey

TTF1 0.389523899600727 -0.389523899600727 -0.0696152507304849 0.00896064527735646 0.00896064527735646 0.653412148435304 brown

WTIP 0.384342468009589 -0.384342468009589 0.0475201866545161 0.0100001504463483 0.0100001504463483 0.759367194040279 turquoise

LTBP4 0.386524271234595 -0.386524271234595 -0.114777173196217 0.00955046315784395 0.00955046315784395 0.458157150975209 turquoise

CUEDC1 0.391343516981429 -0.391343516981429 -0.178985657448629 0.00861834988289416 0.00861834988289416 0.245034858612601 grey

AQP11 0.39080609918508 -0.39080609918508 0.0253129048383671 0.00871825365892461 0.00871825365892461 0.870440422883164 grey

ZNF664 0.386140233406224 -0.386140233406224 0.31158251701775 0.00962833260604472 0.00962833260604472 0.0395067043757571 turquoise

PRKCI 0.390203473091749 -0.390203473091749 0.112126955420421 0.00883146380762765 0.00883146380762765 0.468670323280709 turquoise

ZNF638 0.390466062002337 -0.390466062002337 0.176504794495982 0.00878197889204091 0.00878197889204091 0.25174511114179 blue

POU3F1 0.383629696950008 -0.383629696950008 -0.178754127074222 0.0101509420871772 0.0101509420871772 0.24565593483052 brown

SNX1 0.386582496734757 -0.386582496734757 0.280837747065813 0.00953870442529585 0.00953870442529585 0.0648057258944175 turquoise

RPRD1A 0.388987057244002 -0.388987057244002 0.202446443624664 0.00906384107752852 0.00906384107752852 0.187532683399992 turquoise

ABCG5 0.38307366729386 -0.38307366729386 -0.279169646216986 0.0102699243840542 0.0102699243840542 0.0664808109279215 brown

CCDC62 0.38170261105922 -0.38170261105922 -0.0473003795095795 0.0105684327064489 0.0105684327064489 0.760447324181445 brown

SPIN1 0.383706609294208 -0.383706609294208 0.273942757930211 0.0101345774257116 0.0101345774257116 0.0719530341902177 turquoise

CNOT10 0.388426475244168 -0.388426475244168 0.160522800337796 0.00917268980662027 0.00917268980662027 0.297920404569121 turquoise

PRSS21 0.383073065954525 -0.383073065954525 -0.29741292087827 0.0102700537065974 0.0102700537065974 0.0499211581668459 brown

BET1 0.3872713274386 -0.3872713274386 -0.0963711389662845 0.0094005352348013 0.0094005352348013 0.533743379895221 brown

SEC23A 0.386291649302483 -0.386291649302483 0.155723100342756 0.00959756587769509 0.00959756587769509 0.312788669449728 turquoise

TMTC3 0.385464216941646 -0.385464216941646 0.158176248996151 0.00976673040805283 0.00976673040805283 0.305131594064383 turquoise

TMEM138 0.387506563747588 -0.387506563747588 0.0245072140622477 0.00935374602918914 0.00935374602918914 0.874530734858834 turquoise

ZBTB33 0.385332319783618 -0.385332319783618 0.0454419555204436 0.00979393134013136 0.00979393134013136 0.769597408963096 grey

COL12A1 0.379855765165103 -0.379855765165103 0.172964749382239 0.0109822625258933 0.0109822625258933 0.261532298858518 turquoise

NAA25 0.385136765636581 -0.385136765636581 0.226739347729133 0.00983437984527721 0.00983437984527721 0.138853487581804 turquoise

BOD1 0.382701162065146 -0.382701162065146 0.251421416441112 0.0103503024296599 0.0103503024296599 0.0997031797822695 turquoise

PHYKPL 0.387412739702393 -0.387412739702393 0.221733905688247 0.00937238385855758 0.00937238385855758 0.148022750269429 turquoise

CDA 0.382737732990223 -0.382737732990223 -0.277417615257486 0.0103423874622253 0.0103423874622253 0.068276992253528 brown

GPX7 0.378768302567018 -0.378768302567018 0.135930865444656 0.0112323641957061 0.0112323641957061 0.378967942498738 turquoise

STT3A 0.383900745987841 -0.383900745987841 0.144128942929524 0.0100933715336546 0.0100933715336546 0.350610188221644 turquoise

IFNGR2 0.382803173264975 -0.382803173264975 0.364285007117276 0.0103282373145713 0.0103282373145713 0.015056773582572 turquoise

MRPL12 0.380688441912813 -0.380688441912813 0.190977921954291 0.0107939953468481 0.0107939953468481 0.214309416043505 turquoise

AIFM2 0.384899508024817 -0.384899508024817 -0.0307290673374799 0.00988364679292297 0.00988364679292297 0.843035517475222 blue

FNDC1 0.380143892796921 -0.380143892796921 -0.0304427941445571 0.0109168016228917 0.0109168016228917 0.844479729789032 turquoise

POLH 0.381663156986097 -0.381663156986097 -0.045221916517337 0.0105771316125468 0.0105771316125468 0.770682863823706 turquoise

OXSR1 0.384413471375676 -0.384413471375676 0.185383202727195 0.00998523502678961 0.00998523502678961 0.22829257252414 turquoise

P3H4 0.378543855673311 -0.378543855673311 -0.066835058167577 0.0112845862038211 0.0112845862038211 0.666429593931235 turquoise

SMO 0.38425562933381 -0.38425562933381 0.0965921421608239 0.0100184183197734 0.0100184183197734 0.532801175090033 turquoise

PCDHB5 0.383276446218818 -0.383276446218818 0.0387154443897402 0.0102263948123162 0.0102263948123162 0.802966633811921 grey

BOD1L1 0.386057422177967 -0.386057422177967 0.271439998489414 0.00964519511919792 0.00964519511919792 0.0746961603706856 turquoise

POLDIP2 0.380229765111064 -0.380229765111064 0.251844619784552 0.0108973566808356 0.0108973566808356 0.0991152008571396 turquoise

HSPG2 0.380099450826743 -0.380099450826743 0.0067300481445019 0.0109268767318217 0.0109268767318217 0.965416661830504 turquoise

GPR162 0.378133622599398 -0.378133622599398 -0.18572961174206 0.0113805719275806 0.0113805719275806 0.227409017211649 brown

UBTD1 0.378705230813365 -0.378705230813365 0.0885226850094988 0.0112470181550323 0.0112470181550323 0.567720091567058 turquoise

TKTL1 0.383151452143237 -0.383151452143237 -0.204870599263735 0.0102532079371627 0.0102532079371627 0.182192011936368 brown

COLGALT1 0.380495771886377 -0.380495771886377 0.280719971765232 0.0108373101774144 0.0108373101774144 0.0649228822919638 turquoise

FAM177B 0.378405011389099 -0.378405011389099 0.0503392258886877 0.01131699495023 0.01131699495023 0.745555219050942 blue

YBX3 0.383190860178337 -0.383190860178337 -0.121336620050892 0.0102447478155599 0.0102447478155599 0.432693426346283 blue

VMP1 0.376329130352802 -0.376329130352802 0.225195434317673 0.011811120450367 0.011811120450367 0.141635558681855 turquoise

PCDHB16 0.376863015532244 -0.376863015532244 0.154725797736312 0.0116823102666656 0.0116823102666656 0.315936134611654 turquoise

TRIM59 0.378226383292824 -0.378226383292824 -0.196121347036152 0.011358807038429 0.011358807038429 0.201989596288946 turquoise

SLC19A2 0.377004360192698 -0.377004360192698 -0.0792620221603791 0.0116484098309614 0.0116484098309614 0.609046672232899 turquoise

GBP4 0.38091718869729 -0.38091718869729 -0.297672034235357 0.0107427625763093 0.0107427625763093 0.0497125636703896 brown

DYRK2 0.380954730222884 -0.380954730222884 0.195913383955404 0.0107343742730561 0.0107343742730561 0.202477854099706 turquoise

CBX7 0.380989340116915 -0.380989340116915 0.063416524639797 0.0107266459867901 0.0107266459867901 0.682569246442995 blue

GRB10 0.379430262285144 -0.379430262285144 0.068939103739189 0.0110795487318465 0.0110795487318465 0.656568852947788 turquoise

SIPA1L3 0.377428487101763 -0.377428487101763 -0.270578019172644 0.0115471905186993 0.0115471905186993 0.0756597534699184 grey

SPTAN1 0.381464307285321 -0.381464307285321 0.193223202473886 0.0106210678113262 0.0106210678113262 0.208868698564566 turquoise

MYLK4 0.376643628141439 -0.376643628141439 -0.193767208786484 0.0117350956708675 0.0117350956708675 0.207565121970007 grey

VPS26B 0.375979244790933 -0.375979244790933 -0.0400314605982809 0.011896193819809 0.011896193819809 0.796408745594362 brown
