# Supplementary material for: Identification and development of a novel 5-gene diagnostic model based on immune infiltration analysis of osteoarthritis
Source: J Transl Med. 2021 Dec 23;19:522. doi: 10.1186/s12967-021-03183-9 (PMC8705150; doi:10.1186/s12967-021-03183-9)
Supplement: Supplementary file 3 — Additional file 3: Fig. S1. The classification accuracy and the sensitivity in the four models [file 12967_2021_3183_MOESM3_ESM.pdf]

A

GSE Integ	Real OA	Real normal	
Predict OA	20	3	
Predict normal	2	19	Totals
Totals	22	22	44
Correct	20	19	39
Sensitivity(%)	91		
Specificity(%)		86.4	

B

GSE57218	Real OA	Real normal	
Predict OA	33	0	
Predict normal	0	7	Totals
Totals	33	7	40
Correct	33	7	40
Sensitivity(%)	100		
Specificity(%)		100	

C

GSE129147	Real OA	Real normal	
Predict OA	10	0	
Predict normal	0	9	Totals
Totals	10	9	19
Correct	10	9	19
Sensitivity(%)	100		
Specificity(%)		100	

D

GSE51588	Real OA	Real normal	
Predict OA	40	1	
Predict normal	0	9	Totals
Totals	40	10	50
Correct	40	9	49
Sensitivity(%)	100		
Specificity(%)		90	
